# Supplementary material for: Calculation of the Isobaric Heat Capacities of the Liquid and Solid Phase of Organic Compounds at and around 298.15 K Based on Their “True” Molecular Volume
Source: Molecules. 2019 Apr 24;24(8):1626. doi: 10.3390/molecules24081626 (PMC6514989; doi:10.3390/molecules24081626)
Supplement: Supplementary file 1 [file molecules-24-01626-s001.zip › S03. Experimental and Calculated Cp(liq,298) Data Table to Figure 2.doc]

| **Molecule name** | **Molecular volume** | **Cp(liq,298) exp** | **Cp(liq,298) calc** | **Deviation** | **Dev. in %** |
| --- | --- | --- | --- | --- | --- |
| Water | 19.20 | 75.32 | 35.30 | 40.02 | 53.13 |
| Methanol | 36.90 | 81.92 | 67.77 | 14.15 | 17.27 |
| Formic acid | 39.90 | 99.04 | 73.27 | 25.77 | 26.02 |
| Methylamine | 40.60 | 102.10 | 74.56 | 27.54 | 26.98 |
| Methyl chloride | 42.40 | 80.80 | 77.86 | 2.94 | 3.64 |
| Formamide | 42.50 | 107.90 | 78.04 | 29.86 | 27.67 |
| Trifluoromethane | 42.90 | 86.60 | 78.78 | 7.82 | 9.03 |
| Acetonitrile | 46.20 | 91.70 | 84.83 | 6.87 | 7.49 |
| Methanethiol | 46.60 | 89.10 | 85.56 | 3.54 | 3.97 |
| Bromomethane | 46.60 | 78.70 | 85.56 | -6.86 | -8.72 |
| Oxirane | 47.00 | 87.00 | 86.30 | 0.70 | 0.81 |
| Tetrafluoromethane | 47.80 | 79.90 | 87.76 | -7.86 | -9.84 |
| Acetaldehyde | 47.90 | 89.05 | 87.95 | 1.10 | 1.24 |
| Nitromethane | 51.60 | 105.98 | 94.74 | 11.24 | 10.61 |
| Chlorodifluoromethane | 52.20 | 114.10 | 95.84 | 18.26 | 16.01 |
| Methylhydrazine | 52.30 | 134.93 | 96.02 | 38.91 | 28.84 |
| Methyl iodide | 52.80 | 82.75 | 96.94 | -14.19 | -17.14 |
| Ethanol | 53.60 | 115.90 | 98.40 | 17.50 | 15.10 |
| Chloroethylene | 53.90 | 89.45 | 98.95 | -9.50 | -10.63 |
| Dimethylether | 54.30 | 102.50 | 99.69 | 2.81 | 2.74 |
| Cyclopropane | 54.60 | 81.20 | 100.24 | -19.04 | -23.45 |
| 1,1-Difluoroethane | 54.70 | 118.40 | 100.42 | 17.98 | 15.18 |
| Carbon disulfide | 54.80 | 78.99 | 100.61 | -21.62 | -27.36 |
| Propylene | 55.90 | 102.00 | 102.62 | -0.62 | -0.61 |
| Dichloromethane | 56.60 | 100.00 | 103.91 | -3.91 | -3.91 |
| Acetic acid | 56.70 | 123.10 | 104.09 | 19.01 | 15.44 |
| Ethylamine | 56.70 | 130.00 | 104.09 | 25.91 | 19.93 |
| Chlorotrifluoromethane | 57.10 | 121.60 | 104.82 | 16.78 | 13.80 |
| Cyanoethylene | 57.20 | 98.70 | 105.01 | -6.31 | -6.39 |
| Dimethylamine | 57.20 | 136.80 | 105.01 | 31.79 | 23.24 |
| Methyl formate | 57.30 | 119.10 | 105.19 | 13.91 | 11.68 |
| Bromoethylene | 58.10 | 107.50 | 106.66 | 0.84 | 0.78 |
| Chloroethane | 59.10 | 107.80 | 108.49 | -0.69 | -0.64 |
| Tetrafluoroethene | 59.30 | 113.00 | 108.86 | 4.14 | 3.66 |
| 1,1,1-Trifluoroethane | 59.60 | 109.60 | 109.41 | 0.19 | 0.17 |
| Methylnitrate | 59.60 | 157.19 | 109.41 | 47.78 | 30.40 |
| N-Methylformamide | 59.70 | 123.80 | 109.59 | 14.21 | 11.48 |
| Isoxazole | 60.60 | 108.10 | 111.24 | -3.14 | -2.91 |
| Trifluoroacetonitrile | 60.80 | 118.00 | 111.61 | 6.39 | 5.41 |
| Bromotrifluoromethane | 61.30 | 163.60 | 112.53 | 51.07 | 31.22 |
| Dichlorofluoromethane | 61.50 | 112.60 | 112.90 | -0.30 | -0.26 |
| Acetylchloride | 61.70 | 117.20 | 113.26 | 3.94 | 3.36 |
| Oxetane | 61.90 | 99.60 | 113.63 | -14.03 | -14.09 |
| Ethan-1,2-diol | 62.70 | 149.80 | 115.10 | 34.70 | 23.17 |
| Propanenitrile | 62.90 | 119.30 | 115.46 | 3.84 | 3.22 |
| 2-Oxetanone | 63.20 | 122.10 | 116.01 | 6.09 | 4.98 |
| Bromoethane | 63.30 | 105.80 | 116.20 | -10.40 | -9.83 |
| Ethanethiol | 63.40 | 117.90 | 116.38 | 1.52 | 1.29 |
| Dimethyl sulfide | 63.70 | 118.10 | 116.93 | 1.17 | 0.99 |
| Methyloxiran | 63.80 | 120.40 | 117.11 | 3.29 | 2.73 |
| Propanal | 64.50 | 134.70 | 118.40 | 16.30 | 12.10 |
| Propanone | 64.60 | 124.70 | 118.58 | 6.12 | 4.91 |
| Dibromomethane | 64.90 | 105.40 | 119.13 | -13.73 | -13.03 |
| Furan | 65.00 | 114.56 | 119.32 | -4.76 | -4.15 |
| Allyl alcohol | 65.10 | 146.30 | 119.50 | 26.80 | 18.32 |
| Colamine | 65.60 | 137.70 | 120.42 | 17.28 | 12.55 |
| Methylphosphonyl difluoride | 65.70 | 145.14 | 120.60 | 24.54 | 16.91 |
| Dichlorodifluoromethane | 66.40 | 126.80 | 121.88 | 4.92 | 3.88 |
| Cyclopropylamine | 66.60 | 147.11 | 122.25 | 24.86 | 16.90 |
| s-trans-1,3-Butadiene | 67.20 | 123.65 | 123.35 | 0.30 | 0.24 |
| 1,2-Butadiene | 67.40 | 123.00 | 123.72 | -0.72 | -0.58 |
| Pyrrole | 67.40 | 127.74 | 123.72 | 4.02 | 3.15 |
| Acrylic acid | 67.40 | 144.20 | 123.72 | 20.48 | 14.20 |
| Thiazole | 67.50 | 121.00 | 123.90 | -2.90 | -2.40 |
| cis-1,2-Dichloroethylene | 67.90 | 113.80 | 124.64 | -10.84 | -9.52 |
| trans-1,2-Dichloroethylene | 68.10 | 114.24 | 125.00 | -10.76 | -9.42 |
| Vinylidenechloride | 68.10 | 111.29 | 125.00 | -13.71 | -12.32 |
| 1-Butyne | 68.30 | 132.60 | 125.37 | 7.23 | 5.45 |
| 2-Butyne | 68.40 | 124.30 | 125.55 | -1.25 | -1.01 |
| Nitroethane | 68.40 | 134.22 | 125.55 | 8.67 | 6.46 |
| 1,2-Diaminoethane | 68.50 | 170.00 | 125.74 | 44.26 | 26.04 |
| Chlorotrifluoroethylene | 68.60 | 122.60 | 125.92 | -3.32 | -2.71 |
| Isothiazole | 68.60 | 119.70 | 125.92 | -6.22 | -5.20 |
| 1-Chloro-1,1-difluoroethane | 68.90 | 130.50 | 126.47 | 4.03 | 3.09 |
| Cyclobutane | 69.10 | 106.30 | 126.84 | -20.54 | -19.32 |
| 1,1-Dimethylhydrazine | 69.10 | 164.05 | 126.84 | 37.21 | 22.68 |
| 1,2-Dimethylhydrazine | 69.40 | 171.04 | 127.39 | 43.65 | 25.52 |
| Ethyl iodide | 69.50 | 115.10 | 127.57 | -12.47 | -10.83 |
| DMSO | 69.90 | 145.40 | 128.30 | 17.10 | 11.76 |
| Allyl chloride | 70.10 | 125.10 | 128.67 | -3.57 | -2.86 |
| 1,3-Dioxolane | 70.20 | 122.16 | 128.86 | -6.70 | -5.48 |
| Cyclopentadiene | 70.30 | 115.30 | 129.04 | -13.74 | -11.92 |
| 1-Propanol | 70.30 | 146.88 | 129.04 | 17.84 | 12.15 |
| 2-Propanol | 70.30 | 154.43 | 129.04 | 25.39 | 16.44 |
| 2,3-Dihydrofuran | 70.40 | 122.10 | 129.22 | -7.12 | -5.83 |
| Chloroacetic acid | 70.40 | 179.90 | 129.22 | 50.68 | 28.17 |
| Thietane | 70.50 | 112.50 | 129.41 | -16.91 | -15.03 |
| Trichloromethane | 70.70 | 114.32 | 129.77 | -15.45 | -13.52 |
| cis-2-Butene | 71.60 | 127.00 | 131.42 | -4.42 | -3.48 |
| Thiophene | 72.10 | 123.80 | 132.34 | -8.54 | -6.90 |
| 1,3-Dioxolan-2-one | 72.20 | 133.90 | 132.52 | 1.38 | 1.03 |
| Isobutylene | 72.20 | 121.30 | 132.52 | -11.22 | -9.25 |
| trans-2-Butene | 72.30 | 124.30 | 132.71 | -8.41 | -6.76 |
| 1-Butene | 72.60 | 128.96 | 133.26 | -4.30 | -3.33 |
| Cyclopropanecarbonitrile | 72.70 | 115.50 | 133.44 | -17.94 | -15.53 |
| 1,1-Dichloroethane | 73.20 | 126.27 | 134.36 | -8.09 | -6.41 |
| Ethylenedichloride | 73.30 | 128.81 | 134.54 | -5.73 | -4.45 |
| Propanoic acid | 73.30 | 158.60 | 134.54 | 24.06 | 15.17 |
| 2-Methylacrylonitrile | 73.40 | 126.30 | 134.73 | -8.43 | -6.67 |
| Isopropylamine | 73.40 | 164.00 | 134.73 | 29.27 | 17.85 |
| Trimethylamine | 73.50 | 132.60 | 134.91 | -2.31 | -1.74 |
| Methyl acetate | 74.00 | 140.56 | 135.83 | 4.73 | 3.37 |
| 1-Propylamine | 74.00 | 161.90 | 135.83 | 26.07 | 16.11 |
| Ethyl formate | 74.10 | 144.30 | 136.01 | 8.29 | 5.75 |
| Perfluoroethane | 74.30 | 131.40 | 136.38 | -4.98 | -3.79 |
| 2,5-Furandione | 74.70 | 120.10 | 137.11 | -17.01 | -14.16 |
| Crotonaldehyde | 75.50 | 148.60 | 138.58 | 10.02 | 6.74 |
| Methylphosphonyl chlorofluoride | 75.60 | 153.17 | 138.76 | 14.41 | 9.41 |
| Fluorotrichloromethane | 75.60 | 121.55 | 138.76 | -17.21 | -14.16 |
| 1-Chloropropane | 75.80 | 132.20 | 139.13 | -6.93 | -5.24 |
| Ethylnitrate | 76.40 | 170.30 | 140.23 | 30.07 | 17.66 |
| N,N-Dimethylformamide | 76.50 | 148.36 | 140.41 | 7.95 | 5.36 |
| 1-Methylimidazole | 77.00 | 147.20 | 141.33 | 5.87 | 3.99 |
| Diiodomethane | 77.30 | 133.81 | 141.88 | -8.07 | -6.03 |
| Cyclopentene | 77.30 | 122.38 | 141.88 | -19.50 | -15.93 |
| Tetrahydrofuran | 77.40 | 123.90 | 142.06 | -18.16 | -14.66 |
| 1-Bromo-2-chloroethane | 77.50 | 130.10 | 142.25 | -12.15 | -9.34 |
| Epichlorohydrin | 78.00 | 131.60 | 143.16 | -11.56 | -8.79 |
| Cyclobutanol | 78.10 | 153.70 | 143.35 | 10.35 | 6.74 |
| Butane | 78.20 | 140.90 | 143.53 | -2.63 | -1.87 |
| Propanoyl chloride | 78.20 | 147.30 | 143.53 | 3.77 | 2.56 |
| 1-Methyl-1H-pyrazole | 78.20 | 173.28 | 143.53 | 29.75 | 17.17 |
| Pyridine | 78.80 | 133.00 | 144.63 | -11.63 | -8.75 |
| Methylenecyclobutane | 78.90 | 133.60 | 144.81 | -11.21 | -8.39 |
| gamma-Butyrolactone | 79.30 | 141.40 | 145.55 | -4.15 | -2.93 |
| Propan-1,3-diol | 79.40 | 175.78 | 145.73 | 30.05 | 17.09 |
| 1,2-Propanediol | 79.40 | 180.30 | 145.73 | 34.57 | 19.17 |
| Isobutyronitrile | 79.50 | 156.10 | 145.91 | 10.19 | 6.52 |
| Butyronitrile | 79.60 | 134.20 | 146.10 | -11.90 | -8.87 |
| 2-Bromopropane | 79.90 | 135.60 | 146.65 | -11.05 | -8.15 |
| 2-Propanethiol | 80.00 | 145.35 | 146.83 | -1.48 | -1.02 |
| 1-Bromopropane | 80.00 | 134.60 | 146.83 | -12.23 | -9.09 |
| 1-Propanethiol | 80.10 | 144.56 | 147.02 | -2.46 | -1.70 |
| Pyrrolidine | 80.10 | 156.57 | 147.02 | 9.55 | 6.10 |
| Methyl cellosolve | 80.10 | 176.40 | 147.02 | 29.38 | 16.66 |
| Ethylepoxide | 80.50 | 147.00 | 147.75 | -0.75 | -0.51 |
| Ethyl methyl sulfide | 80.50 | 144.60 | 147.75 | -3.15 | -2.18 |
| 2-Methylfuran | 80.70 | 143.72 | 148.12 | -4.40 | -3.06 |
| Dimethoxymethane | 80.70 | 163.60 | 148.12 | 15.48 | 9.46 |
| Succinonitrile | 80.80 | 145.60 | 148.30 | -2.70 | -1.85 |
| Butanal | 81.20 | 163.70 | 149.03 | 14.67 | 8.96 |
| 1-Methylpyrrole | 81.30 | 150.06 | 149.22 | 0.84 | 0.56 |
| 2-Butanone | 81.30 | 158.40 | 149.22 | 9.18 | 5.80 |
| Spiro[2.2]pentane | 81.30 | 134.52 | 149.22 | -14.70 | -10.93 |
| 2-Pyrrolidone | 81.30 | 169.50 | 149.22 | 20.28 | 11.97 |
| 1,2-Dibromoethane | 81.60 | 136.00 | 149.77 | -13.77 | -10.12 |
| DL-Lactic acid | 81.80 | 210.50 | 150.13 | 60.37 | 28.68 |
| Lactic acid | 81.80 | 210.50 | 150.13 | 60.37 | 28.68 |
| 2-Methoxy-1-propene | 81.90 | 162.30 | 150.32 | 11.98 | 7.38 |
| 2-Cyanopropionaldehyde | 82.00 | 169.50 | 150.50 | 19.00 | 11.21 |
| beta-Cyanopropionaldehyde | 82.10 | 195.80 | 150.68 | 45.12 | 23.04 |
| 1-Cyanobicyclobutane | 82.20 | 131.80 | 150.87 | -19.07 | -14.47 |
| Trichloroethylene | 82.20 | 128.90 | 150.87 | -21.97 | -17.04 |
| Hydroxymethyl acetate | 82.40 | 214.00 | 151.23 | 62.77 | 29.33 |
| Dimethyl disulfide | 82.60 | 146.11 | 151.60 | -5.49 | -3.76 |
| Isoprene | 82.80 | 151.08 | 151.97 | -0.89 | -0.59 |
| Furfural | 83.20 | 162.80 | 152.70 | 10.10 | 6.20 |
| Tribromomethane | 83.20 | 135.10 | 152.70 | -17.60 | -13.03 |
| cis-1,3-Pentadiene | 83.30 | 146.57 | 152.89 | -6.32 | -4.31 |
| Benzene | 83.40 | 136.80 | 153.07 | -16.27 | -11.89 |
| Dimethyl carbonate | 83.50 | 157.90 | 153.25 | 4.65 | 2.94 |
| Refrigerant 115 | 83.50 | 114.20 | 153.25 | -39.05 | -34.20 |
| 1,4-Pentadiene | 83.60 | 146.82 | 153.44 | -6.62 | -4.51 |
| 3-Methyl-1,2-butadiene | 83.70 | 152.42 | 153.62 | -1.20 | -0.79 |
| trans-1,3-Pentadiene | 83.70 | 149.33 | 153.62 | -4.29 | -2.87 |
| 2,3-Pentadiene | 83.80 | 152.34 | 153.80 | -1.46 | -0.96 |
| Methacrylic acid | 84.00 | 161.10 | 154.17 | 6.93 | 4.30 |
| 1,2-Pentadiene | 84.10 | 150.83 | 154.35 | -3.52 | -2.34 |
| Methyl acrylate | 84.30 | 158.80 | 154.72 | 4.08 | 2.57 |
| Dichloroacetic acid | 84.50 | 188.00 | 155.09 | 32.91 | 17.51 |
| Cyclopentane | 84.60 | 128.80 | 155.27 | -26.47 | -20.55 |
| Vinyl acetate | 84.70 | 169.50 | 155.45 | 14.05 | 8.29 |
| 2-Nitropropane | 84.70 | 170.30 | 155.45 | 14.85 | 8.72 |
| 1-Nitropropane | 84.80 | 160.40 | 155.64 | 4.76 | 2.97 |
| Tetrachloromethane | 84.80 | 131.80 | 155.64 | -23.84 | -18.09 |
| Propylenediamine | 85.10 | 205.64 | 156.19 | 49.45 | 24.05 |
| 1,3-Diaminopropane | 85.20 | 204.40 | 156.37 | 48.03 | 23.50 |
| 2-Methylthiazole | 85.30 | 150.70 | 156.55 | -5.85 | -3.88 |
| Trimethylhydrazine | 85.70 | 185.80 | 157.29 | 28.51 | 15.35 |
| 2-Iodopropane | 86.00 | 137.30 | 157.84 | -20.54 | -14.96 |
| 1,4-Dioxane | 86.20 | 150.65 | 158.21 | -7.56 | -5.02 |
| 1-Iodopropane | 86.20 | 136.20 | 158.21 | -22.01 | -16.16 |
| 1,3-Dioxane | 86.30 | 143.90 | 158.39 | -14.49 | -10.07 |
| Thiolane | 86.40 | 140.16 | 158.57 | -18.41 | -13.14 |
| Cyclopentanone | 86.50 | 154.50 | 158.76 | -4.26 | -2.75 |
| 2-Chlorothiophene | 86.50 | 139.43 | 158.76 | -19.33 | -13.86 |
| 2-Methyl-1-propanol | 86.50 | 181.05 | 158.76 | 22.29 | 12.31 |
| 2-Butanol | 86.50 | 196.67 | 158.76 | 37.91 | 19.28 |
| 2-Methyl-2-propanol | 86.80 | 218.60 | 159.31 | 59.29 | 27.12 |
| 1-Butanol | 87.00 | 177.16 | 159.67 | 17.49 | 9.87 |
| 1,1,2-Trichloroethane | 87.20 | 150.90 | 160.04 | -9.14 | -6.06 |
| Cyclobutanecarbonitrile | 87.20 | 146.00 | 160.04 | -14.04 | -9.62 |
| 1,1,1-Trichloroethane | 87.30 | 144.40 | 160.22 | -15.82 | -10.96 |
| Isopropyl methyl ether | 87.50 | 161.90 | 160.59 | 1.31 | 0.81 |
| Ethylurea | 87.50 | 151.50 | 160.59 | -9.09 | -6.00 |
| Ethyl ether | 87.80 | 172.50 | 161.14 | 11.36 | 6.59 |
| Methylpropylether | 87.90 | 165.40 | 161.32 | 4.08 | 2.46 |
| Trimethylethylene | 87.90 | 152.80 | 161.32 | -8.52 | -5.58 |
| 1,2,3-Propanetriol | 88.20 | 218.90 | 161.87 | 57.03 | 26.05 |
| Fluorobenzene | 88.30 | 146.29 | 162.06 | -15.77 | -10.78 |
| Propylenecarbonate | 88.50 | 167.40 | 162.42 | 4.98 | 2.97 |
| Isopentene | 88.50 | 157.30 | 162.42 | -5.12 | -3.26 |
| cis-2-Pentene | 88.70 | 151.80 | 162.79 | -10.99 | -7.24 |
| Morpholine | 88.90 | 164.80 | 163.16 | 1.64 | 1.00 |
| trans-2-Pentene | 88.90 | 157.00 | 163.16 | -6.16 | -3.92 |
| 1,3-Cyclohexadiene | 88.90 | 141.30 | 163.16 | -21.86 | -15.47 |
| Bromotrichloromethane | 89.00 | 148.50 | 163.34 | -14.84 | -9.99 |
| 3-Methyl-1-butene | 89.20 | 156.10 | 163.71 | -7.61 | -4.87 |
| 3-Methylthiophene | 89.20 | 150.62 | 163.71 | -13.09 | -8.69 |
| 2-Methylthiophene | 89.20 | 149.83 | 163.71 | -13.88 | -9.26 |
| 1-Pentene | 89.30 | 155.30 | 163.89 | -8.59 | -5.53 |
| 2,5-Norbornadiene | 89.50 | 161.20 | 164.26 | -3.06 | -1.90 |
| 1,1-Dichloropropane | 89.70 | 152.82 | 164.63 | -11.81 | -7.73 |
| Methyl propanoate | 89.80 | 172.74 | 164.81 | 7.93 | 4.59 |
| 1,2-Dichloropropane | 89.80 | 156.81 | 164.81 | -8.00 | -5.10 |
| 2,2-Dichloropropane | 89.80 | 155.65 | 164.81 | -9.16 | -5.88 |
| Chloral | 89.80 | 151.50 | 164.81 | -13.31 | -8.79 |
| 2-Methylpropanoic acid | 89.80 | 181.70 | 164.81 | 16.89 | 9.30 |
| Furfuranol | 89.80 | 204.01 | 164.81 | 39.20 | 19.22 |
| Butanoic acid | 89.90 | 177.70 | 164.99 | 12.71 | 7.15 |
| 2-Methylpropylamine | 89.90 | 183.20 | 164.99 | 18.21 | 9.94 |
| t-Butylamine | 89.90 | 191.71 | 164.99 | 26.72 | 13.94 |
| 1,3-Dichloropropane | 90.00 | 157.00 | 165.18 | -8.18 | -5.21 |
| 1,4-Cyclohexadiene | 90.00 | 142.20 | 165.18 | -22.98 | -16.16 |
| 3-Chloro-1,1,1-trifluoropropane | 90.40 | 171.08 | 165.91 | 5.17 | 3.02 |
| 2-Bromothiophene | 90.40 | 144.00 | 165.91 | -21.91 | -15.22 |
| Diethylamine | 90.50 | 169.20 | 166.09 | 3.11 | 1.84 |
| Ethyl acetate | 90.60 | 169.06 | 166.28 | 2.78 | 1.65 |
| 1-Butylamine | 90.60 | 179.20 | 166.28 | 12.92 | 7.21 |
| n-Propylformate | 90.90 | 171.50 | 166.83 | 4.67 | 2.72 |
| 1-Bromo-2-chloro-1,1,2-trifluoroethane | 92.00 | 160.30 | 168.84 | -8.54 | -5.33 |
| 2-Bromo-2-chloro-1,1,1-trifluoroethane | 92.00 | 156.60 | 168.84 | -12.24 | -7.82 |
| 2-Chloro-2-methylpropane | 92.20 | 162.00 | 169.21 | -7.21 | -4.45 |
| 2-Chlorobutane | 92.20 | 160.90 | 169.21 | -8.31 | -5.17 |
| Isobutyl chloride | 92.20 | 158.60 | 169.21 | -10.61 | -6.69 |
| N-Methylpropionamide | 92.30 | 179.00 | 169.40 | 9.60 | 5.37 |
| Ethylacetamide | 92.30 | 179.90 | 169.40 | 10.50 | 5.84 |
| Phenol | 92.30 | 200.00 | 169.40 | 30.60 | 15.30 |
| Isopropylnitrate | 92.40 | 191.10 | 169.58 | 21.52 | 11.26 |
| 1-Chlorobutane | 92.50 | 159.64 | 169.76 | -10.12 | -6.34 |
| Cryofluorane | 92.80 | 164.00 | 170.31 | -6.31 | -3.85 |
| Perfluoroacetone | 92.90 | 181.20 | 170.50 | 10.70 | 5.91 |
| 1,3-Difluorobenzene | 93.20 | 159.11 | 171.05 | -11.94 | -7.50 |
| 1,4-Difluorobenzene | 93.20 | 159.10 | 171.05 | -11.95 | -7.51 |
| 1,2-Difluorobenzene | 93.20 | 159.03 | 171.05 | -12.02 | -7.56 |
| Cyclopentanol | 93.40 | 185.40 | 171.41 | 13.99 | 7.54 |
| Dimethylacetamide | 93.50 | 175.60 | 171.60 | 4.00 | 2.28 |
| Tetrahydropyran | 93.50 | 149.60 | 171.60 | -22.00 | -14.70 |
| 3-Methylcyclopentene | 93.90 | 152.30 | 172.33 | -20.03 | -13.15 |
| 2-Methyltetrahydrofuran | 94.10 | 156.89 | 172.70 | -15.81 | -10.08 |
| 1-Methylcyclopentene | 94.20 | 153.10 | 172.88 | -19.78 | -12.92 |
| Isopentane | 94.50 | 164.85 | 173.43 | -8.58 | -5.21 |
| Isobutyroylchloride | 94.50 | 131.80 | 173.43 | -41.63 | -31.59 |
| Butanoyl chloride | 94.70 | 170.70 | 173.80 | -3.10 | -1.81 |
| Aniline | 94.70 | 178.70 | 173.80 | 4.90 | 2.74 |
| 2,2-Dimethylpropane | 94.70 | 153.10 | 173.80 | -20.70 | -13.52 |
| Pentane | 94.90 | 167.19 | 174.16 | -6.97 | -4.17 |
| 1-Ethylpyrazole | 94.90 | 189.50 | 174.16 | 15.34 | 8.09 |
| Cyclohexene | 95.00 | 152.90 | 174.35 | -21.45 | -14.03 |
| 3-Methylenecyclobutanenitrile | 95.20 | 190.80 | 174.72 | 16.08 | 8.43 |
| 2-Picoline | 95.20 | 158.41 | 174.72 | -16.31 | -10.29 |
| 4-Methylpyridine | 95.30 | 158.99 | 174.90 | -15.91 | -10.01 |
| 3-Methylpyridine | 95.30 | 158.69 | 174.90 | -16.21 | -10.21 |
| Quadricyclane | 95.40 | 157.60 | 175.08 | -17.48 | -11.09 |
| Pivalonitrile | 95.90 | 179.37 | 176.00 | 3.37 | 1.88 |
| 1-Ethylimidazole | 96.00 | 186.82 | 176.18 | 10.64 | 5.69 |
| 2,3-Butyleneglycol | 96.00 | 213.00 | 176.18 | 36.82 | 17.29 |
| 1,2-Butanediol | 96.00 | 230.82 | 176.18 | 54.64 | 23.67 |
| Piperidine | 96.10 | 179.86 | 176.37 | 3.49 | 1.94 |
| Tetrahydro-2H-pyran-2-one | 96.10 | 170.70 | 176.37 | -5.67 | -3.32 |
| Methyltrichlorosilane | 96.10 | 163.10 | 176.37 | -13.27 | -8.13 |
| Tetramethylene glycol | 96.10 | 203.79 | 176.37 | 27.42 | 13.46 |
| 1,3-Butyleneglycol | 96.10 | 218.89 | 176.37 | 42.52 | 19.43 |
| Cyclopentylamine | 96.20 | 181.21 | 176.55 | 4.66 | 2.57 |
| Tetrachloroethylene | 96.20 | 157.70 | 176.55 | -18.85 | -11.95 |
| Isobutyl bromide | 96.30 | 163.70 | 176.73 | -13.03 | -7.96 |
| t-Butylmercaptan | 96.40 | 174.90 | 176.92 | -2.02 | -1.15 |
| 2-Methyl-1-propanethiol | 96.40 | 171.88 | 176.92 | -5.04 | -2.93 |
| 2-Bromo-2-methylpropane | 96.40 | 165.70 | 176.92 | -11.22 | -6.77 |
| 3-Methyl-2-butanone | 96.50 | 180.00 | 177.10 | 2.90 | 1.61 |
| 2-Butanethiol | 96.50 | 171.20 | 177.10 | -5.90 | -3.45 |
| Isopropyl methyl sulfide | 96.60 | 172.38 | 177.28 | -4.90 | -2.84 |
| 3-Methylpyrrolidine | 96.60 | 188.28 | 177.28 | 11.00 | 5.84 |
| 1-Cyanobicyclo[2.1.0]pentane | 96.70 | 164.00 | 177.47 | -13.47 | -8.21 |
| 1-Bromobutane | 96.70 | 162.20 | 177.47 | -15.27 | -9.41 |
| 1-Butanethiol | 96.80 | 172.30 | 177.65 | -5.35 | -3.11 |
| Diethylketone | 96.80 | 190.90 | 177.65 | 13.25 | 6.94 |
| N-Methylpyrrolidine | 96.90 | 167.75 | 177.83 | -10.09 | -6.01 |
| 2-Ethoxyethanol | 96.90 | 210.30 | 177.83 | 32.47 | 15.44 |
| Methyl-n-propylketone | 97.00 | 184.10 | 178.02 | 6.08 | 3.30 |
| 1,1-Difluoro-1,2,2-trichloroethane | 97.00 | 167.04 | 178.02 | -10.98 | -6.57 |
| 2,4-Dimethylpyrrole | 97.00 | 192.19 | 178.02 | 14.17 | 7.37 |
| Dimethylmalononitrile | 97.10 | 179.50 | 178.20 | 1.30 | 0.72 |
| Methyl propyl sulfide | 97.20 | 171.63 | 178.38 | -6.75 | -3.94 |
| Diethyl sulphide | 97.20 | 171.42 | 178.38 | -6.96 | -4.06 |
| Pivalaldehyde | 97.20 | 185.58 | 178.38 | 7.20 | 3.88 |
| Glyme | 97.40 | 191.14 | 178.75 | 12.39 | 6.48 |
| Pentanedinitrile | 97.50 | 183.30 | 178.93 | 4.37 | 2.38 |
| n-Valeraldehyde | 97.60 | 174.39 | 179.12 | -4.73 | -2.71 |
| Chlorobenzene | 97.60 | 153.78 | 179.12 | -25.34 | -16.48 |
| Dimethylvinylcarbinol | 97.80 | 208.40 | 179.48 | 28.92 | 13.88 |
| 1,2-Dibromopropane | 98.10 | 172.80 | 180.03 | -7.23 | -4.19 |
| 1,3-Dibromopropane | 98.30 | 163.70 | 180.40 | -16.70 | -10.20 |
| 2-Piperidone | 98.30 | 208.40 | 180.40 | 28.00 | 13.43 |
| Acetylacetone (enol form) | 98.60 | 208.40 | 180.95 | 27.45 | 13.17 |
| Cellosolve acetate | 98.70 | 203.00 | 181.14 | 21.86 | 10.77 |
| Chlorocyclopentane | 98.70 | 152.40 | 181.14 | -28.74 | -18.86 |
| 2-Aminoisobutanol | 98.70 | 229.50 | 181.14 | 48.36 | 21.07 |
| 2,5-Dimethylpyrrole | 98.80 | 195.30 | 181.32 | 13.98 | 7.16 |
| N-Methyl-2-pyrrolidone | 98.90 | 166.26 | 181.50 | -15.24 | -9.17 |
| Dichlorodimethylsilane | 99.20 | 171.50 | 182.05 | -10.55 | -6.15 |
| 2-(Ethylamino)ethanol | 99.40 | 227.00 | 182.42 | 44.58 | 19.64 |
| 4-Fluoroaniline | 99.70 | 195.20 | 182.97 | 12.23 | 6.27 |
| 3-Chloro-1,1,1,3,3-pentafluoropropane | 99.70 | 196.60 | 182.97 | 13.63 | 6.93 |
| 1-Amino-3-methoxypropane | 99.70 | 225.52 | 182.97 | 42.55 | 18.87 |
| Methylbenzene | 100.00 | 158.70 | 183.52 | -24.82 | -15.64 |
| Perfluoropropane | 100.10 | 181.60 | 183.70 | -2.10 | -1.16 |
| Sulfolane | 100.20 | 180.00 | 183.89 | -3.89 | -2.16 |
| 1,5-Hexadiene | 100.40 | 133.10 | 184.25 | -51.15 | -38.43 |
| Cyclohexane | 100.50 | 158.10 | 184.44 | -26.34 | -16.66 |
| Benzonitrile | 100.90 | 165.20 | 185.17 | -19.97 | -12.09 |
| Butanedioic acid | 100.90 | 238.60 | 185.17 | 53.43 | 22.39 |
| 1,2-Dibromotetrafluoroethane | 101.00 | 173.80 | 185.35 | -11.55 | -6.65 |
| Methylcyclopentane | 101.00 | 158.70 | 185.35 | -26.65 | -16.80 |
| Allyl acetate | 101.20 | 184.10 | 185.72 | -1.62 | -0.88 |
| Methyl methacrylate | 101.20 | 188.70 | 185.72 | 2.98 | 1.58 |
| 2-Methyl-1,2-propanediamine | 101.20 | 234.56 | 185.72 | 48.84 | 20.82 |
| 1,1,2,2-Tetrachloroethane | 101.30 | 168.00 | 185.90 | -17.90 | -10.66 |
| Carbon tetrabromide | 101.40 | 162.30 | 186.09 | -23.79 | -14.66 |
| Bromobenzene | 101.80 | 154.29 | 186.82 | -32.53 | -21.09 |
| Benzenethiol | 101.90 | 173.22 | 187.01 | -13.79 | -7.96 |
| Freon 113 | 101.90 | 172.80 | 187.01 | -14.21 | -8.22 |
| 1,1,1-Trichlorotrifluoroethane | 101.90 | 168.60 | 187.01 | -18.41 | -10.92 |
| Chlorotrimethylsilane | 102.20 | 187.50 | 187.56 | -0.06 | -0.03 |
| Tetrahydrofurfuryl alcohol | 102.40 | 190.00 | 187.92 | 2.08 | 1.09 |
| Cyclopentanecarbonitrile | 102.40 | 167.40 | 187.92 | -20.52 | -12.26 |
| Isobutyl iodide | 102.40 | 165.50 | 187.92 | -22.42 | -13.55 |
| 2-Iodobutane | 102.40 | 165.30 | 187.92 | -22.62 | -13.69 |
| Benzaldehyde | 102.60 | 177.90 | 188.29 | -10.39 | -5.84 |
| 1,3-Dioxepane | 102.60 | 167.00 | 188.29 | -21.29 | -12.75 |
| Thiane | 102.60 | 163.30 | 188.29 | -24.99 | -15.30 |
| 7-Oxabicyclo[4.1.0]heptane | 102.70 | 166.10 | 188.47 | -22.37 | -13.47 |
| Cyclopentanethiol | 102.70 | 165.20 | 188.47 | -23.27 | -14.09 |
| Cyclohexanone | 102.80 | 182.20 | 188.66 | -6.46 | -3.54 |
| 1-Iodobutane | 102.90 | 164.50 | 188.84 | -24.34 | -14.80 |
| 2-Methyl-2-butanol | 102.90 | 247.30 | 188.84 | 58.46 | 23.64 |
| 1,2,3,4-Tetrafluorobenzene | 103.00 | 189.90 | 189.02 | 0.88 | 0.46 |
| 1,2,3,5-Tetrafluorobenzene | 103.00 | 190.30 | 189.02 | 1.28 | 0.67 |
| 1,2,4,5-Tetrafluorobenzene | 103.00 | 192.20 | 189.02 | 3.18 | 1.65 |
| Isopentyl alcohol | 103.00 | 209.60 | 189.02 | 20.58 | 9.82 |
| 3-Methylthiolane | 103.10 | 172.85 | 189.21 | -16.36 | -9.46 |
| 2-Methylthiolane | 103.10 | 172.00 | 189.21 | -17.21 | -10.00 |
| 3-Methyl-2-butanol | 103.10 | 245.90 | 189.21 | 56.69 | 23.06 |
| Tetramethylethene | 103.50 | 174.68 | 189.94 | -15.26 | -8.74 |
| Neopentyl alcohol | 103.50 | 244.30 | 189.94 | 54.36 | 22.25 |
| 1-Pentanol | 103.60 | 208.14 | 190.12 | 18.02 | 8.66 |
| MTBE | 103.80 | 187.50 | 190.49 | -2.99 | -1.60 |
| 3-Pentanol | 103.80 | 239.70 | 190.49 | 49.21 | 20.53 |
| 1,2,3-Trichloropropane | 103.90 | 183.60 | 190.67 | -7.07 | -3.85 |
| 2-Fluorotoluene | 104.40 | 171.20 | 191.59 | -20.39 | -11.91 |
| n-Butyl methyl ether | 104.60 | 192.48 | 191.96 | 0.52 | 0.27 |
| Ethyl-n-propylether | 104.60 | 197.20 | 191.96 | 5.24 | 2.66 |
| 4-Fluorotoluene | 104.60 | 173.70 | 191.96 | -18.26 | -10.51 |
| cis-2-Hexene | 105.20 | 178.36 | 193.06 | -14.70 | -8.24 |
| Tetramethylsilane | 105.30 | 204.10 | 193.24 | 10.86 | 5.32 |
| Isopropylacetate | 105.50 | 196.60 | 193.61 | 2.99 | 1.52 |
| 3,3-Dimethyl-1-butene | 105.50 | 188.30 | 193.61 | -5.31 | -2.82 |
| 4-Methylpent-3-en-2-one | 105.50 | 212.50 | 193.61 | 18.89 | 8.89 |
| Methyl methylthiomethyl sulfoxide | 105.50 | 219.87 | 193.61 | 26.26 | 11.94 |
| N-Methylmorpholine | 105.70 | 199.26 | 193.98 | 5.28 | 2.65 |
| Diethyleneglycol | 105.80 | 243.90 | 194.16 | 49.74 | 20.39 |
| Diethylmethylamine | 105.90 | 200.00 | 194.34 | 5.66 | 2.83 |
| 1-Hexene | 106.00 | 183.30 | 194.53 | -11.23 | -6.12 |
| 3-Methylbutanoic acid | 106.20 | 197.10 | 194.89 | 2.21 | 1.12 |
| 1,2-Dichlorobutane | 106.30 | 195.60 | 195.08 | 0.52 | 0.27 |
| Trimethoxyborine | 106.30 | 189.90 | 195.08 | -5.18 | -2.73 |
| Ethynylbenzene | 106.40 | 180.10 | 195.26 | -15.16 | -8.42 |
| 2,5-Dimethylthiophene | 106.40 | 178.32 | 195.26 | -16.94 | -9.50 |
| Pentanoic acid | 106.60 | 210.00 | 195.63 | 14.37 | 6.84 |
| 1,4-Dichlorobutane | 106.70 | 184.00 | 195.81 | -11.81 | -6.42 |
| Phenylhydrazine | 106.70 | 217.00 | 195.81 | 21.19 | 9.76 |
| Amylamine | 106.80 | 218.00 | 195.99 | 22.01 | 10.09 |
| n-Propyl acetate | 106.90 | 194.10 | 196.18 | -2.08 | -1.07 |
| Nitrobenzene | 106.90 | 186.60 | 196.18 | -9.58 | -5.13 |
| Butyric acid methylester | 107.00 | 198.16 | 196.36 | 1.80 | 0.91 |
| Ethyl propanoate | 107.00 | 204.06 | 196.36 | 7.70 | 3.77 |
| 1,3,5-Cycloheptatriene | 107.00 | 162.76 | 196.36 | -33.60 | -20.64 |
| Isobutyl formate | 107.40 | 214.20 | 197.09 | 17.11 | 7.99 |
| n-Butylformate | 107.60 | 200.25 | 197.46 | 2.79 | 1.39 |
| 2,2-Dinitropropane | 107.90 | 206.30 | 198.01 | 8.29 | 4.02 |
| Pentafluorobenzene | 107.90 | 210.79 | 198.01 | 12.78 | 6.06 |
| 3-Dimethylaminopropanenitrile | 107.90 | 212.50 | 198.01 | 14.49 | 6.82 |
| Perfluorocyclobutane | 108.00 | 209.60 | 198.20 | 11.40 | 5.44 |
| Iodobenzene | 108.00 | 158.40 | 198.20 | -39.80 | -25.12 |
| Ethyl cyanoacetate | 108.30 | 220.22 | 198.75 | 21.47 | 9.75 |
| o-Cresol | 108.50 | 229.75 | 199.11 | 30.64 | 13.34 |
| Benzyl alcohol | 108.60 | 215.90 | 199.30 | 16.60 | 7.69 |
| N-Methylbutylamide | 108.70 | 207.00 | 199.48 | 7.52 | 3.63 |
| N,2-Dimethylpropanamide | 108.70 | 209.20 | 199.48 | 9.72 | 4.65 |
| Isopentyl chloride | 108.70 | 179.50 | 199.48 | -19.98 | -11.13 |
| p-Cresol | 108.80 | 221.03 | 199.66 | 21.37 | 9.67 |
| Propylacetamide | 108.90 | 207.94 | 199.85 | 8.09 | 3.89 |
| Phenylisocyanate | 108.90 | 186.20 | 199.85 | -13.65 | -7.33 |
| m-Cresol | 109.00 | 224.93 | 200.03 | 24.90 | 11.07 |
| Anisole | 109.10 | 199.00 | 200.21 | -1.21 | -0.61 |
| Methyl bicyclobutane-1-carboxylate | 109.20 | 192.90 | 200.40 | -7.50 | -3.89 |
| N,N-Dimethylpropionamide | 109.20 | 209.00 | 200.40 | 8.60 | 4.12 |
| 1-Chloropentane | 109.20 | 187.70 | 200.40 | -12.70 | -6.76 |
| N-(1-Methylethyl)ethanamide | 109.30 | 211.00 | 200.58 | 10.42 | 4.94 |
| Benzo[b]furan | 109.30 | 178.70 | 200.58 | -21.88 | -12.24 |
| Cyclohexanol | 109.50 | 213.40 | 200.95 | 12.45 | 5.84 |
| 2-Chloroaniline | 109.70 | 199.00 | 201.31 | -2.31 | -1.16 |
| Cyclooctatetraene | 109.70 | 185.18 | 201.31 | -16.13 | -8.71 |
| 3-Chloroaniline | 109.80 | 198.70 | 201.50 | -2.80 | -1.41 |
| 2-Hydroxybenzaldehyde | 109.90 | 222.00 | 201.68 | 20.32 | 9.15 |
| Norcarane | 110.10 | 187.90 | 202.05 | -14.15 | -7.53 |
| Styrene | 110.40 | 201.90 | 202.60 | -0.70 | -0.35 |
| Cycloheptene | 110.40 | 171.70 | 202.60 | -30.90 | -18.00 |
| Methylvinyldichlorosilane | 110.60 | 177.90 | 202.96 | -25.06 | -14.09 |
| 1,3-Diiodopropane | 110.70 | 169.20 | 203.15 | -33.95 | -20.06 |
| 2,5-Dimethyltetrahydrofuran | 110.80 | 188.13 | 203.33 | -15.20 | -8.08 |
| Neohexane | 111.00 | 189.67 | 203.70 | -14.03 | -7.40 |
| 2,3-Lutidine | 111.00 | 189.55 | 203.70 | -14.15 | -7.46 |
| 2,3-Dimethylbutane | 111.00 | 188.80 | 203.70 | -14.90 | -7.89 |
| 4-Methylcyclohexene | 111.00 | 180.42 | 203.70 | -23.28 | -12.90 |
| 1,1,2,2-Tetrachloro-1,2-difluoroethane | 111.00 | 178.70 | 203.70 | -25.00 | -13.99 |
| 3,4-Lutidine | 111.10 | 196.00 | 203.88 | -7.88 | -4.02 |
| 1-Ethylcyclopentene | 111.20 | 188.30 | 204.07 | -15.77 | -8.37 |
| Methylenecyclohexane | 111.20 | 177.40 | 204.07 | -26.67 | -15.03 |
| Ethylidenecyclopentane | 111.30 | 181.20 | 204.25 | -23.05 | -12.72 |
| 3-Methylpentane | 111.40 | 190.83 | 204.43 | -13.60 | -7.13 |
| Valeryl chloride | 111.40 | 187.90 | 204.43 | -16.53 | -8.80 |
| 2,6-Lutidine | 111.40 | 185.17 | 204.43 | -19.26 | -10.40 |
| Hexane | 111.50 | 197.66 | 204.62 | -6.96 | -3.52 |
| 2-Methylpentane | 111.50 | 193.96 | 204.62 | -10.66 | -5.49 |
| 2,4-Lutidine | 111.50 | 184.76 | 204.62 | -19.86 | -10.75 |
| 2,5-Lutidine | 111.50 | 184.70 | 204.62 | -19.92 | -10.78 |
| 3,5-Lutidine | 111.60 | 184.55 | 204.80 | -20.25 | -10.97 |
| 1,2-Dichlorobenzene | 111.70 | 170.90 | 204.98 | -34.08 | -19.94 |
| N-Methylaniline | 111.80 | 207.10 | 205.17 | 1.93 | 0.93 |
| Benzylamine | 111.90 | 207.18 | 205.35 | 1.83 | 0.88 |
| Caprolactone | 111.90 | 196.80 | 205.35 | -8.55 | -4.34 |
| 1,3-Dichlorobenzene | 111.90 | 170.90 | 205.35 | -34.45 | -20.16 |
| Hexamethylenimine | 112.00 | 205.00 | 205.53 | -0.53 | -0.26 |
| m-Toluidine | 112.20 | 216.70 | 205.90 | 10.80 | 4.98 |
| 1-Cyanobicyclo[3.1.0]hexane | 112.20 | 170.30 | 205.90 | -35.60 | -20.90 |
| o-Toluidine | 112.30 | 209.60 | 206.08 | 3.52 | 1.68 |
| Pinacolone | 112.40 | 206.90 | 206.27 | 0.63 | 0.31 |
| t-Amyl mercaptan | 112.40 | 198.15 | 206.27 | -8.12 | -4.10 |
| 1,1-Dimethylsilacyclobutane | 112.40 | 197.53 | 206.27 | -8.74 | -4.42 |
| 2-Methyl-1-butanethiol | 112.60 | 200.70 | 206.63 | -5.93 | -2.96 |
| 3-Methyl-2-butanethiol | 112.60 | 198.95 | 206.63 | -7.68 | -3.86 |
| 2-Methylpiperidine | 112.70 | 205.00 | 206.82 | -1.82 | -0.89 |
| 4-Methylpiperidine | 112.70 | 209.00 | 206.82 | 2.18 | 1.04 |
| Methyl t-butyl sulfide | 112.70 | 199.95 | 206.82 | -6.87 | -3.43 |
| Ethyltrichlorosilane | 112.70 | 192.60 | 206.82 | -14.22 | -7.38 |
| Benzothiazole | 112.80 | 189.54 | 207.00 | -17.46 | -9.21 |
| N-Methylpiperidine | 112.80 | 184.90 | 207.00 | -22.10 | -11.95 |
| 1,5-Pentanediol | 112.80 | 232.49 | 207.00 | 25.49 | 10.96 |
| Hexafluorobenzene | 112.90 | 221.60 | 207.18 | 14.42 | 6.51 |
| Isopentyl bromide | 112.90 | 187.00 | 207.18 | -20.18 | -10.79 |
| 3-Methyl-1-butanethiol | 113.00 | 200.33 | 207.37 | -7.04 | -3.51 |
| 2,2-Dimethoxypropane | 113.00 | 217.70 | 207.37 | 10.33 | 4.75 |
| Indole | 113.00 | 192.00 | 207.37 | -15.37 | -8.00 |
| Ethylisopropylketone | 113.00 | 187.90 | 207.37 | -19.47 | -10.36 |
| (Chloromethyl)benzene | 113.00 | 182.40 | 207.37 | -24.97 | -13.69 |
| 2-Isopropoxyethanol | 113.20 | 238.80 | 207.73 | 31.07 | 13.01 |
| Butylmethylketone | 113.30 | 213.40 | 207.92 | 5.48 | 2.57 |
| 1-Bromopentane | 113.40 | 219.70 | 208.10 | 11.60 | 5.28 |
| 1-Pentanethiol | 113.50 | 200.40 | 208.28 | -7.88 | -3.93 |
| Methyl isobutyl ketone | 113.60 | 211.90 | 208.47 | 3.43 | 1.62 |
| 2-Propoxyethanol | 113.60 | 241.60 | 208.47 | 33.13 | 13.71 |
| 2-Chlorotoluene | 113.70 | 166.80 | 208.65 | -41.85 | -25.09 |
| 3-Hexanone | 113.90 | 216.90 | 209.02 | 7.88 | 3.63 |
| n-Butylmethylsulfide | 113.90 | 200.92 | 209.02 | -8.10 | -4.03 |
| Ethyl propyl sulfide | 113.90 | 198.41 | 209.02 | -10.61 | -5.35 |
| Benzotrifluoride | 114.10 | 188.45 | 209.39 | -20.94 | -11.11 |
| 2,2’-Diaminodiethylamine | 114.10 | 254.00 | 209.39 | 44.61 | 17.56 |
| 2,5-Dioxaheptane | 114.20 | 224.70 | 209.57 | 15.13 | 6.73 |
| Caproic aldehyde | 114.30 | 210.40 | 209.75 | 0.65 | 0.31 |
| cis-3-Hexen-1-ol | 114.30 | 234.00 | 209.75 | 24.25 | 10.36 |
| Methyl cyclobutanecarboxylate | 114.40 | 190.80 | 209.94 | -19.14 | -10.03 |
| 1,3-Dihydroisobenzofuran | 114.60 | 188.70 | 210.30 | -21.60 | -11.45 |
| trans-3-Hexen-1-ol | 114.60 | 237.20 | 210.30 | 26.90 | 11.34 |
| 1-Hexen-3-ol | 114.60 | 269.30 | 210.30 | 59.00 | 21.91 |
| 2,3-Dihydrobenzofuran | 114.80 | 188.64 | 210.67 | -22.03 | -11.68 |
| 1,4-Dibromobutane | 115.00 | 191.20 | 211.04 | -19.84 | -10.37 |
| t-Butyl vinyl ether | 115.30 | 217.60 | 211.59 | 6.01 | 2.76 |
| Pentachloroethane | 115.30 | 196.20 | 211.59 | -15.39 | -7.84 |
| Cycloheptane | 115.40 | 180.61 | 211.77 | -31.16 | -17.25 |
| 2-(Isopropylamino)ethanol | 115.50 | 258.82 | 211.95 | 46.87 | 18.11 |
| Isobutyl vinyl ether | 115.80 | 231.80 | 212.50 | 19.30 | 8.32 |
| Bromo-2-chlorobenzene | 115.80 | 176.90 | 212.50 | -35.60 | -20.13 |
| Ethyl lactate | 115.80 | 254.00 | 212.50 | 41.50 | 16.34 |
| Diethyl disulfide | 116.00 | 204.01 | 212.87 | -8.86 | -4.34 |
| 3-Bromochlorobenzene | 116.00 | 181.90 | 212.87 | -30.97 | -17.03 |
| Butyl vinyl ether | 116.10 | 231.80 | 213.05 | 18.75 | 8.09 |
| Ethylbenzene | 116.10 | 185.78 | 213.05 | -27.27 | -14.68 |
| Bicyclo[2.2.2]oct-2-ene | 116.10 | 156.73 | 213.05 | -56.32 | -35.94 |
| 1,2-Dimethylbenzene | 116.20 | 187.65 | 213.24 | -25.59 | -13.64 |
| 1,2,3-Tribromopropane | 116.20 | 166.50 | 213.24 | -46.74 | -28.07 |
| Bis(2-chloroethyl) ether | 116.30 | 220.90 | 213.42 | 7.48 | 3.39 |
| Ethyl cyclopropanecarboxylate | 116.60 | 213.00 | 213.97 | -0.97 | -0.46 |
| Benzoyl chloride | 116.60 | 187.00 | 213.97 | -26.97 | -14.42 |
| Trimethylvinylsilane | 116.80 | 198.00 | 214.34 | -16.34 | -8.25 |
| Indene | 116.80 | 186.94 | 214.34 | -27.40 | -14.66 |
| 1,4-Dimethylbenzene | 116.80 | 183.65 | 214.34 | -30.69 | -16.71 |
| 1,3-Dimethylbenzene | 116.80 | 183.18 | 214.34 | -31.16 | -17.01 |
| Methylcyclohexane | 117.00 | 184.96 | 214.70 | -29.74 | -16.08 |
| Diethylcarbonate | 117.10 | 218.55 | 214.89 | 3.66 | 1.68 |
| cis-1,2-Dimethylcyclopentane | 117.20 | 190.80 | 215.07 | -24.27 | -12.72 |
| 1,1,1-Trichloro-3,3,3-trifluoropropane | 117.30 | 199.91 | 215.26 | -15.35 | -7.68 |
| Ethylcyclopentane | 117.40 | 187.40 | 215.44 | -28.04 | -14.96 |
| trans-1,3-Dimethylcyclopentane | 117.60 | 190.80 | 215.81 | -25.01 | -13.11 |
| Pentanedioic acid | 117.60 | 270.50 | 215.81 | 54.69 | 20.22 |
| 1,1,1,3-Tetrachloropropane | 117.70 | 196.40 | 215.99 | -19.59 | -9.97 |
| 1,1-Dimethylcyclopentane | 117.70 | 187.40 | 215.99 | -28.59 | -15.26 |
| 1,1,2,2-Tetrabromoethane | 117.80 | 165.70 | 216.17 | -50.47 | -30.46 |
| 1,5-Cyclooctadiene | 117.90 | 208.10 | 216.36 | -8.26 | -3.97 |
| Suberone | 118.10 | 211.80 | 216.72 | -4.92 | -2.32 |
| Thioanisole | 118.10 | 206.02 | 216.72 | -10.70 | -5.20 |
| Benzyl mercaptan | 118.10 | 202.81 | 216.72 | -13.91 | -6.86 |
| 2-Methoxyphenol | 118.10 | 240.00 | 216.72 | 23.28 | 9.70 |
| Acetophenone | 118.30 | 205.50 | 217.09 | -11.59 | -5.64 |
| 3-(Dimethylamino)propylamine | 118.40 | 248.71 | 217.27 | 31.44 | 12.64 |
| Ethyl dichloroacetate | 118.50 | 209.60 | 217.46 | -7.86 | -3.75 |
| Cyclohexanecarbonitrile | 118.50 | 177.80 | 217.46 | -39.66 | -22.30 |
| Dimethyl malonate | 118.90 | 210.10 | 218.19 | -8.09 | -3.85 |
| Bromocyclohexane | 118.90 | 182.10 | 218.19 | -36.09 | -19.82 |
| Tetrahydro-2H-pyran-2-methanol | 119.00 | 222.00 | 218.37 | 3.63 | 1.63 |
| Cyclopentyl methyl sulfide | 119.00 | 193.18 | 218.37 | -25.19 | -13.04 |
| Cyclohexanethiol | 119.00 | 192.63 | 218.37 | -25.74 | -13.36 |
| Isopentyliodide | 119.00 | 178.70 | 218.37 | -39.67 | -22.20 |
| Diisopropyl ether | 119.20 | 216.74 | 218.74 | -2.00 | -0.92 |
| Pentafluoroaniline | 119.20 | 231.00 | 218.74 | 12.26 | 5.31 |
| 2-Ethyl-1-butanol | 119.30 | 246.65 | 218.92 | 27.73 | 11.24 |
| 3-Methyl-3-pentanol | 119.40 | 293.30 | 219.11 | 74.19 | 25.30 |
| 3,3-Dimethyl-1-butanol | 119.60 | 236.08 | 219.47 | 16.61 | 7.03 |
| 1-Iodopentane | 119.60 | 193.60 | 219.47 | -25.87 | -13.36 |
| 4-Methyl-2-pentanol | 119.70 | 272.34 | 219.66 | 52.68 | 19.34 |
| 3-Methyl-2-pentanol | 119.70 | 275.89 | 219.66 | 56.23 | 20.38 |
| 2-Chloronitrobenzene | 119.80 | 212.30 | 219.84 | -7.54 | -3.55 |
| 3-Hexanol | 119.90 | 269.27 | 220.02 | 49.25 | 18.29 |
| Methyl t-pentyl ether | 120.00 | 222.00 | 220.21 | 1.79 | 0.81 |
| 1,2-Dibromobenzene | 120.00 | 196.80 | 220.21 | -23.41 | -11.89 |
| 2-Methyl-2-pentanol | 120.00 | 289.03 | 220.21 | 68.82 | 23.81 |
| 2-Methyl-1-pentanol | 120.10 | 248.40 | 220.39 | 28.01 | 11.28 |
| 1,3-Dibromobenzene | 120.20 | 192.00 | 220.58 | -28.58 | -14.88 |
| 4-Hydroxy-4-methyl-2-pentanone | 120.50 | 221.30 | 221.13 | 0.17 | 0.08 |
| Ethyl t-butyl ether | 120.50 | 218.00 | 221.13 | -3.13 | -1.43 |
| Tetramethylurea | 120.50 | 229.60 | 221.13 | 8.47 | 3.69 |
| 4-Methoxyaniline | 120.50 | 236.00 | 221.13 | 14.87 | 6.30 |
| 1-Hexanol | 120.60 | 242.70 | 221.31 | 21.39 | 8.81 |
| 2-Hexanol | 120.60 | 256.31 | 221.31 | 35.00 | 13.66 |
| 2-Isopropylthiophene | 120.90 | 211.38 | 221.86 | -10.48 | -4.96 |
| 4-Chloronitrobenzene | 120.90 | 250.20 | 221.86 | 28.34 | 11.33 |
| Ethyl 2-chloropropionate | 121.00 | 220.50 | 222.04 | -1.54 | -0.70 |
| Dipropyl ether | 121.10 | 221.45 | 222.23 | -0.78 | -0.35 |
| 2-Nitrotoluene | 121.20 | 202.10 | 222.41 | -20.31 | -10.05 |
| Pyrrolidinium nitrate | 121.40 | 228.00 | 222.78 | 5.22 | 2.29 |
| Indane | 121.60 | 190.25 | 223.14 | -32.89 | -17.29 |
| Triethylamine | 121.70 | 219.90 | 223.33 | -3.43 | -1.56 |
| N-Ethylmorpholine | 121.90 | 207.32 | 223.69 | -16.37 | -7.90 |
| t-Butylacetate | 122.00 | 231.00 | 223.88 | 7.12 | 3.08 |
| Chloropentafluorobenzene | 122.10 | 223.17 | 224.06 | -0.89 | -0.40 |
| Quinoline | 122.10 | 199.20 | 224.06 | -24.86 | -12.48 |
| Isoquinoline | 122.20 | 196.16 | 224.24 | -28.08 | -14.32 |
| 2,2,3-Trichlorobutyraldehyde | 122.30 | 241.80 | 224.43 | 17.37 | 7.18 |
| Ethyl acetoacetate | 122.30 | 241.80 | 224.43 | 17.37 | 7.18 |
| 1-Heptene | 122.70 | 211.79 | 225.16 | -13.37 | -6.31 |
| Isobutyl acetate | 123.00 | 233.80 | 225.71 | 8.09 | 3.46 |
| Methyl pivalate | 123.10 | 223.00 | 225.89 | -2.89 | -1.30 |
| 1-Chloro-3-isocyanatobenzene | 123.10 | 187.00 | 225.89 | -38.89 | -20.80 |
| n-Hexanoic acid | 123.30 | 225.10 | 226.26 | -1.16 | -0.52 |
| 3,3-Bis(chloromethyl)oxetane | 123.30 | 218.80 | 226.26 | -7.46 | -3.41 |
| 3-Nitrotoluene | 123.30 | 202.50 | 226.26 | -23.76 | -11.73 |
| endo-2-Methylnorbornane | 123.30 | 184.30 | 226.26 | -41.96 | -22.77 |
| 4-Nitrotoluene | 123.30 | 172.40 | 226.26 | -53.86 | -31.24 |
| 1,5-Dichloropentane | 123.40 | 213.40 | 226.45 | -13.05 | -6.11 |
| Methyl valerate | 123.50 | 226.11 | 226.63 | -0.52 | -0.23 |
| n-Hexylamine | 123.50 | 246.00 | 226.63 | 19.37 | 7.87 |
| exo-2-Methylnorbornane | 123.50 | 185.80 | 226.63 | -40.83 | -21.97 |
| n-Butyl acetate | 123.60 | 225.10 | 226.81 | -1.71 | -0.76 |
| Nitrocyclohexane | 123.60 | 258.32 | 226.81 | 31.51 | 12.20 |
| Propyl propanoate | 123.80 | 226.65 | 227.18 | -0.53 | -0.23 |
| Ethyl butanoate | 123.80 | 220.10 | 227.18 | -7.08 | -3.22 |
| Dipropylamine | 123.90 | 252.70 | 227.36 | 25.34 | 10.03 |
| 2,3,4,5,6-Pentafluorotoluene | 124.00 | 225.80 | 227.55 | -1.75 | -0.77 |
| cis-Bicyclo[3.3.0]octane | 124.10 | 213.40 | 227.73 | -14.33 | -6.71 |
| trans-Bicyclo[3.3.0]octane | 124.10 | 180.30 | 227.73 | -47.43 | -26.31 |
| s-Phenethyl alcohol | 124.20 | 257.45 | 227.91 | 29.54 | 11.47 |
| Phenethyl alcohol | 124.50 | 252.64 | 228.46 | 24.18 | 9.57 |
| cis-Bicyclo[4,2,0]octane | 124.60 | 258.60 | 228.65 | 29.95 | 11.58 |
| 1,4-Dimethylpiperazine | 124.90 | 210.11 | 229.20 | -19.09 | -9.08 |
| Heptamethylenimine | 125.30 | 230.00 | 229.93 | 0.07 | 0.03 |
| Cyclohexanemethanol | 125.30 | 236.50 | 229.93 | 6.57 | 2.78 |
| cis-2-Methylcyclohexanol | 125.30 | 268.95 | 229.93 | 39.02 | 14.51 |
| 1-Methylcyclohexanol | 125.30 | 279.05 | 229.93 | 49.12 | 17.60 |
| Cycloheptanol | 125.50 | 250.22 | 230.30 | 19.92 | 7.96 |
| cis- Cyclooctene | 125.50 | 207.80 | 230.30 | -22.50 | -10.83 |
| N-Methylvaleramide | 125.70 | 238.40 | 230.66 | 7.74 | 3.24 |
| Ethoxybenzene | 125.90 | 228.50 | 231.03 | -2.53 | -1.11 |
| 1-Chlorohexane | 125.90 | 216.20 | 231.03 | -14.83 | -6.86 |
| 1,2,4-Trichlorobenzene | 126.00 | 194.90 | 231.21 | -36.31 | -18.63 |
| Butylacetamide | 126.10 | 236.89 | 231.40 | 5.49 | 2.32 |
| 2,6-Xylidine | 126.10 | 238.86 | 231.40 | 7.46 | 3.12 |
| trans-4-Methylcyclohexanol | 126.10 | 202.10 | 231.40 | -29.30 | -14.50 |
| trans-2-Methylcyclohexanol | 126.10 | 262.98 | 231.40 | 31.58 | 12.01 |
| cis-3-Methylcyclohexanol | 126.10 | 199.60 | 231.40 | -31.80 | -15.93 |
| 2-Bromoiodobenzene | 126.10 | 179.90 | 231.40 | -51.50 | -28.63 |
| 3-Bromoiodobenzene | 126.40 | 183.10 | 231.95 | -48.85 | -26.68 |
| Triethylborane | 126.50 | 240.00 | 232.13 | 7.87 | 3.28 |
| alpha-Methylstyrene | 126.50 | 202.10 | 232.13 | -30.03 | -14.86 |
| Naphthalene | 126.50 | 196.06 | 232.13 | -36.07 | -18.40 |
| Hexyleneglycol | 126.60 | 263.10 | 232.32 | 30.78 | 11.70 |
| Ethylidenecyclohexane | 126.90 | 203.80 | 232.87 | -29.07 | -14.26 |
| Triptane | 127.20 | 213.51 | 233.42 | -19.91 | -9.32 |
| 2-Phenylethylamine | 127.30 | 239.24 | 233.60 | 5.64 | 2.36 |
| 3,3-Dimethylpentane | 127.40 | 214.80 | 233.78 | -18.98 | -8.84 |
| 2,2-Dimethylpentane | 127.70 | 221.12 | 234.33 | -13.21 | -5.98 |
| 3-Ethylpentane | 127.70 | 219.58 | 234.33 | -14.75 | -6.72 |
| 2,3-Dimethylpentane | 127.90 | 218.30 | 234.70 | -16.40 | -7.51 |
| 2,4-Dimethylpentane | 128.00 | 224.22 | 234.88 | -10.66 | -4.76 |
| Heptane | 128.20 | 225.33 | 235.25 | -9.92 | -4.40 |
| N,N-Dimethylaniline | 128.20 | 213.40 | 235.25 | -21.85 | -10.24 |
| N-Ethylaniline | 128.30 | 239.30 | 235.43 | 3.87 | 1.62 |
| 2-Methylhexane | 128.30 | 222.92 | 235.43 | -12.51 | -5.61 |
| Allylcyclopentane | 128.50 | 202.90 | 235.80 | -32.90 | -16.22 |
| Methyl benzoate | 128.70 | 221.30 | 236.17 | -14.87 | -6.72 |
| Dimethyl maleate | 128.80 | 263.20 | 236.35 | 26.85 | 10.20 |
| 1-Ethylpiperidine | 129.00 | 232.06 | 236.72 | -4.66 | -2.01 |
| 2,4-Dibromophenol | 129.10 | 259.40 | 236.90 | 22.50 | 8.67 |
| Indole-3-carboxyaldehyde | 129.40 | 220.50 | 237.45 | -16.95 | -7.69 |
| cis-2,4,6-Trimethyl-1,3,5-trioxane | 129.40 | 257.30 | 237.45 | 19.85 | 7.71 |
| 2-t-Butoxyethanol | 129.40 | 273.45 | 237.45 | 36.00 | 13.16 |
| 1,2-Hexanediol | 129.40 | 293.10 | 237.45 | 55.65 | 18.99 |
| 2,4-Dimethyl-3-pentanone | 129.50 | 233.70 | 237.63 | -3.93 | -1.68 |
| Diisopropyl sulfide | 129.50 | 232.00 | 237.63 | -5.63 | -2.43 |
| Cyclohexyl formate | 129.60 | 219.70 | 237.82 | -18.12 | -8.25 |
| 3,4-Dimethylpentanal | 129.70 | 235.60 | 238.00 | -2.40 | -1.02 |
| 2-Methyl-3-hexanone | 129.70 | 241.50 | 238.00 | 3.50 | 1.45 |
| Cinnamaldehyde | 129.80 | 232.60 | 238.19 | -5.59 | -2.40 |
| 4-Heptanone | 129.80 | 246.30 | 238.19 | 8.11 | 3.29 |
| 1,4-Diisocyanatobutane | 130.10 | 225.06 | 238.74 | -13.68 | -6.08 |
| Hexyl bromide | 130.10 | 219.70 | 238.74 | -19.04 | -8.66 |
| 1-Hexanethiol | 130.20 | 230.71 | 238.92 | -8.21 | -3.56 |
| Butylcellosolve | 130.20 | 271.66 | 238.92 | 32.74 | 12.05 |
| 3-Methylhexanal | 130.30 | 246.00 | 239.10 | 6.90 | 2.80 |
| 2-Heptanone | 130.40 | 243.60 | 239.29 | 4.31 | 1.77 |
| Dipropyl sulfide | 130.50 | 225.48 | 239.47 | -13.99 | -6.20 |
| Pentafluoronitrobenzene | 130.50 | 272.96 | 239.47 | 33.49 | 12.27 |
| 1,1-Diethoxyethane | 130.60 | 237.70 | 239.65 | -1.95 | -0.82 |
| 2-Diethylaminoethanol | 130.80 | 281.20 | 240.02 | 41.18 | 14.64 |
| 2,5-Dioxaoctane | 130.90 | 248.90 | 240.20 | 8.70 | 3.49 |
| n-Heptaldehyde | 131.00 | 230.78 | 240.39 | -9.61 | -4.16 |
| Diethyl cellosolve | 131.00 | 259.40 | 240.39 | 19.01 | 7.33 |
| Ethyl cyclobutanecarboxylate | 131.20 | 213.00 | 240.75 | -27.75 | -13.03 |
| 3,4-Dihydro-1H-2-benzopyran | 131.30 | 217.77 | 240.94 | -23.17 | -10.64 |
| 3-Oxabicyclo[3.2.2]nonane | 131.40 | 185.06 | 241.12 | -56.06 | -30.29 |
| 1,1-Dimethoxy-3-cyanopropane | 131.60 | 253.60 | 241.49 | 12.11 | 4.78 |
| Hemimellitene | 131.60 | 216.44 | 241.49 | -25.05 | -11.57 |
| 1,5-Dibromopentane | 131.70 | 220.84 | 241.67 | -20.83 | -9.43 |
| 3,4-Dihydro-2H-1-benzopyran | 131.70 | 214.00 | 241.67 | -27.67 | -12.93 |
| 1,2,4-Trimethylbenzene | 131.90 | 213.11 | 242.04 | -28.93 | -13.57 |
| Cumene | 132.20 | 215.40 | 242.59 | -27.19 | -12.62 |
| Isocumene | 132.20 | 214.72 | 242.59 | -27.87 | -12.98 |
| 1,2-Diiodobenzene | 132.20 | 190.00 | 242.59 | -52.59 | -27.68 |
| Dichlorodiethylsilane | 132.50 | 214.70 | 243.14 | -28.44 | -13.25 |
| Ethyl trichloroacetate | 132.60 | 230.10 | 243.32 | -13.22 | -5.75 |
| 1-Phenylpyrazole | 133.00 | 231.60 | 244.06 | -12.46 | -5.38 |
| 1,1-Dimethylcyclohexane | 133.00 | 209.24 | 244.06 | -34.82 | -16.64 |
| trans-1,3-Dimethylcyclohexane | 133.10 | 212.84 | 244.24 | -31.40 | -14.75 |
| cis-1,4-Dimethylcyclohexane | 133.10 | 212.09 | 244.24 | -32.15 | -15.16 |
| trans-1,2-Dimethylcyclohexane | 133.40 | 209.41 | 244.79 | -35.38 | -16.89 |
| 1,3,5-Trimethylbenzene | 133.50 | 207.85 | 244.97 | -37.12 | -17.86 |
| Ethylcyclohexane | 133.60 | 211.79 | 245.16 | -33.37 | -15.75 |
| Diisobutylene | 133.70 | 240.20 | 245.34 | -5.14 | -2.14 |
| Cyclooctane | 133.70 | 215.53 | 245.34 | -29.81 | -13.83 |
| trans-1,4-Dimethylcyclohexane | 133.80 | 210.25 | 245.52 | -35.27 | -16.78 |
| cis-1,3-Dimethylcyclohexane | 133.80 | 209.37 | 245.52 | -36.15 | -17.27 |
| 3-Azabicyclo[3.2.2]nonane | 133.90 | 238.90 | 245.71 | -6.81 | -2.85 |
| 1,2,3,4-Tetrahydroquinoline | 133.90 | 236.04 | 245.71 | -9.67 | -4.10 |
| 5,6,7,8-Tetrahydroquinoline | 134.00 | 217.41 | 245.89 | -28.48 | -13.10 |
| Propylcyclopentane | 134.10 | 216.27 | 246.07 | -29.80 | -13.78 |
| 1-Phenylimidazole | 134.40 | 249.10 | 246.62 | 2.48 | 0.99 |
| Ethyl 2,3-dichloropropionate | 134.60 | 248.90 | 246.99 | 1.91 | 0.77 |
| Tetramethyoxysilane | 134.60 | 240.50 | 246.99 | -6.49 | -2.70 |
| n-Butyl acrylate | 134.80 | 251.00 | 247.36 | 3.64 | 1.45 |
| Veratrole | 134.80 | 274.00 | 247.36 | 26.64 | 9.72 |
| 2-Phenoxyethanol | 134.80 | 294.63 | 247.36 | 47.27 | 16.04 |
| 1-Phenyl-1-propanone | 135.10 | 243.20 | 247.91 | -4.71 | -1.94 |
| 1-Octyne | 135.10 | 242.14 | 247.91 | -5.77 | -2.38 |
| 4-Octyne | 135.20 | 233.64 | 248.09 | -14.45 | -6.19 |
| Ethyl N,N-dimethylglycinate | 135.30 | 244.20 | 248.27 | -4.07 | -1.67 |
| Dimethyl methylmalonate | 135.40 | 261.70 | 248.46 | 13.24 | 5.06 |
| 1-Naphthol | 135.40 | 284.50 | 248.46 | 36.04 | 12.67 |
| Triethylmethanol | 135.50 | 353.90 | 248.64 | 105.26 | 29.74 |
| Methyl trichlorothioacrylate | 135.60 | 244.90 | 248.82 | -3.92 | -1.60 |
| Ethanediol diacetate | 135.90 | 269.20 | 249.38 | 19.82 | 7.36 |
| 2,2-Dimethyl-3-pentanol | 135.90 | 349.00 | 249.38 | 99.62 | 28.55 |
| 2,4-Dimethyl-3-pentanol | 135.90 | 359.00 | 249.38 | 109.62 | 30.54 |
| Diethyl oxalate | 136.30 | 260.70 | 250.11 | 10.59 | 4.06 |
| 1-Iodohexane | 136.30 | 222.50 | 250.11 | -27.61 | -12.41 |
| 1-Piperazineethanamine | 136.30 | 284.90 | 250.11 | 34.79 | 12.21 |
| 2-Methyl-2-hexanol | 136.70 | 313.54 | 250.84 | 62.70 | 20.00 |
| 2-Methyl-3-hexanol | 136.70 | 324.00 | 250.84 | 73.16 | 22.58 |
| Ethyl t-amyl ether | 136.80 | 243.00 | 251.03 | -8.03 | -3.30 |
| 5-Methyl-2-hexanol | 136.80 | 295.20 | 251.03 | 44.17 | 14.96 |
| 1,3,5-Trimethylhexahydro-s-triazine | 137.00 | 237.75 | 251.39 | -13.64 | -5.74 |
| 2-Heptanol | 137.20 | 298.63 | 251.76 | 46.87 | 15.70 |
| 4-Heptanol | 137.20 | 306.77 | 251.76 | 55.01 | 17.93 |
| 3-Heptanol | 137.20 | 314.20 | 251.76 | 62.44 | 19.87 |
| 1-Heptanol | 137.30 | 274.81 | 251.94 | 22.87 | 8.32 |
| 3-Nitrobenzotrifluoride | 137.30 | 223.80 | 251.94 | -28.14 | -12.58 |
| Perfluorotoluene | 137.90 | 262.30 | 253.04 | 9.26 | 3.53 |
| 8-Methylquinoline | 138.30 | 224.50 | 253.78 | -29.28 | -13.04 |
| Quinaldine | 138.40 | 224.72 | 253.96 | -29.24 | -13.01 |
| 1,2,3,4-Tetrahydronaphthalene | 138.50 | 217.44 | 254.14 | -36.70 | -16.88 |
| Dipropylene glycol | 138.80 | 322.10 | 254.69 | 67.41 | 20.93 |
| trans-2-Octene | 139.10 | 239.30 | 255.25 | -15.95 | -6.66 |
| Caprylene | 139.40 | 241.21 | 255.80 | -14.59 | -6.05 |
| Enanthic acid | 139.80 | 267.31 | 256.53 | 10.78 | 4.03 |
| Isoamyl acetate | 139.90 | 251.20 | 256.71 | -5.51 | -2.19 |
| cis-Hydrindan | 139.90 | 214.18 | 256.71 | -42.53 | -19.86 |
| Ethyl pentanoate | 140.10 | 256.81 | 257.08 | -0.27 | -0.10 |
| Ethyl pivalate | 140.10 | 251.50 | 257.08 | -5.58 | -2.22 |
| 1,6-Dichlorohexane | 140.10 | 239.57 | 257.08 | -17.51 | -7.31 |
| trans-Hydrindan | 140.10 | 209.70 | 257.08 | -47.38 | -22.59 |
| Sulcatone | 140.20 | 268.80 | 257.26 | 11.54 | 4.29 |
| Methyl hexanoate | 140.30 | 245.30 | 257.45 | -12.15 | -4.95 |
| Amyl acetate | 140.30 | 276.10 | 257.45 | 18.65 | 6.76 |
| Diethylene glycol dimethyl ether | 140.40 | 274.10 | 257.63 | 16.47 | 6.01 |
| 1-Chloronaphthalene | 140.50 | 212.60 | 257.81 | -45.21 | -21.27 |
| Hydrocinnamyl alcohol | 141.10 | 280.74 | 258.91 | 21.83 | 7.77 |
| Trichlorotoluene | 141.10 | 218.67 | 258.91 | -40.24 | -18.40 |
| cis-Bicyclo[6.1.0]nonane | 142.00 | 235.10 | 260.56 | -25.46 | -10.83 |
| Cyclohexaneethanol | 142.50 | 266.00 | 261.48 | 4.52 | 1.70 |
| 1-Methylnaphthalene | 142.50 | 224.39 | 261.48 | -37.09 | -16.53 |
| 1-Chloroheptane | 142.60 | 245.40 | 261.67 | -16.27 | -6.63 |
| N,N-Dimethyl-o-toluidine | 142.80 | 280.30 | 262.03 | 18.27 | 6.52 |
| Isooctane | 142.90 | 242.49 | 262.22 | -19.73 | -8.13 |
| 2-Methylnaphthalene | 142.90 | 228.00 | 262.22 | -34.22 | -15.01 |
| 2,3,4-Trimethylpentane | 143.20 | 247.32 | 262.77 | -15.45 | -6.25 |
| 1-Ethyl-3-methylimidazolium bromide | 143.80 | 264.80 | 263.87 | 0.93 | 0.35 |
| 2,3,3-Trimethylpentane | 143.90 | 245.56 | 264.05 | -18.49 | -7.53 |
| Methyl o-toluate | 143.90 | 242.50 | 264.05 | -21.55 | -8.89 |
| 3-Phenylpropylamine | 144.00 | 265.59 | 264.23 | 1.36 | 0.51 |
| 3,3-Dimethylhexane | 144.00 | 246.60 | 264.23 | -17.63 | -7.15 |
| 2,4-Dinitrotoluene | 144.10 | 255.20 | 264.42 | -9.22 | -3.61 |
| 2,2,3-Trimethylpentane | 144.10 | 245.60 | 264.42 | -18.82 | -7.66 |
| 3-Methylheptane | 144.20 | 250.20 | 264.60 | -14.40 | -5.76 |
| Benzyl acetate | 144.20 | 250.20 | 264.60 | -14.40 | -5.76 |
| 2,5-Dimethylhexane | 144.20 | 249.20 | 264.60 | -15.40 | -6.18 |
| 2-Methylheptane | 144.30 | 252.00 | 264.78 | -12.78 | -5.07 |
| Cyclohexyl acetate | 144.50 | 245.40 | 265.15 | -19.75 | -8.05 |
| Allylcyclohexane | 144.50 | 233.50 | 265.15 | -31.65 | -13.55 |
| 2-(Phenoxymethyl)oxirane | 144.80 | 276.00 | 265.70 | 10.30 | 3.73 |
| 4-Methylheptane | 144.80 | 251.08 | 265.70 | -14.62 | -5.82 |
| Octane | 144.90 | 255.68 | 265.88 | -10.20 | -3.99 |
| Methyl m-toluate | 145.00 | 248.70 | 266.07 | -17.37 | -6.98 |
| Ethyl benzoate | 145.50 | 246.00 | 266.99 | -20.99 | -8.53 |
| 2,5,6-Trimethyl-2-cyclohexen-1-one | 146.10 | 258.40 | 268.09 | -9.69 | -3.75 |
| 1,7-Heptanediol | 146.20 | 297.00 | 268.27 | 28.73 | 9.67 |
| 2,4,6-Trimethyl-2-cyclohexen-1-one | 146.50 | 258.60 | 268.82 | -10.22 | -3.95 |
| Tetrahydrodicyclopentadiene | 146.50 | 236.50 | 268.82 | -32.32 | -13.67 |
| Heptyl bromide | 146.80 | 249.41 | 269.37 | -19.96 | -8.00 |
| 1-Heptanethiol | 146.90 | 259.32 | 269.55 | -10.23 | -3.95 |
| 2-Ethylhexanal | 147.00 | 267.80 | 269.74 | -1.94 | -0.72 |
| 2-Octanone | 147.10 | 273.26 | 269.92 | 3.34 | 1.22 |
| Prehnitene | 147.30 | 244.30 | 270.29 | -25.99 | -10.64 |
| 2,5-Dioxanonane | 147.60 | 282.00 | 270.84 | 11.16 | 3.96 |
| Caprylic aldehyde | 147.70 | 259.40 | 271.02 | -11.62 | -4.48 |
| Phthaloyl chloride | 148.20 | 248.50 | 271.94 | -23.44 | -9.43 |
| p-Cymene | 148.60 | 242.30 | 272.67 | -30.37 | -12.53 |
| t-Butylbenzene | 148.60 | 241.59 | 272.67 | -31.08 | -12.87 |
| Triethyleneglycol | 148.80 | 327.60 | 273.04 | 54.56 | 16.65 |
| Butylbenzene | 148.90 | 243.50 | 273.22 | -29.72 | -12.21 |
| 2-Hydroxyethyl-2',2'-dimethylpropionate | 148.90 | 308.00 | 273.22 | 34.78 | 11.29 |
| Dipropyl disulfide | 149.40 | 262.46 | 274.14 | -11.68 | -4.45 |
| 1,2,4,5-Tetramethylbenzene | 149.50 | 220.10 | 274.32 | -54.22 | -24.64 |
| Propylcyclohexane | 150.30 | 242.04 | 275.79 | -33.75 | -13.94 |
| Dipropyl carbonate | 150.50 | 275.55 | 276.16 | -0.61 | -0.22 |
| 2,4-Toluene diisocyanate | 150.60 | 287.80 | 276.34 | 11.46 | 3.98 |
| Butylcyclopentane | 150.70 | 245.35 | 276.52 | -31.17 | -12.71 |
| 4-Ethylacetophenone | 151.00 | 260.70 | 277.07 | -16.37 | -6.28 |
| Heptanedioic acid | 151.00 | 334.30 | 277.07 | 57.23 | 17.12 |
| Phenyltrichlorosilane | 151.20 | 220.70 | 277.44 | -56.74 | -25.71 |
| Di-t-butyl ether | 151.40 | 276.10 | 277.81 | -1.71 | -0.62 |
| n-Butylmethacrylate | 151.40 | 273.80 | 277.81 | -4.01 | -1.46 |
| Methyl dimethylmalonate | 151.60 | 259.30 | 278.18 | -18.88 | -7.28 |
| trans-Decahydroquinoline | 151.60 | 254.81 | 278.18 | -23.37 | -9.17 |
| 3,3-Dinitroazetidinium nitrate | 151.70 | 272.95 | 278.36 | -5.41 | -1.98 |
| 3-Carene | 152.20 | 254.26 | 279.28 | -25.02 | -9.84 |
| 4-Ethyl-3-hexanol | 152.20 | 361.26 | 279.28 | 81.98 | 22.69 |
| 2-Acetoxyanisole | 152.30 | 278.50 | 279.46 | -0.96 | -0.34 |
| Diethyl malonate | 152.30 | 285.00 | 279.46 | 5.54 | 1.94 |
| Ethyl salicylate | 152.70 | 283.07 | 280.19 | 2.88 | 1.02 |
| 4-Methyl-2-heptanol | 152.70 | 312.50 | 280.19 | 32.31 | 10.34 |
| 3-Methyl-2-heptanol | 152.80 | 297.50 | 280.38 | 17.12 | 5.76 |
| 4-Methyl-3-heptanol | 152.80 | 309.20 | 280.38 | 28.82 | 9.32 |
| 3-Methyl-4-heptanol | 152.80 | 355.78 | 280.38 | 75.40 | 21.19 |
| 2,5-Dimethyl-3-hexanol | 152.90 | 374.15 | 280.56 | 93.59 | 25.01 |
| 1-Iodoheptane | 153.00 | 251.80 | 280.74 | -28.94 | -11.49 |
| 2-Ethyl-1-hexanol | 153.00 | 317.50 | 280.74 | 36.76 | 11.58 |
| 4-Methyl-4-heptanol | 153.00 | 366.92 | 280.74 | 86.18 | 23.49 |
| 5-Methyl-2-heptanol | 153.10 | 296.20 | 280.93 | 15.27 | 5.16 |
| 2-Methyl-4-heptanol | 153.10 | 331.80 | 280.93 | 50.87 | 15.33 |
| 5-Methyl-1-heptanol | 153.20 | 304.20 | 281.11 | 23.09 | 7.59 |
| Biphenyl | 153.30 | 259.54 | 281.29 | -21.75 | -8.38 |
| 6-Methyl-3-heptanol | 153.40 | 310.50 | 281.48 | 29.02 | 9.35 |
| 2-Methyl-2-heptanol | 153.40 | 337.60 | 281.48 | 56.12 | 16.62 |
| 2-Methyl-1-heptanol | 153.50 | 313.00 | 281.66 | 31.34 | 10.01 |
| 6-Methyl-2-heptanol | 153.50 | 315.10 | 281.66 | 33.44 | 10.61 |
| 1-Octanol | 153.70 | 312.10 | 282.03 | 30.07 | 9.64 |
| 4-Octanol | 153.90 | 332.09 | 282.39 | 49.70 | 14.96 |
| 3-Octanol | 153.90 | 338.50 | 282.39 | 56.11 | 16.57 |
| 2-Octanol | 154.00 | 330.10 | 282.58 | 47.52 | 14.40 |
| Diisobutyl ether | 154.30 | 276.08 | 283.13 | -7.05 | -2.55 |
| 4,7-Dimethylindane | 154.30 | 241.50 | 283.13 | -41.63 | -17.24 |
| Camphor | 154.40 | 271.20 | 283.31 | -12.11 | -4.47 |
| 1,1-Dimethylindane | 154.40 | 249.40 | 283.31 | -33.91 | -13.60 |
| N-Methyl-2-hydroxyethylammonium propionate | 154.40 | 328.00 | 283.31 | 44.69 | 13.62 |
| Di-n-butylether | 154.50 | 278.16 | 283.49 | -5.33 | -1.92 |
| 4,6-Dimethylindane | 154.50 | 240.90 | 283.49 | -42.59 | -17.68 |
| Dihydropinene | 155.30 | 251.26 | 284.96 | -33.70 | -13.41 |
| 1-Nonene | 156.10 | 270.36 | 286.43 | -16.07 | -5.94 |
| trans-Decalin | 156.10 | 229.17 | 286.43 | -57.26 | -24.99 |
| Bicyclopentyl | 156.50 | 238.90 | 287.16 | -48.26 | -20.20 |
| n-Octanoic acid | 156.60 | 304.00 | 287.35 | 16.65 | 5.48 |
| Isopentyl propionate | 156.70 | 285.20 | 287.53 | -2.33 | -0.82 |
| n-Octylamine | 156.90 | 302.00 | 287.90 | 14.10 | 4.67 |
| Fenchone | 156.90 | 269.80 | 287.90 | -18.10 | -6.71 |
| Methylenanthate | 157.00 | 285.10 | 288.08 | -2.98 | -1.05 |
| Butyl butanoate | 157.00 | 282.00 | 288.08 | -6.08 | -2.16 |
| n-Hexylacetate | 157.20 | 282.80 | 288.45 | -5.65 | -2.00 |
| Amyl propionate | 157.20 | 245.20 | 288.45 | -43.25 | -17.64 |
| Di-n-butylamine | 157.30 | 292.90 | 288.63 | 4.27 | 1.46 |
| Tetraethylmethane | 157.70 | 278.20 | 289.36 | -11.16 | -4.01 |
| 2,2,4,4-Tetramethylpentane | 158.60 | 266.30 | 291.02 | -24.72 | -9.28 |
| Butanoic anhydride | 158.80 | 283.70 | 291.38 | -7.68 | -2.71 |
| Cyclohexanepropanol | 159.20 | 293.00 | 292.12 | 0.88 | 0.30 |
| 2,7-Dimethylnaphthalene | 159.20 | 251.85 | 292.12 | -40.27 | -15.99 |
| 1-Chlorooctane | 159.30 | 274.70 | 292.30 | -17.60 | -6.41 |
| Pulegone | 159.70 | 274.90 | 293.03 | -18.13 | -6.60 |
| 2,2,3,3-Tetramethylpentane | 159.90 | 271.50 | 293.40 | -21.90 | -8.07 |
| 1-Methyl-4-isopropylcyclohexene | 160.20 | 257.80 | 293.95 | -36.15 | -14.02 |
| Di-t-butylsulfide | 160.70 | 281.74 | 294.87 | -13.13 | -4.66 |
| 1-Propyl-3-methylimidazolium bromide | 160.70 | 281.40 | 294.87 | -13.47 | -4.79 |
| N-(2-Trimethylsilylethyl)ethylenimine | 160.80 | 300.80 | 295.05 | 5.75 | 1.91 |
| 4-Methoxycarbonylhomocubane | 160.80 | 288.00 | 295.05 | -7.05 | -2.45 |
| N,N-Diethylaniline | 160.80 | 274.50 | 295.05 | -20.55 | -7.49 |
| Nonane | 161.50 | 284.43 | 296.34 | -11.91 | -4.19 |
| 4-Allyl-2-methoxyphenol | 161.80 | 343.10 | 296.89 | 46.21 | 13.47 |
| 2,6-Dimethylheptan-4-one | 161.90 | 297.30 | 297.07 | 0.23 | 0.08 |
| Ethyl cyclohexanecarboxylate | 162.40 | 271.50 | 297.99 | -26.49 | -9.76 |
| 5-Nonanone | 163.20 | 303.60 | 299.45 | 4.15 | 1.37 |
| 1,6-Hexamethylene diisocyanate | 163.50 | 294.00 | 300.00 | -6.00 | -2.04 |
| 1-Bromooctane | 163.50 | 272.16 | 300.00 | -27.84 | -10.23 |
| 1-Octanethiol | 163.60 | 300.40 | 300.19 | 0.21 | 0.07 |
| Di-n-butylsulfide | 163.90 | 284.34 | 300.74 | -16.40 | -5.77 |
| 2,4,4-Trimethyl-2-nitropentane | 164.10 | 232.48 | 301.11 | -68.63 | -29.52 |
| n-Nonanal | 164.30 | 290.40 | 301.47 | -11.07 | -3.81 |
| 1,2-Dipropoxyethane | 164.40 | 309.00 | 301.66 | 7.34 | 2.38 |
| 1,8-Cineole | 164.40 | 270.72 | 301.66 | -30.94 | -11.43 |
| 2,4,5-Trinitrotoluene | 164.60 | 311.70 | 302.02 | 9.68 | 3.10 |
| Neopentylbenzene | 165.20 | 271.90 | 303.12 | -31.22 | -11.48 |
| 1-Ethyl-3-methylimidazolium thiocyanate | 165.40 | 281.45 | 303.49 | -22.04 | -7.83 |
| t-Butylcyclohexane | 165.50 | 264.80 | 303.67 | -38.87 | -14.68 |
| 1-Ethyl-3-methylimidazolium acetate | 166.00 | 321.90 | 304.59 | 17.31 | 5.38 |
| N-Phenylpiperidine | 166.00 | 324.11 | 304.59 | 19.52 | 6.02 |
| 1-Methyltetrahydrothiophenium dicyanamide | 166.00 | 338.50 | 304.59 | 33.91 | 10.02 |
| Perfluoromethyldiethylamine | 166.10 | 337.46 | 304.77 | 32.69 | 9.69 |
| 2-(2-(2-Methoxyethoxy)ethoxy)ethanol | 166.10 | 357.05 | 304.77 | 52.28 | 14.64 |
| Hexamethyldisilane | 166.40 | 256.10 | 305.32 | -49.22 | -19.22 |
| Neral | 166.90 | 304.60 | 306.24 | -1.64 | -0.54 |
| Butylcyclohexane | 167.00 | 271.04 | 306.42 | -35.38 | -13.06 |
| Octanedioic acid | 167.20 | 366.20 | 306.79 | 59.41 | 16.22 |
| 1-Phenyl-1-methylsilacyclobutane | 167.60 | 273.20 | 307.53 | -34.33 | -12.56 |
| Hexamethylphosphoramide | 167.80 | 321.30 | 307.89 | 13.41 | 4.17 |
| N-Cyclopentylpiperidine | 167.90 | 282.02 | 308.08 | -26.06 | -9.24 |
| Hexamethyldisiloxane | 168.30 | 309.09 | 308.81 | 0.28 | 0.09 |
| Diethyl succinate | 168.40 | 330.50 | 308.99 | 21.51 | 6.51 |
| Ethylene glycol dipropionate | 169.00 | 331.80 | 310.09 | 21.71 | 6.54 |
| 1-Ethyl-3-methylimidazolium tetrafluoroborate | 169.10 | 305.00 | 310.28 | -5.28 | -1.73 |
| 1,I-Dimethylazoxyethane | 169.90 | 316.50 | 311.74 | 4.76 | 1.50 |
| 3,7-Dimethyl-6-octen-1-yn-3-ol | 170.00 | 385.30 | 311.93 | 73.37 | 19.04 |
| 1,4-Dibromo-2,3-dichlorohexafluorobutane | 170.40 | 298.50 | 312.66 | -14.16 | -4.74 |
| 1-Ethyl-3-methylimidazolium hydrogen sulfate | 170.50 | 295.50 | 312.85 | -17.35 | -5.87 |
| 2-Phenyltoluene | 170.50 | 275.77 | 312.85 | -37.08 | -13.44 |
| Diphenyl sulfide | 170.60 | 271.10 | 313.03 | -41.93 | -15.47 |
| Diphenylmethane | 170.60 | 266.10 | 313.03 | -46.93 | -17.64 |
| 4-Nonanol | 170.60 | 367.86 | 313.03 | 54.83 | 14.91 |
| 5-Nonanol | 170.60 | 370.75 | 313.03 | 57.72 | 15.57 |
| 3-Nonanol | 170.60 | 373.63 | 313.03 | 60.60 | 16.22 |
| 1-Nonanol | 170.70 | 341.00 | 313.21 | 27.79 | 8.15 |
| 2-Nonanol | 170.70 | 356.32 | 313.21 | 43.11 | 12.10 |
| Benzophenone | 170.90 | 289.59 | 313.58 | -23.99 | -8.28 |
| 1-Butyl-3-methylimidazolium chloride | 171.10 | 317.00 | 313.95 | 3.05 | 0.96 |
| Tetraethylsilane | 171.10 | 297.70 | 313.95 | -16.25 | -5.46 |
| N-Methyl-2-hydroxyethylammonium butanoate | 171.10 | 361.00 | 313.95 | 47.05 | 13.03 |
| Dimethylphenylvinylsilane | 171.90 | 284.80 | 315.41 | -30.61 | -10.75 |
| Tripropylamine | 172.00 | 301.00 | 315.60 | -14.60 | -4.85 |
| Dimethyl phthalate | 172.30 | 303.10 | 316.15 | -13.05 | -4.30 |
| Benzyl methacrylate | 172.30 | 269.90 | 316.15 | -46.25 | -17.13 |
| trans-Geraniol | 172.60 | 346.10 | 316.70 | 29.40 | 8.50 |
| 1-Decene | 172.80 | 300.83 | 317.06 | -16.23 | -5.40 |
| 1,3-Dimethylimidazolium methosulfate | 172.80 | 341.00 | 317.06 | 23.94 | 7.02 |
| Ethyl trans-cinnamate | 172.90 | 296.36 | 317.25 | -20.89 | -7.05 |
| Pelargonic acid | 173.20 | 362.37 | 317.80 | 44.57 | 12.30 |
| Diethyleneglycol monobutyl ether | 173.30 | 354.89 | 317.98 | 36.91 | 10.40 |
| Methyl octanoate | 173.40 | 314.23 | 318.17 | -3.94 | -1.25 |
| Pentyl butyrate | 173.90 | 311.70 | 319.08 | -7.38 | -2.37 |
| Diethyleneglycol diethyl ether | 174.00 | 347.50 | 319.27 | 28.23 | 8.12 |
| Linalool | 174.20 | 372.40 | 319.63 | 52.77 | 14.17 |
| 5-Trimethylsilyl-2-norbornene | 174.60 | 290.40 | 320.37 | -29.97 | -10.32 |
| Benzophenoneimine | 175.70 | 333.48 | 322.38 | 11.10 | 3.33 |
| 1-Chlorononane | 176.00 | 296.44 | 322.93 | -26.49 | -8.94 |
| 1-Ethyl-3-methylimidazolium dicyanamide | 176.10 | 314.64 | 323.12 | -8.48 | -2.69 |
| 5-Methylnonane | 177.30 | 314.43 | 325.32 | -10.89 | -3.46 |
| N -(2-Trimethylsilylethyl)trimethylenimine | 177.40 | 304.60 | 325.50 | -20.90 | -6.86 |
| Ethyl hydrocinnamate | 177.50 | 286.60 | 325.69 | -39.09 | -13.64 |
| 4-Methylnonane | 177.60 | 317.36 | 325.87 | -8.51 | -2.68 |
| 1-Ethylferrocene | 177.60 | 335.30 | 325.87 | 9.43 | 2.81 |
| 3-Methylnonane | 177.60 | 308.99 | 325.87 | -16.88 | -5.46 |
| 1-Butyl-3-methylimidazolium bromide | 177.70 | 317.00 | 326.05 | -9.05 | -2.86 |
| 1-Ethyladamantane | 177.70 | 258.45 | 326.05 | -67.60 | -26.16 |
| 2-Methylnonane | 178.00 | 313.30 | 326.60 | -13.30 | -4.25 |
| Tolan | 178.00 | 297.50 | 326.60 | -29.10 | -9.78 |
| Decane | 178.20 | 316.32 | 326.97 | -10.65 | -3.37 |
| 1,3-Dimethyladamantane | 178.20 | 258.31 | 326.97 | -68.66 | -26.58 |
| cis,trans,trans-1,5,9-Cyclododecatriene | 178.40 | 287.76 | 327.34 | -39.58 | -13.75 |
| Cyclohexyl butanoate | 178.60 | 300.70 | 327.70 | -27.00 | -8.98 |
| beta-Citronellol | 179.30 | 357.90 | 328.99 | 28.91 | 8.08 |
| 1-Ethyl-3-methylimidazolium methanesulfonate | 180.00 | 345.52 | 330.27 | 15.25 | 4.41 |
| Hexamethylbenzene | 180.00 | 370.70 | 330.27 | 40.43 | 10.91 |
| 1-Bromononane | 180.20 | 308.32 | 330.64 | -22.32 | -7.24 |
| 1,1-Diphenylethylene | 180.70 | 299.20 | 331.56 | -32.36 | -10.81 |
| trans-Stilbene | 180.80 | 343.10 | 331.74 | 11.36 | 3.31 |
| Di-t-butyldisulfide | 180.90 | 313.63 | 331.92 | -18.29 | -5.83 |
| Bis(trimethylsilyl)methane | 180.90 | 357.50 | 331.92 | 25.58 | 7.15 |
| Capraldehyde | 181.00 | 319.70 | 332.11 | -12.41 | -3.88 |
| 1-Ethyl-3-methylimidazolium trifluoroacetate | 181.00 | 316.00 | 332.11 | -16.11 | -5.10 |
| 1,2,3,4-Tetrahydrophenanthrene | 181.10 | 278.26 | 332.29 | -54.03 | -19.42 |
| N-Methyldiphenylamine | 181.60 | 301.20 | 333.21 | -32.01 | -10.63 |
| Diphenylmethylamine | 182.00 | 301.20 | 333.94 | -32.74 | -10.87 |
| Perfluoromethylcyclohexane | 182.80 | 353.10 | 335.41 | 17.69 | 5.01 |
| 2,5,8,11-Tetraoxododecane | 183.40 | 367.78 | 336.51 | 31.27 | 8.50 |
| 1-Ethyl-3-methylimidazolium methylphosphonate | 183.60 | 354.64 | 336.88 | 17.76 | 5.01 |
| Diphenylcarbodiimide | 183.70 | 304.60 | 337.06 | -32.46 | -10.66 |
| N-Cyclohexylpiperidine | 184.10 | 287.76 | 337.79 | -50.03 | -17.39 |
| Benzhydryl chloride | 184.20 | 290.40 | 337.98 | -47.58 | -16.38 |
| Nonanedioic acid | 184.40 | 398.10 | 338.34 | 59.76 | 15.01 |
| Tetraethylurea | 184.50 | 345.70 | 338.53 | 7.17 | 2.07 |
| 1-Ethyltetrahydrothiophenium dicyanamide | 184.90 | 335.38 | 339.26 | -3.88 | -1.16 |
| 1,1,4,7-Tetramethylindane | 185.10 | 302.50 | 339.63 | -37.13 | -12.27 |
| 2-Ethylbiphenyl | 185.20 | 302.73 | 339.81 | -37.08 | -12.25 |
| Benzyldimethylvinylsilane | 185.40 | 312.20 | 340.18 | -27.98 | -8.96 |
| 4-Propyl-4-heptanol | 185.70 | 446.60 | 340.73 | 105.87 | 23.71 |
| 1,2-Diphenylethane | 186.10 | 320.10 | 341.46 | -21.36 | -6.67 |
| 3,7-Dimethyl-1-octanol | 186.10 | 367.21 | 341.46 | 25.75 | 7.01 |
| 1-Butyl-3-methylimidazolium nitrate | 186.20 | 353.50 | 341.65 | 11.85 | 3.35 |
| 3,3'-Bitolyl | 186.20 | 295.45 | 341.65 | -46.20 | -15.64 |
| 2,2'-Dimethylbiphenyl | 186.60 | 298.06 | 342.38 | -44.32 | -14.87 |
| 2-Methyldiphenylmethane | 186.60 | 296.58 | 342.38 | -45.80 | -15.44 |
| 1,1-Diphenylethane | 186.80 | 295.00 | 342.75 | -47.75 | -16.19 |
| n-Decyl alcohol | 187.00 | 377.00 | 343.11 | 33.89 | 8.99 |
| Diisoamyl ether | 187.20 | 379.00 | 343.48 | 35.52 | 9.37 |
| 5-Decanol | 187.30 | 405.77 | 343.66 | 62.11 | 15.31 |
| 1-Propylpyridinium tetrafluoroborate | 187.40 | 363.00 | 343.85 | 19.15 | 5.28 |
| 2,3-Dihydro-1,1,4,6-tetramethyl-1H-indene | 187.50 | 299.60 | 344.03 | -44.43 | -14.83 |
| N-Methyl-2-hydroxyethylammonium pentanoate | 187.80 | 401.00 | 344.58 | 56.42 | 14.07 |
| Bicyclohexyl | 188.70 | 283.00 | 346.23 | -63.23 | -22.34 |
| 3-Methylbenzophenone | 189.10 | 325.10 | 346.97 | -21.87 | -6.73 |
| 1-Ethyl-3-methylimidazolium methylsulfate | 189.20 | 341.00 | 347.15 | -6.15 | -1.80 |
| 1-Undecene | 189.50 | 329.95 | 347.70 | -17.75 | -5.38 |
| 1-Ethyl-3-methylimidazolium tricyanomethide | 189.90 | 358.70 | 348.43 | 10.27 | 2.86 |
| n-Decanoic acid | 189.90 | 361.10 | 348.43 | 12.67 | 3.51 |
| n-Octylacetate | 190.00 | 339.70 | 348.62 | -8.92 | -2.62 |
| Methyl nonanoate | 190.10 | 345.63 | 348.80 | -3.17 | -0.92 |
| n-Decylamine | 190.30 | 361.00 | 349.17 | 11.83 | 3.28 |
| Ethyl caprylate | 190.50 | 343.13 | 349.53 | -6.40 | -1.87 |
| 1-Ethyl-3-methylimidazolium hexafluorophosphate | 190.60 | 343.60 | 349.72 | -6.12 | -1.78 |
| 1-Butyl-3-methylimidazolium iodide | 190.80 | 314.00 | 350.08 | -36.08 | -11.49 |
| Tris(pentafluoroethyl)amine | 191.00 | 379.49 | 350.45 | 29.04 | 7.65 |
| Tetraglycol | 191.80 | 428.90 | 351.92 | 76.98 | 17.95 |
| 2-(2'-Hydroxyethoxy)ethyl pivalate | 191.90 | 368.83 | 352.10 | 16.73 | 4.54 |
| 1-Benzyl-3-methylimidazolium chloride | 192.00 | 339.40 | 352.28 | -12.88 | -3.80 |
| 1-(2-Hydroxyethyl)-3-methyl-1H-imidazolium 2,2,2-trifluoroacetate | 192.20 | 369.00 | 352.65 | 16.35 | 4.43 |
| 1-Ethylpyridinium hexafluorophosphate | 192.30 | 293.04 | 352.84 | -59.80 | -20.41 |
| delta-Undecanolactone | 193.60 | 342.70 | 355.22 | -12.52 | -3.65 |
| N-Benzylbenzaldehyde imine | 193.70 | 384.66 | 355.40 | 29.26 | 7.61 |
| 1-Propylferrocene | 194.50 | 319.50 | 356.87 | -37.37 | -11.70 |
| 2,4,6,8-Tetramethylcyclotetrasiloxane | 194.70 | 354.40 | 357.24 | -2.84 | -0.80 |
| 2-Methyldecane | 194.70 | 341.21 | 357.24 | -16.03 | -4.70 |
| Undecane | 194.90 | 345.05 | 357.60 | -12.55 | -3.64 |
| Cyclohexyl valerate | 195.10 | 323.00 | 357.97 | -34.97 | -10.83 |
| 1-Ethyl-3-methylimidazolium trifluoromethylsulfonate | 195.20 | 362.80 | 358.15 | 4.65 | 1.28 |
| Hexamethylcyclotrisiloxane | 195.90 | 360.00 | 359.44 | 0.56 | 0.16 |
| 1-Bromodecane | 196.90 | 334.40 | 361.27 | -26.87 | -8.04 |
| 6-Undecanone | 197.00 | 362.70 | 361.46 | 1.24 | 0.34 |
| 1-Decanethiol | 197.00 | 350.41 | 361.46 | -11.05 | -3.15 |
| Bis(piperidino)methane | 197.10 | 364.62 | 361.64 | 2.98 | 0.82 |
| Dibutyl acetal | 197.30 | 352.20 | 362.01 | -9.81 | -2.78 |
| Glyceryltriacetate | 197.30 | 384.70 | 362.01 | 22.69 | 5.90 |
| 1,2-Dibutoxyethane | 197.80 | 350.00 | 362.92 | -12.92 | -3.69 |
| 1-Butyl-3-methylimidazolium thiocyanate | 198.70 | 385.00 | 364.58 | 20.42 | 5.31 |
| 1-(1-Isocyanato-1-methylethyl-3-(1-methylethenyl)benzene | 200.20 | 382.00 | 367.33 | 14.67 | 3.84 |
| 1-Butyl-3-methylimidazolium acetate | 200.70 | 383.20 | 368.24 | 14.96 | 3.90 |
| 2-Ethylhexyl acrylate | 201.00 | 355.10 | 368.79 | -13.69 | -3.86 |
| Decanedioic acid | 201.10 | 430.00 | 368.98 | 61.02 | 14.19 |
| Tetraethoxysilane | 201.60 | 364.40 | 369.90 | -5.50 | -1.51 |
| Ethylene glycol dibutyrate | 202.00 | 380.30 | 370.63 | 9.67 | 2.54 |
| 1-Butyl-3-methylimidazolium tetrafluoroborate | 202.30 | 363.00 | 371.18 | -8.18 | -2.25 |
| 4-Isopropylbiphenyl | 202.70 | 343.90 | 371.91 | -28.01 | -8.15 |
| beta-Ionone | 203.80 | 382.80 | 373.93 | 8.87 | 2.32 |
| Perfluoroheptane | 203.80 | 419.00 | 373.93 | 45.07 | 10.76 |
| 1-Butylpyridinium tetrafluoroborate | 204.00 | 383.80 | 374.30 | 9.50 | 2.48 |
| 1-Undecanol | 204.10 | 406.34 | 374.48 | 31.86 | 7.84 |
| Diethyl phthalate | 204.20 | 366.10 | 374.66 | -8.56 | -2.34 |
| 1-Ethyl-3-methylimidazolium ethosulfate | 204.60 | 378.00 | 375.40 | 2.60 | 0.69 |
| 1-Methoxydecane | 204.80 | 370.80 | 375.77 | -4.97 | -1.34 |
| 1-Methylazepanium methosulfate | 205.30 | 332.08 | 376.68 | -44.60 | -13.43 |
| 1-Dodecene | 206.20 | 360.66 | 378.33 | -17.67 | -4.90 |
| n-Undecanoic acid | 206.50 | 415.10 | 378.88 | 36.22 | 8.72 |
| Methyl decanoate | 207.10 | 372.03 | 379.98 | -7.95 | -2.14 |
| 1-Propyl-3-methylimidazolium hexafluorophosphate | 207.50 | 374.40 | 380.72 | -6.32 | -1.69 |
| Diethyl terephthalate | 207.60 | 380.70 | 380.90 | -0.20 | -0.05 |
| 2,2,4,6,6-Pentamethylheptane | 207.60 | 350.98 | 380.90 | -29.92 | -8.53 |
| 1-Butyl-3-methylimidazolium dicyanoamide | 208.00 | 365.00 | 381.64 | -16.64 | -4.56 |
| 1-Propylpyridinium hexafluorophosphate | 208.90 | 328.70 | 383.29 | -54.59 | -16.61 |
| 3-Nitro-3-(4-nitrophenyl)pentane | 209.30 | 409.79 | 384.02 | 25.77 | 6.29 |
| 1-Ethyl-3-methylimidazolium dimethylphosphate | 209.30 | 411.78 | 384.02 | 27.76 | 6.74 |
| Cyclam | 210.10 | 374.61 | 385.49 | -10.88 | -2.90 |
| Pseudoionone | 210.20 | 382.70 | 385.67 | -2.97 | -0.78 |
| 1-Hexyl-3-methylimidazolium bromide | 210.20 | 344.00 | 385.67 | -41.67 | -12.11 |
| 1,1'-Diethylferrocene | 210.40 | 348.20 | 386.04 | -37.84 | -10.87 |
| 6,10-Dimethyl-4,5,9-undecatrien-2-one | 211.30 | 382.70 | 387.69 | -4.99 | -1.30 |
| 1-Butylferrocene | 211.30 | 363.80 | 387.69 | -23.89 | -6.57 |
| 1-(3-Cyanopropyl)-pyridinium dicyanamide | 211.50 | 422.00 | 388.06 | 33.94 | 8.04 |
| Dodecane | 211.60 | 376.10 | 388.24 | -12.14 | -3.23 |
| 1-Isobutylferrocene | 212.10 | 346.90 | 389.16 | -42.26 | -12.18 |
| 1-Bromoundecane | 213.60 | 373.23 | 391.91 | -18.68 | -5.00 |
| 1,4-Di-t-butylbenzene | 213.60 | 347.84 | 391.91 | -44.07 | -12.67 |
| Tris-(carboethoxy)methane | 213.80 | 419.80 | 392.27 | 27.53 | 6.56 |
| Tripropylene glycol | 214.30 | 440.60 | 393.19 | 47.41 | 10.76 |
| 1-Butyl-3-methylimidazolium trifluoroacetate | 214.70 | 408.20 | 393.93 | 14.27 | 3.50 |
| Tridecanolactone | 215.50 | 398.30 | 395.39 | 2.91 | 0.73 |
| 1,3-Bis(trimethylsilyl)propane | 215.60 | 394.34 | 395.58 | -1.24 | -0.31 |
| 1-Butyl-2-methylimidazolium trifluoroacetate | 216.80 | 407.90 | 397.78 | 10.12 | 2.48 |
| Heptylcyclohexane | 217.00 | 363.20 | 398.14 | -34.94 | -9.62 |
| NHTPN | 217.80 | 380.14 | 399.61 | -19.47 | -5.12 |
| Undecanedioic acid | 217.80 | 461.90 | 399.61 | 62.29 | 13.49 |
| 1-Butyltetrahydrothiophenium dicyanamide | 218.20 | 395.19 | 400.35 | -5.16 | -1.31 |
| Octyl methacrylate | 218.20 | 386.00 | 400.35 | -14.35 | -3.72 |
| 4-t-Butylbiphenyl | 218.20 | 383.60 | 400.35 | -16.75 | -4.37 |
| 1-Butyl-2,3-dimethylimidazolium tetrafluoroborate | 218.70 | 416.01 | 401.26 | 14.75 | 3.54 |
| Nonyl acrylate | 218.70 | 417.90 | 401.26 | 16.64 | 3.98 |
| 1-Butyl-4-methylpyridinium tetrafluoroborate | 219.00 | 414.00 | 401.81 | 12.19 | 2.94 |
| 1-Butyl-3-methylpyridinium tetrafluoroborate | 219.00 | 388.00 | 401.81 | -13.81 | -3.56 |
| 1-Dodecanol | 220.80 | 438.42 | 405.12 | 33.30 | 7.60 |
| 1-Ethyl-3-methylpyridinium ethylsulfate | 221.30 | 389.00 | 406.03 | -17.03 | -4.38 |
| 1-Butyl-3-methylimidazolium methosulfate | 221.40 | 416.00 | 406.22 | 9.78 | 2.35 |
| Tributylamine | 222.50 | 391.83 | 408.23 | -16.40 | -4.19 |
| 1H,1H-Perfluorooctan-1-ol | 222.70 | 468.60 | 408.60 | 60.00 | 12.80 |
| 1-Tridecene | 222.90 | 391.80 | 408.97 | -17.17 | -4.38 |
| 1-Benzyl-3-methylimidazolium tetrafluoroborate | 222.90 | 387.50 | 408.97 | -21.47 | -5.54 |
| Lauric acid | 223.20 | 404.28 | 409.52 | -5.24 | -1.30 |
| Dibenzyldisulfide | 223.60 | 420.71 | 410.25 | 10.46 | 2.49 |
| Methyl undecylate | 223.80 | 406.24 | 410.62 | -4.38 | -1.08 |
| m-Terphenyl | 223.80 | 417.10 | 410.62 | 6.48 | 1.55 |
| Ethyl decanoate | 223.80 | 403.34 | 410.62 | -7.28 | -1.80 |
| Hexyl hexanoate | 223.80 | 394.92 | 410.62 | -15.70 | -3.98 |
| Butyl octanoate | 223.90 | 397.41 | 410.80 | -13.39 | -3.37 |
| Octyl butanoate | 223.90 | 383.28 | 410.80 | -27.52 | -7.18 |
| o-Terphenyl | 224.00 | 369.05 | 410.99 | -41.94 | -11.36 |
| 1-Butyl-3-methylimidazolium hexafluorophosphate | 224.10 | 407.70 | 411.17 | -3.47 | -0.85 |
| Perfluorobicyclo[4.4.0]dec-1,6-ene | 225.10 | 428.40 | 413.00 | 15.40 | 3.59 |
| 1-Butyl-1-methylpyrrolidinium dicyanamide | 225.80 | 413.00 | 414.29 | -1.29 | -0.31 |
| 2,5,8,11,14-Pentaoxopentadecane | 226.40 | 457.10 | 415.39 | 41.71 | 9.13 |
| 1-(3-Cyanopropyl)-2,3-dimethylimidazolium dicyanamide | 227.80 | 444.00 | 417.96 | 26.04 | 5.87 |
| 1-Butyl-3-methylimidazolium trifluoromethylsulfonate | 228.20 | 417.00 | 418.69 | -1.69 | -0.41 |
| 6,10-Dimethyl-2-undecanone | 228.50 | 428.40 | 419.24 | 9.16 | 2.14 |
| Tridecane | 228.60 | 407.10 | 419.42 | -12.32 | -3.03 |
| 1,1,3,3,5,5-Hexamethyl-1,3,5-trisilacyclohexane | 229.00 | 400.95 | 420.16 | -19.21 | -4.79 |
| Diethynyldiphenylsilane | 229.00 | 382.00 | 420.16 | -38.16 | -9.99 |
| 1-Bromododecane | 230.30 | 399.31 | 422.54 | -23.23 | -5.82 |
| 1,6-Dibromo-2,3,5-trichlorononafluorohexane | 230.40 | 418.60 | 422.73 | -4.13 | -0.99 |
| n-Dodecylmercaptan | 230.40 | 442.70 | 422.73 | 19.97 | 4.51 |
| 1,3-Bis-(1-isocyanato-1-methylethyl)benzene | 231.70 | 464.00 | 425.11 | 38.89 | 8.38 |
| Octamethyltrisiloxane | 231.80 | 420.64 | 425.29 | -4.65 | -1.11 |
| 1,4-Bis-(1-isocyanato-1-methylethyl)benzene | 231.80 | 415.10 | 425.29 | -10.19 | -2.46 |
| Dodecanedioic acid | 234.50 | 493.80 | 430.25 | 63.55 | 12.87 |
| 1-Hexyl-3-methylimidazolium tetrafluoroborate | 234.70 | 416.00 | 430.61 | -14.61 | -3.51 |
| Pentaglycol | 234.80 | 515.50 | 430.80 | 84.70 | 16.43 |
| Nonyl methacrylate | 234.90 | 419.30 | 430.98 | -11.68 | -2.79 |
| Dibutyl succinate | 235.20 | 436.24 | 431.53 | 4.71 | 1.08 |
| Tributylmethanol | 235.70 | 548.60 | 432.45 | 116.15 | 21.17 |
| Perfluoro-cis-decalin | 237.50 | 449.80 | 435.75 | 14.05 | 3.12 |
| 1-Tridecanol | 237.50 | 476.00 | 435.75 | 40.25 | 8.46 |
| 1,2-Diethylpyridinium ethylsulfate | 238.20 | 412.00 | 437.03 | -25.03 | -6.08 |
| Perfluoro-trans-decalin | 239.60 | 446.80 | 439.60 | 7.20 | 1.61 |
| Methyl laurate | 240.20 | 432.68 | 440.70 | -8.02 | -1.85 |
| 1-Butyl-2,3-dimethylimidazolium hexafluorophosphate | 240.70 | 433.60 | 441.62 | -8.02 | -1.85 |
| Diethyleneglycol-di-n-butylether | 240.80 | 452.00 | 441.80 | 10.20 | 2.26 |
| 1-Pentyl-3-methylimidazolium hexafluorophosphate | 241.00 | 437.40 | 442.17 | -4.77 | -1.09 |
| 1-Butyl-1-methylpyrrolidinium trifluoromethanesulfonate | 243.50 | 435.00 | 446.76 | -11.76 | -2.70 |
| Tolbutamide | 244.60 | 450.81 | 448.77 | 2.04 | 0.45 |
| Tetradecane | 244.90 | 438.90 | 449.32 | -10.42 | -2.38 |
| Tripropionine | 245.00 | 481.30 | 449.51 | 31.79 | 6.61 |
| 2-Tetradecanone | 247.30 | 415.20 | 453.73 | -38.53 | -9.28 |
| 1-Octyl-3-methylimidazolium bromide | 247.30 | 392.00 | 453.73 | -61.73 | -15.75 |
| 1H,1H-Perfluorononan-1-ol | 250.30 | 515.90 | 459.23 | 56.67 | 10.98 |
| Decylcyclopentane | 250.50 | 426.52 | 459.60 | -33.08 | -7.76 |
| 1-Ethyl-3-methylimidazolium toluenesulfonate | 251.00 | 484.20 | 460.51 | 23.69 | 4.89 |
| 1,13-Tridecanedioic acid | 251.20 | 525.70 | 460.88 | 64.82 | 12.33 |
| Decyl methacrylate | 251.70 | 452.90 | 461.80 | -8.90 | -1.96 |
| Pentaethylene glycol monomethyl ether | 252.10 | 537.12 | 462.53 | 74.59 | 13.89 |
| Myristyl alcohol | 254.20 | 505.80 | 466.38 | 39.42 | 7.79 |
| Oxacyclohexadecan-2-one | 255.20 | 444.60 | 468.22 | -23.62 | -5.31 |
| 1-Methyl-3-propylimidazolium 2-amino-4-carboxybutanoate | 255.20 | 517.10 | 468.22 | 48.88 | 9.45 |
| Tetrapropylene glycol | 255.30 | 559.80 | 468.40 | 91.40 | 16.33 |
| Octamethylcyclotetrasiloxane | 255.90 | 495.94 | 469.50 | 26.44 | 5.33 |
| 1-Hexyl-3-methylimidazolium hexafluorophosphate | 256.70 | 424.00 | 470.97 | -46.97 | -11.08 |
| Ethyl dodecanoate | 256.90 | 464.00 | 471.34 | -7.34 | -1.58 |
| 1,4,7,10,13,16-Hexaoxacyclooctadecane | 258.10 | 460.00 | 473.54 | -13.54 | -2.94 |
| 1-Hexyl-3-methylimidazolium trifluoromethylsulfonate | 261.40 | 502.30 | 479.59 | 22.71 | 4.52 |
| Pentadecane | 261.60 | 469.90 | 479.96 | -10.06 | -2.14 |
| 1,1,3,3-Tetraethyl-5,5-dimethylcyclotrisiloxane | 262.00 | 502.90 | 480.69 | 22.21 | 4.42 |
| 3,7,11-Trimethyl-1-dodecen-3-ol | 262.20 | 574.50 | 481.06 | 93.44 | 16.26 |
| 1-Tetradecanethiol | 263.70 | 501.70 | 483.81 | 17.89 | 3.57 |
| 1-Bromotetradecane | 263.70 | 450.86 | 483.81 | -32.95 | -7.31 |
| Octamethyltetrasiloxane | 263.90 | 509.60 | 484.18 | 25.42 | 4.99 |
| Ethyl 1-ethylnicotinate ethosulfate | 265.10 | 513.00 | 486.38 | 26.62 | 5.19 |
| Decylcyclohexane | 266.70 | 455.60 | 489.31 | -33.71 | -7.40 |
| 1,14-Tetradecanedioic acid | 267.90 | 557.60 | 491.52 | 66.08 | 11.85 |
| N-(4-Methoxybenzylidene)-4-n-butylaniline | 268.10 | 477.00 | 491.88 | -14.88 | -3.12 |
| 1-Butyl-1-methylpyrrolidinium tetracyanoborate | 268.30 | 524.00 | 492.25 | 31.75 | 6.06 |
| Tetrapropyl silicate | 268.40 | 460.10 | 492.43 | -32.33 | -7.03 |
| 1-Octyl-3-methylimidazolium tetrafluoroborate | 268.60 | 498.00 | 492.80 | 5.20 | 1.04 |
| 1-Ethyl-3-methylimidazolium bis(trifluoromethanesulfonyl) amide | 268.70 | 500.00 | 492.98 | 7.02 | 1.40 |
| Pentaethylene glycol dimethyl ether | 269.50 | 547.44 | 494.45 | 52.99 | 9.68 |
| 1-Benzyl-3-methylimidazolium tetrafluoroethanesulfonate | 269.70 | 502.80 | 494.82 | 7.98 | 1.59 |
| 4-Cyano-3-fluorophenyl 4-butylbenzoate | 269.80 | 478.16 | 495.00 | -16.84 | -3.52 |
| 1-Butyl-3-methylpyridinium tetracyanoborate | 270.70 | 495.00 | 496.65 | -1.65 | -0.33 |
| N-Ethylpyridinium bis(trifluoromethylsulfonyl)amide | 270.90 | 502.15 | 497.02 | 5.13 | 1.02 |
| 1-Pentadecanol | 270.90 | 535.10 | 497.02 | 38.08 | 7.12 |
| 2,2,4,4,6,8,8-Heptamethylnonane | 271.30 | 458.80 | 497.75 | -38.95 | -8.49 |
| Nonyl N-phenylcarbamate | 272.40 | 471.10 | 499.77 | -28.67 | -6.09 |
| Dibutyl phthalate | 272.70 | 476.00 | 500.32 | -24.32 | -5.11 |
| Cetene | 273.00 | 485.83 | 500.87 | -15.04 | -3.10 |
| Methyl tetradecanoate | 273.60 | 497.91 | 501.97 | -4.06 | -0.82 |
| 1-Ethyl-3-methylimidazolium 2-(2-methoxyethoxy)ethylsulfate | 274.00 | 526.00 | 502.71 | 23.29 | 4.43 |
| 1H,1H-Perfluorodecan-1-ol | 274.50 | 563.90 | 503.62 | 60.28 | 10.69 |
| 1-Heptyl-3-methylimidazolium hexafluorophosphate | 274.60 | 500.60 | 503.81 | -3.21 | -0.64 |
| 1,1,3,3-Tetramethyl-1,3-diphenyldisiloxane | 277.60 | 508.10 | 509.31 | -1.21 | -0.24 |
| Hexaethylene glycol | 277.80 | 620.10 | 509.68 | 110.42 | 17.81 |
| Cetane | 278.20 | 501.61 | 510.41 | -8.80 | -1.75 |
| 4-Butyl-4'-ethoxyazobenzene | 281.80 | 535.10 | 517.01 | 18.09 | 3.38 |
| 1-Ethyl-2,3-dimethylimidazolium bis(trifluoromethanesulfonyl) amide | 282.60 | 493.00 | 518.48 | -25.48 | -5.17 |
| 1-Methyl-3-butylimidazolium saccharinate | 284.30 | 565.66 | 521.60 | 44.06 | 7.79 |
| 1-Isopropyl-3-methylimidazolium bis(trifluoromethanesulfonyl) amide | 284.90 | 529.90 | 522.70 | 7.20 | 1.36 |
| 1-Propyl-3-methylimidazolium bis(trifluoromethanesulfonyl) amide | 285.10 | 534.90 | 523.07 | 11.83 | 2.21 |
| 1-Butyl-3-methylimidazolium toluenesulfonate | 285.80 | 548.40 | 524.35 | 24.05 | 4.39 |
| N-Ethyl-2-methylpyridinium bis(trifluoromethylsulfonyl)amide | 286.50 | 534.50 | 525.64 | 8.86 | 1.66 |
| 1,1,1,3,5,5,5-Heptamethyl-3-phenyltrisiloxane | 287.30 | 519.60 | 527.10 | -7.50 | -1.44 |
| N,N-Dimethyl-2-pentylnonylamine | 288.10 | 538.10 | 528.57 | 9.53 | 1.77 |
| Ethyl myristate | 290.20 | 526.32 | 532.42 | -6.10 | -1.16 |
| Di-n-octylamine | 290.90 | 507.20 | 533.71 | -26.51 | -5.23 |
| 1-Octyl-3-methylimidazolium hexafluorophosphate | 291.40 | 536.10 | 534.62 | 1.48 | 0.28 |
| Hexaethylcyclohexane | 293.30 | 530.10 | 538.11 | -8.01 | -1.51 |
| Decamethyltetrasiloxane | 294.20 | 538.77 | 539.76 | -0.99 | -0.18 |
| 1-Octyl-3-methylimidazolium trifluoromethylsulfonate | 294.80 | 577.70 | 540.86 | 36.84 | 6.38 |
| Heptadecane | 294.90 | 534.34 | 541.04 | -6.70 | -1.25 |
| Hexaethylcyclotrisiloxane | 295.30 | 535.10 | 541.78 | -6.68 | -1.25 |
| 1-Cyclopropylmethyl-3-methylimidazolium bis(trifluoromethanesulfonyl) amide | 295.80 | 539.10 | 542.70 | -3.60 | -0.67 |
| Glyceryl tributanoate | 296.10 | 555.30 | 543.25 | 12.05 | 2.17 |
| 1-Hexadecanethiol | 297.10 | 574.00 | 545.08 | 28.92 | 5.04 |
| Trimethyl butylammonium bis(trifluoromethylsulfonyl)amide | 298.10 | 559.20 | 546.91 | 12.29 | 2.20 |
| 6,10,14-Trimethylpentadeca-3,5-diene-2-one | 298.70 | 555.20 | 548.02 | 7.18 | 1.29 |
| 1,2-Diethylpyridinium bis(trifluoromethanesulfonyl) amide | 299.20 | 566.10 | 548.93 | 17.17 | 3.03 |
| 1-Butyl-3-methylimidazolium bis(trifluoromethanesulfonyl) amide | 301.00 | 536.00 | 552.23 | -16.23 | -3.03 |
| 1-sec-Butyl-3-methylimidazolium bis(trifluoromethanesulfonyl) amide | 301.20 | 557.10 | 552.60 | 4.50 | 0.81 |
| Hexadecanedioic acid | 301.30 | 621.40 | 552.78 | 68.62 | 11.04 |
| 1-Methyl-1-propylpyrrolidinium bis(trifluoromethanesulfonyl) amide | 301.70 | 554.00 | 553.52 | 0.48 | 0.09 |
| 1-Isobutyl-3-methylimidazolium bis(trifluoromethanesulfonyl) amide | 301.70 | 557.10 | 553.52 | 3.58 | 0.64 |
| 1-Propyl-2,3-dimethylimidazolium bis(trifluoromethanesulfonyl) amide | 301.90 | 554.50 | 553.89 | 0.61 | 0.11 |
| 1H,1H-Perfluoroundecan-1-ol | 302.10 | 602.40 | 554.25 | 48.15 | 7.99 |
| N-Propyl-2-methylpyridinium bis(trifluoromethylsulfonyl)amide | 303.10 | 557.96 | 556.09 | 1.87 | 0.34 |
| N-Butylpyridinium bis(trifluoromethanesulfonyl) amide | 303.60 | 566.52 | 557.00 | 9.52 | 1.68 |
| N-Propyl-3-methylpyridinium bis(trifluoromethylsulfonyl)amide | 303.90 | 517.00 | 557.55 | -40.55 | -7.84 |
| 1-Nonyl-3-methylimidazolium hexafluorophosphate | 307.30 | 569.40 | 563.79 | 5.61 | 0.99 |
| N-Octylisoquinolinium thiocyanate | 309.80 | 522.00 | 568.38 | -46.38 | -8.88 |
| Octadecane | 311.60 | 564.40 | 571.68 | -7.28 | -1.29 |
| Pentapropylene glycol | 312.40 | 685.80 | 573.15 | 112.65 | 16.43 |
| N-Ethyl-4-dimethylaminopyridinium bis(trifluoromethanesulfonyl) amide | 315.50 | 594.30 | 578.83 | 15.47 | 2.60 |
| (9Z)-Octadecenoic acid | 317.00 | 577.00 | 581.58 | -4.58 | -0.79 |
| 1-Pentyl-3-methylimidazolium bis(trifluoromethanesulfonyl) amide | 317.10 | 595.60 | 581.77 | 13.83 | 2.32 |
| Decamethylcyclopentasiloxane | 318.70 | 634.63 | 584.70 | 49.93 | 7.87 |
| 1-Ethyl-2-propylpyridinium bis(trifluoromethanesulfonyl) amide | 318.90 | 593.90 | 585.07 | 8.83 | 1.49 |
| 1-Isobutyl-1-methylpyrrolidinium bis(trifluoromethylsulfonyl)amide | 319.80 | 582.20 | 586.72 | -4.52 | -0.78 |
| 1-Isobutyl-3-methylpyridinium bis(trifluoromethylsulfonyl)amide | 320.10 | 579.00 | 587.27 | -8.27 | -1.43 |
| N-Butyl-3-methylpyridinium bis(trifluoromethylsulfonyl)amide | 320.40 | 578.10 | 587.82 | -9.72 | -1.68 |
| 1-Butyl-1-methylpyrrolidinium bis(trifluoromethylsulfonyl)amide | 320.80 | 572.00 | 588.56 | -16.56 | -2.89 |
| 1-Butyl-3-cyanopyridinium bis(trifluoromethylsulfonyl)amide | 321.60 | 586.00 | 590.02 | -4.02 | -0.69 |
| Trihexylamine | 322.40 | 568.12 | 591.49 | -23.37 | -4.11 |
| 1-Benzyl-3-methylimidazolium bis(trifluoromethylsulfonyl) amide | 322.60 | 607.80 | 591.86 | 15.94 | 2.62 |
| 1-Decyl-3-methylimidazolium hexafluorophosphate | 324.70 | 603.30 | 595.71 | 7.59 | 1.26 |
| Pristane | 325.30 | 569.76 | 596.81 | -27.05 | -4.75 |
| 1-Cyclopentylmethyl-3-methylimidazolium bis(trifluoromethanesulfonyl) amide | 325.40 | 560.60 | 596.99 | -36.39 | -6.49 |
| Ricinelaidic acid | 325.80 | 646.00 | 597.73 | 48.27 | 7.47 |
| 1-Chlorooctadecane | 326.30 | 606.70 | 598.64 | 8.06 | 1.33 |
| 1H,1H-Perfluorododecan-1-ol | 328.00 | 645.20 | 601.76 | 43.44 | 6.73 |
| Tri-p-cresyl phosphate | 328.10 | 578.00 | 601.95 | -23.95 | -4.14 |
| Nonadecane | 328.30 | 604.00 | 602.31 | 1.69 | 0.28 |
| Tri(butyl) methylphosphonium methylsulfate | 328.90 | 617.80 | 603.41 | 14.39 | 2.33 |
| Stearyl mercaptan | 330.50 | 648.00 | 606.35 | 41.65 | 6.43 |
| Methyltributylammonium serinate | 331.10 | 635.00 | 607.45 | 27.55 | 4.34 |
| Perfluorododecane | 333.40 | 546.80 | 611.67 | -64.87 | -11.86 |
| 1,1,1,5,5,5-Hexamethyl-3,3-diphenyltrisiloxane | 333.60 | 648.00 | 612.04 | 35.96 | 5.55 |
| 1-Hexyl-3-methylimidazolium bis(trifluoromethanesulfonyl) amide | 335.00 | 629.20 | 614.60 | 14.60 | 2.32 |
| Tetrabutoxysilane | 335.20 | 580.20 | 614.97 | -34.77 | -5.99 |
| Dibutyl decanedioate | 336.00 | 619.00 | 616.44 | 2.56 | 0.41 |
| 1-Ethyl-2-butylpyridinium bis(trifluoromethanesulfonyl) amide | 336.30 | 623.60 | 616.99 | 6.61 | 1.06 |
| 1-Butyl-1-methylpiperidinium bis(trifluoromethylsulfonyl)amide | 336.30 | 607.50 | 616.99 | -9.49 | -1.56 |
| N-Hexylpyridinium bis(trifluoromethanesulfonyl) amide | 336.70 | 612.00 | 617.72 | -5.72 | -0.94 |
| 1-Methyl-1-pentylpyrrolidinium bis(trifluoromethanesulfonyl) amide | 337.50 | 622.60 | 619.19 | 3.41 | 0.55 |
| 1-Butyl-3-methylimidazolium octylsulfate | 338.50 | 635.00 | 621.02 | 13.98 | 2.20 |
| Methyltributylammonium L-threoninate | 338.60 | 753.00 | 621.21 | 131.79 | 17.50 |
| 3,7,11,15-Tetramethyl-1-hexadecyn-3-ol | 340.80 | 712.50 | 625.24 | 87.26 | 12.25 |
| 1-Cyclohexylmethyl-3-methylimidazolium bis(trifluoromethanesulfonyl) amide | 341.40 | 617.10 | 626.34 | -9.24 | -1.50 |
| Isophytol | 344.10 | 729.70 | 631.30 | 98.40 | 13.49 |
| Eicosane | 345.50 | 663.60 | 633.87 | 29.73 | 4.48 |
| N-Butyl-4-dimethylaminopyridinium bis(trifluoromethanesulfonyl) amide | 347.60 | 657.71 | 637.72 | 19.99 | 3.04 |
| 1-Heptyl-3-methylimidazolium bis(trifluoromethanesulfonyl) amide | 349.90 | 659.20 | 641.94 | 17.26 | 2.62 |
| 2,3-Dimethyl-1-Hexylimidazolium bis(trifluoromethanesulfonyl) amide | 350.70 | 686.00 | 643.40 | 42.60 | 6.21 |
| Heptylpentaoxyethylene | 352.40 | 722.90 | 646.52 | 76.38 | 10.57 |
| 1-Ethyl-2-pentylpyridinium bis(trifluoromethanesulfonyl) amide | 353.00 | 652.70 | 647.62 | 5.08 | 0.78 |
| 1,1-Diphenyldodecane | 353.00 | 593.70 | 647.62 | -53.92 | -9.08 |
| 1-Hexyl-3-methylpyridinium bis(trifluoromethylsulfonyl)amide | 353.60 | 624.00 | 648.72 | -24.72 | -3.96 |
| 1-Hexyl-1-methylpyrrolidinium bis(trifluoromethanesulfonyl) amide | 354.20 | 655.10 | 649.82 | 5.28 | 0.81 |
| 1-Hexyl-3-cyanopyridinium bis(trifluoromethylsulfonyl)amide | 354.70 | 658.00 | 650.74 | 7.26 | 1.10 |
| 1-Hexyl-4-cyanopyridinium bis(trifluoromethylsulfonyl)amide | 354.70 | 633.00 | 650.74 | -17.74 | -2.80 |
| Ethyl stearate | 357.40 | 605.00 | 655.69 | -50.69 | -8.38 |
| 1-Dodecyl-3-methylimidazolium hexafluorophosphate | 358.50 | 666.30 | 657.71 | 8.59 | 1.29 |
| 1-Octyl-3-methylimidazolium bis(trifluoromethanesulfonyl) amide | 368.10 | 654.00 | 675.32 | -21.32 | -3.26 |
| 1,1-Diphenyl-3,3,5,5,7,7-hexamethylcyclotetrasiloxane | 368.30 | 633.00 | 675.69 | -42.69 | -6.74 |
| 1-Methyl-1-heptylpyrrolidinium bis(trifluoromethanesulfonyl) amide | 368.50 | 685.10 | 676.06 | 9.04 | 1.32 |
| 1-Octylpyridinium bis(trifluoromethylsulfonyl)amide | 369.10 | 686.00 | 677.16 | 8.84 | 1.29 |
| 1-Ethyl-2-hexylpyridinium bis(trifluoromethanesulfonyl) amide | 369.80 | 685.50 | 678.44 | 7.06 | 1.03 |
| Hexapropylene glycol | 369.90 | 807.50 | 678.62 | 128.88 | 15.96 |
| 1,1,3,3-Tetraethyl-5,5-diphenylcylotrisiloxane | 370.10 | 629.60 | 678.99 | -49.39 | -7.84 |
| 1-Hexyl-3,5-dimethylpyridinium bis(trifluoromethylsulfonyl)amide | 370.20 | 620.00 | 679.17 | -59.17 | -9.54 |
| 1-(3,4,5,6-Perfluorohexyl)-3-methylimidazolium-3-methylimidazolium bis(trifluoromethanesulfonyl) amide | 376.10 | 725.00 | 690.00 | 35.00 | 4.83 |
| Docosane | 378.90 | 698.00 | 695.13 | 2.87 | 0.41 |
| 1H,1H-Perfluorotetradecan-1-ol | 379.80 | 725.30 | 696.78 | 28.52 | 3.93 |
| 4-Dimethylamino-1-hexylpyridinium bis(trifluoromethanesulfonyl) amide | 380.90 | 731.00 | 698.80 | 32.20 | 4.40 |
| Octaethylcyclotetrasiloxane | 385.40 | 746.00 | 707.06 | 38.94 | 5.22 |
| N-Octyl-3-methylpyridinium bis(trifluoromethanesulfonyl) amide | 386.30 | 669.00 | 708.71 | -39.71 | -5.94 |
| 1-Ethyl-2-heptylpyridinium bis(trifluoromethanesulfonyl) amide | 386.40 | 717.50 | 708.89 | 8.61 | 1.20 |
| 1-Methyl-1-octylpyrrolidinium bis(trifluoromethanesulfonyl) amide | 387.60 | 716.30 | 711.09 | 5.21 | 0.73 |
| 1-Octyl-3-cyanopyridinium bis(trifluoromethylsulfonyl)amide | 387.90 | 709.00 | 711.64 | -2.64 | -0.37 |
| Tetrabutylphosphonium L-serinate | 389.50 | 748.00 | 714.58 | 33.42 | 4.47 |
| 4'-Heptyl-m-tercyclohexyl | 393.10 | 668.60 | 721.18 | -52.58 | -7.86 |
| 4'-Heptyl-p-tercyclohexyl | 393.20 | 752.70 | 721.37 | 31.33 | 4.16 |
| 1-Hexyl-3-methylimidazolium tris(pentafluoroethyl)trifluorophosphate | 393.70 | 730.00 | 722.28 | 7.72 | 1.06 |
| N-Hexyl-3-methyl-4-dimethylaminopyridinium bis(trifluoromethanesulfonyl) amide | 393.90 | 725.00 | 722.65 | 2.35 | 0.32 |
| Butyl 1-butylnicotinate bis(trifluoromethylsulfonyl)amide | 395.30 | 707.00 | 725.22 | -18.22 | -2.58 |
| Glycerol tricaproate | 397.20 | 753.10 | 728.70 | 24.40 | 3.24 |
| Bis(2-ethylhexyl) adipate | 399.20 | 701.50 | 732.37 | -30.87 | -4.40 |
| Ethyl tri(butyl)phosphonium diethylphosphate | 400.60 | 711.00 | 734.94 | -23.94 | -3.37 |
| 1-Decyl-3-methylimidazolium bis(trifluoromethanesulfonyl) amide | 401.30 | 754.80 | 736.22 | 18.58 | 2.46 |
| 1-Ethyl-2-octylpyridinium bis(trifluoromethanesulfonyl) amide | 401.60 | 749.40 | 736.77 | 12.63 | 1.68 |
| Di-n-octyladipate | 402.60 | 701.50 | 738.61 | -37.11 | -5.29 |
| Dihexyl sebacate | 402.70 | 710.90 | 738.79 | -27.89 | -3.92 |
| DEHP | 403.70 | 708.98 | 740.63 | -31.65 | -4.46 |
| Bis(1-ethylhexyl) o-phthalate | 405.00 | 704.60 | 743.01 | -38.41 | -5.45 |
| Di-n-octyl phthalate | 406.70 | 707.10 | 746.13 | -39.03 | -5.52 |
| 1-Ethyl-2-nonylpyridinium bis(trifluoromethanesulfonyl) amide | 416.10 | 778.50 | 763.37 | 15.13 | 1.94 |
| Tetrabutylphosphonium L-valinate | 417.30 | 747.00 | 765.57 | -18.57 | -2.49 |
| 1-Methyl-1-decylpyrrolidinium bis(trifluoromethanesulfonyl) amide | 421.00 | 778.90 | 772.36 | 6.54 | 0.84 |
| Tri-n-octylamine | 422.90 | 750.80 | 775.85 | -25.05 | -3.34 |
| Pentacosane | 429.00 | 769.00 | 787.04 | -18.04 | -2.35 |
| 11-Cyclohexyleicosane | 432.40 | 787.40 | 793.27 | -5.87 | -0.75 |
| 1-Dodecyl-3-methylimidazolium bis(trifluoromethanesulfonyl) amide | 435.80 | 820.20 | 799.51 | 20.69 | 2.52 |
| 1-Ethyl-2-decylpyridinium bis(trifluoromethanesulfonyl) amide | 436.60 | 811.20 | 800.98 | 10.22 | 1.26 |
| 1-Hexyl-2-propyl-3,5-diethylpyridinium bis(trifluoromethylsulfonyl)amide | 448.10 | 766.00 | 822.07 | -56.07 | -7.32 |
| Bis(2-ethylhexyl) azelate | 450.20 | 799.70 | 825.93 | -26.23 | -3.28 |
| Heptacosane | 462.40 | 828.40 | 848.31 | -19.91 | -2.40 |
| Bis(2-ethylhexyl) sebacate | 467.30 | 849.40 | 857.29 | -7.89 | -0.93 |
| Vitamin E acetate | 500.90 | 897.90 | 918.93 | -21.03 | -2.34 |
| Dodecahydrosqualene | 506.50 | 886.36 | 929.20 | -42.84 | -4.83 |
| 11-Decylheneicosane | 527.50 | 949.80 | 967.73 | -17.93 | -1.89 |
| Unatriacontane | 529.20 | 912.10 | 970.84 | -58.74 | -6.44 |
| Glyceryl tricaprate | 595.10 | 1108.80 | 1091.73 | 17.07 | 1.54 |
| Tetra-1-octoxysilane | 602.30 | 1095.40 | 1104.94 | -9.54 | -0.87 |
| Tetradecyl trihexylphosphonium bis(trifluoromethylsulfonyl)amide | 726.80 | 1298.80 | 1333.32 | -34.52 | -2.66 |
| Tetra-1-decoxysilane | 736.00 | 1373.54 | 1350.20 | 23.34 | 1.70 |
| Trihexyltetradecylphosphonium tris(pentafluoroethyl)trifluorophosphate | 792.60 | 1441.40 | 1454.02 | -12.62 | -0.88 |
| Benzene hexa-n-heptanoate | 848.20 | 1500.00 | 1556.02 | -56.02 | -3.73 |
| Trihexyltetradecylphosphonium bis(2,2,4-trimethylpentyl)phosphinate | 875.80 | 1635.00 | 1606.65 | 28.35 | 1.73 |
| Trielaidin | 979.20 | 1841.76 | 1796.32 | 45.44 | 2.47 |
| Tristearin | 997.60 | 1787.47 | 1830.08 | -42.61 | -2.38 |
| 2,3,6,7,10,11-Hexaoctanoyloxytriphenylene | 1072.50 | 1956.10 | 1967.47 | -11.37 | -0.58 |
