# Supplementary material for: Calculation of the Isobaric Heat Capacities of the Liquid and Solid Phase of Organic Compounds at and around 298.15 K Based on Their “True” Molecular Volume
Source: Molecules. 2019 Apr 24;24(8):1626. doi: 10.3390/molecules24081626 (PMC6514989; doi:10.3390/molecules24081626)
Supplement: Supplementary file 1 [file molecules-24-01626-s001.zip › S04. Experimental and Calculated Cp(liq,298) Data Table to Figure 4.doc]

| **Molecule name** | **Molecular volume** | **Cp(liq,298) exp** | **Cp(liq,298) calc** | **Deviation** | **Dev. in %** |
| --- | --- | --- | --- | --- | --- |
| Methylamine | 40.60 | 102.10 | 67.42 | 34.68 | 33.97 |
| Methyl chloride | 42.40 | 80.80 | 70.71 | 10.09 | 12.48 |
| Formamide | 42.50 | 107.90 | 70.90 | 37.00 | 34.30 |
| Trifluoromethane | 42.90 | 86.60 | 71.63 | 14.97 | 17.29 |
| Acetonitrile | 46.20 | 91.70 | 77.67 | 14.03 | 15.30 |
| Bromomethane | 46.60 | 78.70 | 78.40 | 0.30 | 0.38 |
| Methanethiol | 46.60 | 89.10 | 78.40 | 10.70 | 12.01 |
| Oxirane | 47.00 | 87.00 | 79.13 | 7.87 | 9.04 |
| Tetrafluoromethane | 47.80 | 79.90 | 80.60 | -0.70 | -0.87 |
| Acetaldehyde | 47.90 | 89.05 | 80.78 | 8.27 | 9.29 |
| Nitromethane | 51.60 | 105.98 | 87.55 | 18.43 | 17.39 |
| Chlorodifluoromethane | 52.20 | 114.10 | 88.65 | 25.45 | 22.31 |
| Methylhydrazine | 52.30 | 134.93 | 88.83 | 46.10 | 34.16 |
| Methyl iodide | 52.80 | 82.75 | 89.75 | -7.00 | -8.46 |
| Chloroethylene | 53.90 | 89.45 | 91.76 | -2.31 | -2.58 |
| Dimethylether | 54.30 | 102.50 | 92.49 | 10.01 | 9.76 |
| Cyclopropane | 54.60 | 81.20 | 93.04 | -11.84 | -14.58 |
| 1,1-Difluoroethane | 54.70 | 118.40 | 93.22 | 25.18 | 21.26 |
| Carbon disulfide | 54.80 | 78.99 | 93.41 | -14.42 | -18.25 |
| Propylene | 55.90 | 102.00 | 95.42 | 6.58 | 6.45 |
| Dichloromethane | 56.60 | 100.00 | 96.70 | 3.30 | 3.30 |
| Ethylamine | 56.70 | 130.00 | 96.89 | 33.11 | 25.47 |
| Chlorotrifluoromethane | 57.10 | 121.60 | 97.62 | 23.98 | 19.72 |
| Cyanoethylene | 57.20 | 98.70 | 97.80 | 0.90 | 0.91 |
| Dimethylamine | 57.20 | 136.80 | 97.80 | 39.00 | 28.51 |
| Methyl formate | 57.30 | 119.10 | 97.98 | 21.12 | 17.73 |
| Bromoethylene | 58.10 | 107.50 | 99.45 | 8.05 | 7.49 |
| Chloroethane | 59.10 | 107.80 | 101.28 | 6.52 | 6.05 |
| Tetrafluoroethene | 59.30 | 113.00 | 101.64 | 11.36 | 10.05 |
| 1,1,1-Trifluoroethane | 59.60 | 109.60 | 102.19 | 7.41 | 6.76 |
| Methylnitrate | 59.60 | 157.19 | 102.19 | 55.00 | 34.99 |
| N-Methylformamide | 59.70 | 123.80 | 102.38 | 21.42 | 17.31 |
| Isoxazole | 60.60 | 108.10 | 104.02 | 4.08 | 3.77 |
| Trifluoroacetonitrile | 60.80 | 118.00 | 104.39 | 13.61 | 11.53 |
| Bromotrifluoromethane | 61.30 | 163.60 | 105.30 | 58.30 | 35.63 |
| Dichlorofluoromethane | 61.50 | 112.60 | 105.67 | 6.93 | 6.15 |
| Acetylchloride | 61.70 | 117.20 | 106.04 | 11.16 | 9.53 |
| Oxetane | 61.90 | 99.60 | 106.40 | -6.80 | -6.83 |
| Propanenitrile | 62.90 | 119.30 | 108.23 | 11.07 | 9.28 |
| 2-Oxetanone | 63.20 | 122.10 | 108.78 | 13.32 | 10.91 |
| Bromoethane | 63.30 | 105.80 | 108.96 | -3.16 | -2.99 |
| Ethanethiol | 63.40 | 117.90 | 109.15 | 8.75 | 7.42 |
| Dimethyl sulfide | 63.70 | 118.10 | 109.70 | 8.40 | 7.12 |
| Methyloxiran | 63.80 | 120.40 | 109.88 | 10.52 | 8.74 |
| Propanal | 64.50 | 134.70 | 111.16 | 23.54 | 17.48 |
| Propanone | 64.60 | 124.70 | 111.34 | 13.36 | 10.71 |
| Dibromomethane | 64.90 | 105.40 | 111.89 | -6.49 | -6.16 |
| Furan | 65.00 | 114.56 | 112.08 | 2.48 | 2.17 |
| Methylphosphonyl difluoride | 65.70 | 145.14 | 113.36 | 31.78 | 21.90 |
| Dichlorodifluoromethane | 66.40 | 126.80 | 114.64 | 12.16 | 9.59 |
| Cyclopropylamine | 66.60 | 147.11 | 115.00 | 32.11 | 21.82 |
| s-trans-1,3-Butadiene | 67.20 | 123.65 | 116.10 | 7.55 | 6.10 |
| 1,2-Butadiene | 67.40 | 123.00 | 116.47 | 6.53 | 5.31 |
| Pyrrole | 67.40 | 127.74 | 116.47 | 11.27 | 8.82 |
| Thiazole | 67.50 | 121.00 | 116.65 | 4.35 | 3.59 |
| cis-1,2-Dichloroethylene | 67.90 | 113.80 | 117.38 | -3.58 | -3.15 |
| trans-1,2-Dichloroethylene | 68.10 | 114.24 | 117.75 | -3.51 | -3.07 |
| Vinylidenechloride | 68.10 | 111.29 | 117.75 | -6.46 | -5.80 |
| 1-Butyne | 68.30 | 132.60 | 118.12 | 14.48 | 10.92 |
| 2-Butyne | 68.40 | 124.30 | 118.30 | 6.00 | 4.83 |
| Nitroethane | 68.40 | 134.22 | 118.30 | 15.92 | 11.86 |
| 1,2-Diaminoethane | 68.50 | 170.00 | 118.48 | 51.52 | 30.30 |
| Isothiazole | 68.60 | 119.70 | 118.67 | 1.03 | 0.86 |
| Chlorotrifluoroethylene | 68.60 | 122.60 | 118.67 | 3.93 | 3.21 |
| 1-Chloro-1,1-difluoroethane | 68.90 | 130.50 | 119.21 | 11.29 | 8.65 |
| Cyclobutane | 69.10 | 106.30 | 119.58 | -13.28 | -12.49 |
| 1,1-Dimethylhydrazine | 69.10 | 164.05 | 119.58 | 44.47 | 27.11 |
| 1,2-Dimethylhydrazine | 69.40 | 171.04 | 120.13 | 50.91 | 29.77 |
| Ethyl iodide | 69.50 | 115.10 | 120.31 | -5.21 | -4.53 |
| DMSO | 69.90 | 145.40 | 121.04 | 24.36 | 16.75 |
| Allyl chloride | 70.10 | 125.10 | 121.41 | 3.69 | 2.95 |
| 1,3-Dioxolane | 70.20 | 122.16 | 121.59 | 0.57 | 0.46 |
| Cyclopentadiene | 70.30 | 115.30 | 121.78 | -6.48 | -5.62 |
| 2,3-Dihydrofuran | 70.40 | 122.10 | 121.96 | 0.14 | 0.11 |
| Thietane | 70.50 | 112.50 | 122.14 | -9.64 | -8.57 |
| Trichloromethane | 70.70 | 114.32 | 122.51 | -8.19 | -7.16 |
| cis-2-Butene | 71.60 | 127.00 | 124.16 | 2.84 | 2.24 |
| Thiophene | 72.10 | 123.80 | 125.07 | -1.27 | -1.03 |
| Isobutylene | 72.20 | 121.30 | 125.25 | -3.95 | -3.26 |
| 1,3-Dioxolan-2-one | 72.20 | 133.90 | 125.25 | 8.65 | 6.46 |
| trans-2-Butene | 72.30 | 124.30 | 125.44 | -1.14 | -0.91 |
| 1-Butene | 72.60 | 128.96 | 125.99 | 2.97 | 2.31 |
| Cyclopropanecarbonitrile | 72.70 | 115.50 | 126.17 | -10.67 | -9.24 |
| 1,1-Dichloroethane | 73.20 | 126.27 | 127.08 | -0.81 | -0.64 |
| Ethylenedichloride | 73.30 | 128.81 | 127.27 | 1.54 | 1.20 |
| 2-Methylacrylonitrile | 73.40 | 126.30 | 127.45 | -1.15 | -0.91 |
| Isopropylamine | 73.40 | 164.00 | 127.45 | 36.55 | 22.29 |
| Trimethylamine | 73.50 | 132.60 | 127.63 | 4.97 | 3.75 |
| Methyl acetate | 74.00 | 140.56 | 128.55 | 12.01 | 8.55 |
| 1-Propylamine | 74.00 | 161.90 | 128.55 | 33.35 | 20.60 |
| Ethyl formate | 74.10 | 144.30 | 128.73 | 15.57 | 10.79 |
| Perfluoroethane | 74.30 | 131.40 | 129.10 | 2.30 | 1.75 |
| 2,5-Furandione | 74.70 | 120.10 | 129.83 | -9.73 | -8.10 |
| Crotonaldehyde | 75.50 | 148.60 | 131.29 | 17.31 | 11.65 |
| Fluorotrichloromethane | 75.60 | 121.55 | 131.48 | -9.93 | -8.17 |
| Methylphosphonyl chlorofluoride | 75.60 | 153.17 | 131.48 | 21.69 | 14.16 |
| 1-Chloropropane | 75.80 | 132.20 | 131.84 | 0.36 | 0.27 |
| Ethylnitrate | 76.40 | 170.30 | 132.94 | 37.36 | 21.94 |
| N,N-Dimethylformamide | 76.50 | 148.36 | 133.12 | 15.24 | 10.27 |
| 1-Methylimidazole | 77.00 | 147.20 | 134.04 | 13.16 | 8.94 |
| Diiodomethane | 77.30 | 133.81 | 134.59 | -0.78 | -0.58 |
| Cyclopentene | 77.30 | 122.38 | 134.59 | -12.21 | -9.98 |
| Tetrahydrofuran | 77.40 | 123.90 | 134.77 | -10.87 | -8.77 |
| 1-Bromo-2-chloroethane | 77.50 | 130.10 | 134.95 | -4.85 | -3.73 |
| Epichlorohydrin | 78.00 | 131.60 | 135.87 | -4.27 | -3.24 |
| Butane | 78.20 | 140.90 | 136.24 | 4.66 | 3.31 |
| Propanoyl chloride | 78.20 | 147.30 | 136.24 | 11.06 | 7.51 |
| 1-Methyl-1H-pyrazole | 78.20 | 173.28 | 136.24 | 37.04 | 21.38 |
| Pyridine | 78.80 | 133.00 | 137.33 | -4.33 | -3.26 |
| Methylenecyclobutane | 78.90 | 133.60 | 137.52 | -3.92 | -2.93 |
| gamma-Butyrolactone | 79.30 | 141.40 | 138.25 | 3.15 | 2.23 |
| Isobutyronitrile | 79.50 | 156.10 | 138.61 | 17.49 | 11.20 |
| Butyronitrile | 79.60 | 134.20 | 138.80 | -4.60 | -3.43 |
| 2-Bromopropane | 79.90 | 135.60 | 139.35 | -3.75 | -2.76 |
| 1-Bromopropane | 80.00 | 134.60 | 139.53 | -4.93 | -3.66 |
| 2-Propanethiol | 80.00 | 145.35 | 139.53 | 5.82 | 4.00 |
| 1-Propanethiol | 80.10 | 144.56 | 139.71 | 4.85 | 3.35 |
| Pyrrolidine | 80.10 | 156.57 | 139.71 | 16.86 | 10.77 |
| Ethyl methyl sulfide | 80.50 | 144.60 | 140.45 | 4.15 | 2.87 |
| Ethylepoxide | 80.50 | 147.00 | 140.45 | 6.55 | 4.46 |
| 2-Methylfuran | 80.70 | 143.72 | 140.81 | 2.91 | 2.02 |
| Dimethoxymethane | 80.70 | 163.60 | 140.81 | 22.79 | 13.93 |
| Succinonitrile | 80.80 | 145.60 | 140.99 | 4.61 | 3.16 |
| Butanal | 81.20 | 163.70 | 141.73 | 21.97 | 13.42 |
| Spiro[2.2]pentane | 81.30 | 134.52 | 141.91 | -7.39 | -5.49 |
| 1-Methylpyrrole | 81.30 | 150.06 | 141.91 | 8.15 | 5.43 |
| 2-Butanone | 81.30 | 158.40 | 141.91 | 16.49 | 10.41 |
| 2-Pyrrolidone | 81.30 | 169.50 | 141.91 | 27.59 | 16.28 |
| 1,2-Dibromoethane | 81.60 | 136.00 | 142.46 | -6.46 | -4.75 |
| 2-Methoxy-1-propene | 81.90 | 162.30 | 143.01 | 19.29 | 11.89 |
| 2-Cyanopropionaldehyde | 82.00 | 169.50 | 143.19 | 26.31 | 15.52 |
| beta-Cyanopropionaldehyde | 82.10 | 195.80 | 143.37 | 52.43 | 26.78 |
| 1-Cyanobicyclobutane | 82.20 | 131.80 | 143.56 | -11.76 | -8.92 |
| Trichloroethylene | 82.20 | 128.90 | 143.56 | -14.66 | -11.37 |
| Dimethyl disulfide | 82.60 | 146.11 | 144.29 | 1.82 | 1.25 |
| Isoprene | 82.80 | 151.08 | 144.65 | 6.43 | 4.25 |
| Tribromomethane | 83.20 | 135.10 | 145.39 | -10.29 | -7.61 |
| Furfural | 83.20 | 162.80 | 145.39 | 17.41 | 10.70 |
| cis-1,3-Pentadiene | 83.30 | 146.57 | 145.57 | 1.00 | 0.68 |
| Benzene | 83.40 | 136.80 | 145.75 | -8.95 | -6.54 |
| Dimethyl carbonate | 83.50 | 157.90 | 145.94 | 11.96 | 7.58 |
| Refrigerant 115 | 83.50 | 114.20 | 145.94 | -31.74 | -27.79 |
| 1,4-Pentadiene | 83.60 | 146.82 | 146.12 | 0.70 | 0.48 |
| trans-1,3-Pentadiene | 83.70 | 149.33 | 146.30 | 3.03 | 2.03 |
| 3-Methyl-1,2-butadiene | 83.70 | 152.42 | 146.30 | 6.12 | 4.01 |
| 2,3-Pentadiene | 83.80 | 152.34 | 146.49 | 5.85 | 3.84 |
| 1,2-Pentadiene | 84.10 | 150.83 | 147.03 | 3.80 | 2.52 |
| Methyl acrylate | 84.30 | 158.80 | 147.40 | 11.40 | 7.18 |
| Cyclopentane | 84.60 | 128.80 | 147.95 | -19.15 | -14.87 |
| Vinyl acetate | 84.70 | 169.50 | 148.13 | 21.37 | 12.61 |
| 2-Nitropropane | 84.70 | 170.30 | 148.13 | 22.17 | 13.02 |
| 1-Nitropropane | 84.80 | 160.40 | 148.32 | 12.08 | 7.53 |
| Tetrachloromethane | 84.80 | 131.80 | 148.32 | -16.52 | -12.53 |
| Propylenediamine | 85.10 | 205.64 | 148.86 | 56.78 | 27.61 |
| 1,3-Diaminopropane | 85.20 | 204.40 | 149.05 | 55.35 | 27.08 |
| 2-Methylthiazole | 85.30 | 150.70 | 149.23 | 1.47 | 0.98 |
| Trimethylhydrazine | 85.70 | 185.80 | 149.96 | 35.84 | 19.29 |
| 2-Iodopropane | 86.00 | 137.30 | 150.51 | -13.21 | -9.62 |
| 1,4-Dioxane | 86.20 | 150.65 | 150.88 | -0.23 | -0.15 |
| 1-Iodopropane | 86.20 | 136.20 | 150.88 | -14.68 | -10.78 |
| 1,3-Dioxane | 86.30 | 143.90 | 151.06 | -7.16 | -4.98 |
| Thiolane | 86.40 | 140.16 | 151.24 | -11.08 | -7.91 |
| Cyclopentanone | 86.50 | 154.50 | 151.43 | 3.07 | 1.99 |
| 2-Chlorothiophene | 86.50 | 139.43 | 151.43 | -12.00 | -8.60 |
| 1,1,2-Trichloroethane | 87.20 | 150.90 | 152.71 | -1.81 | -1.20 |
| Cyclobutanecarbonitrile | 87.20 | 146.00 | 152.71 | -6.71 | -4.59 |
| 1,1,1-Trichloroethane | 87.30 | 144.40 | 152.89 | -8.49 | -5.88 |
| Ethylurea | 87.50 | 151.50 | 153.26 | -1.76 | -1.16 |
| Isopropyl methyl ether | 87.50 | 161.90 | 153.26 | 8.64 | 5.34 |
| Ethyl ether | 87.80 | 172.50 | 153.81 | 18.69 | 10.84 |
| Trimethylethylene | 87.90 | 152.80 | 153.99 | -1.19 | -0.78 |
| Methylpropylether | 87.90 | 165.40 | 153.99 | 11.41 | 6.90 |
| Fluorobenzene | 88.30 | 146.29 | 154.72 | -8.43 | -5.76 |
| Isopentene | 88.50 | 157.30 | 155.09 | 2.21 | 1.41 |
| Propylenecarbonate | 88.50 | 167.40 | 155.09 | 12.31 | 7.36 |
| cis-2-Pentene | 88.70 | 151.80 | 155.45 | -3.65 | -2.41 |
| trans-2-Pentene | 88.90 | 157.00 | 155.82 | 1.18 | 0.75 |
| Morpholine | 88.90 | 164.80 | 155.82 | 8.98 | 5.45 |
| 1,3-Cyclohexadiene | 88.90 | 141.30 | 155.82 | -14.52 | -10.28 |
| Bromotrichloromethane | 89.00 | 148.50 | 156.00 | -7.50 | -5.05 |
| 3-Methyl-1-butene | 89.20 | 156.10 | 156.37 | -0.27 | -0.17 |
| 3-Methylthiophene | 89.20 | 150.62 | 156.37 | -5.75 | -3.82 |
| 2-Methylthiophene | 89.20 | 149.83 | 156.37 | -6.54 | -4.36 |
| 1-Pentene | 89.30 | 155.30 | 156.55 | -1.25 | -0.81 |
| 2,5-Norbornadiene | 89.50 | 161.20 | 156.92 | 4.28 | 2.66 |
| 1,1-Dichloropropane | 89.70 | 152.82 | 157.28 | -4.46 | -2.92 |
| 1,2-Dichloropropane | 89.80 | 156.81 | 157.47 | -0.66 | -0.42 |
| 2,2-Dichloropropane | 89.80 | 155.65 | 157.47 | -1.82 | -1.17 |
| Chloral | 89.80 | 151.50 | 157.47 | -5.97 | -3.94 |
| Methyl propanoate | 89.80 | 172.74 | 157.47 | 15.27 | 8.84 |
| 2-Methylpropylamine | 89.90 | 183.20 | 157.65 | 25.55 | 13.95 |
| t-Butylamine | 89.90 | 191.71 | 157.65 | 34.06 | 17.77 |
| 1,3-Dichloropropane | 90.00 | 157.00 | 157.83 | -0.83 | -0.53 |
| 1,4-Cyclohexadiene | 90.00 | 142.20 | 157.83 | -15.63 | -10.99 |
| 3-Chloro-1,1,1-trifluoropropane | 90.40 | 171.08 | 158.56 | 12.52 | 7.32 |
| 2-Bromothiophene | 90.40 | 144.00 | 158.56 | -14.56 | -10.11 |
| Diethylamine | 90.50 | 169.20 | 158.75 | 10.45 | 6.18 |
| Ethyl acetate | 90.60 | 169.06 | 158.93 | 10.13 | 5.99 |
| 1-Butylamine | 90.60 | 179.20 | 158.93 | 20.27 | 11.31 |
| n-Propylformate | 90.90 | 171.50 | 159.48 | 12.02 | 7.01 |
| 1-Bromo-2-chloro-1,1,2-trifluoroethane | 92.00 | 160.30 | 161.49 | -1.19 | -0.74 |
| 2-Bromo-2-chloro-1,1,1-trifluoroethane | 92.00 | 156.60 | 161.49 | -4.89 | -3.12 |
| 2-Chloro-2-methylpropane | 92.20 | 162.00 | 161.86 | 0.14 | 0.09 |
| 2-Chlorobutane | 92.20 | 160.90 | 161.86 | -0.96 | -0.60 |
| Isobutyl chloride | 92.20 | 158.60 | 161.86 | -3.26 | -2.06 |
| N-Methylpropionamide | 92.30 | 179.00 | 162.04 | 16.96 | 9.47 |
| Ethylacetamide | 92.30 | 179.90 | 162.04 | 17.86 | 9.93 |
| Isopropylnitrate | 92.40 | 191.10 | 162.23 | 28.87 | 15.11 |
| 1-Chlorobutane | 92.50 | 159.64 | 162.41 | -2.77 | -1.73 |
| Cryofluorane | 92.80 | 164.00 | 162.96 | 1.04 | 0.64 |
| Perfluoroacetone | 92.90 | 181.20 | 163.14 | 18.06 | 9.97 |
| 1,3-Difluorobenzene | 93.20 | 159.11 | 163.69 | -4.58 | -2.88 |
| 1,4-Difluorobenzene | 93.20 | 159.10 | 163.69 | -4.59 | -2.88 |
| 1,2-Difluorobenzene | 93.20 | 159.03 | 163.69 | -4.66 | -2.93 |
| Dimethylacetamide | 93.50 | 175.60 | 164.24 | 11.36 | 6.47 |
| Tetrahydropyran | 93.50 | 149.60 | 164.24 | -14.64 | -9.79 |
| 3-Methylcyclopentene | 93.90 | 152.30 | 164.97 | -12.67 | -8.32 |
| 2-Methyltetrahydrofuran | 94.10 | 156.89 | 165.34 | -8.45 | -5.38 |
| 1-Methylcyclopentene | 94.20 | 153.10 | 165.52 | -12.42 | -8.11 |
| Isopentane | 94.50 | 164.85 | 166.07 | -1.22 | -0.74 |
| Isobutyroylchloride | 94.50 | 131.80 | 166.07 | -34.27 | -26.00 |
| Butanoyl chloride | 94.70 | 170.70 | 166.43 | 4.27 | 2.50 |
| Aniline | 94.70 | 178.70 | 166.43 | 12.27 | 6.86 |
| 2,2-Dimethylpropane | 94.70 | 153.10 | 166.43 | -13.33 | -8.71 |
| Pentane | 94.90 | 167.19 | 166.80 | 0.39 | 0.23 |
| 1-Ethylpyrazole | 94.90 | 189.50 | 166.80 | 22.70 | 11.98 |
| Cyclohexene | 95.00 | 152.90 | 166.98 | -14.08 | -9.21 |
| 2-Picoline | 95.20 | 158.41 | 167.35 | -8.94 | -5.64 |
| 3-Methylenecyclobutanenitrile | 95.20 | 190.80 | 167.35 | 23.45 | 12.29 |
| 4-Methylpyridine | 95.30 | 158.99 | 167.53 | -8.54 | -5.37 |
| 3-Methylpyridine | 95.30 | 158.69 | 167.53 | -8.84 | -5.57 |
| Quadricyclane | 95.40 | 157.60 | 167.72 | -10.12 | -6.42 |
| Pivalonitrile | 95.90 | 179.37 | 168.63 | 10.74 | 5.99 |
| 1-Ethylimidazole | 96.00 | 186.82 | 168.81 | 18.01 | 9.64 |
| Tetrahydro-2H-pyran-2-one | 96.10 | 170.70 | 169.00 | 1.70 | 1.00 |
| Methyltrichlorosilane | 96.10 | 163.10 | 169.00 | -5.90 | -3.62 |
| Piperidine | 96.10 | 179.86 | 169.00 | 10.86 | 6.04 |
| Tetrachloroethylene | 96.20 | 157.70 | 169.18 | -11.48 | -7.28 |
| Cyclopentylamine | 96.20 | 181.21 | 169.18 | 12.03 | 6.64 |
| Isobutyl bromide | 96.30 | 163.70 | 169.36 | -5.66 | -3.46 |
| 2-Methyl-1-propanethiol | 96.40 | 171.88 | 169.55 | 2.33 | 1.36 |
| 2-Bromo-2-methylpropane | 96.40 | 165.70 | 169.55 | -3.85 | -2.32 |
| t-Butylmercaptan | 96.40 | 174.90 | 169.55 | 5.35 | 3.06 |
| 2-Butanethiol | 96.50 | 171.20 | 169.73 | 1.47 | 0.86 |
| 3-Methyl-2-butanone | 96.50 | 180.00 | 169.73 | 10.27 | 5.71 |
| Isopropyl methyl sulfide | 96.60 | 172.38 | 169.91 | 2.47 | 1.43 |
| 3-Methylpyrrolidine | 96.60 | 188.28 | 169.91 | 18.37 | 9.76 |
| 1-Cyanobicyclo[2.1.0]pentane | 96.70 | 164.00 | 170.10 | -6.10 | -3.72 |
| 1-Bromobutane | 96.70 | 162.20 | 170.10 | -7.90 | -4.87 |
| 1-Butanethiol | 96.80 | 172.30 | 170.28 | 2.02 | 1.17 |
| Diethylketone | 96.80 | 190.90 | 170.28 | 20.62 | 10.80 |
| N-Methylpyrrolidine | 96.90 | 167.75 | 170.46 | -2.72 | -1.62 |
| 1,1-Difluoro-1,2,2-trichloroethane | 97.00 | 167.04 | 170.64 | -3.60 | -2.16 |
| Methyl-n-propylketone | 97.00 | 184.10 | 170.64 | 13.46 | 7.31 |
| 2,4-Dimethylpyrrole | 97.00 | 192.19 | 170.64 | 21.55 | 11.21 |
| Dimethylmalononitrile | 97.10 | 179.50 | 170.83 | 8.67 | 4.83 |
| Diethyl sulphide | 97.20 | 171.42 | 171.01 | 0.41 | 0.24 |
| Methyl propyl sulfide | 97.20 | 171.63 | 171.01 | 0.62 | 0.36 |
| Pivalaldehyde | 97.20 | 185.58 | 171.01 | 14.57 | 7.85 |
| Glyme | 97.40 | 191.14 | 171.38 | 19.76 | 10.34 |
| Pentanedinitrile | 97.50 | 183.30 | 171.56 | 11.74 | 6.41 |
| n-Valeraldehyde | 97.60 | 174.39 | 171.74 | 2.65 | 1.52 |
| Chlorobenzene | 97.60 | 153.78 | 171.74 | -17.96 | -11.68 |
| 1,2-Dibromopropane | 98.10 | 172.80 | 172.66 | 0.14 | 0.08 |
| 1,3-Dibromopropane | 98.30 | 163.70 | 173.02 | -9.32 | -5.70 |
| 2-Piperidone | 98.30 | 208.40 | 173.02 | 35.38 | 16.98 |
| Chlorocyclopentane | 98.70 | 152.40 | 173.76 | -21.36 | -14.01 |
| 2,5-Dimethylpyrrole | 98.80 | 195.30 | 173.94 | 21.36 | 10.94 |
| N-Methyl-2-pyrrolidone | 98.90 | 166.26 | 174.12 | -7.86 | -4.73 |
| Dichlorodimethylsilane | 99.20 | 171.50 | 174.67 | -3.17 | -1.85 |
| 4-Fluoroaniline | 99.70 | 195.20 | 175.59 | 19.61 | 10.05 |
| 3-Chloro-1,1,1,3,3-pentafluoropropane | 99.70 | 196.60 | 175.59 | 21.01 | 10.69 |
| 1-Amino-3-methoxypropane | 99.70 | 225.52 | 175.59 | 49.93 | 22.14 |
| Methylbenzene | 100.00 | 158.70 | 176.14 | -17.44 | -10.99 |
| Perfluoropropane | 100.10 | 181.60 | 176.32 | 5.28 | 2.91 |
| Sulfolane | 100.20 | 180.00 | 176.50 | 3.50 | 1.94 |
| 1,5-Hexadiene | 100.40 | 133.10 | 176.87 | -43.77 | -32.88 |
| Cyclohexane | 100.50 | 158.10 | 177.05 | -18.95 | -11.99 |
| Benzonitrile | 100.90 | 165.20 | 177.78 | -12.58 | -7.62 |
| 1,2-Dibromotetrafluoroethane | 101.00 | 173.80 | 177.97 | -4.17 | -2.40 |
| Methylcyclopentane | 101.00 | 158.70 | 177.97 | -19.27 | -12.14 |
| Allyl acetate | 101.20 | 184.10 | 178.33 | 5.77 | 3.13 |
| Methyl methacrylate | 101.20 | 188.70 | 178.33 | 10.37 | 5.49 |
| 2-Methyl-1,2-propanediamine | 101.20 | 234.56 | 178.33 | 56.23 | 23.97 |
| 1,1,2,2-Tetrachloroethane | 101.30 | 168.00 | 178.51 | -10.51 | -6.26 |
| Carbon tetrabromide | 101.40 | 162.30 | 178.70 | -16.40 | -10.10 |
| Bromobenzene | 101.80 | 154.29 | 179.43 | -25.14 | -16.29 |
| Benzenethiol | 101.90 | 173.22 | 179.61 | -6.39 | -3.69 |
| Freon 113 | 101.90 | 172.80 | 179.61 | -6.81 | -3.94 |
| 1,1,1-Trichlorotrifluoroethane | 101.90 | 168.60 | 179.61 | -11.01 | -6.53 |
| Chlorotrimethylsilane | 102.20 | 187.50 | 180.16 | 7.34 | 3.91 |
| Cyclopentanecarbonitrile | 102.40 | 167.40 | 180.53 | -13.13 | -7.84 |
| Isobutyl iodide | 102.40 | 165.50 | 180.53 | -15.03 | -9.08 |
| 2-Iodobutane | 102.40 | 165.30 | 180.53 | -15.23 | -9.21 |
| Benzaldehyde | 102.60 | 177.90 | 180.89 | -2.99 | -1.68 |
| 1,3-Dioxepane | 102.60 | 167.00 | 180.89 | -13.89 | -8.32 |
| Thiane | 102.60 | 163.30 | 180.89 | -17.59 | -10.77 |
| 7-Oxabicyclo[4.1.0]heptane | 102.70 | 166.10 | 181.08 | -14.98 | -9.02 |
| Cyclopentanethiol | 102.70 | 165.20 | 181.08 | -15.88 | -9.61 |
| Cyclohexanone | 102.80 | 182.20 | 181.26 | 0.94 | 0.52 |
| 1-Iodobutane | 102.90 | 164.50 | 181.44 | -16.94 | -10.30 |
| 1,2,3,4-Tetrafluorobenzene | 103.00 | 189.90 | 181.63 | 8.27 | 4.36 |
| 1,2,3,5-Tetrafluorobenzene | 103.00 | 190.30 | 181.63 | 8.67 | 4.56 |
| 1,2,4,5-Tetrafluorobenzene | 103.00 | 192.20 | 181.63 | 10.57 | 5.50 |
| 3-Methylthiolane | 103.10 | 172.85 | 181.81 | -8.96 | -5.18 |
| 2-Methylthiolane | 103.10 | 172.00 | 181.81 | -9.81 | -5.70 |
| Tetramethylethene | 103.50 | 174.68 | 182.54 | -7.86 | -4.50 |
| MTBE | 103.80 | 187.50 | 183.09 | 4.41 | 2.35 |
| 1,2,3-Trichloropropane | 103.90 | 183.60 | 183.27 | 0.33 | 0.18 |
| 2-Fluorotoluene | 104.40 | 171.20 | 184.19 | -12.99 | -7.59 |
| n-Butyl methyl ether | 104.60 | 192.48 | 184.55 | 7.93 | 4.12 |
| 4-Fluorotoluene | 104.60 | 173.70 | 184.55 | -10.85 | -6.25 |
| Ethyl-n-propylether | 104.60 | 197.20 | 184.55 | 12.65 | 6.41 |
| cis-2-Hexene | 105.20 | 178.36 | 185.65 | -7.29 | -4.09 |
| Tetramethylsilane | 105.30 | 204.10 | 185.84 | 18.26 | 8.95 |
| 3,3-Dimethyl-1-butene | 105.50 | 188.30 | 186.20 | 2.10 | 1.11 |
| Isopropylacetate | 105.50 | 196.60 | 186.20 | 10.40 | 5.29 |
| 4-Methylpent-3-en-2-one | 105.50 | 212.50 | 186.20 | 26.30 | 12.38 |
| Methyl methylthiomethyl sulfoxide | 105.50 | 219.87 | 186.20 | 33.67 | 15.31 |
| N-Methylmorpholine | 105.70 | 199.26 | 186.57 | 12.69 | 6.37 |
| Diethylmethylamine | 105.90 | 200.00 | 186.93 | 13.07 | 6.53 |
| 1-Hexene | 106.00 | 183.30 | 187.12 | -3.82 | -2.08 |
| Trimethoxyborine | 106.30 | 189.90 | 187.67 | 2.23 | 1.18 |
| 1,2-Dichlorobutane | 106.30 | 195.60 | 187.67 | 7.93 | 4.06 |
| Ethynylbenzene | 106.40 | 180.10 | 187.85 | -7.75 | -4.30 |
| 2,5-Dimethylthiophene | 106.40 | 178.32 | 187.85 | -9.53 | -5.34 |
| 1,4-Dichlorobutane | 106.70 | 184.00 | 188.40 | -4.40 | -2.39 |
| Phenylhydrazine | 106.70 | 217.00 | 188.40 | 28.60 | 13.18 |
| Amylamine | 106.80 | 218.00 | 188.58 | 29.42 | 13.49 |
| Nitrobenzene | 106.90 | 186.60 | 188.76 | -2.16 | -1.16 |
| n-Propyl acetate | 106.90 | 194.10 | 188.76 | 5.34 | 2.75 |
| Butyric acid methylester | 107.00 | 198.16 | 188.95 | 9.21 | 4.65 |
| Ethyl propanoate | 107.00 | 204.06 | 188.95 | 15.11 | 7.41 |
| 1,3,5-Cycloheptatriene | 107.00 | 162.76 | 188.95 | -26.19 | -16.09 |
| Isobutyl formate | 107.40 | 214.20 | 189.68 | 24.52 | 11.45 |
| n-Butylformate | 107.60 | 200.25 | 190.05 | 10.20 | 5.10 |
| 2,2-Dinitropropane | 107.90 | 206.30 | 190.59 | 15.71 | 7.61 |
| Pentafluorobenzene | 107.90 | 210.79 | 190.59 | 20.20 | 9.58 |
| 3-Dimethylaminopropanenitrile | 107.90 | 212.50 | 190.59 | 21.91 | 10.31 |
| Perfluorocyclobutane | 108.00 | 209.60 | 190.78 | 18.82 | 8.98 |
| Iodobenzene | 108.00 | 158.40 | 190.78 | -32.38 | -20.44 |
| Ethyl cyanoacetate | 108.30 | 220.22 | 191.33 | 28.89 | 13.12 |
| Isopentyl chloride | 108.70 | 179.50 | 192.06 | -12.56 | -7.00 |
| N-Methylbutylamide | 108.70 | 207.00 | 192.06 | 14.94 | 7.22 |
| N,2-Dimethylpropanamide | 108.70 | 209.20 | 192.06 | 17.14 | 8.19 |
| Phenylisocyanate | 108.90 | 186.20 | 192.42 | -6.22 | -3.34 |
| Propylacetamide | 108.90 | 207.94 | 192.42 | 15.52 | 7.46 |
| Anisole | 109.10 | 199.00 | 192.79 | 6.21 | 3.12 |
| Methyl bicyclobutane-1-carboxylate | 109.20 | 192.90 | 192.97 | -0.07 | -0.04 |
| 1-Chloropentane | 109.20 | 187.70 | 192.97 | -5.27 | -2.81 |
| N,N-Dimethylpropionamide | 109.20 | 209.00 | 192.97 | 16.03 | 7.67 |
| Benzo[b]furan | 109.30 | 178.70 | 193.16 | -14.46 | -8.09 |
| N-(1-Methylethyl)ethanamide | 109.30 | 211.00 | 193.16 | 17.84 | 8.46 |
| 2-Chloroaniline | 109.70 | 199.00 | 193.89 | 5.11 | 2.57 |
| Cyclooctatetraene | 109.70 | 185.18 | 193.89 | -8.71 | -4.70 |
| 3-Chloroaniline | 109.80 | 198.70 | 194.07 | 4.63 | 2.33 |
| Norcarane | 110.10 | 187.90 | 194.62 | -6.72 | -3.58 |
| Styrene | 110.40 | 201.90 | 195.17 | 6.73 | 3.33 |
| Cycloheptene | 110.40 | 171.70 | 195.17 | -23.47 | -13.67 |
| Methylvinyldichlorosilane | 110.60 | 177.90 | 195.54 | -17.64 | -9.91 |
| 1,3-Diiodopropane | 110.70 | 169.20 | 195.72 | -26.52 | -15.67 |
| 2,5-Dimethyltetrahydrofuran | 110.80 | 188.13 | 195.90 | -7.77 | -4.13 |
| Neohexane | 111.00 | 189.67 | 196.27 | -6.60 | -3.48 |
| 2,3-Lutidine | 111.00 | 189.55 | 196.27 | -6.72 | -3.54 |
| 2,3-Dimethylbutane | 111.00 | 188.80 | 196.27 | -7.47 | -3.96 |
| 4-Methylcyclohexene | 111.00 | 180.42 | 196.27 | -15.85 | -8.78 |
| 1,1,2,2-Tetrachloro-1,2-difluoroethane | 111.00 | 178.70 | 196.27 | -17.57 | -9.83 |
| 3,4-Lutidine | 111.10 | 196.00 | 196.45 | -0.45 | -0.23 |
| 1-Ethylcyclopentene | 111.20 | 188.30 | 196.63 | -8.33 | -4.43 |
| Methylenecyclohexane | 111.20 | 177.40 | 196.63 | -19.23 | -10.84 |
| Ethylidenecyclopentane | 111.30 | 181.20 | 196.82 | -15.62 | -8.62 |
| 3-Methylpentane | 111.40 | 190.83 | 197.00 | -6.17 | -3.23 |
| Valeryl chloride | 111.40 | 187.90 | 197.00 | -9.10 | -4.84 |
| 2,6-Lutidine | 111.40 | 185.17 | 197.00 | -11.83 | -6.39 |
| Hexane | 111.50 | 197.66 | 197.18 | 0.48 | 0.24 |
| 2-Methylpentane | 111.50 | 193.96 | 197.18 | -3.22 | -1.66 |
| 2,4-Lutidine | 111.50 | 184.76 | 197.18 | -12.42 | -6.72 |
| 2,5-Lutidine | 111.50 | 184.70 | 197.18 | -12.48 | -6.76 |
| 3,5-Lutidine | 111.60 | 184.55 | 197.37 | -12.82 | -6.94 |
| 1,2-Dichlorobenzene | 111.70 | 170.90 | 197.55 | -26.65 | -15.59 |
| N-Methylaniline | 111.80 | 207.10 | 197.73 | 9.37 | 4.52 |
| Caprolactone | 111.90 | 196.80 | 197.92 | -1.12 | -0.57 |
| Benzylamine | 111.90 | 207.18 | 197.92 | 9.26 | 4.47 |
| 1,3-Dichlorobenzene | 111.90 | 170.90 | 197.92 | -27.02 | -15.81 |
| Hexamethylenimine | 112.00 | 205.00 | 198.10 | 6.90 | 3.37 |
| m-Toluidine | 112.20 | 216.70 | 198.46 | 18.24 | 8.42 |
| 1-Cyanobicyclo[3.1.0]hexane | 112.20 | 170.30 | 198.46 | -28.16 | -16.54 |
| o-Toluidine | 112.30 | 209.60 | 198.65 | 10.95 | 5.23 |
| t-Amyl mercaptan | 112.40 | 198.15 | 198.83 | -0.68 | -0.34 |
| 1,1-Dimethylsilacyclobutane | 112.40 | 197.53 | 198.83 | -1.30 | -0.66 |
| Pinacolone | 112.40 | 206.90 | 198.83 | 8.07 | 3.90 |
| 3-Methyl-2-butanethiol | 112.60 | 198.95 | 199.20 | -0.25 | -0.12 |
| 2-Methyl-1-butanethiol | 112.60 | 200.70 | 199.20 | 1.50 | 0.75 |
| Methyl t-butyl sulfide | 112.70 | 199.95 | 199.38 | 0.57 | 0.29 |
| 2-Methylpiperidine | 112.70 | 205.00 | 199.38 | 5.62 | 2.74 |
| Ethyltrichlorosilane | 112.70 | 192.60 | 199.38 | -6.78 | -3.52 |
| 4-Methylpiperidine | 112.70 | 209.00 | 199.38 | 9.62 | 4.60 |
| Benzothiazole | 112.80 | 189.54 | 199.56 | -10.02 | -5.29 |
| N-Methylpiperidine | 112.80 | 184.90 | 199.56 | -14.66 | -7.93 |
| Isopentyl bromide | 112.90 | 187.00 | 199.75 | -12.75 | -6.82 |
| Hexafluorobenzene | 112.90 | 221.60 | 199.75 | 21.85 | 9.86 |
| 3-Methyl-1-butanethiol | 113.00 | 200.33 | 199.93 | 0.40 | 0.20 |
| Indole | 113.00 | 192.00 | 199.93 | -7.93 | -4.13 |
| Ethylisopropylketone | 113.00 | 187.90 | 199.93 | -12.03 | -6.40 |
| (Chloromethyl)benzene | 113.00 | 182.40 | 199.93 | -17.53 | -9.61 |
| 2,2-Dimethoxypropane | 113.00 | 217.70 | 199.93 | 17.77 | 8.16 |
| Butylmethylketone | 113.30 | 213.40 | 200.48 | 12.92 | 6.06 |
| 1-Bromopentane | 113.40 | 219.70 | 200.66 | 19.04 | 8.67 |
| 1-Pentanethiol | 113.50 | 200.40 | 200.84 | -0.44 | -0.22 |
| Methyl isobutyl ketone | 113.60 | 211.90 | 201.03 | 10.87 | 5.13 |
| 2-Chlorotoluene | 113.70 | 166.80 | 201.21 | -34.41 | -20.63 |
| n-Butylmethylsulfide | 113.90 | 200.92 | 201.58 | -0.66 | -0.33 |
| Ethyl propyl sulfide | 113.90 | 198.41 | 201.58 | -3.17 | -1.60 |
| 3-Hexanone | 113.90 | 216.90 | 201.58 | 15.32 | 7.07 |
| Benzotrifluoride | 114.10 | 188.45 | 201.94 | -13.49 | -7.16 |
| 2,2’-Diaminodiethylamine | 114.10 | 254.00 | 201.94 | 52.06 | 20.50 |
| 2,5-Dioxaheptane | 114.20 | 224.70 | 202.12 | 22.58 | 10.05 |
| Caproic aldehyde | 114.30 | 210.40 | 202.31 | 8.09 | 3.85 |
| Methyl cyclobutanecarboxylate | 114.40 | 190.80 | 202.49 | -11.69 | -6.13 |
| 1,3-Dihydroisobenzofuran | 114.60 | 188.70 | 202.86 | -14.16 | -7.50 |
| 2,3-Dihydrobenzofuran | 114.80 | 188.64 | 203.22 | -14.58 | -7.73 |
| 1,4-Dibromobutane | 115.00 | 191.20 | 203.59 | -12.39 | -6.48 |
| Pentachloroethane | 115.30 | 196.20 | 204.14 | -7.94 | -4.05 |
| t-Butyl vinyl ether | 115.30 | 217.60 | 204.14 | 13.46 | 6.19 |
| Cycloheptane | 115.40 | 180.61 | 204.32 | -23.71 | -13.13 |
| Isobutyl vinyl ether | 115.80 | 231.80 | 205.05 | 26.75 | 11.54 |
| Bromo-2-chlorobenzene | 115.80 | 176.90 | 205.05 | -28.15 | -15.91 |
| Diethyl disulfide | 116.00 | 204.01 | 205.42 | -1.41 | -0.69 |
| 3-Bromochlorobenzene | 116.00 | 181.90 | 205.42 | -23.52 | -12.93 |
| Ethylbenzene | 116.10 | 185.78 | 205.60 | -19.82 | -10.67 |
| Butyl vinyl ether | 116.10 | 231.80 | 205.60 | 26.20 | 11.30 |
| Bicyclo[2.2.2]oct-2-ene | 116.10 | 156.73 | 205.60 | -48.87 | -31.18 |
| 1,2-Dimethylbenzene | 116.20 | 187.65 | 205.79 | -18.14 | -9.66 |
| 1,2,3-Tribromopropane | 116.20 | 166.50 | 205.79 | -39.29 | -23.59 |
| Bis(2-chloroethyl) ether | 116.30 | 220.90 | 205.97 | 14.93 | 6.76 |
| Ethyl cyclopropanecarboxylate | 116.60 | 213.00 | 206.52 | 6.48 | 3.04 |
| Benzoyl chloride | 116.60 | 187.00 | 206.52 | -19.52 | -10.44 |
| Trimethylvinylsilane | 116.80 | 198.00 | 206.88 | -8.88 | -4.49 |
| Indene | 116.80 | 186.94 | 206.88 | -19.94 | -10.67 |
| 1,4-Dimethylbenzene | 116.80 | 183.65 | 206.88 | -23.23 | -12.65 |
| 1,3-Dimethylbenzene | 116.80 | 183.18 | 206.88 | -23.70 | -12.94 |
| Methylcyclohexane | 117.00 | 184.96 | 207.25 | -22.29 | -12.05 |
| Diethylcarbonate | 117.10 | 218.55 | 207.43 | 11.12 | 5.09 |
| cis-1,2-Dimethylcyclopentane | 117.20 | 190.80 | 207.62 | -16.82 | -8.81 |
| 1,1,1-Trichloro-3,3,3-trifluoropropane | 117.30 | 199.91 | 207.80 | -7.89 | -3.95 |
| Ethylcyclopentane | 117.40 | 187.40 | 207.98 | -20.58 | -10.98 |
| trans-1,3-Dimethylcyclopentane | 117.60 | 190.80 | 208.35 | -17.55 | -9.20 |
| 1,1,1,3-Tetrachloropropane | 117.70 | 196.40 | 208.53 | -12.13 | -6.18 |
| 1,1-Dimethylcyclopentane | 117.70 | 187.40 | 208.53 | -21.13 | -11.28 |
| 1,1,2,2-Tetrabromoethane | 117.80 | 165.70 | 208.71 | -43.01 | -25.96 |
| 1,5-Cyclooctadiene | 117.90 | 208.10 | 208.90 | -0.80 | -0.38 |
| Suberone | 118.10 | 211.80 | 209.26 | 2.54 | 1.20 |
| Thioanisole | 118.10 | 206.02 | 209.26 | -3.24 | -1.57 |
| Benzyl mercaptan | 118.10 | 202.81 | 209.26 | -6.45 | -3.18 |
| Acetophenone | 118.30 | 205.50 | 209.63 | -4.13 | -2.01 |
| 3-(Dimethylamino)propylamine | 118.40 | 248.71 | 209.81 | 38.90 | 15.64 |
| Ethyl dichloroacetate | 118.50 | 209.60 | 209.99 | -0.39 | -0.19 |
| Cyclohexanecarbonitrile | 118.50 | 177.80 | 209.99 | -32.19 | -18.11 |
| Dimethyl malonate | 118.90 | 210.10 | 210.73 | -0.63 | -0.30 |
| Bromocyclohexane | 118.90 | 182.10 | 210.73 | -28.63 | -15.72 |
| Cyclopentyl methyl sulfide | 119.00 | 193.18 | 210.91 | -17.73 | -9.18 |
| Cyclohexanethiol | 119.00 | 192.63 | 210.91 | -18.28 | -9.49 |
| Isopentyliodide | 119.00 | 178.70 | 210.91 | -32.21 | -18.02 |
| Diisopropyl ether | 119.20 | 216.74 | 211.28 | 5.46 | 2.52 |
| Pentafluoroaniline | 119.20 | 231.00 | 211.28 | 19.72 | 8.54 |
| 1-Iodopentane | 119.60 | 193.60 | 212.01 | -18.41 | -9.51 |
| 2-Chloronitrobenzene | 119.80 | 212.30 | 212.37 | -0.07 | -0.04 |
| Methyl t-pentyl ether | 120.00 | 222.00 | 212.74 | 9.26 | 4.17 |
| 1,2-Dibromobenzene | 120.00 | 196.80 | 212.74 | -15.94 | -8.10 |
| 1,3-Dibromobenzene | 120.20 | 192.00 | 213.11 | -21.11 | -10.99 |
| Ethyl t-butyl ether | 120.50 | 218.00 | 213.66 | 4.34 | 1.99 |
| Tetramethylurea | 120.50 | 229.60 | 213.66 | 15.94 | 6.94 |
| 4-Methoxyaniline | 120.50 | 236.00 | 213.66 | 22.34 | 9.47 |
| 2-Isopropylthiophene | 120.90 | 211.38 | 214.39 | -3.01 | -1.42 |
| 4-Chloronitrobenzene | 120.90 | 250.20 | 214.39 | 35.81 | 14.31 |
| Ethyl 2-chloropropionate | 121.00 | 220.50 | 214.57 | 5.93 | 2.69 |
| Dipropyl ether | 121.10 | 221.45 | 214.75 | 6.70 | 3.02 |
| 2-Nitrotoluene | 121.20 | 202.10 | 214.94 | -12.84 | -6.35 |
| Pyrrolidinium nitrate | 121.40 | 228.00 | 215.30 | 12.70 | 5.57 |
| Indane | 121.60 | 190.25 | 215.67 | -25.42 | -13.36 |
| Triethylamine | 121.70 | 219.90 | 215.85 | 4.05 | 1.84 |
| N-Ethylmorpholine | 121.90 | 207.32 | 216.22 | -8.90 | -4.29 |
| t-Butylacetate | 122.00 | 231.00 | 216.40 | 14.60 | 6.32 |
| Chloropentafluorobenzene | 122.10 | 223.17 | 216.58 | 6.59 | 2.95 |
| Quinoline | 122.10 | 199.20 | 216.58 | -17.38 | -8.73 |
| Isoquinoline | 122.20 | 196.16 | 216.77 | -20.61 | -10.51 |
| 2,2,3-Trichlorobutyraldehyde | 122.30 | 241.80 | 216.95 | 24.85 | 10.28 |
| Ethyl acetoacetate | 122.30 | 241.80 | 216.95 | 24.85 | 10.28 |
| 1-Heptene | 122.70 | 211.79 | 217.68 | -5.89 | -2.78 |
| Isobutyl acetate | 123.00 | 233.80 | 218.23 | 15.57 | 6.66 |
| Methyl pivalate | 123.10 | 223.00 | 218.41 | 4.59 | 2.06 |
| 1-Chloro-3-isocyanatobenzene | 123.10 | 187.00 | 218.41 | -31.41 | -16.80 |
| 3,3-Bis(chloromethyl)oxetane | 123.30 | 218.80 | 218.78 | 0.02 | 0.01 |
| 3-Nitrotoluene | 123.30 | 202.50 | 218.78 | -16.28 | -8.04 |
| endo-2-Methylnorbornane | 123.30 | 184.30 | 218.78 | -34.48 | -18.71 |
| 4-Nitrotoluene | 123.30 | 172.40 | 218.78 | -46.38 | -26.90 |
| 1,5-Dichloropentane | 123.40 | 213.40 | 218.96 | -5.56 | -2.61 |
| Methyl valerate | 123.50 | 226.11 | 219.15 | 6.96 | 3.08 |
| n-Hexylamine | 123.50 | 246.00 | 219.15 | 26.85 | 10.92 |
| exo-2-Methylnorbornane | 123.50 | 185.80 | 219.15 | -33.35 | -17.95 |
| n-Butyl acetate | 123.60 | 225.10 | 219.33 | 5.77 | 2.56 |
| Nitrocyclohexane | 123.60 | 258.32 | 219.33 | 38.99 | 15.09 |
| Ethyl butanoate | 123.80 | 220.10 | 219.70 | 0.40 | 0.18 |
| Propyl propanoate | 123.80 | 226.65 | 219.70 | 6.95 | 3.07 |
| Dipropylamine | 123.90 | 252.70 | 219.88 | 32.82 | 12.99 |
| 2,3,4,5,6-Pentafluorotoluene | 124.00 | 225.80 | 220.06 | 5.74 | 2.54 |
| cis-Bicyclo[3.3.0]octane | 124.10 | 213.40 | 220.24 | -6.84 | -3.21 |
| trans-Bicyclo[3.3.0]octane | 124.10 | 180.30 | 220.24 | -39.94 | -22.15 |
| cis-Bicyclo[4,2,0]octane | 124.60 | 258.60 | 221.16 | 37.44 | 14.48 |
| 1,4-Dimethylpiperazine | 124.90 | 210.11 | 221.71 | -11.60 | -5.52 |
| Heptamethylenimine | 125.30 | 230.00 | 222.44 | 7.56 | 3.29 |
| cis- Cyclooctene | 125.50 | 207.80 | 222.81 | -15.01 | -7.22 |
| N-Methylvaleramide | 125.70 | 238.40 | 223.17 | 15.23 | 6.39 |
| Ethoxybenzene | 125.90 | 228.50 | 223.54 | 4.96 | 2.17 |
| 1-Chlorohexane | 125.90 | 216.20 | 223.54 | -7.34 | -3.39 |
| 1,2,4-Trichlorobenzene | 126.00 | 194.90 | 223.72 | -28.82 | -14.79 |
| Butylacetamide | 126.10 | 236.89 | 223.90 | 12.99 | 5.48 |
| 2,6-Xylidine | 126.10 | 238.86 | 223.90 | 14.96 | 6.26 |
| 2-Bromoiodobenzene | 126.10 | 179.90 | 223.90 | -44.00 | -24.46 |
| 3-Bromoiodobenzene | 126.40 | 183.10 | 224.45 | -41.35 | -22.59 |
| Triethylborane | 126.50 | 240.00 | 224.64 | 15.36 | 6.40 |
| alpha-Methylstyrene | 126.50 | 202.10 | 224.64 | -22.54 | -11.15 |
| Naphthalene | 126.50 | 196.06 | 224.64 | -28.58 | -14.58 |
| Ethylidenecyclohexane | 126.90 | 203.80 | 225.37 | -21.57 | -10.58 |
| Triptane | 127.20 | 213.51 | 225.92 | -12.41 | -5.81 |
| 2-Phenylethylamine | 127.30 | 239.24 | 226.10 | 13.14 | 5.49 |
| 3,3-Dimethylpentane | 127.40 | 214.80 | 226.28 | -11.48 | -5.35 |
| 2,2-Dimethylpentane | 127.70 | 221.12 | 226.83 | -5.71 | -2.58 |
| 3-Ethylpentane | 127.70 | 219.58 | 226.83 | -7.25 | -3.30 |
| 2,3-Dimethylpentane | 127.90 | 218.30 | 227.20 | -8.90 | -4.08 |
| 2,4-Dimethylpentane | 128.00 | 224.22 | 227.38 | -3.16 | -1.41 |
| Heptane | 128.20 | 225.33 | 227.75 | -2.42 | -1.07 |
| N,N-Dimethylaniline | 128.20 | 213.40 | 227.75 | -14.35 | -6.72 |
| 2-Methylhexane | 128.30 | 222.92 | 227.93 | -5.01 | -2.25 |
| N-Ethylaniline | 128.30 | 239.30 | 227.93 | 11.37 | 4.75 |
| Allylcyclopentane | 128.50 | 202.90 | 228.30 | -25.40 | -12.52 |
| Methyl benzoate | 128.70 | 221.30 | 228.66 | -7.36 | -3.33 |
| Dimethyl maleate | 128.80 | 263.20 | 228.85 | 34.35 | 13.05 |
| 1-Ethylpiperidine | 129.00 | 232.06 | 229.21 | 2.85 | 1.23 |
| Indole-3-carboxyaldehyde | 129.40 | 220.50 | 229.94 | -9.44 | -4.28 |
| cis-2,4,6-Trimethyl-1,3,5-trioxane | 129.40 | 257.30 | 229.94 | 27.36 | 10.63 |
| Diisopropyl sulfide | 129.50 | 232.00 | 230.13 | 1.87 | 0.81 |
| 2,4-Dimethyl-3-pentanone | 129.50 | 233.70 | 230.13 | 3.57 | 1.53 |
| Cyclohexyl formate | 129.60 | 219.70 | 230.31 | -10.61 | -4.83 |
| 3,4-Dimethylpentanal | 129.70 | 235.60 | 230.49 | 5.11 | 2.17 |
| 2-Methyl-3-hexanone | 129.70 | 241.50 | 230.49 | 11.01 | 4.56 |
| Cinnamaldehyde | 129.80 | 232.60 | 230.68 | 1.92 | 0.83 |
| 4-Heptanone | 129.80 | 246.30 | 230.68 | 15.62 | 6.34 |
| 1,4-Diisocyanatobutane | 130.10 | 225.06 | 231.23 | -6.17 | -2.74 |
| Hexyl bromide | 130.10 | 219.70 | 231.23 | -11.53 | -5.25 |
| 1-Hexanethiol | 130.20 | 230.71 | 231.41 | -0.70 | -0.30 |
| 3-Methylhexanal | 130.30 | 246.00 | 231.59 | 14.41 | 5.86 |
| 2-Heptanone | 130.40 | 243.60 | 231.78 | 11.82 | 4.85 |
| Dipropyl sulfide | 130.50 | 225.48 | 231.96 | -6.48 | -2.87 |
| Pentafluoronitrobenzene | 130.50 | 272.96 | 231.96 | 41.00 | 15.02 |
| 1,1-Diethoxyethane | 130.60 | 237.70 | 232.14 | 5.56 | 2.34 |
| 2,5-Dioxaoctane | 130.90 | 248.90 | 232.69 | 16.21 | 6.51 |
| n-Heptaldehyde | 131.00 | 230.78 | 232.87 | -2.09 | -0.91 |
| Diethyl cellosolve | 131.00 | 259.40 | 232.87 | 26.53 | 10.23 |
| Ethyl cyclobutanecarboxylate | 131.20 | 213.00 | 233.24 | -20.24 | -9.50 |
| 3,4-Dihydro-1H-2-benzopyran | 131.30 | 217.77 | 233.42 | -15.65 | -7.19 |
| 3-Oxabicyclo[3.2.2]nonane | 131.40 | 185.06 | 233.61 | -48.55 | -26.23 |
| Hemimellitene | 131.60 | 216.44 | 233.97 | -17.53 | -8.10 |
| 1,1-Dimethoxy-3-cyanopropane | 131.60 | 253.60 | 233.97 | 19.63 | 7.74 |
| 1,5-Dibromopentane | 131.70 | 220.84 | 234.15 | -13.31 | -6.03 |
| 3,4-Dihydro-2H-1-benzopyran | 131.70 | 214.00 | 234.15 | -20.15 | -9.42 |
| 1,2,4-Trimethylbenzene | 131.90 | 213.11 | 234.52 | -21.41 | -10.05 |
| Cumene | 132.20 | 215.40 | 235.07 | -19.67 | -9.13 |
| Isocumene | 132.20 | 214.72 | 235.07 | -20.35 | -9.48 |
| 1,2-Diiodobenzene | 132.20 | 190.00 | 235.07 | -45.07 | -23.72 |
| Dichlorodiethylsilane | 132.50 | 214.70 | 235.62 | -20.92 | -9.74 |
| Ethyl trichloroacetate | 132.60 | 230.10 | 235.80 | -5.70 | -2.48 |
| 1-Phenylpyrazole | 133.00 | 231.60 | 236.53 | -4.93 | -2.13 |
| 1,1-Dimethylcyclohexane | 133.00 | 209.24 | 236.53 | -27.29 | -13.04 |
| trans-1,3-Dimethylcyclohexane | 133.10 | 212.84 | 236.72 | -23.88 | -11.22 |
| cis-1,4-Dimethylcyclohexane | 133.10 | 212.09 | 236.72 | -24.63 | -11.61 |
| trans-1,2-Dimethylcyclohexane | 133.40 | 209.41 | 237.27 | -27.86 | -13.30 |
| 1,3,5-Trimethylbenzene | 133.50 | 207.85 | 237.45 | -29.60 | -14.24 |
| Ethylcyclohexane | 133.60 | 211.79 | 237.63 | -25.84 | -12.20 |
| Diisobutylene | 133.70 | 240.20 | 237.81 | 2.39 | 0.99 |
| Cyclooctane | 133.70 | 215.53 | 237.81 | -22.28 | -10.34 |
| trans-1,4-Dimethylcyclohexane | 133.80 | 210.25 | 238.00 | -27.75 | -13.20 |
| cis-1,3-Dimethylcyclohexane | 133.80 | 209.37 | 238.00 | -28.63 | -13.67 |
| 3-Azabicyclo[3.2.2]nonane | 133.90 | 238.90 | 238.18 | 0.72 | 0.30 |
| 1,2,3,4-Tetrahydroquinoline | 133.90 | 236.04 | 238.18 | -2.14 | -0.91 |
| 5,6,7,8-Tetrahydroquinoline | 134.00 | 217.41 | 238.36 | -20.95 | -9.64 |
| Propylcyclopentane | 134.10 | 216.27 | 238.55 | -22.28 | -10.30 |
| 1-Phenylimidazole | 134.40 | 249.10 | 239.10 | 10.00 | 4.02 |
| Tetramethyoxysilane | 134.60 | 240.50 | 239.46 | 1.04 | 0.43 |
| Ethyl 2,3-dichloropropionate | 134.60 | 248.90 | 239.46 | 9.44 | 3.79 |
| n-Butyl acrylate | 134.80 | 251.00 | 239.83 | 11.17 | 4.45 |
| Veratrole | 134.80 | 274.00 | 239.83 | 34.17 | 12.47 |
| 1-Octyne | 135.10 | 242.14 | 240.38 | 1.76 | 0.73 |
| 1-Phenyl-1-propanone | 135.10 | 243.20 | 240.38 | 2.82 | 1.16 |
| 4-Octyne | 135.20 | 233.64 | 240.56 | -6.92 | -2.96 |
| Ethyl N,N-dimethylglycinate | 135.30 | 244.20 | 240.74 | 3.46 | 1.42 |
| Dimethyl methylmalonate | 135.40 | 261.70 | 240.93 | 20.77 | 7.94 |
| Methyl trichlorothioacrylate | 135.60 | 244.90 | 241.29 | 3.61 | 1.47 |
| Ethanediol diacetate | 135.90 | 269.20 | 241.84 | 27.36 | 10.16 |
| Diethyl oxalate | 136.30 | 260.70 | 242.57 | 18.13 | 6.95 |
| 1-Iodohexane | 136.30 | 222.50 | 242.57 | -20.07 | -9.02 |
| 1-Piperazineethanamine | 136.30 | 284.90 | 242.57 | 42.33 | 14.86 |
| Ethyl t-amyl ether | 136.80 | 243.00 | 243.49 | -0.49 | -0.20 |
| 1,3,5-Trimethylhexahydro-s-triazine | 137.00 | 237.75 | 243.85 | -6.10 | -2.57 |
| 3-Nitrobenzotrifluoride | 137.30 | 223.80 | 244.40 | -20.60 | -9.21 |
| Perfluorotoluene | 137.90 | 262.30 | 245.50 | 16.80 | 6.40 |
| 8-Methylquinoline | 138.30 | 224.50 | 246.23 | -21.73 | -9.68 |
| Quinaldine | 138.40 | 224.72 | 246.42 | -21.70 | -9.66 |
| 1,2,3,4-Tetrahydronaphthalene | 138.50 | 217.44 | 246.60 | -29.16 | -13.41 |
| trans-2-Octene | 139.10 | 239.30 | 247.70 | -8.40 | -3.51 |
| Caprylene | 139.40 | 241.21 | 248.25 | -7.04 | -2.92 |
| Isoamyl acetate | 139.90 | 251.20 | 249.16 | 2.04 | 0.81 |
| cis-Hydrindan | 139.90 | 214.18 | 249.16 | -34.98 | -16.33 |
| Ethyl pivalate | 140.10 | 251.50 | 249.53 | 1.97 | 0.78 |
| Ethyl pentanoate | 140.10 | 256.81 | 249.53 | 7.28 | 2.84 |
| 1,6-Dichlorohexane | 140.10 | 239.57 | 249.53 | -9.96 | -4.16 |
| trans-Hydrindan | 140.10 | 209.70 | 249.53 | -39.83 | -18.99 |
| Sulcatone | 140.20 | 268.80 | 249.71 | 19.09 | 7.10 |
| Methyl hexanoate | 140.30 | 245.30 | 249.89 | -4.59 | -1.87 |
| Amyl acetate | 140.30 | 276.10 | 249.89 | 26.21 | 9.49 |
| Diethylene glycol dimethyl ether | 140.40 | 274.10 | 250.08 | 24.02 | 8.76 |
| 1-Chloronaphthalene | 140.50 | 212.60 | 250.26 | -37.66 | -17.71 |
| Trichlorotoluene | 141.10 | 218.67 | 251.36 | -32.69 | -14.95 |
| cis-Bicyclo[6.1.0]nonane | 142.00 | 235.10 | 253.01 | -17.91 | -7.62 |
| 1-Methylnaphthalene | 142.50 | 224.39 | 253.92 | -29.53 | -13.16 |
| 1-Chloroheptane | 142.60 | 245.40 | 254.10 | -8.70 | -3.55 |
| N,N-Dimethyl-o-toluidine | 142.80 | 280.30 | 254.47 | 25.83 | 9.22 |
| Isooctane | 142.90 | 242.49 | 254.65 | -12.16 | -5.02 |
| 2-Methylnaphthalene | 142.90 | 228.00 | 254.65 | -26.65 | -11.69 |
| 2,3,4-Trimethylpentane | 143.20 | 247.32 | 255.20 | -7.88 | -3.19 |
| 1-Ethyl-3-methylimidazolium bromide | 143.80 | 264.80 | 256.30 | 8.50 | 3.21 |
| 2,3,3-Trimethylpentane | 143.90 | 245.56 | 256.48 | -10.92 | -4.45 |
| Methyl o-toluate | 143.90 | 242.50 | 256.48 | -13.98 | -5.77 |
| 3-Phenylpropylamine | 144.00 | 265.59 | 256.67 | 8.92 | 3.36 |
| 3,3-Dimethylhexane | 144.00 | 246.60 | 256.67 | -10.07 | -4.08 |
| 2,4-Dinitrotoluene | 144.10 | 255.20 | 256.85 | -1.65 | -0.65 |
| 2,2,3-Trimethylpentane | 144.10 | 245.60 | 256.85 | -11.25 | -4.58 |
| 3-Methylheptane | 144.20 | 250.20 | 257.03 | -6.83 | -2.73 |
| Benzyl acetate | 144.20 | 250.20 | 257.03 | -6.83 | -2.73 |
| 2,5-Dimethylhexane | 144.20 | 249.20 | 257.03 | -7.83 | -3.14 |
| 2-Methylheptane | 144.30 | 252.00 | 257.22 | -5.22 | -2.07 |
| Cyclohexyl acetate | 144.50 | 245.40 | 257.58 | -12.18 | -4.96 |
| Allylcyclohexane | 144.50 | 233.50 | 257.58 | -24.08 | -10.31 |
| 4-Methylheptane | 144.80 | 251.08 | 258.13 | -7.05 | -2.81 |
| 2-(Phenoxymethyl)oxirane | 144.80 | 276.00 | 258.13 | 17.87 | 6.47 |
| Octane | 144.90 | 255.68 | 258.31 | -2.63 | -1.03 |
| Methyl m-toluate | 145.00 | 248.70 | 258.50 | -9.80 | -3.94 |
| Ethyl benzoate | 145.50 | 246.00 | 259.41 | -13.41 | -5.45 |
| 2,5,6-Trimethyl-2-cyclohexen-1-one | 146.10 | 258.40 | 260.51 | -2.11 | -0.82 |
| 2,4,6-Trimethyl-2-cyclohexen-1-one | 146.50 | 258.60 | 261.24 | -2.64 | -1.02 |
| Tetrahydrodicyclopentadiene | 146.50 | 236.50 | 261.24 | -24.74 | -10.46 |
| Heptyl bromide | 146.80 | 249.41 | 261.79 | -12.38 | -4.96 |
| 1-Heptanethiol | 146.90 | 259.32 | 261.97 | -2.65 | -1.02 |
| 2-Ethylhexanal | 147.00 | 267.80 | 262.16 | 5.64 | 2.11 |
| 2-Octanone | 147.10 | 273.26 | 262.34 | 10.92 | 4.00 |
| Prehnitene | 147.30 | 244.30 | 262.71 | -18.41 | -7.53 |
| 2,5-Dioxanonane | 147.60 | 282.00 | 263.26 | 18.74 | 6.65 |
| Caprylic aldehyde | 147.70 | 259.40 | 263.44 | -4.04 | -1.56 |
| Phthaloyl chloride | 148.20 | 248.50 | 264.35 | -15.85 | -6.38 |
| p-Cymene | 148.60 | 242.30 | 265.09 | -22.79 | -9.40 |
| t-Butylbenzene | 148.60 | 241.59 | 265.09 | -23.50 | -9.73 |
| Butylbenzene | 148.90 | 243.50 | 265.63 | -22.13 | -9.09 |
| Dipropyl disulfide | 149.40 | 262.46 | 266.55 | -4.09 | -1.56 |
| 1,2,4,5-Tetramethylbenzene | 149.50 | 220.10 | 266.73 | -46.63 | -21.19 |
| Propylcyclohexane | 150.30 | 242.04 | 268.20 | -26.16 | -10.81 |
| Dipropyl carbonate | 150.50 | 275.55 | 268.56 | 6.99 | 2.54 |
| 2,4-Toluene diisocyanate | 150.60 | 287.80 | 268.75 | 19.05 | 6.62 |
| Butylcyclopentane | 150.70 | 245.35 | 268.93 | -23.58 | -9.61 |
| 4-Ethylacetophenone | 151.00 | 260.70 | 269.48 | -8.78 | -3.37 |
| Phenyltrichlorosilane | 151.20 | 220.70 | 269.84 | -49.14 | -22.27 |
| n-Butylmethacrylate | 151.40 | 273.80 | 270.21 | 3.59 | 1.31 |
| Di-t-butyl ether | 151.40 | 276.10 | 270.21 | 5.89 | 2.13 |
| Methyl dimethylmalonate | 151.60 | 259.30 | 270.58 | -11.28 | -4.35 |
| trans-Decahydroquinoline | 151.60 | 254.81 | 270.58 | -15.77 | -6.19 |
| 3,3-Dinitroazetidinium nitrate | 151.70 | 272.95 | 270.76 | 2.19 | 0.80 |
| 3-Carene | 152.20 | 254.26 | 271.67 | -17.41 | -6.85 |
| 2-Acetoxyanisole | 152.30 | 278.50 | 271.86 | 6.64 | 2.39 |
| Diethyl malonate | 152.30 | 285.00 | 271.86 | 13.14 | 4.61 |
| 1-Iodoheptane | 153.00 | 251.80 | 273.14 | -21.34 | -8.47 |
| Biphenyl | 153.30 | 259.54 | 273.69 | -14.15 | -5.45 |
| Diisobutyl ether | 154.30 | 276.08 | 275.52 | 0.56 | 0.20 |
| 4,7-Dimethylindane | 154.30 | 241.50 | 275.52 | -34.02 | -14.09 |
| Camphor | 154.40 | 271.20 | 275.70 | -4.50 | -1.66 |
| 1,1-Dimethylindane | 154.40 | 249.40 | 275.70 | -26.30 | -10.55 |
| Di-n-butylether | 154.50 | 278.16 | 275.88 | 2.28 | 0.82 |
| 4,6-Dimethylindane | 154.50 | 240.90 | 275.88 | -34.98 | -14.52 |
| Dihydropinene | 155.30 | 251.26 | 277.35 | -26.09 | -10.38 |
| 1-Nonene | 156.10 | 270.36 | 278.81 | -8.45 | -3.13 |
| trans-Decalin | 156.10 | 229.17 | 278.81 | -49.64 | -21.66 |
| Bicyclopentyl | 156.50 | 238.90 | 279.54 | -40.64 | -17.01 |
| Isopentyl propionate | 156.70 | 285.20 | 279.91 | 5.29 | 1.85 |
| Fenchone | 156.90 | 269.80 | 280.28 | -10.48 | -3.88 |
| n-Octylamine | 156.90 | 302.00 | 280.28 | 21.72 | 7.19 |
| Butyl butanoate | 157.00 | 282.00 | 280.46 | 1.54 | 0.55 |
| Methylenanthate | 157.00 | 285.10 | 280.46 | 4.64 | 1.63 |
| n-Hexylacetate | 157.20 | 282.80 | 280.83 | 1.97 | 0.70 |
| Amyl propionate | 157.20 | 245.20 | 280.83 | -35.63 | -14.53 |
| Di-n-butylamine | 157.30 | 292.90 | 281.01 | 11.89 | 4.06 |
| Tetraethylmethane | 157.70 | 278.20 | 281.74 | -3.54 | -1.27 |
| 2,2,4,4-Tetramethylpentane | 158.60 | 266.30 | 283.39 | -17.09 | -6.42 |
| Butanoic anhydride | 158.80 | 283.70 | 283.75 | -0.05 | -0.02 |
| 2,7-Dimethylnaphthalene | 159.20 | 251.85 | 284.49 | -32.64 | -12.96 |
| 1-Chlorooctane | 159.30 | 274.70 | 284.67 | -9.97 | -3.63 |
| Pulegone | 159.70 | 274.90 | 285.40 | -10.50 | -3.82 |
| 2,2,3,3-Tetramethylpentane | 159.90 | 271.50 | 285.77 | -14.27 | -5.26 |
| 1-Methyl-4-isopropylcyclohexene | 160.20 | 257.80 | 286.32 | -28.52 | -11.06 |
| Di-t-butylsulfide | 160.70 | 281.74 | 287.23 | -5.49 | -1.95 |
| 1-Propyl-3-methylimidazolium bromide | 160.70 | 281.40 | 287.23 | -5.83 | -2.07 |
| 4-Methoxycarbonylhomocubane | 160.80 | 288.00 | 287.41 | 0.59 | 0.20 |
| N,N-Diethylaniline | 160.80 | 274.50 | 287.41 | -12.91 | -4.70 |
| N-(2-Trimethylsilylethyl)ethylenimine | 160.80 | 300.80 | 287.41 | 13.39 | 4.45 |
| Nonane | 161.50 | 284.43 | 288.70 | -4.27 | -1.50 |
| 2,6-Dimethylheptan-4-one | 161.90 | 297.30 | 289.43 | 7.87 | 2.65 |
| Ethyl cyclohexanecarboxylate | 162.40 | 271.50 | 290.34 | -18.84 | -6.94 |
| 5-Nonanone | 163.20 | 303.60 | 291.81 | 11.79 | 3.88 |
| 1,6-Hexamethylene diisocyanate | 163.50 | 294.00 | 292.36 | 1.64 | 0.56 |
| 1-Bromooctane | 163.50 | 272.16 | 292.36 | -20.20 | -7.42 |
| 1-Octanethiol | 163.60 | 300.40 | 292.54 | 7.86 | 2.62 |
| Di-n-butylsulfide | 163.90 | 284.34 | 293.09 | -8.75 | -3.08 |
| 2,4,4-Trimethyl-2-nitropentane | 164.10 | 232.48 | 293.45 | -60.97 | -26.23 |
| n-Nonanal | 164.30 | 290.40 | 293.82 | -3.42 | -1.18 |
| 1,2-Dipropoxyethane | 164.40 | 309.00 | 294.00 | 15.00 | 4.85 |
| 1,8-Cineole | 164.40 | 270.72 | 294.00 | -23.28 | -8.60 |
| 2,4,5-Trinitrotoluene | 164.60 | 311.70 | 294.37 | 17.33 | 5.56 |
| Neopentylbenzene | 165.20 | 271.90 | 295.47 | -23.57 | -8.67 |
| 1-Ethyl-3-methylimidazolium thiocyanate | 165.40 | 281.45 | 295.83 | -14.38 | -5.11 |
| t-Butylcyclohexane | 165.50 | 264.80 | 296.02 | -31.22 | -11.79 |
| 1-Ethyl-3-methylimidazolium acetate | 166.00 | 321.90 | 296.93 | 24.97 | 7.76 |
| N-Phenylpiperidine | 166.00 | 324.11 | 296.93 | 27.18 | 8.39 |
| 1-Methyltetrahydrothiophenium dicyanamide | 166.00 | 338.50 | 296.93 | 41.57 | 12.28 |
| Perfluoromethyldiethylamine | 166.10 | 337.46 | 297.12 | 40.34 | 11.96 |
| Hexamethyldisilane | 166.40 | 256.10 | 297.66 | -41.56 | -16.23 |
| Neral | 166.90 | 304.60 | 298.58 | 6.02 | 1.98 |
| Butylcyclohexane | 167.00 | 271.04 | 298.76 | -27.72 | -10.23 |
| 1-Phenyl-1-methylsilacyclobutane | 167.60 | 273.20 | 299.86 | -26.66 | -9.76 |
| Hexamethylphosphoramide | 167.80 | 321.30 | 300.23 | 21.07 | 6.56 |
| N-Cyclopentylpiperidine | 167.90 | 282.02 | 300.41 | -18.39 | -6.52 |
| Hexamethyldisiloxane | 168.30 | 309.09 | 301.14 | 7.95 | 2.57 |
| Diethyl succinate | 168.40 | 330.50 | 301.32 | 29.18 | 8.83 |
| Ethylene glycol dipropionate | 169.00 | 331.80 | 302.42 | 29.38 | 8.85 |
| 1-Ethyl-3-methylimidazolium tetrafluoroborate | 169.10 | 305.00 | 302.61 | 2.39 | 0.78 |
| 1,I-Dimethylazoxyethane | 169.90 | 316.50 | 304.07 | 12.43 | 3.93 |
| 1,4-Dibromo-2,3-dichlorohexafluorobutane | 170.40 | 298.50 | 304.99 | -6.49 | -2.17 |
| 2-Phenyltoluene | 170.50 | 275.77 | 305.17 | -29.40 | -10.66 |
| Diphenyl sulfide | 170.60 | 271.10 | 305.35 | -34.25 | -12.63 |
| Diphenylmethane | 170.60 | 266.10 | 305.35 | -39.25 | -14.75 |
| Benzophenone | 170.90 | 289.59 | 305.90 | -16.31 | -5.63 |
| Tetraethylsilane | 171.10 | 297.70 | 306.27 | -8.57 | -2.88 |
| 1-Butyl-3-methylimidazolium chloride | 171.10 | 317.00 | 306.27 | 10.73 | 3.39 |
| Dimethylphenylvinylsilane | 171.90 | 284.80 | 307.73 | -22.93 | -8.05 |
| Tripropylamine | 172.00 | 301.00 | 307.91 | -6.91 | -2.30 |
| Dimethyl phthalate | 172.30 | 303.10 | 308.46 | -5.36 | -1.77 |
| Benzyl methacrylate | 172.30 | 269.90 | 308.46 | -38.56 | -14.29 |
| 1-Decene | 172.80 | 300.83 | 309.38 | -8.55 | -2.84 |
| 1,3-Dimethylimidazolium methosulfate | 172.80 | 341.00 | 309.38 | 31.62 | 9.27 |
| Ethyl trans-cinnamate | 172.90 | 296.36 | 309.56 | -13.20 | -4.45 |
| Methyl octanoate | 173.40 | 314.23 | 310.48 | 3.75 | 1.19 |
| Pentyl butyrate | 173.90 | 311.70 | 311.39 | 0.31 | 0.10 |
| Diethyleneglycol diethyl ether | 174.00 | 347.50 | 311.57 | 35.93 | 10.34 |
| 5-Trimethylsilyl-2-norbornene | 174.60 | 290.40 | 312.67 | -22.27 | -7.67 |
| Benzophenoneimine | 175.70 | 333.48 | 314.69 | 18.79 | 5.64 |
| 1-Chlorononane | 176.00 | 296.44 | 315.23 | -18.79 | -6.34 |
| 1-Ethyl-3-methylimidazolium dicyanamide | 176.10 | 314.64 | 315.42 | -0.78 | -0.25 |
| 5-Methylnonane | 177.30 | 314.43 | 317.61 | -3.18 | -1.01 |
| N -(2-Trimethylsilylethyl)trimethylenimine | 177.40 | 304.60 | 317.80 | -13.20 | -4.33 |
| Ethyl hydrocinnamate | 177.50 | 286.60 | 317.98 | -31.38 | -10.95 |
| 4-Methylnonane | 177.60 | 317.36 | 318.16 | -0.80 | -0.25 |
| 3-Methylnonane | 177.60 | 308.99 | 318.16 | -9.17 | -2.97 |
| 1-Ethylferrocene | 177.60 | 335.30 | 318.16 | 17.14 | 5.11 |
| 1-Butyl-3-methylimidazolium bromide | 177.70 | 317.00 | 318.35 | -1.35 | -0.42 |
| 1-Ethyladamantane | 177.70 | 258.45 | 318.35 | -59.90 | -23.18 |
| 2-Methylnonane | 178.00 | 313.30 | 318.90 | -5.60 | -1.79 |
| Tolan | 178.00 | 297.50 | 318.90 | -21.40 | -7.19 |
| Decane | 178.20 | 316.32 | 319.26 | -2.94 | -0.93 |
| 1,3-Dimethyladamantane | 178.20 | 258.31 | 319.26 | -60.95 | -23.60 |
| cis,trans,trans-1,5,9-Cyclododecatriene | 178.40 | 287.76 | 319.63 | -31.87 | -11.07 |
| Cyclohexyl butanoate | 178.60 | 300.70 | 319.99 | -19.29 | -6.42 |
| 1-Ethyl-3-methylimidazolium methanesulfonate | 180.00 | 345.52 | 322.56 | 22.96 | 6.65 |
| Hexamethylbenzene | 180.00 | 370.70 | 322.56 | 48.14 | 12.99 |
| 1-Bromononane | 180.20 | 308.32 | 322.92 | -14.60 | -4.74 |
| 1,1-Diphenylethylene | 180.70 | 299.20 | 323.84 | -24.64 | -8.23 |
| trans-Stilbene | 180.80 | 343.10 | 324.02 | 19.08 | 5.56 |
| Di-t-butyldisulfide | 180.90 | 313.63 | 324.20 | -10.57 | -3.37 |
| Bis(trimethylsilyl)methane | 180.90 | 357.50 | 324.20 | 33.30 | 9.31 |
| Capraldehyde | 181.00 | 319.70 | 324.39 | -4.69 | -1.47 |
| 1-Ethyl-3-methylimidazolium trifluoroacetate | 181.00 | 316.00 | 324.39 | -8.39 | -2.65 |
| 1,2,3,4-Tetrahydrophenanthrene | 181.10 | 278.26 | 324.57 | -46.31 | -16.64 |
| N-Methyldiphenylamine | 181.60 | 301.20 | 325.48 | -24.28 | -8.06 |
| Diphenylmethylamine | 182.00 | 301.20 | 326.22 | -25.02 | -8.31 |
| Perfluoromethylcyclohexane | 182.80 | 353.10 | 327.68 | 25.42 | 7.20 |
| 2,5,8,11-Tetraoxododecane | 183.40 | 367.78 | 328.78 | 39.00 | 10.60 |
| Diphenylcarbodiimide | 183.70 | 304.60 | 329.33 | -24.73 | -8.12 |
| N-Cyclohexylpiperidine | 184.10 | 287.76 | 330.06 | -42.30 | -14.70 |
| Benzhydryl chloride | 184.20 | 290.40 | 330.24 | -39.84 | -13.72 |
| Tetraethylurea | 184.50 | 345.70 | 330.79 | 14.91 | 4.31 |
| 1-Ethyltetrahydrothiophenium dicyanamide | 184.90 | 335.38 | 331.52 | 3.86 | 1.15 |
| 1,1,4,7-Tetramethylindane | 185.10 | 302.50 | 331.89 | -29.39 | -9.72 |
| 2-Ethylbiphenyl | 185.20 | 302.73 | 332.07 | -29.34 | -9.69 |
| Benzyldimethylvinylsilane | 185.40 | 312.20 | 332.44 | -20.24 | -6.48 |
| 1,2-Diphenylethane | 186.10 | 320.10 | 333.72 | -13.62 | -4.26 |
| 1-Butyl-3-methylimidazolium nitrate | 186.20 | 353.50 | 333.90 | 19.60 | 5.54 |
| 3,3'-Bitolyl | 186.20 | 295.45 | 333.90 | -38.45 | -13.02 |
| 2,2'-Dimethylbiphenyl | 186.60 | 298.06 | 334.64 | -36.58 | -12.27 |
| 2-Methyldiphenylmethane | 186.60 | 296.58 | 334.64 | -38.06 | -12.83 |
| 1,1-Diphenylethane | 186.80 | 295.00 | 335.00 | -40.00 | -13.56 |
| Diisoamyl ether | 187.20 | 379.00 | 335.73 | 43.27 | 11.42 |
| 1-Propylpyridinium tetrafluoroborate | 187.40 | 363.00 | 336.10 | 26.90 | 7.41 |
| 2,3-Dihydro-1,1,4,6-tetramethyl-1H-indene | 187.50 | 299.60 | 336.28 | -36.68 | -12.24 |
| Bicyclohexyl | 188.70 | 283.00 | 338.48 | -55.48 | -19.60 |
| 3-Methylbenzophenone | 189.10 | 325.10 | 339.21 | -14.11 | -4.34 |
| 1-Ethyl-3-methylimidazolium methylsulfate | 189.20 | 341.00 | 339.39 | 1.61 | 0.47 |
| 1-Undecene | 189.50 | 329.95 | 339.94 | -9.99 | -3.03 |
| 1-Ethyl-3-methylimidazolium tricyanomethide | 189.90 | 358.70 | 340.68 | 18.02 | 5.03 |
| n-Octylacetate | 190.00 | 339.70 | 340.86 | -1.16 | -0.34 |
| Methyl nonanoate | 190.10 | 345.63 | 341.04 | 4.59 | 1.33 |
| n-Decylamine | 190.30 | 361.00 | 341.41 | 19.59 | 5.43 |
| Ethyl caprylate | 190.50 | 343.13 | 341.77 | 1.36 | 0.40 |
| 1-Ethyl-3-methylimidazolium hexafluorophosphate | 190.60 | 343.60 | 341.96 | 1.64 | 0.48 |
| 1-Butyl-3-methylimidazolium iodide | 190.80 | 314.00 | 342.32 | -28.32 | -9.02 |
| Tris(pentafluoroethyl)amine | 191.00 | 379.49 | 342.69 | 36.80 | 9.70 |
| 1-Benzyl-3-methylimidazolium chloride | 192.00 | 339.40 | 344.52 | -5.12 | -1.51 |
| 1-Ethylpyridinium hexafluorophosphate | 192.30 | 293.04 | 345.07 | -52.03 | -17.75 |
| delta-Undecanolactone | 193.60 | 342.70 | 347.45 | -4.75 | -1.39 |
| N-Benzylbenzaldehyde imine | 193.70 | 384.66 | 347.63 | 37.03 | 9.63 |
| 1-Propylferrocene | 194.50 | 319.50 | 349.09 | -29.59 | -9.26 |
| 2,4,6,8-Tetramethylcyclotetrasiloxane | 194.70 | 354.40 | 349.46 | 4.94 | 1.39 |
| 2-Methyldecane | 194.70 | 341.21 | 349.46 | -8.25 | -2.42 |
| Undecane | 194.90 | 345.05 | 349.83 | -4.78 | -1.38 |
| Cyclohexyl valerate | 195.10 | 323.00 | 350.19 | -27.19 | -8.42 |
| 1-Ethyl-3-methylimidazolium trifluoromethylsulfonate | 195.20 | 362.80 | 350.38 | 12.42 | 3.42 |
| Hexamethylcyclotrisiloxane | 195.90 | 360.00 | 351.66 | 8.34 | 2.32 |
| 1-Bromodecane | 196.90 | 334.40 | 353.49 | -19.09 | -5.71 |
| 1-Decanethiol | 197.00 | 350.41 | 353.67 | -3.26 | -0.93 |
| 6-Undecanone | 197.00 | 362.70 | 353.67 | 9.03 | 2.49 |
| Bis(piperidino)methane | 197.10 | 364.62 | 353.85 | 10.77 | 2.95 |
| Dibutyl acetal | 197.30 | 352.20 | 354.22 | -2.02 | -0.57 |
| Glyceryltriacetate | 197.30 | 384.70 | 354.22 | 30.48 | 7.92 |
| 1,2-Dibutoxyethane | 197.80 | 350.00 | 355.13 | -5.13 | -1.47 |
| 1-Butyl-3-methylimidazolium thiocyanate | 198.70 | 385.00 | 356.78 | 28.22 | 7.33 |
| 1-(1-Isocyanato-1-methylethyl-3-(1-methylethenyl)benzene | 200.20 | 382.00 | 359.53 | 22.47 | 5.88 |
| 1-Butyl-3-methylimidazolium acetate | 200.70 | 383.20 | 360.44 | 22.76 | 5.94 |
| 2-Ethylhexyl acrylate | 201.00 | 355.10 | 360.99 | -5.89 | -1.66 |
| Tetraethoxysilane | 201.60 | 364.40 | 362.09 | 2.31 | 0.63 |
| Ethylene glycol dibutyrate | 202.00 | 380.30 | 362.82 | 17.48 | 4.60 |
| 1-Butyl-3-methylimidazolium tetrafluoroborate | 202.30 | 363.00 | 363.37 | -0.37 | -0.10 |
| 4-Isopropylbiphenyl | 202.70 | 343.90 | 364.10 | -20.20 | -5.87 |
| beta-Ionone | 203.80 | 382.80 | 366.12 | 16.68 | 4.36 |
| Perfluoroheptane | 203.80 | 419.00 | 366.12 | 52.88 | 12.62 |
| 1-Butylpyridinium tetrafluoroborate | 204.00 | 383.80 | 366.48 | 17.32 | 4.51 |
| Diethyl phthalate | 204.20 | 366.10 | 366.85 | -0.75 | -0.20 |
| 1-Ethyl-3-methylimidazolium ethosulfate | 204.60 | 378.00 | 367.58 | 10.42 | 2.76 |
| 1-Methoxydecane | 204.80 | 370.80 | 367.95 | 2.85 | 0.77 |
| 1-Methylazepanium methosulfate | 205.30 | 332.08 | 368.86 | -36.78 | -11.08 |
| 1-Dodecene | 206.20 | 360.66 | 370.51 | -9.85 | -2.73 |
| Methyl decanoate | 207.10 | 372.03 | 372.16 | -0.13 | -0.03 |
| 1-Propyl-3-methylimidazolium hexafluorophosphate | 207.50 | 374.40 | 372.89 | 1.51 | 0.40 |
| Diethyl terephthalate | 207.60 | 380.70 | 373.07 | 7.63 | 2.00 |
| 2,2,4,6,6-Pentamethylheptane | 207.60 | 350.98 | 373.07 | -22.09 | -6.29 |
| 1-Butyl-3-methylimidazolium dicyanoamide | 208.00 | 365.00 | 373.80 | -8.80 | -2.41 |
| 1-Propylpyridinium hexafluorophosphate | 208.90 | 328.70 | 375.45 | -46.75 | -14.22 |
| 3-Nitro-3-(4-nitrophenyl)pentane | 209.30 | 409.79 | 376.18 | 33.61 | 8.20 |
| 1-Ethyl-3-methylimidazolium dimethylphosphate | 209.30 | 411.78 | 376.18 | 35.60 | 8.64 |
| Cyclam | 210.10 | 374.61 | 377.65 | -3.04 | -0.81 |
| Pseudoionone | 210.20 | 382.70 | 377.83 | 4.87 | 1.27 |
| 1-Hexyl-3-methylimidazolium bromide | 210.20 | 344.00 | 377.83 | -33.83 | -9.83 |
| 1,1'-Diethylferrocene | 210.40 | 348.20 | 378.20 | -30.00 | -8.61 |
| 6,10-Dimethyl-4,5,9-undecatrien-2-one | 211.30 | 382.70 | 379.84 | 2.86 | 0.75 |
| 1-Butylferrocene | 211.30 | 363.80 | 379.84 | -16.04 | -4.41 |
| 1-(3-Cyanopropyl)-pyridinium dicyanamide | 211.50 | 422.00 | 380.21 | 41.79 | 9.90 |
| Dodecane | 211.60 | 376.10 | 380.39 | -4.29 | -1.14 |
| 1-Isobutylferrocene | 212.10 | 346.90 | 381.31 | -34.41 | -9.92 |
| 1-Bromoundecane | 213.60 | 373.23 | 384.05 | -10.82 | -2.90 |
| 1,4-Di-t-butylbenzene | 213.60 | 347.84 | 384.05 | -36.21 | -10.41 |
| Tris-(carboethoxy)methane | 213.80 | 419.80 | 384.42 | 35.38 | 8.43 |
| 1-Butyl-3-methylimidazolium trifluoroacetate | 214.70 | 408.20 | 386.07 | 22.13 | 5.42 |
| Tridecanolactone | 215.50 | 398.30 | 387.53 | 10.77 | 2.70 |
| 1,3-Bis(trimethylsilyl)propane | 215.60 | 394.34 | 387.71 | 6.63 | 1.68 |
| 1-Butyl-2-methylimidazolium trifluoroacetate | 216.80 | 407.90 | 389.91 | 17.99 | 4.41 |
| Heptylcyclohexane | 217.00 | 363.20 | 390.28 | -27.08 | -7.45 |
| NHTPN | 217.80 | 380.14 | 391.74 | -11.60 | -3.05 |
| 1-Butyltetrahydrothiophenium dicyanamide | 218.20 | 395.19 | 392.47 | 2.72 | 0.69 |
| Octyl methacrylate | 218.20 | 386.00 | 392.47 | -6.47 | -1.68 |
| 4-t-Butylbiphenyl | 218.20 | 383.60 | 392.47 | -8.87 | -2.31 |
| 1-Butyl-2,3-dimethylimidazolium tetrafluoroborate | 218.70 | 416.01 | 393.39 | 22.62 | 5.44 |
| Nonyl acrylate | 218.70 | 417.90 | 393.39 | 24.51 | 5.87 |
| 1-Butyl-3-methylpyridinium tetrafluoroborate | 219.00 | 388.00 | 393.94 | -5.94 | -1.53 |
| 1-Butyl-4-methylpyridinium tetrafluoroborate | 219.00 | 414.00 | 393.94 | 20.06 | 4.85 |
| 1-Ethyl-3-methylpyridinium ethylsulfate | 221.30 | 389.00 | 398.15 | -9.15 | -2.35 |
| 1-Butyl-3-methylimidazolium methosulfate | 221.40 | 416.00 | 398.33 | 17.67 | 4.25 |
| Tributylamine | 222.50 | 391.83 | 400.34 | -8.51 | -2.17 |
| 1-Tridecene | 222.90 | 391.80 | 401.07 | -9.27 | -2.37 |
| 1-Benzyl-3-methylimidazolium tetrafluoroborate | 222.90 | 387.50 | 401.07 | -13.57 | -3.50 |
| Dibenzyldisulfide | 223.60 | 420.71 | 402.35 | 18.36 | 4.36 |
| Ethyl decanoate | 223.80 | 403.34 | 402.72 | 0.62 | 0.15 |
| Methyl undecylate | 223.80 | 406.24 | 402.72 | 3.52 | 0.87 |
| Hexyl hexanoate | 223.80 | 394.92 | 402.72 | -7.80 | -1.98 |
| m-Terphenyl | 223.80 | 417.10 | 402.72 | 14.38 | 3.45 |
| Butyl octanoate | 223.90 | 397.41 | 402.90 | -5.49 | -1.38 |
| Octyl butanoate | 223.90 | 383.28 | 402.90 | -19.62 | -5.12 |
| o-Terphenyl | 224.00 | 369.05 | 403.09 | -34.04 | -9.22 |
| 1-Butyl-3-methylimidazolium hexafluorophosphate | 224.10 | 407.70 | 403.27 | 4.43 | 1.09 |
| Perfluorobicyclo[4.4.0]dec-1,6-ene | 225.10 | 428.40 | 405.10 | 23.30 | 5.44 |
| 1-Butyl-1-methylpyrrolidinium dicyanamide | 225.80 | 413.00 | 406.38 | 6.62 | 1.60 |
| 2,5,8,11,14-Pentaoxopentadecane | 226.40 | 457.10 | 407.48 | 49.62 | 10.86 |
| 1-(3-Cyanopropyl)-2,3-dimethylimidazolium dicyanamide | 227.80 | 444.00 | 410.04 | 33.96 | 7.65 |
| 1-Butyl-3-methylimidazolium trifluoromethylsulfonate | 228.20 | 417.00 | 410.77 | 6.23 | 1.49 |
| 6,10-Dimethyl-2-undecanone | 228.50 | 428.40 | 411.32 | 17.08 | 3.99 |
| Tridecane | 228.60 | 407.10 | 411.51 | -4.41 | -1.08 |
| 1,1,3,3,5,5-Hexamethyl-1,3,5-trisilacyclohexane | 229.00 | 400.95 | 412.24 | -11.29 | -2.82 |
| Diethynyldiphenylsilane | 229.00 | 382.00 | 412.24 | -30.24 | -7.92 |
| 1-Bromododecane | 230.30 | 399.31 | 414.62 | -15.31 | -3.83 |
| 1,6-Dibromo-2,3,5-trichlorononafluorohexane | 230.40 | 418.60 | 414.80 | 3.80 | 0.91 |
| n-Dodecylmercaptan | 230.40 | 442.70 | 414.80 | 27.90 | 6.30 |
| 1,3-Bis-(1-isocyanato-1-methylethyl)benzene | 231.70 | 464.00 | 417.18 | 46.82 | 10.09 |
| 1,4-Bis-(1-isocyanato-1-methylethyl)benzene | 231.80 | 415.10 | 417.36 | -2.26 | -0.55 |
| Octamethyltrisiloxane | 231.80 | 420.64 | 417.36 | 3.28 | 0.78 |
| 1-Hexyl-3-methylimidazolium tetrafluoroborate | 234.70 | 416.00 | 422.67 | -6.67 | -1.60 |
| Nonyl methacrylate | 234.90 | 419.30 | 423.04 | -3.74 | -0.89 |
| Dibutyl succinate | 235.20 | 436.24 | 423.59 | 12.65 | 2.90 |
| Perfluoro-cis-decalin | 237.50 | 449.80 | 427.80 | 22.00 | 4.89 |
| 1,2-Diethylpyridinium ethylsulfate | 238.20 | 412.00 | 429.08 | -17.08 | -4.14 |
| Perfluoro-trans-decalin | 239.60 | 446.80 | 431.64 | 15.16 | 3.39 |
| Methyl laurate | 240.20 | 432.68 | 432.74 | -0.06 | -0.01 |
| 1-Butyl-2,3-dimethylimidazolium hexafluorophosphate | 240.70 | 433.60 | 433.65 | -0.05 | -0.01 |
| Diethyleneglycol-di-n-butylether | 240.80 | 452.00 | 433.84 | 18.16 | 4.02 |
| 1-Pentyl-3-methylimidazolium hexafluorophosphate | 241.00 | 437.40 | 434.20 | 3.20 | 0.73 |
| 1-Butyl-1-methylpyrrolidinium trifluoromethanesulfonate | 243.50 | 435.00 | 438.78 | -3.78 | -0.87 |
| Tolbutamide | 244.60 | 450.81 | 440.79 | 10.02 | 2.22 |
| Tetradecane | 244.90 | 438.90 | 441.34 | -2.44 | -0.56 |
| Tripropionine | 245.00 | 481.30 | 441.52 | 39.78 | 8.26 |
| 2-Tetradecanone | 247.30 | 415.20 | 445.73 | -30.53 | -7.35 |
| 1-Octyl-3-methylimidazolium bromide | 247.30 | 392.00 | 445.73 | -53.73 | -13.71 |
| Decylcyclopentane | 250.50 | 426.52 | 451.59 | -25.07 | -5.88 |
| 1-Ethyl-3-methylimidazolium toluenesulfonate | 251.00 | 484.20 | 452.50 | 31.70 | 6.55 |
| Decyl methacrylate | 251.70 | 452.90 | 453.79 | -0.89 | -0.20 |
| Oxacyclohexadecan-2-one | 255.20 | 444.60 | 460.19 | -15.59 | -3.51 |
| 1-Methyl-3-propylimidazolium 2-amino-4-carboxybutanoate | 255.20 | 517.10 | 460.19 | 56.91 | 11.01 |
| Octamethylcyclotetrasiloxane | 255.90 | 495.94 | 461.47 | 34.47 | 6.95 |
| 1-Hexyl-3-methylimidazolium hexafluorophosphate | 256.70 | 424.00 | 462.94 | -38.94 | -9.18 |
| Ethyl dodecanoate | 256.90 | 464.00 | 463.30 | 0.70 | 0.15 |
| 1,4,7,10,13,16-Hexaoxacyclooctadecane | 258.10 | 460.00 | 465.50 | -5.50 | -1.20 |
| 1-Hexyl-3-methylimidazolium trifluoromethylsulfonate | 261.40 | 502.30 | 471.54 | 30.76 | 6.12 |
| Pentadecane | 261.60 | 469.90 | 471.90 | -2.00 | -0.43 |
| 1,1,3,3-Tetraethyl-5,5-dimethylcyclotrisiloxane | 262.00 | 502.90 | 472.64 | 30.26 | 6.02 |
| 1-Bromotetradecane | 263.70 | 450.86 | 475.75 | -24.89 | -5.52 |
| 1-Tetradecanethiol | 263.70 | 501.70 | 475.75 | 25.95 | 5.17 |
| Octamethyltetrasiloxane | 263.90 | 509.60 | 476.11 | 33.49 | 6.57 |
| Ethyl 1-ethylnicotinate ethosulfate | 265.10 | 513.00 | 478.31 | 34.69 | 6.76 |
| Decylcyclohexane | 266.70 | 455.60 | 481.24 | -25.64 | -5.63 |
| N-(4-Methoxybenzylidene)-4-n-butylaniline | 268.10 | 477.00 | 483.80 | -6.80 | -1.43 |
| 1-Butyl-1-methylpyrrolidinium tetracyanoborate | 268.30 | 524.00 | 484.17 | 39.83 | 7.60 |
| Tetrapropyl silicate | 268.40 | 460.10 | 484.35 | -24.25 | -5.27 |
| 1-Octyl-3-methylimidazolium tetrafluoroborate | 268.60 | 498.00 | 484.72 | 13.28 | 2.67 |
| 1-Ethyl-3-methylimidazolium bis(trifluoromethanesulfonyl) amide | 268.70 | 500.00 | 484.90 | 15.10 | 3.02 |
| Pentaethylene glycol dimethyl ether | 269.50 | 547.44 | 486.36 | 61.08 | 11.16 |
| 1-Benzyl-3-methylimidazolium tetrafluoroethanesulfonate | 269.70 | 502.80 | 486.73 | 16.07 | 3.20 |
| 4-Cyano-3-fluorophenyl 4-butylbenzoate | 269.80 | 478.16 | 486.91 | -8.75 | -1.83 |
| 1-Butyl-3-methylpyridinium tetracyanoborate | 270.70 | 495.00 | 488.56 | 6.44 | 1.30 |
| N-Ethylpyridinium bis(trifluoromethylsulfonyl)amide | 270.90 | 502.15 | 488.93 | 13.22 | 2.63 |
| 2,2,4,4,6,8,8-Heptamethylnonane | 271.30 | 458.80 | 489.66 | -30.86 | -6.73 |
| Nonyl N-phenylcarbamate | 272.40 | 471.10 | 491.67 | -20.57 | -4.37 |
| Dibutyl phthalate | 272.70 | 476.00 | 492.22 | -16.22 | -3.41 |
| Cetene | 273.00 | 485.83 | 492.77 | -6.94 | -1.43 |
| Methyl tetradecanoate | 273.60 | 497.91 | 493.87 | 4.04 | 0.81 |
| 1-Ethyl-3-methylimidazolium 2-(2-methoxyethoxy)ethylsulfate | 274.00 | 526.00 | 494.60 | 31.40 | 5.97 |
| 1-Heptyl-3-methylimidazolium hexafluorophosphate | 274.60 | 500.60 | 495.70 | 4.90 | 0.98 |
| 1,1,3,3-Tetramethyl-1,3-diphenyldisiloxane | 277.60 | 508.10 | 501.19 | 6.91 | 1.36 |
| Cetane | 278.20 | 501.61 | 502.29 | -0.68 | -0.13 |
| 4-Butyl-4'-ethoxyazobenzene | 281.80 | 535.10 | 508.88 | 26.22 | 4.90 |
| 1-Ethyl-2,3-dimethylimidazolium bis(trifluoromethanesulfonyl) amide | 282.60 | 493.00 | 510.34 | -17.34 | -3.52 |
| 1-Methyl-3-butylimidazolium saccharinate | 284.30 | 565.66 | 513.45 | 52.21 | 9.23 |
| 1-Isopropyl-3-methylimidazolium bis(trifluoromethanesulfonyl) amide | 284.90 | 529.90 | 514.55 | 15.35 | 2.90 |
| 1-Propyl-3-methylimidazolium bis(trifluoromethanesulfonyl) amide | 285.10 | 534.90 | 514.92 | 19.98 | 3.74 |
| 1-Butyl-3-methylimidazolium toluenesulfonate | 285.80 | 548.40 | 516.20 | 32.20 | 5.87 |
| N-Ethyl-2-methylpyridinium bis(trifluoromethylsulfonyl)amide | 286.50 | 534.50 | 517.48 | 17.02 | 3.18 |
| 1,1,1,3,5,5,5-Heptamethyl-3-phenyltrisiloxane | 287.30 | 519.60 | 518.94 | 0.66 | 0.13 |
| N,N-Dimethyl-2-pentylnonylamine | 288.10 | 538.10 | 520.41 | 17.69 | 3.29 |
| Ethyl myristate | 290.20 | 526.32 | 524.25 | 2.07 | 0.39 |
| Di-n-octylamine | 290.90 | 507.20 | 525.53 | -18.33 | -3.61 |
| 1-Octyl-3-methylimidazolium hexafluorophosphate | 291.40 | 536.10 | 526.45 | 9.65 | 1.80 |
| Hexaethylcyclohexane | 293.30 | 530.10 | 529.92 | 0.18 | 0.03 |
| Decamethyltetrasiloxane | 294.20 | 538.77 | 531.57 | 7.20 | 1.34 |
| 1-Octyl-3-methylimidazolium trifluoromethylsulfonate | 294.80 | 577.70 | 532.67 | 45.03 | 7.79 |
| Heptadecane | 294.90 | 534.34 | 532.85 | 1.49 | 0.28 |
| Hexaethylcyclotrisiloxane | 295.30 | 535.10 | 533.58 | 1.52 | 0.28 |
| 1-Cyclopropylmethyl-3-methylimidazolium bis(trifluoromethanesulfonyl) amide | 295.80 | 539.10 | 534.50 | 4.60 | 0.85 |
| Glyceryl tributanoate | 296.10 | 555.30 | 535.05 | 20.25 | 3.65 |
| 1-Hexadecanethiol | 297.10 | 574.00 | 536.88 | 37.12 | 6.47 |
| Trimethyl butylammonium bis(trifluoromethylsulfonyl)amide | 298.10 | 559.20 | 538.71 | 20.49 | 3.66 |
| 6,10,14-Trimethylpentadeca-3,5-diene-2-one | 298.70 | 555.20 | 539.81 | 15.39 | 2.77 |
| 1,2-Diethylpyridinium bis(trifluoromethanesulfonyl) amide | 299.20 | 566.10 | 540.72 | 25.38 | 4.48 |
| 1-Butyl-3-methylimidazolium bis(trifluoromethanesulfonyl) amide | 301.00 | 536.00 | 544.02 | -8.02 | -1.50 |
| 1-sec-Butyl-3-methylimidazolium bis(trifluoromethanesulfonyl) amide | 301.20 | 557.10 | 544.38 | 12.72 | 2.28 |
| 1-Methyl-1-propylpyrrolidinium bis(trifluoromethanesulfonyl) amide | 301.70 | 554.00 | 545.30 | 8.70 | 1.57 |
| 1-Isobutyl-3-methylimidazolium bis(trifluoromethanesulfonyl) amide | 301.70 | 557.10 | 545.30 | 11.80 | 2.12 |
| 1-Propyl-2,3-dimethylimidazolium bis(trifluoromethanesulfonyl) amide | 301.90 | 554.50 | 545.66 | 8.84 | 1.59 |
| N-Propyl-2-methylpyridinium bis(trifluoromethylsulfonyl)amide | 303.10 | 557.96 | 547.86 | 10.10 | 1.81 |
| N-Butylpyridinium bis(trifluoromethanesulfonyl) amide | 303.60 | 566.52 | 548.78 | 17.74 | 3.13 |
| N-Propyl-3-methylpyridinium bis(trifluoromethylsulfonyl)amide | 303.90 | 517.00 | 549.32 | -32.32 | -6.25 |
| 1-Nonyl-3-methylimidazolium hexafluorophosphate | 307.30 | 569.40 | 555.55 | 13.85 | 2.43 |
| N-Octylisoquinolinium thiocyanate | 309.80 | 522.00 | 560.12 | -38.12 | -7.30 |
| Octadecane | 311.60 | 564.40 | 563.42 | 0.98 | 0.17 |
| N-Ethyl-4-dimethylaminopyridinium bis(trifluoromethanesulfonyl) amide | 315.50 | 594.30 | 570.56 | 23.74 | 4.00 |
| 1-Pentyl-3-methylimidazolium bis(trifluoromethanesulfonyl) amide | 317.10 | 595.60 | 573.48 | 22.12 | 3.71 |
| Decamethylcyclopentasiloxane | 318.70 | 634.63 | 576.41 | 58.22 | 9.17 |
| 1-Ethyl-2-propylpyridinium bis(trifluoromethanesulfonyl) amide | 318.90 | 593.90 | 576.78 | 17.12 | 2.88 |
| 1-Isobutyl-1-methylpyrrolidinium bis(trifluoromethylsulfonyl)amide | 319.80 | 582.20 | 578.43 | 3.77 | 0.65 |
| 1-Isobutyl-3-methylpyridinium bis(trifluoromethylsulfonyl)amide | 320.10 | 579.00 | 578.97 | 0.03 | 0.00 |
| N-Butyl-3-methylpyridinium bis(trifluoromethylsulfonyl)amide | 320.40 | 578.10 | 579.52 | -1.42 | -0.25 |
| 1-Butyl-1-methylpyrrolidinium bis(trifluoromethylsulfonyl)amide | 320.80 | 572.00 | 580.26 | -8.26 | -1.44 |
| 1-Butyl-3-cyanopyridinium bis(trifluoromethylsulfonyl)amide | 321.60 | 586.00 | 581.72 | 4.28 | 0.73 |
| Trihexylamine | 322.40 | 568.12 | 583.18 | -15.06 | -2.65 |
| 1-Benzyl-3-methylimidazolium bis(trifluoromethylsulfonyl) amide | 322.60 | 607.80 | 583.55 | 24.25 | 3.99 |
| 1-Decyl-3-methylimidazolium hexafluorophosphate | 324.70 | 603.30 | 587.39 | 15.91 | 2.64 |
| Pristane | 325.30 | 569.76 | 588.49 | -18.73 | -3.29 |
| 1-Cyclopentylmethyl-3-methylimidazolium bis(trifluoromethanesulfonyl) amide | 325.40 | 560.60 | 588.68 | -28.08 | -5.01 |
| 1-Chlorooctadecane | 326.30 | 606.70 | 590.32 | 16.38 | 2.70 |
| Tri-p-cresyl phosphate | 328.10 | 578.00 | 593.62 | -15.62 | -2.70 |
| Nonadecane | 328.30 | 604.00 | 593.98 | 10.02 | 1.66 |
| Tri(butyl) methylphosphonium methylsulfate | 328.90 | 617.80 | 595.08 | 22.72 | 3.68 |
| Stearyl mercaptan | 330.50 | 648.00 | 598.01 | 49.99 | 7.71 |
| Perfluorododecane | 333.40 | 546.80 | 603.32 | -56.52 | -10.34 |
| 1,1,1,5,5,5-Hexamethyl-3,3-diphenyltrisiloxane | 333.60 | 648.00 | 603.68 | 44.32 | 6.84 |
| 1-Hexyl-3-methylimidazolium bis(trifluoromethanesulfonyl) amide | 335.00 | 629.20 | 606.25 | 22.95 | 3.65 |
| Tetrabutoxysilane | 335.20 | 580.20 | 606.61 | -26.41 | -4.55 |
| Dibutyl decanedioate | 336.00 | 619.00 | 608.08 | 10.92 | 1.76 |
| 1-Butyl-1-methylpiperidinium bis(trifluoromethylsulfonyl)amide | 336.30 | 607.50 | 608.62 | -1.12 | -0.19 |
| 1-Ethyl-2-butylpyridinium bis(trifluoromethanesulfonyl) amide | 336.30 | 623.60 | 608.62 | 14.98 | 2.40 |
| N-Hexylpyridinium bis(trifluoromethanesulfonyl) amide | 336.70 | 612.00 | 609.36 | 2.64 | 0.43 |
| 1-Methyl-1-pentylpyrrolidinium bis(trifluoromethanesulfonyl) amide | 337.50 | 622.60 | 610.82 | 11.78 | 1.89 |
| 1-Butyl-3-methylimidazolium octylsulfate | 338.50 | 635.00 | 612.65 | 22.35 | 3.52 |
| 1-Cyclohexylmethyl-3-methylimidazolium bis(trifluoromethanesulfonyl) amide | 341.40 | 617.10 | 617.96 | -0.86 | -0.14 |
| Eicosane | 345.50 | 663.60 | 625.46 | 38.14 | 5.75 |
| N-Butyl-4-dimethylaminopyridinium bis(trifluoromethanesulfonyl) amide | 347.60 | 657.71 | 629.31 | 28.40 | 4.32 |
| 1-Heptyl-3-methylimidazolium bis(trifluoromethanesulfonyl) amide | 349.90 | 659.20 | 633.52 | 25.68 | 3.90 |
| 2,3-Dimethyl-1-Hexylimidazolium bis(trifluoromethanesulfonyl) amide | 350.70 | 686.00 | 634.98 | 51.02 | 7.44 |
| 1-Ethyl-2-pentylpyridinium bis(trifluoromethanesulfonyl) amide | 353.00 | 652.70 | 639.19 | 13.51 | 2.07 |
| 1,1-Diphenyldodecane | 353.00 | 593.70 | 639.19 | -45.49 | -7.66 |
| 1-Hexyl-3-methylpyridinium bis(trifluoromethylsulfonyl)amide | 353.60 | 624.00 | 640.29 | -16.29 | -2.61 |
| 1-Hexyl-1-methylpyrrolidinium bis(trifluoromethanesulfonyl) amide | 354.20 | 655.10 | 641.39 | 13.71 | 2.09 |
| 1-Hexyl-4-cyanopyridinium bis(trifluoromethylsulfonyl)amide | 354.70 | 633.00 | 642.30 | -9.30 | -1.47 |
| 1-Hexyl-3-cyanopyridinium bis(trifluoromethylsulfonyl)amide | 354.70 | 658.00 | 642.30 | 15.70 | 2.39 |
| Ethyl stearate | 357.40 | 605.00 | 647.24 | -42.24 | -6.98 |
| 1-Dodecyl-3-methylimidazolium hexafluorophosphate | 358.50 | 666.30 | 649.26 | 17.04 | 2.56 |
| 1-Octyl-3-methylimidazolium bis(trifluoromethanesulfonyl) amide | 368.10 | 654.00 | 666.83 | -12.83 | -1.96 |
| 1,1-Diphenyl-3,3,5,5,7,7-hexamethylcyclotetrasiloxane | 368.30 | 633.00 | 667.19 | -34.19 | -5.40 |
| 1-Methyl-1-heptylpyrrolidinium bis(trifluoromethanesulfonyl) amide | 368.50 | 685.10 | 667.56 | 17.54 | 2.56 |
| 1-Octylpyridinium bis(trifluoromethylsulfonyl)amide | 369.10 | 686.00 | 668.66 | 17.34 | 2.53 |
| 1-Ethyl-2-hexylpyridinium bis(trifluoromethanesulfonyl) amide | 369.80 | 685.50 | 669.94 | 15.56 | 2.27 |
| 1,1,3,3-Tetraethyl-5,5-diphenylcylotrisiloxane | 370.10 | 629.60 | 670.49 | -40.89 | -6.49 |
| 1-Hexyl-3,5-dimethylpyridinium bis(trifluoromethylsulfonyl)amide | 370.20 | 620.00 | 670.67 | -50.67 | -8.17 |
| 1-(3,4,5,6-Perfluorohexyl)-3-methylimidazolium-3-methylimidazolium bis(trifluoromethanesulfonyl) amide | 376.10 | 725.00 | 681.47 | 43.53 | 6.00 |
| Docosane | 378.90 | 698.00 | 686.59 | 11.41 | 1.63 |
| 4-Dimethylamino-1-hexylpyridinium bis(trifluoromethanesulfonyl) amide | 380.90 | 731.00 | 690.25 | 40.75 | 5.57 |
| Octaethylcyclotetrasiloxane | 385.40 | 746.00 | 698.49 | 47.51 | 6.37 |
| N-Octyl-3-methylpyridinium bis(trifluoromethanesulfonyl) amide | 386.30 | 669.00 | 700.14 | -31.14 | -4.65 |
| 1-Ethyl-2-heptylpyridinium bis(trifluoromethanesulfonyl) amide | 386.40 | 717.50 | 700.32 | 17.18 | 2.39 |
| 1-Methyl-1-octylpyrrolidinium bis(trifluoromethanesulfonyl) amide | 387.60 | 716.30 | 702.52 | 13.78 | 1.92 |
| 1-Octyl-3-cyanopyridinium bis(trifluoromethylsulfonyl)amide | 387.90 | 709.00 | 703.07 | 5.93 | 0.84 |
| 4'-Heptyl-m-tercyclohexyl | 393.10 | 668.60 | 712.58 | -43.98 | -6.58 |
| 4'-Heptyl-p-tercyclohexyl | 393.20 | 752.70 | 712.77 | 39.93 | 5.31 |
| 1-Hexyl-3-methylimidazolium tris(pentafluoroethyl)trifluorophosphate | 393.70 | 730.00 | 713.68 | 16.32 | 2.24 |
| N-Hexyl-3-methyl-4-dimethylaminopyridinium bis(trifluoromethanesulfonyl) amide | 393.90 | 725.00 | 714.05 | 10.95 | 1.51 |
| Butyl 1-butylnicotinate bis(trifluoromethylsulfonyl)amide | 395.30 | 707.00 | 716.61 | -9.61 | -1.36 |
| Glycerol tricaproate | 397.20 | 753.10 | 720.09 | 33.01 | 4.38 |
| Bis(2-ethylhexyl) adipate | 399.20 | 701.50 | 723.75 | -22.25 | -3.17 |
| Ethyl tri(butyl)phosphonium diethylphosphate | 400.60 | 711.00 | 726.31 | -15.31 | -2.15 |
| 1-Decyl-3-methylimidazolium bis(trifluoromethanesulfonyl) amide | 401.30 | 754.80 | 727.59 | 27.21 | 3.60 |
| 1-Ethyl-2-octylpyridinium bis(trifluoromethanesulfonyl) amide | 401.60 | 749.40 | 728.14 | 21.26 | 2.84 |
| Di-n-octyladipate | 402.60 | 701.50 | 729.97 | -28.47 | -4.06 |
| Dihexyl sebacate | 402.70 | 710.90 | 730.15 | -19.25 | -2.71 |
| DEHP | 403.70 | 708.98 | 731.98 | -23.00 | -3.24 |
| Bis(1-ethylhexyl) o-phthalate | 405.00 | 704.60 | 734.36 | -29.76 | -4.22 |
| Di-n-octyl phthalate | 406.70 | 707.10 | 737.47 | -30.37 | -4.30 |
| 1-Ethyl-2-nonylpyridinium bis(trifluoromethanesulfonyl) amide | 416.10 | 778.50 | 754.68 | 23.82 | 3.06 |
| Tetrabutylphosphonium L-valinate | 417.30 | 747.00 | 756.88 | -9.88 | -1.32 |
| 1-Methyl-1-decylpyrrolidinium bis(trifluoromethanesulfonyl) amide | 421.00 | 778.90 | 763.65 | 15.25 | 1.96 |
| Tri-n-octylamine | 422.90 | 750.80 | 767.13 | -16.33 | -2.17 |
| Pentacosane | 429.00 | 769.00 | 778.29 | -9.29 | -1.21 |
| 11-Cyclohexyleicosane | 432.40 | 787.40 | 784.51 | 2.89 | 0.37 |
| 1-Dodecyl-3-methylimidazolium bis(trifluoromethanesulfonyl) amide | 435.80 | 820.20 | 790.74 | 29.46 | 3.59 |
| 1-Ethyl-2-decylpyridinium bis(trifluoromethanesulfonyl) amide | 436.60 | 811.20 | 792.20 | 19.00 | 2.34 |
| 1-Hexyl-2-propyl-3,5-diethylpyridinium bis(trifluoromethylsulfonyl)amide | 448.10 | 766.00 | 813.25 | -47.25 | -6.17 |
| Bis(2-ethylhexyl) azelate | 450.20 | 799.70 | 817.09 | -17.39 | -2.17 |
| Heptacosane | 462.40 | 828.40 | 839.42 | -11.02 | -1.33 |
| Bis(2-ethylhexyl) sebacate | 467.30 | 849.40 | 848.39 | 1.01 | 0.12 |
| Vitamin E acetate | 500.90 | 897.90 | 909.89 | -11.99 | -1.33 |
| Dodecahydrosqualene | 506.50 | 886.36 | 920.13 | -33.77 | -3.81 |
| 11-Decylheneicosane | 527.50 | 949.80 | 958.57 | -8.77 | -0.92 |
| Unatriacontane | 529.20 | 912.10 | 961.68 | -49.58 | -5.44 |
| Glyceryl tricaprate | 595.10 | 1108.80 | 1082.30 | 26.50 | 2.39 |
| Tetra-1-octoxysilane | 602.30 | 1095.40 | 1095.47 | -0.07 | -0.01 |
| Tetradecyl trihexylphosphonium bis(trifluoromethylsulfonyl)amide | 726.80 | 1298.80 | 1323.34 | -24.54 | -1.89 |
| Tetra-1-decoxysilane | 736.00 | 1373.54 | 1340.18 | 33.36 | 2.43 |
| Trihexyltetradecylphosphonium tris(pentafluoroethyl)trifluorophosphate | 792.60 | 1441.40 | 1443.77 | -2.37 | -0.16 |
| Benzene hexa-n-heptanoate | 848.20 | 1500.00 | 1545.53 | -45.53 | -3.04 |
| Trihexyltetradecylphosphonium bis(2,2,4-trimethylpentyl)phosphinate | 875.80 | 1635.00 | 1596.05 | 38.95 | 2.38 |
| Trielaidin | 979.20 | 1841.76 | 1785.30 | 56.46 | 3.07 |
| Tristearin | 997.60 | 1787.47 | 1818.97 | -31.50 | -1.76 |
| 2,3,6,7,10,11-Hexaoctanoyloxytriphenylene | 1072.50 | 1956.10 | 1956.06 | 0.04 | 0.00 |
