# Supplementary material for: Calculation of the Isobaric Heat Capacities of the Liquid and Solid Phase of Organic Compounds at and around 298.15 K Based on Their “True” Molecular Volume
Source: Molecules. 2019 Apr 24;24(8):1626. doi: 10.3390/molecules24081626 (PMC6514989; doi:10.3390/molecules24081626)
Supplement: Supplementary file 1 [file molecules-24-01626-s001.zip › S05. Experimental and Calculated Cp(liq,298) Data Table to Figure 6.doc]

| **Molecule name** | **Molecular volume** | **Cp(liq,298) exp** | **Cp(liq,298) calc** | **Deviation** | **Dev. in %** |
| --- | --- | --- | --- | --- | --- |
| Water | 19.20 | 75.32 | 55.93 | 19.39 | 25.74 |
| Methanol | 36.90 | 81.92 | 91.18 | -9.26 | -11.30 |
| Formic acid | 39.90 | 99.04 | 97.15 | 1.89 | 1.91 |
| Ethanol | 53.60 | 115.90 | 124.43 | -8.53 | -7.36 |
| Acetic acid | 56.70 | 123.10 | 130.60 | -7.50 | -6.09 |
| Ethan-1,2-diol | 62.70 | 149.80 | 142.55 | 7.25 | 4.84 |
| Allyl alcohol | 65.10 | 146.30 | 147.32 | -1.02 | -0.70 |
| Colamine | 65.60 | 137.70 | 148.32 | -10.62 | -7.71 |
| Acrylic acid | 67.40 | 144.20 | 151.90 | -7.70 | -5.34 |
| 2-Propanol | 70.30 | 154.43 | 157.68 | -3.25 | -2.10 |
| 1-Propanol | 70.30 | 146.88 | 157.68 | -10.80 | -7.35 |
| Chloroacetic acid | 70.40 | 179.90 | 157.88 | 22.02 | 12.24 |
| Propanoic acid | 73.30 | 158.60 | 163.65 | -5.05 | -3.19 |
| Cyclobutanol | 78.10 | 153.70 | 173.21 | -19.51 | -12.69 |
| Propan-1,3-diol | 79.40 | 175.78 | 175.80 | -0.02 | -0.01 |
| 1,2-Propanediol | 79.40 | 180.30 | 175.80 | 4.50 | 2.50 |
| Methyl cellosolve | 80.10 | 176.40 | 177.19 | -0.79 | -0.45 |
| DL-Lactic acid | 81.80 | 210.50 | 180.58 | 29.92 | 14.22 |
| Lactic acid | 81.80 | 210.50 | 180.58 | 29.92 | 14.22 |
| Hydroxymethyl acetate | 82.40 | 214.00 | 181.77 | 32.23 | 15.06 |
| Methacrylic acid | 84.00 | 161.10 | 184.96 | -23.86 | -14.81 |
| Dichloroacetic acid | 84.50 | 188.00 | 185.95 | 2.05 | 1.09 |
| 2-Butanol | 86.50 | 196.67 | 189.93 | 6.74 | 3.43 |
| 2-Methyl-1-propanol | 86.50 | 181.05 | 189.93 | -8.88 | -4.91 |
| 2-Methyl-2-propanol | 86.80 | 218.60 | 190.53 | 28.07 | 12.84 |
| 1-Butanol | 87.00 | 177.16 | 190.93 | -13.77 | -7.77 |
| 1,2,3-Propanetriol | 88.20 | 218.90 | 193.32 | 25.58 | 11.69 |
| Furfuranol | 89.80 | 204.01 | 196.50 | 7.51 | 3.68 |
| 2-Methylpropanoic acid | 89.80 | 181.70 | 196.50 | -14.80 | -8.15 |
| Butanoic acid | 89.90 | 177.70 | 196.70 | -19.00 | -10.69 |
| Phenol | 92.30 | 200.00 | 201.48 | -1.48 | -0.74 |
| Cyclopentanol | 93.40 | 185.40 | 203.67 | -18.27 | -9.86 |
| 2,3-Butyleneglycol | 96.00 | 213.00 | 208.85 | 4.15 | 1.95 |
| 1,2-Butanediol | 96.00 | 230.82 | 208.85 | 21.97 | 9.52 |
| Tetramethylene glycol | 96.10 | 203.79 | 209.05 | -5.26 | -2.58 |
| 1,3-Butyleneglycol | 96.10 | 218.89 | 209.05 | 9.84 | 4.50 |
| 2-Ethoxyethanol | 96.90 | 210.30 | 210.64 | -0.34 | -0.16 |
| Dimethylvinylcarbinol | 97.80 | 208.40 | 212.43 | -4.03 | -1.94 |
| Acetylacetone (enol form) | 98.60 | 208.40 | 214.03 | -5.63 | -2.70 |
| Cellosolve acetate | 98.70 | 203.00 | 214.23 | -11.23 | -5.53 |
| 2-Aminoisobutanol | 98.70 | 229.50 | 214.23 | 15.27 | 6.66 |
| 2-(Ethylamino)ethanol | 99.40 | 227.00 | 215.62 | 11.38 | 5.01 |
| Butanedioic acid | 100.90 | 238.60 | 218.61 | 19.99 | 8.38 |
| Tetrahydrofurfuryl alcohol | 102.40 | 190.00 | 221.59 | -31.59 | -16.63 |
| 2-Methyl-2-butanol | 102.90 | 247.30 | 222.59 | 24.71 | 9.99 |
| Isopentyl alcohol | 103.00 | 209.60 | 222.79 | -13.19 | -6.29 |
| 3-Methyl-2-butanol | 103.10 | 245.90 | 222.99 | 22.91 | 9.32 |
| Neopentyl alcohol | 103.50 | 244.30 | 223.78 | 20.52 | 8.40 |
| 1-Pentanol | 103.60 | 208.14 | 223.98 | -15.84 | -7.61 |
| 3-Pentanol | 103.80 | 239.70 | 224.38 | 15.32 | 6.39 |
| Diethyleneglycol | 105.80 | 243.90 | 228.36 | 15.54 | 6.37 |
| 3-Methylbutanoic acid | 106.20 | 197.10 | 229.16 | -32.06 | -16.26 |
| Pentanoic acid | 106.60 | 210.00 | 229.95 | -19.95 | -9.50 |
| o-Cresol | 108.50 | 229.75 | 233.74 | -3.99 | -1.74 |
| Benzyl alcohol | 108.60 | 215.90 | 233.94 | -18.04 | -8.35 |
| p-Cresol | 108.80 | 221.03 | 234.33 | -13.30 | -6.02 |
| m-Cresol | 109.00 | 224.93 | 234.73 | -9.80 | -4.36 |
| Cyclohexanol | 109.50 | 213.40 | 235.73 | -22.33 | -10.46 |
| 2-Hydroxybenzaldehyde | 109.90 | 222.00 | 236.53 | -14.53 | -6.54 |
| 1,5-Pentanediol | 112.80 | 232.49 | 242.30 | -9.81 | -4.22 |
| 2-Isopropoxyethanol | 113.20 | 238.80 | 243.10 | -4.30 | -1.80 |
| 2-Propoxyethanol | 113.60 | 241.60 | 243.89 | -2.29 | -0.95 |
| cis-3-Hexen-1-ol | 114.30 | 234.00 | 245.29 | -11.29 | -4.82 |
| trans-3-Hexen-1-ol | 114.60 | 237.20 | 245.88 | -8.68 | -3.66 |
| 1-Hexen-3-ol | 114.60 | 269.30 | 245.88 | 23.42 | 8.70 |
| 2-(Isopropylamino)ethanol | 115.50 | 258.82 | 247.68 | 11.14 | 4.31 |
| Ethyl lactate | 115.80 | 254.00 | 248.27 | 5.73 | 2.25 |
| Pentanedioic acid | 117.60 | 270.50 | 251.86 | 18.64 | 6.89 |
| 2-Methoxyphenol | 118.10 | 240.00 | 252.85 | -12.85 | -5.36 |
| Tetrahydro-2H-pyran-2-methanol | 119.00 | 222.00 | 254.64 | -32.64 | -14.70 |
| 2-Ethyl-1-butanol | 119.30 | 246.65 | 255.24 | -8.59 | -3.48 |
| 3-Methyl-3-pentanol | 119.40 | 293.30 | 255.44 | 37.86 | 12.91 |
| 3,3-Dimethyl-1-butanol | 119.60 | 236.08 | 255.84 | -19.76 | -8.37 |
| 4-Methyl-2-pentanol | 119.70 | 272.34 | 256.04 | 16.30 | 5.99 |
| 3-Methyl-2-pentanol | 119.70 | 275.89 | 256.04 | 19.85 | 7.20 |
| 3-Hexanol | 119.90 | 269.27 | 256.44 | 12.83 | 4.77 |
| 2-Methyl-2-pentanol | 120.00 | 289.03 | 256.64 | 32.39 | 11.21 |
| 2-Methyl-1-pentanol | 120.10 | 248.40 | 256.83 | -8.43 | -3.40 |
| 4-Hydroxy-4-methyl-2-pentanone | 120.50 | 221.30 | 257.63 | -36.33 | -16.42 |
| 2-Hexanol | 120.60 | 256.31 | 257.83 | -1.52 | -0.59 |
| 1-Hexanol | 120.60 | 242.70 | 257.83 | -15.13 | -6.23 |
| n-Hexanoic acid | 123.30 | 225.10 | 263.21 | -38.11 | -16.93 |
| s-Phenethyl alcohol | 124.20 | 257.45 | 265.00 | -7.55 | -2.93 |
| Phenethyl alcohol | 124.50 | 252.64 | 265.59 | -12.95 | -5.13 |
| cis-2-Methylcyclohexanol | 125.30 | 268.95 | 267.19 | 1.76 | 0.66 |
| 1-Methylcyclohexanol | 125.30 | 279.05 | 267.19 | 11.86 | 4.25 |
| Cyclohexanemethanol | 125.30 | 236.50 | 267.19 | -30.69 | -12.98 |
| Cycloheptanol | 125.50 | 250.22 | 267.59 | -17.37 | -6.94 |
| trans-2-Methylcyclohexanol | 126.10 | 262.98 | 268.78 | -5.80 | -2.21 |
| trans-4-Methylcyclohexanol | 126.10 | 202.10 | 268.78 | -66.68 | -32.99 |
| cis-3-Methylcyclohexanol | 126.10 | 199.60 | 268.78 | -69.18 | -34.66 |
| Hexyleneglycol | 126.60 | 263.10 | 269.78 | -6.68 | -2.54 |
| 2,4-Dibromophenol | 129.10 | 259.40 | 274.75 | -15.35 | -5.92 |
| 2-t-Butoxyethanol | 129.40 | 273.45 | 275.35 | -1.90 | -0.70 |
| 1,2-Hexanediol | 129.40 | 293.10 | 275.35 | 17.75 | 6.06 |
| Butylcellosolve | 130.20 | 271.66 | 276.94 | -5.28 | -1.95 |
| 2-Diethylaminoethanol | 130.80 | 281.20 | 278.14 | 3.06 | 1.09 |
| 2-Phenoxyethanol | 134.80 | 294.63 | 286.10 | 8.53 | 2.89 |
| 1-Naphthol | 135.40 | 284.50 | 287.30 | -2.80 | -0.98 |
| Triethylmethanol | 135.50 | 353.90 | 287.50 | 66.40 | 18.76 |
| 2,2-Dimethyl-3-pentanol | 135.90 | 349.00 | 288.29 | 60.71 | 17.39 |
| 2,4-Dimethyl-3-pentanol | 135.90 | 359.00 | 288.29 | 70.71 | 19.70 |
| 2-Methyl-2-hexanol | 136.70 | 313.54 | 289.89 | 23.65 | 7.54 |
| 2-Methyl-3-hexanol | 136.70 | 324.00 | 289.89 | 34.11 | 10.53 |
| 5-Methyl-2-hexanol | 136.80 | 295.20 | 290.09 | 5.11 | 1.73 |
| 2-Heptanol | 137.20 | 298.63 | 290.88 | 7.75 | 2.59 |
| 4-Heptanol | 137.20 | 306.77 | 290.88 | 15.89 | 5.18 |
| 3-Heptanol | 137.20 | 314.20 | 290.88 | 23.32 | 7.42 |
| 1-Heptanol | 137.30 | 274.81 | 291.08 | -16.27 | -5.92 |
| Dipropylene glycol | 138.80 | 322.10 | 294.07 | 28.03 | 8.70 |
| Enanthic acid | 139.80 | 267.31 | 296.06 | -28.75 | -10.75 |
| Hydrocinnamyl alcohol | 141.10 | 280.74 | 298.65 | -17.91 | -6.38 |
| Cyclohexaneethanol | 142.50 | 266.00 | 301.43 | -35.43 | -13.32 |
| 1,7-Heptanediol | 146.20 | 297.00 | 308.80 | -11.80 | -3.97 |
| Triethyleneglycol | 148.80 | 327.60 | 313.98 | 13.62 | 4.16 |
| 2-Hydroxyethyl-2',2'-dimethylpropionate | 148.90 | 308.00 | 314.18 | -6.18 | -2.01 |
| Heptanedioic acid | 151.00 | 334.30 | 318.36 | 15.94 | 4.77 |
| 4-Ethyl-3-hexanol | 152.20 | 361.26 | 320.75 | 40.51 | 11.21 |
| 4-Methyl-2-heptanol | 152.70 | 312.50 | 321.74 | -9.24 | -2.96 |
| Ethyl salicylate | 152.70 | 283.07 | 321.74 | -38.67 | -13.66 |
| 4-Methyl-3-heptanol | 152.80 | 309.20 | 321.94 | -12.74 | -4.12 |
| 3-Methyl-2-heptanol | 152.80 | 297.50 | 321.94 | -24.44 | -8.22 |
| 3-Methyl-4-heptanol | 152.80 | 355.78 | 321.94 | 33.84 | 9.51 |
| 2,5-Dimethyl-3-hexanol | 152.90 | 374.15 | 322.14 | 52.01 | 13.90 |
| 2-Ethyl-1-hexanol | 153.00 | 317.50 | 322.34 | -4.84 | -1.52 |
| 4-Methyl-4-heptanol | 153.00 | 366.92 | 322.34 | 44.58 | 12.15 |
| 2-Methyl-4-heptanol | 153.10 | 331.80 | 322.54 | 9.26 | 2.79 |
| 5-Methyl-2-heptanol | 153.10 | 296.20 | 322.54 | -26.34 | -8.89 |
| 5-Methyl-1-heptanol | 153.20 | 304.20 | 322.74 | -18.54 | -6.09 |
| 6-Methyl-3-heptanol | 153.40 | 310.50 | 323.14 | -12.64 | -4.07 |
| 2-Methyl-2-heptanol | 153.40 | 337.60 | 323.14 | 14.46 | 4.28 |
| 6-Methyl-2-heptanol | 153.50 | 315.10 | 323.34 | -8.24 | -2.61 |
| 2-Methyl-1-heptanol | 153.50 | 313.00 | 323.34 | -10.34 | -3.30 |
| 1-Octanol | 153.70 | 312.10 | 323.73 | -11.63 | -3.73 |
| 4-Octanol | 153.90 | 332.09 | 324.13 | 7.96 | 2.40 |
| 3-Octanol | 153.90 | 338.50 | 324.13 | 14.37 | 4.24 |
| 2-Octanol | 154.00 | 330.10 | 324.33 | 5.77 | 1.75 |
| N-Methyl-2-hydroxyethylammonium propionate | 154.40 | 328.00 | 325.13 | 2.87 | 0.88 |
| n-Octanoic acid | 156.60 | 304.00 | 329.51 | -25.51 | -8.39 |
| Cyclohexanepropanol | 159.20 | 293.00 | 334.69 | -41.69 | -14.23 |
| 4-Allyl-2-methoxyphenol | 161.80 | 343.10 | 339.86 | 3.24 | 0.94 |
| 2-(2-(2-Methoxyethoxy)ethoxy)ethanol | 166.10 | 357.05 | 348.42 | 8.63 | 2.42 |
| Octanedioic acid | 167.20 | 366.20 | 350.61 | 15.59 | 4.26 |
| 3,7-Dimethyl-6-octen-1-yn-3-ol | 170.00 | 385.30 | 356.19 | 29.11 | 7.56 |
| 1-Ethyl-3-methylimidazolium hydrogen sulfate | 170.50 | 295.50 | 357.18 | -61.68 | -20.87 |
| 4-Nonanol | 170.60 | 367.86 | 357.38 | 10.48 | 2.85 |
| 5-Nonanol | 170.60 | 370.75 | 357.38 | 13.37 | 3.61 |
| 3-Nonanol | 170.60 | 373.63 | 357.38 | 16.25 | 4.35 |
| 2-Nonanol | 170.70 | 356.32 | 357.58 | -1.26 | -0.35 |
| 1-Nonanol | 170.70 | 341.00 | 357.58 | -16.58 | -4.86 |
| N-Methyl-2-hydroxyethylammonium butanoate | 171.10 | 361.00 | 358.38 | 2.62 | 0.73 |
| trans-Geraniol | 172.60 | 346.10 | 361.37 | -15.27 | -4.41 |
| Pelargonic acid | 173.20 | 362.37 | 362.56 | -0.19 | -0.05 |
| Diethyleneglycol monobutyl ether | 173.30 | 354.89 | 362.76 | -7.87 | -2.22 |
| Linalool | 174.20 | 372.40 | 364.55 | 7.85 | 2.11 |
| beta-Citronellol | 179.30 | 357.90 | 374.71 | -16.81 | -4.70 |
| 1-Ethyl-3-methylimidazolium methylphosphonate | 183.60 | 354.64 | 383.27 | -28.63 | -8.07 |
| Nonanedioic acid | 184.40 | 398.10 | 384.86 | 13.24 | 3.33 |
| 4-Propyl-4-heptanol | 185.70 | 446.60 | 387.45 | 59.15 | 13.24 |
| 3,7-Dimethyl-1-octanol | 186.10 | 367.21 | 388.25 | -21.04 | -5.73 |
| n-Decyl alcohol | 187.00 | 377.00 | 390.04 | -13.04 | -3.46 |
| 5-Decanol | 187.30 | 405.77 | 390.63 | 15.14 | 3.73 |
| N-Methyl-2-hydroxyethylammonium pentanoate | 187.80 | 401.00 | 391.63 | 9.37 | 2.34 |
| n-Decanoic acid | 189.90 | 361.10 | 395.81 | -34.71 | -9.61 |
| Tetraglycol | 191.80 | 428.90 | 399.59 | 29.31 | 6.83 |
| 2-(2'-Hydroxyethoxy)ethyl pivalate | 191.90 | 368.83 | 399.79 | -30.96 | -8.40 |
| 1-(2-Hydroxyethyl)-3-methyl-1H-imidazolium 2,2,2-trifluoroacetate | 192.20 | 369.00 | 400.39 | -31.39 | -8.51 |
| Decanedioic acid | 201.10 | 430.00 | 418.11 | 11.89 | 2.76 |
| 1-Undecanol | 204.10 | 406.34 | 424.08 | -17.74 | -4.37 |
| n-Undecanoic acid | 206.50 | 415.10 | 428.86 | -13.76 | -3.32 |
| Tripropylene glycol | 214.30 | 440.60 | 444.39 | -3.79 | -0.86 |
| Undecanedioic acid | 217.80 | 461.90 | 451.36 | 10.54 | 2.28 |
| 1-Dodecanol | 220.80 | 438.42 | 457.34 | -18.92 | -4.31 |
| 1H,1H-Perfluorooctan-1-ol | 222.70 | 468.60 | 461.12 | 7.48 | 1.60 |
| Lauric acid | 223.20 | 404.28 | 462.11 | -57.83 | -14.31 |
| Dodecanedioic acid | 234.50 | 493.80 | 484.61 | 9.19 | 1.86 |
| Pentaglycol | 234.80 | 515.50 | 485.21 | 30.29 | 5.88 |
| Tributylmethanol | 235.70 | 548.60 | 487.00 | 61.60 | 11.23 |
| 1-Tridecanol | 237.50 | 476.00 | 490.59 | -14.59 | -3.06 |
| 1H,1H-Perfluorononan-1-ol | 250.30 | 515.90 | 516.07 | -0.17 | -0.03 |
| 1,13-Tridecanedioic acid | 251.20 | 525.70 | 517.86 | 7.84 | 1.49 |
| Pentaethylene glycol monomethyl ether | 252.10 | 537.12 | 519.66 | 17.46 | 3.25 |
| Myristyl alcohol | 254.20 | 505.80 | 523.84 | -18.04 | -3.57 |
| Tetrapropylene glycol | 255.30 | 559.80 | 526.03 | 33.77 | 6.03 |
| 3,7,11-Trimethyl-1-dodecen-3-ol | 262.20 | 574.50 | 539.77 | 34.73 | 6.05 |
| 1,14-Tetradecanedioic acid | 267.90 | 557.60 | 551.12 | 6.48 | 1.16 |
| 1-Pentadecanol | 270.90 | 535.10 | 557.09 | -21.99 | -4.11 |
| 1H,1H-Perfluorodecan-1-ol | 274.50 | 563.90 | 564.26 | -0.36 | -0.06 |
| Hexaethylene glycol | 277.80 | 620.10 | 570.83 | 49.27 | 7.95 |
| Hexadecanedioic acid | 301.30 | 621.40 | 617.62 | 3.78 | 0.61 |
| 1H,1H-Perfluoroundecan-1-ol | 302.10 | 602.40 | 619.21 | -16.81 | -2.79 |
| Pentapropylene glycol | 312.40 | 685.80 | 639.72 | 46.08 | 6.72 |
| (9Z)-Octadecenoic acid | 317.00 | 577.00 | 648.88 | -71.88 | -12.46 |
| Ricinelaidic acid | 325.80 | 646.00 | 666.40 | -20.40 | -3.16 |
| 1H,1H-Perfluorododecan-1-ol | 328.00 | 645.20 | 670.78 | -25.58 | -3.96 |
| Methyltributylammonium serinate | 331.10 | 635.00 | 676.95 | -41.95 | -6.61 |
| Methyltributylammonium L-threoninate | 338.60 | 753.00 | 691.88 | 61.12 | 8.12 |
| 3,7,11,15-Tetramethyl-1-hexadecyn-3-ol | 340.80 | 712.50 | 696.27 | 16.23 | 2.28 |
| Isophytol | 344.10 | 729.70 | 702.84 | 26.86 | 3.68 |
| Heptylpentaoxyethylene | 352.40 | 722.90 | 719.36 | 3.54 | 0.49 |
| Hexapropylene glycol | 369.90 | 807.50 | 754.21 | 53.29 | 6.60 |
| 1H,1H-Perfluorotetradecan-1-ol | 379.80 | 725.30 | 773.92 | -48.62 | -6.70 |
| Tetrabutylphosphonium L-serinate | 389.50 | 748.00 | 793.23 | -45.23 | -6.05 |
