# Supplementary material for: Calculation of the Isobaric Heat Capacities of the Liquid and Solid Phase of Organic Compounds at and around 298.15 K Based on Their “True” Molecular Volume
Source: Molecules. 2019 Apr 24;24(8):1626. doi: 10.3390/molecules24081626 (PMC6514989; doi:10.3390/molecules24081626)
Supplement: Supplementary file 1 [file molecules-24-01626-s001.zip › S06. Experimental and Calculated Cp(liq,298) Data Table to Figure 8.doc]

| **Molecule name** | **Molecular volume** | **Cp(liq,298) exp** | **Cp(liq,298) calc** | **Deviation** | **Dev. in %** |
| --- | --- | --- | --- | --- | --- |
| Methanol | 36.90 | 81.92 | 91.18 | -9.26 | -11.30 |
| Formic acid | 39.90 | 99.04 | 97.06 | 1.98 | 2.00 |
| Ethanol | 53.60 | 115.90 | 123.94 | -8.04 | -6.93 |
| Acetic acid | 56.70 | 123.10 | 130.02 | -6.92 | -5.62 |
| Allyl alcohol | 65.10 | 146.30 | 146.50 | -0.20 | -0.14 |
| Colamine | 65.60 | 137.70 | 147.48 | -9.78 | -7.10 |
| Acrylic acid | 67.40 | 144.20 | 151.01 | -6.81 | -4.72 |
| 1-Propanol | 70.30 | 146.88 | 156.70 | -9.82 | -6.69 |
| 2-Propanol | 70.30 | 154.43 | 156.70 | -2.27 | -1.47 |
| Chloroacetic acid | 70.40 | 179.90 | 156.90 | 23.00 | 12.79 |
| Propanoic acid | 73.30 | 158.60 | 162.58 | -3.98 | -2.51 |
| Cyclobutanol | 78.10 | 153.70 | 172.00 | -18.30 | -11.91 |
| Methyl cellosolve | 80.10 | 176.40 | 175.92 | 0.48 | 0.27 |
| Hydroxymethyl acetate | 82.40 | 214.00 | 180.44 | 33.56 | 15.68 |
| Methacrylic acid | 84.00 | 161.10 | 183.58 | -22.48 | -13.95 |
| Dichloroacetic acid | 84.50 | 188.00 | 184.56 | 3.44 | 1.83 |
| 2-Butanol | 86.50 | 196.67 | 188.48 | 8.19 | 4.16 |
| 2-Methyl-1-propanol | 86.50 | 181.05 | 188.48 | -7.43 | -4.10 |
| 2-Methyl-2-propanol | 86.80 | 218.60 | 189.07 | 29.53 | 13.51 |
| 1-Butanol | 87.00 | 177.16 | 189.46 | -12.30 | -6.94 |
| 2-Methylpropanoic acid | 89.80 | 181.70 | 194.95 | -13.25 | -7.29 |
| Furfuranol | 89.80 | 204.01 | 194.95 | 9.06 | 4.44 |
| Butanoic acid | 89.90 | 177.70 | 195.15 | -17.45 | -9.82 |
| Phenol | 92.30 | 200.00 | 199.86 | 0.14 | 0.07 |
| Cyclopentanol | 93.40 | 185.40 | 202.02 | -16.62 | -8.96 |
| 2-Ethoxyethanol | 96.90 | 210.30 | 208.88 | 1.42 | 0.67 |
| Dimethylvinylcarbinol | 97.80 | 208.40 | 210.65 | -2.25 | -1.08 |
| Acetylacetone (enol form) | 98.60 | 208.40 | 212.22 | -3.82 | -1.83 |
| 2-Aminoisobutanol | 98.70 | 229.50 | 212.41 | 17.09 | 7.44 |
| Cellosolve acetate | 98.70 | 203.00 | 212.41 | -9.41 | -4.64 |
| 2-(Ethylamino)ethanol | 99.40 | 227.00 | 213.79 | 13.21 | 5.82 |
| Tetrahydrofurfuryl alcohol | 102.40 | 190.00 | 219.67 | -29.67 | -15.62 |
| 2-Methyl-2-butanol | 102.90 | 247.30 | 220.65 | 26.65 | 10.77 |
| Isopentyl alcohol | 103.00 | 209.60 | 220.85 | -11.25 | -5.37 |
| 3-Methyl-2-butanol | 103.10 | 245.90 | 221.05 | 24.85 | 10.11 |
| Neopentyl alcohol | 103.50 | 244.30 | 221.83 | 22.47 | 9.20 |
| 1-Pentanol | 103.60 | 208.14 | 222.03 | -13.89 | -6.67 |
| 3-Pentanol | 103.80 | 239.70 | 222.42 | 17.28 | 7.21 |
| 3-Methylbutanoic acid | 106.20 | 197.10 | 227.13 | -30.03 | -15.23 |
| Pentanoic acid | 106.60 | 210.00 | 227.91 | -17.91 | -8.53 |
| o-Cresol | 108.50 | 229.75 | 231.64 | -1.89 | -0.82 |
| Benzyl alcohol | 108.60 | 215.90 | 231.84 | -15.94 | -7.38 |
| p-Cresol | 108.80 | 221.03 | 232.23 | -11.20 | -5.07 |
| m-Cresol | 109.00 | 224.93 | 232.62 | -7.69 | -3.42 |
| Cyclohexanol | 109.50 | 213.40 | 233.60 | -20.20 | -9.47 |
| 2-Hydroxybenzaldehyde | 109.90 | 222.00 | 234.39 | -12.39 | -5.58 |
| 2-Isopropoxyethanol | 113.20 | 238.80 | 240.86 | -2.06 | -0.86 |
| 2-Propoxyethanol | 113.60 | 241.60 | 241.65 | -0.05 | -0.02 |
| cis-3-Hexen-1-ol | 114.30 | 234.00 | 243.02 | -9.02 | -3.85 |
| 1-Hexen-3-ol | 114.60 | 269.30 | 243.61 | 25.69 | 9.54 |
| trans-3-Hexen-1-ol | 114.60 | 237.20 | 243.61 | -6.41 | -2.70 |
| 2-(Isopropylamino)ethanol | 115.50 | 258.82 | 245.37 | 13.45 | 5.20 |
| Ethyl lactate | 115.80 | 254.00 | 245.96 | 8.04 | 3.16 |
| 2-Methoxyphenol | 118.10 | 240.00 | 250.47 | -10.47 | -4.36 |
| Tetrahydro-2H-pyran-2-methanol | 119.00 | 222.00 | 252.24 | -30.24 | -13.62 |
| 2-Ethyl-1-butanol | 119.30 | 246.65 | 252.83 | -6.18 | -2.50 |
| 3-Methyl-3-pentanol | 119.40 | 293.30 | 253.02 | 40.28 | 13.73 |
| 3,3-Dimethyl-1-butanol | 119.60 | 236.08 | 253.42 | -17.34 | -7.34 |
| 3-Methyl-2-pentanol | 119.70 | 275.89 | 253.61 | 22.28 | 8.07 |
| 4-Methyl-2-pentanol | 119.70 | 272.34 | 253.61 | 18.73 | 6.88 |
| 3-Hexanol | 119.90 | 269.27 | 254.00 | 15.27 | 5.67 |
| 2-Methyl-2-pentanol | 120.00 | 289.03 | 254.20 | 34.83 | 12.05 |
| 2-Methyl-1-pentanol | 120.10 | 248.40 | 254.40 | -6.00 | -2.41 |
| 4-Hydroxy-4-methyl-2-pentanone | 120.50 | 221.30 | 255.18 | -33.88 | -15.31 |
| 1-Hexanol | 120.60 | 242.70 | 255.38 | -12.68 | -5.22 |
| 2-Hexanol | 120.60 | 256.31 | 255.38 | 0.93 | 0.36 |
| n-Hexanoic acid | 123.30 | 225.10 | 260.67 | -35.57 | -15.80 |
| s-Phenethyl alcohol | 124.20 | 257.45 | 262.44 | -4.99 | -1.94 |
| Phenethyl alcohol | 124.50 | 252.64 | 263.03 | -10.39 | -4.11 |
| Cyclohexanemethanol | 125.30 | 236.50 | 264.60 | -28.10 | -11.88 |
| 1-Methylcyclohexanol | 125.30 | 279.05 | 264.60 | 14.45 | 5.18 |
| cis-2-Methylcyclohexanol | 125.30 | 268.95 | 264.60 | 4.35 | 1.62 |
| Cycloheptanol | 125.50 | 250.22 | 264.99 | -14.77 | -5.90 |
| cis-3-Methylcyclohexanol | 126.10 | 199.60 | 266.17 | -66.57 | -33.35 |
| trans-4-Methylcyclohexanol | 126.10 | 202.10 | 266.17 | -64.07 | -31.70 |
| trans-2-Methylcyclohexanol | 126.10 | 262.98 | 266.17 | -3.19 | -1.21 |
| 2,4-Dibromophenol | 129.10 | 259.40 | 272.05 | -12.65 | -4.88 |
| 2-t-Butoxyethanol | 129.40 | 273.45 | 272.64 | 0.81 | 0.30 |
| Butylcellosolve | 130.20 | 271.66 | 274.21 | -2.55 | -0.94 |
| 2-Diethylaminoethanol | 130.80 | 281.20 | 275.39 | 5.81 | 2.07 |
| 2-Phenoxyethanol | 134.80 | 294.63 | 283.24 | 11.39 | 3.87 |
| 1-Naphthol | 135.40 | 284.50 | 284.41 | 0.09 | 0.03 |
| Triethylmethanol | 135.50 | 353.90 | 284.61 | 69.29 | 19.58 |
| 2,4-Dimethyl-3-pentanol | 135.90 | 359.00 | 285.39 | 73.61 | 20.50 |
| 2,2-Dimethyl-3-pentanol | 135.90 | 349.00 | 285.39 | 63.61 | 18.23 |
| 2-Methyl-3-hexanol | 136.70 | 324.00 | 286.96 | 37.04 | 11.43 |
| 2-Methyl-2-hexanol | 136.70 | 313.54 | 286.96 | 26.58 | 8.48 |
| 5-Methyl-2-hexanol | 136.80 | 295.20 | 287.16 | 8.04 | 2.72 |
| 3-Heptanol | 137.20 | 314.20 | 287.94 | 26.26 | 8.36 |
| 4-Heptanol | 137.20 | 306.77 | 287.94 | 18.83 | 6.14 |
| 2-Heptanol | 137.20 | 298.63 | 287.94 | 10.69 | 3.58 |
| 1-Heptanol | 137.30 | 274.81 | 288.14 | -13.33 | -4.85 |
| Enanthic acid | 139.80 | 267.31 | 293.04 | -25.73 | -9.63 |
| Hydrocinnamyl alcohol | 141.10 | 280.74 | 295.59 | -14.85 | -5.29 |
| Cyclohexaneethanol | 142.50 | 266.00 | 298.34 | -32.34 | -12.16 |
| 2-Hydroxyethyl-2',2'-dimethylpropionate | 148.90 | 308.00 | 310.90 | -2.90 | -0.94 |
| 4-Ethyl-3-hexanol | 152.20 | 361.26 | 317.37 | 43.89 | 12.15 |
| Ethyl salicylate | 152.70 | 283.07 | 318.35 | -35.28 | -12.46 |
| 4-Methyl-2-heptanol | 152.70 | 312.50 | 318.35 | -5.85 | -1.87 |
| 3-Methyl-4-heptanol | 152.80 | 355.78 | 318.55 | 37.23 | 10.46 |
| 3-Methyl-2-heptanol | 152.80 | 297.50 | 318.55 | -21.05 | -7.07 |
| 4-Methyl-3-heptanol | 152.80 | 309.20 | 318.55 | -9.35 | -3.02 |
| 2,5-Dimethyl-3-hexanol | 152.90 | 374.15 | 318.74 | 55.41 | 14.81 |
| 4-Methyl-4-heptanol | 153.00 | 366.92 | 318.94 | 47.98 | 13.08 |
| 2-Ethyl-1-hexanol | 153.00 | 317.50 | 318.94 | -1.44 | -0.45 |
| 5-Methyl-2-heptanol | 153.10 | 296.20 | 319.14 | -22.94 | -7.74 |
| 2-Methyl-4-heptanol | 153.10 | 331.80 | 319.14 | 12.66 | 3.82 |
| 5-Methyl-1-heptanol | 153.20 | 304.20 | 319.33 | -15.13 | -4.97 |
| 2-Methyl-2-heptanol | 153.40 | 337.60 | 319.72 | 17.88 | 5.29 |
| 6-Methyl-3-heptanol | 153.40 | 310.50 | 319.72 | -9.22 | -2.97 |
| 2-Methyl-1-heptanol | 153.50 | 313.00 | 319.92 | -6.92 | -2.21 |
| 6-Methyl-2-heptanol | 153.50 | 315.10 | 319.92 | -4.82 | -1.53 |
| 1-Octanol | 153.70 | 312.10 | 320.31 | -8.21 | -2.63 |
| 3-Octanol | 153.90 | 338.50 | 320.71 | 17.79 | 5.26 |
| 4-Octanol | 153.90 | 332.09 | 320.71 | 11.38 | 3.43 |
| 2-Octanol | 154.00 | 330.10 | 320.90 | 9.20 | 2.79 |
| N-Methyl-2-hydroxyethylammonium propionate | 154.40 | 328.00 | 321.69 | 6.31 | 1.92 |
| n-Octanoic acid | 156.60 | 304.00 | 326.00 | -22.00 | -7.24 |
| Cyclohexanepropanol | 159.20 | 293.00 | 331.10 | -38.10 | -13.00 |
| 4-Allyl-2-methoxyphenol | 161.80 | 343.10 | 336.20 | 6.90 | 2.01 |
| 2-(2-(2-Methoxyethoxy)ethoxy)ethanol | 166.10 | 357.05 | 344.64 | 12.41 | 3.48 |
| 3,7-Dimethyl-6-octen-1-yn-3-ol | 170.00 | 385.30 | 352.29 | 33.01 | 8.57 |
| 1-Ethyl-3-methylimidazolium hydrogen sulfate | 170.50 | 295.50 | 353.27 | -57.77 | -19.55 |
| 3-Nonanol | 170.60 | 373.63 | 353.47 | 20.16 | 5.40 |
| 5-Nonanol | 170.60 | 370.75 | 353.47 | 17.28 | 4.66 |
| 4-Nonanol | 170.60 | 367.86 | 353.47 | 14.39 | 3.91 |
| 1-Nonanol | 170.70 | 341.00 | 353.66 | -12.66 | -3.71 |
| 2-Nonanol | 170.70 | 356.32 | 353.66 | 2.66 | 0.75 |
| N-Methyl-2-hydroxyethylammonium butanoate | 171.10 | 361.00 | 354.45 | 6.55 | 1.81 |
| trans-Geraniol | 172.60 | 346.10 | 357.39 | -11.29 | -3.26 |
| Pelargonic acid | 173.20 | 362.37 | 358.57 | 3.80 | 1.05 |
| Diethyleneglycol monobutyl ether | 173.30 | 354.89 | 358.76 | -3.87 | -1.09 |
| Linalool | 174.20 | 372.40 | 360.53 | 11.87 | 3.19 |
| beta-Citronellol | 179.30 | 357.90 | 370.54 | -12.64 | -3.53 |
| 1-Ethyl-3-methylimidazolium methylphosphonate | 183.60 | 354.64 | 378.97 | -24.33 | -6.86 |
| 4-Propyl-4-heptanol | 185.70 | 446.60 | 383.09 | 63.51 | 14.22 |
| 3,7-Dimethyl-1-octanol | 186.10 | 367.21 | 383.88 | -16.67 | -4.54 |
| n-Decyl alcohol | 187.00 | 377.00 | 385.64 | -8.64 | -2.29 |
| 5-Decanol | 187.30 | 405.77 | 386.23 | 19.54 | 4.82 |
| N-Methyl-2-hydroxyethylammonium pentanoate | 187.80 | 401.00 | 387.21 | 13.79 | 3.44 |
| n-Decanoic acid | 189.90 | 361.10 | 391.33 | -30.23 | -8.37 |
| 2-(2'-Hydroxyethoxy)ethyl pivalate | 191.90 | 368.83 | 395.25 | -26.42 | -7.16 |
| 1-(2-Hydroxyethyl)-3-methyl-1H-imidazolium 2,2,2-trifluoroacetate | 192.20 | 369.00 | 395.84 | -26.84 | -7.27 |
| 1-Undecanol | 204.10 | 406.34 | 419.19 | -12.85 | -3.16 |
| n-Undecanoic acid | 206.50 | 415.10 | 423.90 | -8.80 | -2.12 |
| 1-Dodecanol | 220.80 | 438.42 | 451.95 | -13.53 | -3.09 |
| 1H,1H-Perfluorooctan-1-ol | 222.70 | 468.60 | 455.68 | 12.92 | 2.76 |
| Lauric acid | 223.20 | 404.28 | 456.66 | -52.38 | -12.96 |
| Tributylmethanol | 235.70 | 548.60 | 481.18 | 67.42 | 12.29 |
| 1-Tridecanol | 237.50 | 476.00 | 484.71 | -8.71 | -1.83 |
| 1H,1H-Perfluorononan-1-ol | 250.30 | 515.90 | 509.82 | 6.08 | 1.18 |
| Pentaethylene glycol monomethyl ether | 252.10 | 537.12 | 513.35 | 23.77 | 4.42 |
| Myristyl alcohol | 254.20 | 505.80 | 517.47 | -11.67 | -2.31 |
| 3,7,11-Trimethyl-1-dodecen-3-ol | 262.20 | 574.50 | 533.17 | 41.33 | 7.19 |
| 1-Pentadecanol | 270.90 | 535.10 | 550.24 | -15.14 | -2.83 |
| 1H,1H-Perfluorodecan-1-ol | 274.50 | 563.90 | 557.30 | 6.60 | 1.17 |
| 1H,1H-Perfluoroundecan-1-ol | 302.10 | 602.40 | 611.44 | -9.04 | -1.50 |
| (9Z)-Octadecenoic acid | 317.00 | 577.00 | 640.67 | -63.67 | -11.04 |
| 1H,1H-Perfluorododecan-1-ol | 328.00 | 645.20 | 662.25 | -17.05 | -2.64 |
| Methyltributylammonium serinate | 331.10 | 635.00 | 668.34 | -33.34 | -5.25 |
| Methyltributylammonium L-threoninate | 338.60 | 753.00 | 683.05 | 69.95 | 9.29 |
| 3,7,11,15-Tetramethyl-1-hexadecyn-3-ol | 340.80 | 712.50 | 687.37 | 25.13 | 3.53 |
| Isophytol | 344.10 | 729.70 | 693.84 | 35.86 | 4.91 |
| Heptylpentaoxyethylene | 352.40 | 722.90 | 710.12 | 12.78 | 1.77 |
| 1H,1H-Perfluorotetradecan-1-ol | 379.80 | 725.30 | 763.88 | -38.58 | -5.32 |
| Tetrabutylphosphonium L-serinate | 389.50 | 748.00 | 782.91 | -34.91 | -4.67 |
