# Supplementary material for: Calculation of the Isobaric Heat Capacities of the Liquid and Solid Phase of Organic Compounds at and around 298.15 K Based on Their “True” Molecular Volume
Source: Molecules. 2019 Apr 24;24(8):1626. doi: 10.3390/molecules24081626 (PMC6514989; doi:10.3390/molecules24081626)
Supplement: Supplementary file 1 [file molecules-24-01626-s001.zip › S08. Experimental and Calculated Cp(sol,298) Data Table to Figure 11.doc]

| **Molecule name** | **Molecular volume** | **Cp(sol,298) exp** | **Cp(sol,298) calc** | **Deviation** | **Dev. in %** |
| --- | --- | --- | --- | --- | --- |
| Urea | 53.90 | 93.80 | 76.50 | 17.30 | 18.45 |
| Chloroethylene | 53.90 | 59.40 | 76.50 | -17.10 | -28.78 |
| Tetrazole | 54.50 | 76.52 | 77.32 | -0.80 | -1.04 |
| 1,2,4-Triazole | 57.90 | 78.70 | 81.97 | -3.27 | -4.16 |
| Acetamide | 58.90 | 86.65 | 83.34 | 3.31 | 3.82 |
| Imidazole | 61.20 | 83.93 | 86.49 | -2.56 | -3.05 |
| 1H-Pyrazole | 62.50 | 81.00 | 88.27 | -7.27 | -8.98 |
| Dicyanomethane | 64.10 | 110.29 | 90.46 | 19.83 | 17.98 |
| Thiourea | 64.30 | 96.90 | 90.74 | 6.16 | 6.36 |
| Propanone | 64.60 | 96.20 | 91.15 | 5.05 | 5.25 |
| Hydrazinecarboxamide | 66.30 | 110.60 | 93.48 | 17.12 | 15.48 |
| Glycine | 68.00 | 99.30 | 95.81 | 3.49 | 3.52 |
| Thioacetamide | 69.20 | 100.30 | 97.45 | 2.85 | 2.84 |
| Acrylamide | 69.70 | 110.58 | 98.13 | 12.45 | 11.26 |
| s-Triazine | 69.90 | 95.57 | 98.41 | -2.84 | -2.97 |
| Methylurea | 70.20 | 114.74 | 98.82 | 15.92 | 13.88 |
| 1,3-Dioxolan-2-one | 72.20 | 117.12 | 101.56 | 15.56 | 13.29 |
| Oxalamide | 73.20 | 110.86 | 102.93 | 7.93 | 7.16 |
| Trifluoroacetamide | 73.30 | 110.00 | 103.06 | 6.94 | 6.31 |
| Dimethylene urethane | 74.30 | 113.80 | 104.43 | 9.37 | 8.23 |
| Pyrazine | 74.30 | 102.35 | 104.43 | -2.08 | -2.03 |
| 2,5-Furandione | 74.70 | 67.40 | 104.98 | -37.58 | -55.76 |
| Diformylhydrazine | 74.80 | 99.12 | 105.12 | -6.00 | -6.05 |
| Hydrazinecarbothioamide | 76.30 | 110.90 | 107.17 | 3.73 | 3.36 |
| Dimethyl sulfone | 78.00 | 125.35 | 109.50 | 15.85 | 12.65 |
| 2-Methylimidazole | 78.00 | 105.09 | 109.50 | -4.41 | -4.20 |
| 2,4-Imidazolidinedione | 78.20 | 117.09 | 109.77 | 7.32 | 6.25 |
| Nitroguanidine | 79.10 | 129.30 | 111.01 | 18.29 | 14.15 |
| 1,3,5-Trioxane | 79.10 | 111.40 | 111.01 | 0.39 | 0.35 |
| 2,5-Pyrrolidinedione | 82.70 | 123.88 | 115.94 | 7.94 | 6.41 |
| Imidodicarbonic diamide | 83.20 | 131.30 | 116.62 | 14.68 | 11.18 |
| Benzene | 83.40 | 97.90 | 116.89 | -18.99 | -19.40 |
| (L)-Alanine | 84.60 | 120.90 | 118.54 | 2.36 | 1.95 |
| beta-Alanine | 84.70 | 116.40 | 118.67 | -2.27 | -1.95 |
| 1,3-Oxazolidine-2-thione | 84.90 | 117.10 | 118.95 | -1.85 | -1.58 |
| Sarcosine | 85.00 | 128.90 | 119.08 | 9.82 | 7.61 |
| Methyl phosphonic dichloride | 85.50 | 131.12 | 119.77 | 11.35 | 8.66 |
| Methacrylamide | 86.00 | 143.80 | 120.45 | 23.35 | 16.23 |
| Methylammonium iodide | 86.90 | 93.93 | 121.69 | -27.76 | -29.55 |
| 1,1,2-Trichloroethane | 87.20 | 143.10 | 122.10 | 21.00 | 14.68 |
| 2-Thioxo-4-imidazolidinone | 87.30 | 137.05 | 122.23 | 14.82 | 10.81 |
| N,N-Dimethylurea | 87.60 | 134.30 | 122.65 | 11.65 | 8.68 |
| N,N'-Dimethylurea | 87.90 | 133.30 | 123.06 | 10.24 | 7.68 |
| 5-Amino-3-methylisoxazole | 88.30 | 149.10 | 123.60 | 25.50 | 17.10 |
| Carbonothioic dihydrazide | 88.40 | 125.30 | 123.74 | 1.56 | 1.24 |
| Propylenecarbonate | 88.50 | 122.51 | 123.88 | -1.37 | -1.12 |
| 3-Amino-5-methylisoxazole | 88.50 | 125.10 | 123.88 | 1.22 | 0.98 |
| Malonamide | 89.30 | 133.38 | 124.97 | 8.41 | 6.30 |
| 2-Aminopyridine | 90.20 | 142.47 | 126.21 | 16.26 | 11.42 |
| Uracil | 90.40 | 120.50 | 126.48 | -5.98 | -4.96 |
| Isobutyramide | 91.50 | 148.10 | 127.99 | 20.11 | 13.58 |
| 1,4-Dioxane-2,5-dione | 91.50 | 133.28 | 127.99 | 5.29 | 3.97 |
| Ethylene oxalate | 91.80 | 141.90 | 128.40 | 13.50 | 9.52 |
| 2,4-Hexadiyne | 91.90 | 133.57 | 128.53 | 5.04 | 3.77 |
| 1,3-Thiazolidine-2-thione | 92.00 | 125.30 | 128.67 | -3.37 | -2.69 |
| 2-Ethylimidazole | 93.30 | 147.08 | 130.45 | 16.63 | 11.31 |
| Cyanuric acid | 93.70 | 133.50 | 131.00 | 2.50 | 1.87 |
| Cytosine | 93.70 | 132.60 | 131.00 | 1.60 | 1.21 |
| 2-Mercapto-2-thiazoline | 94.20 | 125.30 | 131.68 | -6.38 | -5.09 |
| 5-Methylhydantoin | 94.40 | 143.11 | 131.96 | 11.15 | 7.79 |
| 1,4-Benzoquinone | 94.70 | 129.00 | 132.37 | -3.37 | -2.61 |
| Methimazole | 95.30 | 129.80 | 133.19 | -3.39 | -2.61 |
| Glycine anhydride | 96.10 | 134.00 | 134.28 | -0.28 | -0.21 |
| Purine | 96.70 | 106.90 | 135.11 | -28.21 | -26.39 |
| Barbituric acid | 98.20 | 141.10 | 137.16 | 3.94 | 2.79 |
| 1,3-Dibromopropane | 98.30 | 156.10 | 137.30 | 18.80 | 12.05 |
| Norbornene | 98.90 | 129.90 | 138.12 | -8.22 | -6.33 |
| Nortricyclene | 100.90 | 129.00 | 140.86 | -11.86 | -9.19 |
| 2-Aminobutyric acid | 101.00 | 146.40 | 140.99 | 5.41 | 3.69 |
| Methyl methacrylate | 101.20 | 165.30 | 141.27 | 24.03 | 14.54 |
| 4-Aminobutyric acid | 101.40 | 146.40 | 141.54 | 4.86 | 3.32 |
| Carbon tetrabromide | 101.40 | 144.30 | 141.54 | 2.76 | 1.91 |
| Hypoxanthine | 101.60 | 134.50 | 141.82 | -7.32 | -5.44 |
| Iodoform | 101.70 | 157.50 | 141.95 | 15.55 | 9.87 |
| Tetrahydro-2H-1,3-oxazine-2-thione | 102.00 | 139.70 | 142.36 | -2.66 | -1.91 |
| 1H-Benzotriazole | 102.20 | 178.70 | 142.64 | 36.06 | 20.18 |
| (L)-Cysteine | 102.60 | 162.30 | 143.19 | 19.11 | 11.78 |
| 1,3,5,7-Tetroxane | 103.10 | 142.22 | 143.87 | -1.65 | -1.16 |
| Butylene carbonate | 103.20 | 139.34 | 144.01 | -4.67 | -3.35 |
| Benzoxazole | 103.90 | 141.59 | 144.97 | -3.38 | -2.38 |
| Isopropylurea | 104.20 | 170.30 | 145.38 | 24.92 | 14.63 |
| 1,3-Dithiane | 104.70 | 123.84 | 146.06 | -22.22 | -17.94 |
| 1H-Indazole | 104.70 | 128.80 | 146.06 | -17.26 | -13.40 |
| 1,4-Cyclohexanedione | 105.00 | 161.50 | 146.47 | 15.03 | 9.31 |
| 1,1-Diamino-2,2-dinitroethylene | 105.10 | 176.59 | 146.61 | 29.98 | 16.98 |
| 1,2-Diaminobenzene | 105.50 | 150.82 | 147.16 | 3.66 | 2.43 |
| Succindiamide | 105.80 | 158.17 | 147.57 | 10.60 | 6.70 |
| Benzimidazole | 106.00 | 128.80 | 147.84 | -19.04 | -14.78 |
| 1,3-Diaminobenzene | 106.10 | 153.70 | 147.98 | 5.72 | 3.72 |
| 1,3,5-Trithiane | 106.70 | 135.30 | 148.80 | -13.50 | -9.98 |
| 5-Methyluracil | 106.70 | 150.20 | 148.80 | 1.40 | 0.93 |
| Norbornane | 107.30 | 151.00 | 149.62 | 1.38 | 0.91 |
| Proline | 107.50 | 150.40 | 149.90 | 0.50 | 0.34 |
| Iodobenzene | 108.00 | 112.10 | 150.58 | -38.48 | -34.33 |
| Pivalamide | 108.30 | 159.80 | 150.99 | 8.81 | 5.51 |
| Thiobarbituric acid | 108.60 | 137.20 | 151.40 | -14.20 | -10.35 |
| 6-Aminopurine | 109.00 | 147.00 | 151.95 | -4.95 | -3.37 |
| Vitamin B3 | 109.20 | 151.30 | 152.22 | -0.92 | -0.61 |
| 4-Chloroaniline | 109.80 | 151.20 | 153.05 | -1.85 | -1.22 |
| 3-Acetyl-2-oxazolidinone | 110.20 | 164.70 | 153.59 | 11.11 | 6.74 |
| 1,4-Dichlorobenzene | 111.90 | 147.76 | 155.92 | -8.16 | -5.52 |
| p-Toluidine | 112.30 | 165.20 | 156.47 | 8.73 | 5.29 |
| Guanine | 112.60 | 160.70 | 156.88 | 3.82 | 2.38 |
| Asparagine | 113.20 | 159.80 | 157.70 | 2.10 | 1.31 |
| Benzamide | 113.40 | 153.80 | 157.98 | -4.18 | -2.71 |
| 2-Amino-5-nitropyridine | 113.50 | 158.00 | 158.11 | -0.11 | -0.07 |
| Xanthine | 113.60 | 151.34 | 158.25 | -6.91 | -4.57 |
| 2,2-Bis(fluoromethyl)-1,3-difluoropropane | 113.90 | 212.55 | 158.66 | 53.89 | 25.35 |
| DABCO | 114.20 | 153.18 | 159.07 | -5.89 | -3.85 |
| Allantoin | 114.40 | 179.90 | 159.34 | 20.56 | 11.43 |
| epsilon-Caprolactam | 114.40 | 156.78 | 159.34 | -2.56 | -1.64 |
| Glycylglycine | 115.50 | 163.97 | 160.85 | 3.12 | 1.90 |
| Creatine | 116.00 | 184.50 | 161.54 | 22.96 | 12.45 |
| 2-Nitroaniline | 116.40 | 166.00 | 162.08 | 3.92 | 2.36 |
| Thianaphthene | 116.40 | 163.01 | 162.08 | 0.93 | 0.57 |
| Valine | 117.00 | 165.00 | 162.90 | 2.10 | 1.27 |
| Phthalic anhydride | 117.70 | 160.00 | 163.86 | -3.86 | -2.41 |
| 3-Nitroaniline | 118.00 | 158.84 | 164.27 | -5.43 | -3.42 |
| 4-Nitroaniline | 118.10 | 154.20 | 164.41 | -10.21 | -6.62 |
| 5-Aminovaleric acid | 118.10 | 163.70 | 164.41 | -0.71 | -0.43 |
| Phthalonitrile | 118.20 | 161.30 | 164.55 | -3.25 | -2.01 |
| 1,4-Benzenedicarbonitrile | 118.40 | 161.20 | 164.82 | -3.62 | -2.25 |
| 1-Azabicyclo[2.2.2]octane | 118.50 | 169.37 | 164.96 | 4.41 | 2.60 |
| t-Butylurea | 119.80 | 185.50 | 166.74 | 18.76 | 10.11 |
| N,N-Diethylurea | 119.80 | 183.30 | 166.74 | 16.56 | 9.03 |
| 1,4-Dibromobenzene | 120.20 | 145.70 | 167.29 | -21.59 | -14.82 |
| sec-Butylurea | 120.30 | 207.90 | 167.42 | 40.48 | 19.47 |
| 1,3-Benzoxazole-2-thione | 120.30 | 153.10 | 167.42 | -14.32 | -9.36 |
| n-Butylurea | 120.90 | 207.90 | 168.25 | 39.65 | 19.07 |
| 4-Chloronitrobenzene | 120.90 | 169.90 | 168.25 | 1.65 | 0.97 |
| 3-Chloronitrobenzene | 120.90 | 168.20 | 168.25 | -0.05 | -0.03 |
| N,N'-Diethylurea | 121.40 | 219.00 | 168.93 | 50.07 | 22.86 |
| 2-Benzimidazolethiol | 122.00 | 171.23 | 169.75 | 1.48 | 0.86 |
| Uric acid | 122.10 | 166.10 | 169.89 | -3.79 | -2.28 |
| Isoquinoline | 122.20 | 177.68 | 170.03 | 7.65 | 4.31 |
| N-Methylbenzaldehydeimine | 122.50 | 164.45 | 170.44 | -5.99 | -3.64 |
| Glutaramide | 122.50 | 170.21 | 170.44 | -0.23 | -0.13 |
| 2-Thiobenzoxazole | 122.70 | 153.10 | 170.71 | -17.61 | -11.50 |
| Bicyclo[2.2.2]octane | 123.00 | 157.69 | 171.12 | -13.43 | -8.52 |
| Benzimidazole-2-thione | 123.00 | 171.23 | 171.12 | 0.11 | 0.06 |
| 4-Dimethylaminopyridine | 123.10 | 181.32 | 171.26 | 10.06 | 5.55 |
| 2-(Acetylamino)-N-methylethanamide | 123.30 | 177.75 | 171.53 | 6.22 | 3.50 |
| 4-Nitrotoluene | 123.30 | 172.30 | 171.53 | 0.77 | 0.45 |
| Benzenecarbothioamide | 123.50 | 152.80 | 171.81 | -19.01 | -12.44 |
| 2-Aminobenzothiazole | 123.70 | 153.20 | 172.08 | -18.88 | -12.32 |
| 1,3-Dimethyluracil | 124.00 | 170.00 | 172.49 | -2.49 | -1.46 |
| Lactide | 124.20 | 184.30 | 172.76 | 11.54 | 6.26 |
| endo-2-Cyanonorbornane | 124.70 | 193.93 | 173.45 | 20.48 | 10.56 |
| t-Butylacetamide | 124.80 | 190.00 | 173.59 | 16.41 | 8.64 |
| exo-Bicyclo[2.2.1]heptane-2-carbonitrile | 125.00 | 200.00 | 173.86 | 26.14 | 13.07 |
| 1-Norbornyl cyanide | 125.20 | 172.90 | 174.13 | -1.23 | -0.71 |
| N-Methylpivalamide | 125.30 | 183.00 | 174.27 | 8.73 | 4.77 |
| N-Isobutylacetamide | 125.90 | 190.00 | 175.09 | 14.91 | 7.85 |
| 1,3,5-Trichlorobenzene | 126.10 | 158.35 | 175.37 | -17.02 | -10.75 |
| 1-Norbornylisocyanide | 126.20 | 196.70 | 175.50 | 21.20 | 10.78 |
| 1,2-Dinitrobenzene | 126.40 | 195.00 | 175.78 | 19.22 | 9.86 |
| 1,3,5,7,9-Pentoxecane | 126.40 | 171.50 | 175.78 | -4.28 | -2.49 |
| Naphthalene | 126.50 | 168.20 | 175.91 | -7.71 | -4.59 |
| Benzenesulfonamide | 127.70 | 172.91 | 177.56 | -4.65 | -2.69 |
| Hexamethylenetetramine | 128.00 | 152.29 | 177.97 | -25.68 | -16.86 |
| 2-(Dinitromethylene)-1,3-diazacyclopentane | 128.70 | 205.41 | 178.93 | 26.48 | 12.89 |
| Enantholactam | 128.80 | 205.00 | 179.06 | 25.94 | 12.65 |
| Acetanilide | 128.80 | 179.30 | 179.06 | 0.24 | 0.13 |
| 3,4,5,6-Tetrahydrophthalic anhydride | 129.00 | 193.39 | 179.34 | 14.05 | 7.27 |
| Ornithine | 129.00 | 191.20 | 179.34 | 11.86 | 6.20 |
| 1,3-Benzothiazole-2-thione | 129.20 | 157.30 | 179.61 | -22.31 | -14.18 |
| 2-Methylindole | 129.20 | 176.09 | 179.61 | -3.52 | -2.00 |
| Hexachloroethane | 129.30 | 198.24 | 179.75 | 18.49 | 9.33 |
| exo-Dicyclopentadiene | 129.40 | 188.70 | 179.89 | 8.81 | 4.67 |
| 1-Benzopyran-4-one | 129.40 | 176.80 | 179.89 | -3.09 | -1.74 |
| 1,4-Dinitrobenzene | 129.90 | 192.00 | 180.57 | 11.43 | 5.95 |
| 1,3-Dinitrobenzene | 129.90 | 188.30 | 180.57 | 7.73 | 4.11 |
| Dimethyl fumarate | 130.10 | 199.20 | 180.84 | 18.36 | 9.22 |
| 3-Trichlorosilylpropionitrile | 130.60 | 186.10 | 181.53 | 4.57 | 2.46 |
| 2,4-Dichlorobenzaldehyde | 130.70 | 186.50 | 181.67 | 4.83 | 2.59 |
| Glutamine | 131.30 | 184.18 | 182.49 | 1.69 | 0.92 |
| Alanylglycine | 132.00 | 168.00 | 183.45 | -15.45 | -9.19 |
| Leucine | 132.40 | 200.80 | 183.99 | 16.81 | 8.37 |
| 1,4-Diiodobenzene | 132.60 | 160.77 | 184.27 | -23.50 | -14.62 |
| Pyridinium tetrafluoroborate | 132.70 | 209.52 | 184.40 | 25.12 | 11.99 |
| 4-Methyl-2-nitroaniline | 132.70 | 205.40 | 184.40 | 21.00 | 10.22 |
| endo-5-Norbornene-2,3-dicarboxylic anhydride | 132.80 | 183.30 | 184.54 | -1.24 | -0.68 |
| Isoleucine | 133.00 | 188.28 | 184.81 | 3.47 | 1.84 |
| 2-Methyl-4-nitroaniline | 134.00 | 217.10 | 186.18 | 30.92 | 14.24 |
| 1,4-Phenylene diisocyanate | 134.40 | 211.70 | 186.73 | 24.97 | 11.79 |
| cis-Cyclohexane-1,2-dicarboxylic anhydride | 134.50 | 207.40 | 186.87 | 20.53 | 9.90 |
| epsilon-Aminocaproic acid | 134.80 | 175.70 | 187.28 | -11.58 | -6.59 |
| Boratrane | 135.80 | 187.20 | 188.65 | -1.45 | -0.77 |
| (1,1-Dimethylethyl)urea | 136.00 | 185.50 | 188.92 | -3.42 | -1.85 |
| 5-Amino-2-mercaptobenzimidazole | 136.20 | 172.00 | 189.20 | -17.20 | -10.00 |
| Bullvalene | 136.40 | 190.38 | 189.47 | 0.91 | 0.48 |
| 2-Naphthylamine | 138.00 | 125.90 | 191.66 | -65.76 | -52.23 |
| Methyl N-phenylurethane | 138.20 | 203.30 | 191.94 | 11.36 | 5.59 |
| 1,3,5-Tribromobenzene | 138.60 | 161.70 | 192.48 | -30.78 | -19.04 |
| 2-Aminobenzenesulfonamide | 138.70 | 173.92 | 192.62 | -18.70 | -10.75 |
| 4-Aminobenzenesulfonamide | 139.10 | 199.75 | 193.17 | 6.58 | 3.30 |
| Adipamide | 139.20 | 203.50 | 193.30 | 10.20 | 5.01 |
| 2-(Acetylamino)-N-methylpropanamide | 140.00 | 201.30 | 194.40 | 6.90 | 3.43 |
| 1,2,4,5-Tetrachlorobenzene | 140.10 | 202.50 | 194.54 | 7.96 | 3.93 |
| 2-Amino-4-methylbenzothiazole | 140.50 | 182.30 | 195.09 | -12.79 | -7.01 |
| 2-Amino-6-methylbenzothiazole | 140.50 | 197.08 | 195.09 | 1.99 | 1.01 |
| 1,3,5-Trichlorotrifluorobenzene | 140.70 | 197.00 | 195.36 | 1.64 | 0.83 |
| 2-Chloronaphthalene | 140.80 | 235.64 | 195.50 | 40.14 | 17.04 |
| 3-Nitrophthalic anhydride | 140.80 | 224.02 | 195.50 | 28.52 | 12.73 |
| Acenaphthylene | 141.00 | 183.59 | 195.77 | -12.18 | -6.63 |
| 3-Nitrophthalimide | 142.80 | 222.29 | 198.23 | 24.06 | 10.82 |
| 2-Methylnaphthalene | 142.90 | 195.80 | 198.37 | -2.57 | -1.31 |
| 2-Acetylbenzofuran | 143.10 | 192.10 | 198.65 | -6.55 | -3.41 |
| 2,2,3,3-Tetramethylbutane | 143.20 | 232.20 | 198.78 | 33.42 | 14.39 |
| 4-Nitrophthalic anhydride | 143.20 | 175.74 | 198.78 | -23.04 | -13.11 |
| 2-Methylbenzenesulfonamide | 143.70 | 193.48 | 199.47 | -5.99 | -3.09 |
| 4-Methylbenzenesulfonamide | 144.00 | 205.46 | 199.88 | 5.58 | 2.72 |
| DAAF | 144.20 | 215.53 | 200.15 | 15.38 | 7.14 |
| 4-Methyl-4-cyclohexene-1,2-dicarboxylic anhydride | 144.70 | 203.51 | 200.84 | 2.67 | 1.31 |
| Nickelocene | 144.70 | 202.39 | 200.84 | 1.55 | 0.77 |
| 2-(Dinitromethylene)-1,3-diazacyclohexane | 144.90 | 207.76 | 201.11 | 6.65 | 3.20 |
| Ferrocene | 145.00 | 195.90 | 201.25 | -5.35 | -2.73 |
| Methyl 4-methylbenzoate | 145.00 | 199.10 | 201.25 | -2.15 | -1.08 |
| 4-Nitrophthalimide | 145.00 | 199.62 | 201.25 | -1.62 | -0.81 |
| Adamantane | 145.10 | 190.00 | 201.38 | -11.38 | -5.99 |
| 4-Methyl-2-(dinitromethylene)-1,3-diazacyclopentane | 145.20 | 218.89 | 201.52 | 17.37 | 7.93 |
| 4,4'-Bipyridine | 145.30 | 185.60 | 201.66 | -16.06 | -8.65 |
| 1-Ethylpyridinium bromide | 145.70 | 215.30 | 202.21 | 13.09 | 6.08 |
| N-Propylpiperidine | 145.70 | 202.30 | 202.21 | 0.09 | 0.05 |
| Azaferrocene | 146.10 | 183.40 | 202.75 | -19.35 | -10.55 |
| N-(2-Cyanoethyl)aniline | 146.20 | 174.60 | 202.89 | -28.29 | -16.20 |
| 2-Thiaadamantane | 147.00 | 213.00 | 203.99 | 9.01 | 4.23 |
| Acenaphthene | 147.10 | 191.38 | 204.12 | -12.74 | -6.66 |
| 4-Dimethylaminobenzaldehyde | 147.50 | 234.00 | 204.67 | 29.33 | 12.53 |
| 2-Nitrobenzenesulfonamide | 148.00 | 179.54 | 205.36 | -25.82 | -14.38 |
| Alanylalanine | 148.30 | 194.90 | 205.77 | -10.87 | -5.58 |
| Camphene | 148.40 | 235.13 | 205.90 | 29.23 | 12.43 |
| 2,4,6-Trimethoxy-s-triazine | 148.50 | 207.00 | 206.04 | 0.96 | 0.46 |
| 1-Benzylimidazole | 148.90 | 182.10 | 206.59 | -24.49 | -13.45 |
| beta-Alanyl-beta-alanine | 149.00 | 196.00 | 206.72 | -10.72 | -5.47 |
| 1-Benzylpyrazole | 149.10 | 259.61 | 206.86 | 52.75 | 20.32 |
| 2,4,6-Trimethylbenzonitrile | 149.10 | 220.50 | 206.86 | 13.64 | 6.19 |
| 4-Nitrophenetole | 149.20 | 246.00 | 207.00 | 39.00 | 15.85 |
| 2,4,6-Tribromoaniline | 149.20 | 181.40 | 207.00 | -25.60 | -14.11 |
| 1,2,4,5-Tetramethylbenzene | 149.50 | 215.10 | 207.41 | 7.69 | 3.58 |
| Manganocene | 149.50 | 208.15 | 207.41 | 0.74 | 0.36 |
| Pentaerythrityl tetrachloride | 149.60 | 198.30 | 207.55 | -9.25 | -4.66 |
| 2-Mercapto-5-methoxybenzimidazole | 149.70 | 195.40 | 207.68 | -12.28 | -6.29 |
| Terephthaloyl chloride | 149.80 | 207.90 | 207.82 | 0.08 | 0.04 |
| Pyromellitic dianhydride | 150.50 | 219.90 | 208.78 | 11.12 | 5.06 |
| 2,4,6-Trimethylphenyl isocyanide | 150.90 | 241.80 | 209.33 | 32.47 | 13.43 |
| 2-Chloro-5-(trichloromethyl)pyridine | 150.90 | 192.00 | 209.33 | -17.33 | -9.02 |
| 4-Nitrobenzenesulfonamide | 151.00 | 219.98 | 209.46 | 10.52 | 4.78 |
| 3-Nitrobenzenesulfonamide | 151.00 | 199.97 | 209.46 | -9.49 | -4.75 |
| Chloranil | 151.10 | 193.80 | 209.60 | -15.80 | -8.15 |
| 2-Chloro-6-(trichloromethyl)pyridine | 151.40 | 189.35 | 210.01 | -20.66 | -10.91 |
| trans-Decahydroquinoline | 151.60 | 193.79 | 210.29 | -16.50 | -8.51 |
| 2-Ethyl-4-pyridinecarbothioamide | 152.00 | 201.30 | 210.83 | -9.53 | -4.74 |
| 4-Nitroacetanilide | 152.10 | 230.50 | 210.97 | 19.53 | 8.47 |
| Dibenzofuran | 152.60 | 199.01 | 211.65 | -12.64 | -6.35 |
| 1,2,4,5-Benzenetetracarbonitrile | 153.10 | 222.30 | 212.34 | 9.96 | 4.48 |
| 1,3,5-Trinitrobenzene | 153.20 | 222.70 | 212.48 | 10.22 | 4.59 |
| Biphenyl | 153.30 | 198.17 | 212.61 | -14.44 | -7.29 |
| 3,4-Dimethoxybenzaldehyde | 154.10 | 213.50 | 213.71 | -0.21 | -0.10 |
| Camphor | 154.40 | 242.70 | 214.12 | 28.58 | 11.78 |
| Carbazole | 154.50 | 194.00 | 214.26 | -20.26 | -10.44 |
| Ethyl N-phenylcarbamate | 155.00 | 227.20 | 214.94 | 12.26 | 5.40 |
| 1,8-Dimethylnaphthalene | 155.30 | 242.80 | 215.35 | 27.45 | 11.30 |
| Pimelamide | 155.40 | 230.82 | 215.49 | 15.33 | 6.64 |
| 4-Methoxycinnamaldehyde | 155.50 | 217.40 | 215.63 | 1.77 | 0.82 |
| 1,1,3,3-Tetramethyl-1,3-disilacyclobutane | 155.50 | 216.94 | 215.63 | 1.31 | 0.61 |
| Phenylalanine | 155.60 | 203.10 | 215.76 | -12.66 | -6.23 |
| 2-Chloro-N,N-dimethylnicotinamide | 155.90 | 259.40 | 216.17 | 43.23 | 16.66 |
| 2,4,6-Trimethylbenzonitrile N-oxide | 157.50 | 236.10 | 218.36 | 17.74 | 7.51 |
| 2,3,5,6-Tetramethyl-1,4-benzoquinone | 158.20 | 231.54 | 219.32 | 12.22 | 5.28 |
| Fluorene | 158.30 | 203.13 | 219.46 | -16.33 | -8.04 |
| Guajen | 158.70 | 216.47 | 220.01 | -3.54 | -1.63 |
| 2,6-Dimethylnaphthalene | 159.20 | 204.20 | 220.69 | -16.49 | -8.08 |
| 2,7-Dimethylnaphthalene | 159.20 | 204.39 | 220.69 | -16.30 | -7.98 |
| RDX | 159.50 | 248.90 | 221.10 | 27.80 | 11.17 |
| 2-(Dinitromethylene)-1,3-diazacycloheptane | 160.20 | 215.40 | 222.06 | -6.66 | -3.09 |
| Citrulline | 160.40 | 232.60 | 222.34 | 10.26 | 4.41 |
| 1,10-Phenanthroline | 160.80 | 198.74 | 222.88 | -24.14 | -12.15 |
| 2-(Chloromethylthio)benzothiazole | 161.00 | 249.80 | 223.16 | 26.64 | 10.67 |
| Phenazine | 161.00 | 234.30 | 223.16 | 11.14 | 4.76 |
| Dibenzo[b,d]thiophene | 161.00 | 223.73 | 223.16 | 0.57 | 0.26 |
| N-Butylpiperidine | 162.40 | 242.97 | 225.07 | 17.90 | 7.37 |
| 1-Cyanoadamantane | 162.80 | 218.60 | 225.62 | -7.02 | -3.21 |
| (L)-Arginine | 162.90 | 232.00 | 225.76 | 6.24 | 2.69 |
| 3-Cyano-4-methoxymethyl-6-methyl-(2-pyridone) | 163.00 | 211.76 | 225.90 | -14.14 | -6.68 |
| Diphenyl ether | 163.00 | 216.56 | 225.90 | -9.34 | -4.31 |
| Pentamethylbenzene | 163.10 | 210.50 | 226.03 | -15.53 | -7.38 |
| 1,3-Diethylbarbituric acid | 163.20 | 248.50 | 226.17 | 22.33 | 8.99 |
| 1-Bromoadamantane | 163.30 | 215.59 | 226.31 | -10.72 | -4.97 |
| 4,4'-Difluorobiphenyl | 163.40 | 222.77 | 226.44 | -3.67 | -1.65 |
| 1-Adamantyl isocyanide | 163.90 | 215.20 | 227.13 | -11.93 | -5.54 |
| Dibenzo-p-dioxin | 164.10 | 206.70 | 227.40 | -20.70 | -10.02 |
| Barbital | 164.30 | 228.70 | 227.68 | 1.02 | 0.45 |
| Phenanthridine | 164.60 | 201.87 | 228.09 | -26.22 | -12.99 |
| 2-Phenylaniline | 164.80 | 221.86 | 228.36 | -6.50 | -2.93 |
| Benzo[h]quinoline | 165.20 | 206.36 | 228.91 | -22.55 | -10.93 |
| Dibenzo[b,e]pyridine | 165.40 | 205.07 | 229.18 | -24.11 | -11.76 |
| AIBN | 165.50 | 237.55 | 229.32 | 8.23 | 3.46 |
| Pentaerythritol tetrabromide | 165.60 | 218.57 | 229.46 | -10.89 | -4.98 |
| 5-Methyl-5-phenylhydantoin | 165.60 | 235.85 | 229.46 | 6.39 | 2.71 |
| 2,4,6-Trinitrotoluene | 166.20 | 243.30 | 230.28 | 13.02 | 5.35 |
| Caffeine | 167.10 | 232.00 | 231.51 | 0.49 | 0.21 |
| Hexachlorobenzene | 167.20 | 201.29 | 231.65 | -30.36 | -15.08 |
| 2-Chlorobiphenyl | 167.60 | 211.92 | 232.20 | -20.28 | -9.57 |
| 4-Chlorobiphenyl | 167.80 | 243.76 | 232.47 | 11.29 | 4.63 |
| Bicyclo[3.3.3]undecane | 168.20 | 213.20 | 233.02 | -19.82 | -9.29 |
| 8-Aminocaprylic acid | 168.20 | 251.70 | 233.02 | 18.68 | 7.42 |
| 1,8-Dinitronaphthalene | 168.30 | 254.40 | 233.15 | 21.25 | 8.35 |
| Phenanthrene | 168.90 | 220.30 | 233.98 | -13.68 | -6.21 |
| 3,4-Dimethoxyphenylacetonitrile | 168.90 | 243.50 | 233.98 | 9.52 | 3.91 |
| 1,5-Dinitronaphthalene | 169.50 | 259.40 | 234.80 | 24.60 | 9.48 |
| 3-Dimethylaminomethylindole | 169.70 | 283.70 | 235.07 | 48.63 | 17.14 |
| Anthracene | 169.70 | 210.50 | 235.07 | -24.57 | -11.67 |
| 4-Phenyltoluene | 169.90 | 238.74 | 235.34 | 3.40 | 1.42 |
| Dimethylterephthalate | 170.10 | 261.10 | 235.62 | 25.48 | 9.76 |
| Cyclohexylbenzene | 170.50 | 263.17 | 236.17 | 27.00 | 10.26 |
| Diphenyl sulfide | 170.60 | 239.70 | 236.30 | 3.40 | 1.42 |
| Diphenylmethane | 170.60 | 233.50 | 236.30 | -2.80 | -1.20 |
| N-Methylcarbazole | 170.80 | 217.84 | 236.58 | -18.74 | -8.60 |
| Benzophenone | 170.90 | 224.80 | 236.71 | -11.91 | -5.30 |
| Suberamide | 171.60 | 245.33 | 237.67 | 7.66 | 3.12 |
| Antipyrine | 171.70 | 268.20 | 237.81 | 30.39 | 11.33 |
| 2-Phenylbenzimidazole | 171.90 | 208.09 | 238.08 | -29.99 | -14.41 |
| Monuron | 172.00 | 256.00 | 238.22 | 17.78 | 6.95 |
| 1,3-Diethyl-2-thiobarbituric acid | 172.70 | 236.90 | 239.18 | -2.28 | -0.96 |
| N-Acetylnorvaline-N'-methylamide | 172.80 | 239.69 | 239.32 | 0.37 | 0.16 |
| Xanthone | 172.90 | 226.00 | 239.45 | -13.45 | -5.95 |
| Phenoxathiin | 173.10 | 213.77 | 239.73 | -25.96 | -12.14 |
| 1,2,3-Trichloro-4,5,6-trimethylbenzene | 173.30 | 252.34 | 240.00 | 12.34 | 4.89 |
| trans-Azobenzene | 173.40 | 229.00 | 240.14 | -11.14 | -4.86 |
| Propyl phenylcarbamate | 173.60 | 263.60 | 240.41 | 23.19 | 8.80 |
| 2-Methyl-3-amino-4-methoxymethyl-5-aminomethylpyridine | 173.70 | 307.10 | 240.55 | 66.55 | 21.67 |
| BAFF | 173.80 | 257.90 | 240.69 | 17.21 | 6.67 |
| 5-Trimethylsilyl-2-norbornene | 174.60 | 241.40 | 241.78 | -0.38 | -0.16 |
| 1-Adamantylcarboxamide | 175.20 | 220.50 | 242.60 | -22.10 | -10.02 |
| 9,10-Dihydrophenanthrene | 175.90 | 243.80 | 243.56 | 0.24 | 0.10 |
| Pentamethylchlorobenzene | 176.70 | 244.80 | 244.66 | 0.14 | 0.06 |
| Benzalaniline | 176.80 | 302.67 | 244.79 | 57.88 | 19.12 |
| 9,10-Dihydroanthracene | 176.90 | 219.06 | 244.93 | -25.87 | -11.81 |
| Naphthalene-1,5-diisocyanate | 177.20 | 223.40 | 245.34 | -21.94 | -9.82 |
| Tolan | 178.00 | 225.90 | 246.44 | -20.54 | -9.09 |
| Diphenylsulfoxide | 178.30 | 239.70 | 246.85 | -7.15 | -2.98 |
| Alanylglycylglycine | 179.40 | 236.00 | 248.35 | -12.35 | -5.23 |
| 1-Acetyladamantane | 179.90 | 217.50 | 249.04 | -31.54 | -14.50 |
| Hexamethylbenzene | 180.00 | 245.60 | 249.18 | -3.58 | -1.46 |
| trans-Stilbene | 180.80 | 235.00 | 250.27 | -15.27 | -6.50 |
| 1,4-Anthraquinone | 181.60 | 223.34 | 251.37 | -28.03 | -12.55 |
| Tetrahydroanthracene | 181.60 | 247.02 | 251.37 | -4.35 | -1.76 |
| 9,10-Anthraquinone | 181.70 | 240.20 | 251.50 | -11.30 | -4.71 |
| Alanylvaline | 182.00 | 240.00 | 251.91 | -11.91 | -4.96 |
| 4-Aminoantipyrine | 182.10 | 294.60 | 252.05 | 42.55 | 14.44 |
| Tryptophane | 182.20 | 238.15 | 252.19 | -14.04 | -5.89 |
| 4-Methylphenanthrene | 182.20 | 262.80 | 252.19 | 10.61 | 4.04 |
| 1,2,3,4-Tetrahydro-N-methylcarbazole | 182.30 | 241.35 | 252.33 | -10.98 | -4.55 |
| Thianthrene | 182.70 | 224.97 | 252.87 | -27.90 | -12.40 |
| Acetylferrocene | 183.30 | 246.00 | 253.69 | -7.69 | -3.13 |
| Fluoranthene | 183.40 | 230.25 | 253.83 | -23.58 | -10.24 |
| 9-Ethyl-9H-carbazole | 184.10 | 238.20 | 254.79 | -16.59 | -6.96 |
| Pyrene | 185.50 | 229.70 | 256.71 | -27.01 | -11.76 |
| Bis(4-aminophenyl) ether | 185.60 | 278.20 | 256.84 | 21.36 | 7.68 |
| 1,3-Di-t-butyl urea | 185.80 | 295.60 | 257.12 | 38.48 | 13.02 |
| 1,2-Diphenylethane | 186.10 | 253.76 | 257.53 | -3.77 | -1.49 |
| 4,4'-Dimethylbiphenyl | 186.20 | 242.26 | 257.67 | -15.41 | -6.36 |
| Octyl urea | 187.70 | 278.70 | 259.72 | 18.98 | 6.81 |
| Azelamide | 188.30 | 270.30 | 260.54 | 9.76 | 3.61 |
| Diphenylsulfone | 188.40 | 244.30 | 260.68 | -16.38 | -6.70 |
| [2.2]Paracyclophane | 189.10 | 252.70 | 261.64 | -8.94 | -3.54 |
| Pentaerythrityl tetraiodide | 189.40 | 209.60 | 262.05 | -52.45 | -25.02 |
| Diphenylcarbonate | 189.40 | 263.13 | 262.05 | 1.08 | 0.41 |
| Diamantane | 189.60 | 223.40 | 262.32 | -38.92 | -17.42 |
| 1-Methoxycarbonyladamantane | 190.00 | 264.20 | 262.87 | 1.33 | 0.50 |
| Benzil | 190.40 | 245.70 | 263.42 | -17.72 | -7.21 |
| 2-Benzylbenzimidazole | 190.80 | 228.92 | 263.96 | -35.04 | -15.31 |
| Diphenyldisulfide | 190.90 | 241.70 | 264.10 | -22.40 | -9.27 |
| Dimethyl cubane-1,4-dicarboxylate | 191.90 | 251.10 | 265.47 | -14.37 | -5.72 |
| 1-Benzyl-3-methylimidazolium chloride | 192.00 | 302.40 | 265.61 | 36.79 | 12.17 |
| 1,2,3,4,5,6,7,8-Octahydroanthracene | 193.10 | 277.96 | 267.11 | 10.85 | 3.90 |
| [2,2]Metaparacyclophane | 193.10 | 261.50 | 267.11 | -5.61 | -2.15 |
| 2,9-Dimethyl-1,10-phenanthroline | 193.20 | 253.00 | 267.25 | -14.25 | -5.63 |
| 4,4-Methylenedianiline | 193.90 | 270.90 | 268.21 | 2.69 | 0.99 |
| (L)-Cystine | 194.50 | 261.92 | 269.03 | -7.11 | -2.72 |
| BNFF | 197.70 | 308.50 | 273.41 | 35.09 | 11.37 |
| 4,5,9,10-Tetrahydropyrene | 197.70 | 257.12 | 273.41 | -16.29 | -6.34 |
| Alanylnorleucine | 198.30 | 276.00 | 274.24 | 1.76 | 0.64 |
| Tetramethylthiuram disulfide | 200.00 | 301.70 | 276.56 | 25.14 | 8.33 |
| Tetryl | 202.30 | 290.90 | 279.71 | 11.19 | 3.85 |
| [2,2]Metacyclophane | 203.10 | 240.60 | 280.81 | -40.21 | -16.71 |
| 1,2,3,6,7,8-Hexahydropyrene | 203.10 | 255.54 | 280.81 | -25.27 | -9.89 |
| 9-Methoxy-1,4-anthraquinone | 203.30 | 269.21 | 281.08 | -11.87 | -4.41 |
| Perfluorobiphenyl | 203.50 | 317.40 | 281.36 | 36.04 | 11.36 |
| Sebacamide | 206.00 | 289.66 | 284.78 | 4.88 | 1.68 |
| 3,3',4,4'-Tetraaminodiphenyl ether | 206.20 | 319.70 | 285.05 | 34.65 | 10.84 |
| 4'-Bromomethyl-2-cyanobiphenyl | 206.50 | 257.00 | 285.46 | -28.46 | -11.08 |
| Isoproturon | 209.40 | 293.28 | 289.44 | 3.84 | 1.31 |
| cis-5a,6,11a,12-Tetrahdro[1,4]benzoxazino[3,2-b]-[1,4]-benzoxazine | 209.50 | 290.71 | 289.57 | 1.14 | 0.39 |
| HMX | 210.40 | 293.00 | 290.80 | 2.20 | 0.75 |
| Triphenylene | 210.60 | 259.20 | 291.08 | -31.88 | -12.30 |
| 4,4'-Diaminodiphenyl sulfone | 211.20 | 314.90 | 291.90 | 23.00 | 7.30 |
| Perhydrophenanthrene | 211.20 | 289.50 | 291.90 | -2.40 | -0.83 |
| 6-Aminoflavone | 211.30 | 277.90 | 292.04 | -14.14 | -5.09 |
| Benz[a]anthracene | 212.20 | 273.60 | 293.27 | -19.67 | -7.19 |
| Tetracene | 212.90 | 260.50 | 294.23 | -33.73 | -12.95 |
| 1,4-Di-t-butylbenzene | 213.60 | 323.15 | 295.19 | 27.96 | 8.65 |
| 4,4'-Diaminodiphenyldisulfide | 213.70 | 294.00 | 295.32 | -1.32 | -0.45 |
| Methyl tetryl | 215.00 | 326.40 | 297.10 | 29.30 | 8.98 |
| 4,4'-Dichlorodiphenyl sulphone | 216.90 | 269.30 | 299.71 | -30.41 | -11.29 |
| Dimethyl-2,6-naphthalinedicarboxylate | 217.40 | 283.80 | 300.39 | -16.59 | -5.85 |
| 5,5-Diphenylhydantoin | 217.60 | 287.59 | 300.66 | -13.07 | -4.55 |
| Tri-t-butylmethane | 217.70 | 354.80 | 300.80 | 54.00 | 15.22 |
| 4-t-Butylbiphenyl | 218.20 | 304.73 | 301.49 | 3.24 | 1.06 |
| 1,3,5,7-Tetramethyl-2,4,6,8-tetrathiaadamantane | 218.30 | 295.85 | 301.62 | -5.77 | -1.95 |
| 1,1'-Diacetylferrocene | 218.50 | 293.90 | 301.90 | -8.00 | -2.72 |
| trans-1,2-Dibenzoylethylene | 219.80 | 275.30 | 303.68 | -28.38 | -10.31 |
| anti-trans-Truxane | 219.90 | 275.30 | 303.81 | -28.51 | -10.36 |
| 4'-Propylbiphenyl-4-carbonitrile | 220.00 | 302.97 | 303.95 | -0.98 | -0.32 |
| t-Butyl 1-phenylethylcarbamate | 220.70 | 313.81 | 304.91 | 8.90 | 2.84 |
| 4-Trichlorosilylbiphenyl | 221.30 | 291.20 | 305.73 | -14.53 | -4.99 |
| Undecanediamide | 221.70 | 314.53 | 306.28 | 8.25 | 2.62 |
| 4,4'-Diisocyanatodiphenylmethane | 221.70 | 307.00 | 306.28 | 0.72 | 0.24 |
| Hexyl phenylcarbamate | 222.50 | 336.80 | 307.37 | 29.43 | 8.74 |
| 1,3,5,7-Tetramethyl-2,4,6,8,9,10-hexathiaadamantane | 222.60 | 301.62 | 307.51 | -5.89 | -1.95 |
| 1-Benzyl-3-methylimidazolium tetrafluoroborate | 222.90 | 305.40 | 307.92 | -2.52 | -0.83 |
| p-Terphenyl | 223.20 | 279.60 | 308.33 | -28.73 | -10.28 |
| m-Terphenyl | 223.80 | 281.00 | 309.15 | -28.15 | -10.02 |
| o-Terphenyl | 224.00 | 274.75 | 309.43 | -34.68 | -12.62 |
| 1,2-Dibenzoylethane | 225.60 | 291.20 | 311.62 | -20.42 | -7.01 |
| Perylene | 227.00 | 274.90 | 313.54 | -38.64 | -14.05 |
| 2,2'-Diindanyl | 227.00 | 332.60 | 313.54 | 19.06 | 5.73 |
| 4-t-Butyldiphenyl ether | 227.80 | 304.06 | 314.63 | -10.57 | -3.48 |
| Diethynyldiphenylsilane | 229.00 | 305.20 | 316.27 | -11.07 | -3.63 |
| cis-5a,6,11a,12-Tetrahdro[1,4]benzothiazino[3,2-b]-[1,4]-benzothiazine | 229.40 | 296.89 | 316.82 | -19.93 | -6.71 |
| Triptycene | 231.90 | 283.10 | 320.25 | -37.15 | -13.12 |
| Benzylferrocene | 232.50 | 312.60 | 321.07 | -8.47 | -2.71 |
| 1-Methyl-7-isopropylphenanthrene | 233.30 | 294.60 | 322.16 | -27.56 | -9.36 |
| [3,3]Paracyclophane | 233.50 | 324.30 | 322.44 | 1.86 | 0.57 |
| Benzoylferrocene | 234.80 | 313.60 | 324.22 | -10.62 | -3.39 |
| Triphenylamine | 235.10 | 297.50 | 324.63 | -27.13 | -9.12 |
| 3,4,4'-Trichlorocarbanilide | 236.70 | 284.13 | 326.82 | -42.69 | -15.02 |
| Dodecanediamide | 238.40 | 327.84 | 329.15 | -1.31 | -0.40 |
| Tritane | 241.20 | 308.80 | 332.98 | -24.18 | -7.83 |
| 3-Phenyl-5-phenoxymethyl-2-oxazolidinone | 242.40 | 310.60 | 334.62 | -24.02 | -7.73 |
| N-Cyclohexyl-2,4,6-trimethylbenzaldehyde imine | 242.60 | 394.51 | 334.90 | 59.61 | 15.11 |
| Triphenylphosphane | 244.00 | 312.50 | 336.82 | -24.32 | -7.78 |
| 1,2-Diphenylbenzimidazole | 247.60 | 318.70 | 341.75 | -23.05 | -7.23 |
| Dicumyl | 247.80 | 321.10 | 342.02 | -20.92 | -6.51 |
| Tetracyclopropylsuccinonitrile | 248.10 | 321.80 | 342.43 | -20.63 | -6.41 |
| 4-Nitro-4'-t-butyl-diphenylether | 251.00 | 359.52 | 346.40 | 13.12 | 3.65 |
| Triphenylethylene | 252.00 | 309.20 | 347.77 | -38.57 | -12.47 |
| Triphenylmethyl chloride | 253.80 | 367.27 | 350.24 | 17.03 | 4.64 |
| 1,1,1-Triphenylethane | 255.20 | 316.70 | 352.15 | -35.45 | -11.19 |
| 2,2-Bis(4-cyanatophenyl)propane | 255.20 | 355.64 | 352.15 | 3.49 | 0.98 |
| 1,1,2-Triphenylethane | 256.00 | 319.70 | 353.25 | -33.55 | -10.49 |
| 1,2'-Dinaphthylmethane | 256.10 | 314.60 | 353.39 | -38.79 | -12.33 |
| Pentacene | 256.30 | 311.50 | 353.66 | -42.16 | -13.53 |
| TPPPO | 256.50 | 317.00 | 353.93 | -36.93 | -11.65 |
| Myclobutanil | 257.10 | 362.78 | 354.75 | 8.03 | 2.21 |
| Coronene | 259.80 | 317.17 | 358.45 | -41.28 | -13.02 |
| 2-Pentadecanone | 264.00 | 426.80 | 364.20 | 62.60 | 14.67 |
| N-Benzoyl-o-aminodiphenylamine | 264.80 | 356.60 | 365.30 | -8.70 | -2.44 |
| Tetraisopropoxysilane | 265.60 | 359.48 | 366.39 | -6.91 | -1.92 |
| Chlorotriphenylsilane | 267.20 | 337.60 | 368.59 | -30.99 | -9.18 |
| Tetrapropyl silicate | 268.40 | 363.39 | 370.23 | -6.84 | -1.88 |
| 1-Benzyl-3-methylimidazolium tetrafluoroethanesulfonate | 269.70 | 430.80 | 372.01 | 58.79 | 13.65 |
| Tetradecanediamide | 272.90 | 383.31 | 376.39 | 6.92 | 1.81 |
| N-Adamantan-1-yl-4-bromobenzenesulfonamide | 276.60 | 396.60 | 381.46 | 15.14 | 3.82 |
| Cetane | 278.20 | 441.80 | 383.65 | 58.15 | 13.16 |
| N-Adamantan-1-yl-4-nitrobenzenesulfonamide | 281.50 | 420.30 | 388.17 | 32.13 | 7.65 |
| 4,4'-Di-t-butylbiphenyl | 282.80 | 412.41 | 389.95 | 22.46 | 5.45 |
| 2,4,6-Triphenyltriazine | 283.90 | 345.10 | 391.45 | -46.35 | -13.43 |
| N-(4-Ethoxybenzylidene)-4-butylaniline | 285.40 | 429.09 | 393.51 | 35.58 | 8.29 |
| 4-Hexyloxybenzylideneaniline | 286.60 | 404.52 | 395.15 | 9.37 | 2.32 |
| N-Adamantan-1-yl-4-trifluoromethylbenzenesulfonamide | 289.50 | 418.10 | 399.12 | 18.98 | 4.54 |
| 2,4,6-Triphenylpyridine | 290.60 | 358.60 | 400.63 | -42.03 | -11.72 |
| Hexacyclopropylethane | 291.40 | 396.10 | 401.72 | -5.62 | -1.42 |
| N-(3,5-Dimethyladamantan-1-yl)-benzenesulfonamide | 291.70 | 357.80 | 402.13 | -44.33 | -12.39 |
| 4-Hexyloxybenzylideneamino-4'-fluorobenzene | 291.80 | 442.55 | 402.27 | 40.28 | 9.10 |
| 1,1'-Diphenyl-1,1'-bicyclopentyl | 291.80 | 375.50 | 402.27 | -26.77 | -7.13 |
| 1,2,3-Triphenylbenzene | 292.80 | 352.37 | 403.64 | -51.27 | -14.55 |
| m-Quaterphenyl | 293.90 | 359.50 | 405.15 | -45.65 | -12.70 |
| 1,3,5-Triphenylbenzene | 294.00 | 361.00 | 405.28 | -44.28 | -12.27 |
| p-Quaterphenyl | 294.00 | 363.50 | 405.28 | -41.78 | -11.50 |
| Perchlorobiphenyl | 295.20 | 344.00 | 406.93 | -62.93 | -18.29 |
| o-Quaterphenyl | 295.20 | 359.10 | 406.93 | -47.83 | -13.32 |
| 4-Hexyloxybenzylideneamino-4'-chlorobenzene | 301.10 | 434.94 | 415.01 | 19.93 | 4.58 |
| 4-Hexyloxybenzylidene-4'-toluidine | 303.20 | 441.40 | 417.88 | 23.52 | 5.33 |
| N-(4-Hexyloxybenzylidene)-4-aminobenzonitrile | 304.30 | 432.51 | 419.39 | 13.12 | 3.03 |
| cis-(5,12)-7,7,14,14-Hexamethyl-1,4,8,11-tetraazacyclotetradecane | 306.70 | 444.80 | 422.68 | 22.12 | 4.97 |
| Methyl hexadecanoate | 307.00 | 474.47 | 423.09 | 51.38 | 10.83 |
| Tetraphenylmethane | 307.60 | 368.20 | 423.91 | -55.71 | -15.13 |
| N-(3,5-Dimethyladamantan-1-yl)-4-bromobenzenesulfonamide | 310.20 | 458.60 | 427.47 | 31.13 | 6.79 |
| Octadecane | 311.60 | 485.64 | 429.39 | 56.25 | 11.58 |
| N-(3,5-Dimethyladamantan-1-yl)-4-nitrobenzenesulfonamide | 315.00 | 482.30 | 434.04 | 48.26 | 10.01 |
| 3-Phenyl-5-phenoxymethyl-2-N-phenyliminooxazolidine | 316.50 | 400.20 | 436.10 | -35.90 | -8.97 |
| p-Diacetylbenzene diethyl ketal | 319.30 | 462.00 | 439.93 | 22.07 | 4.78 |
| 1,1,1,2-Tetraphenylethane | 319.80 | 395.40 | 440.61 | -45.21 | -11.44 |
| 1,1'-Diphenyl-1,1'-bicyclohexyl | 321.50 | 403.80 | 442.94 | -39.14 | -9.69 |
| N-(3,5-Dimethyladamantan-1-yl)-4-trifluoromethylbenzenesulfonamide | 322.50 | 480.10 | 444.31 | 35.79 | 7.45 |
| 1,1,2,2-Tetraphenylethane | 322.80 | 399.60 | 444.72 | -45.12 | -11.29 |
| Tetraphenylethylene | 323.40 | 388.70 | 445.54 | -56.84 | -14.62 |
| Potassium tetraphenyl boride | 323.50 | 418.23 | 445.68 | -27.45 | -6.56 |
| Bisphenol A diglycidyl ether | 324.80 | 485.90 | 447.46 | 38.44 | 7.91 |
| Tetraphenylsilane | 325.80 | 395.80 | 448.83 | -53.03 | -13.40 |
| 1,1'-Dibenzoylferrocene | 327.60 | 466.10 | 451.30 | 14.80 | 3.18 |
| Tetra-2-butoxysilane | 329.70 | 487.27 | 454.17 | 33.10 | 6.79 |
| Dibenzo-18-crown-6 | 333.20 | 439.70 | 458.96 | -19.26 | -4.38 |
| Tetrabutylammonium iodide | 333.20 | 442.50 | 458.96 | -16.46 | -3.72 |
| Tetra-2-methyl-1-propoxysilane | 334.30 | 477.59 | 460.47 | 17.12 | 3.58 |
| N,N'-Bis(p-methoxyphenyl)terephthalamide | 334.80 | 467.80 | 461.15 | 6.65 | 1.42 |
| N,N'-Bis-(m-methoxyphenyl)terephtalamide | 334.80 | 458.10 | 461.15 | -3.05 | -0.67 |
| Tetrabutoxysilane | 335.20 | 493.68 | 461.70 | 31.98 | 6.48 |
| N-(4-Pentyloxybenzylidene)-4-butylaniline | 336.30 | 478.20 | 463.21 | 14.99 | 3.13 |
| Ammonium tetraphenyl boride | 336.90 | 434.93 | 464.03 | -29.10 | -6.69 |
| Eicosane | 345.50 | 479.90 | 475.81 | 4.09 | 0.85 |
| 4-Nitrophenyl-4'-octyloxybenzoate | 347.70 | 510.00 | 478.82 | 31.18 | 6.11 |
| Di(4'-methoxyphenyl)cyclohexane-1,4-dicarboxylate | 348.10 | 478.60 | 479.37 | -0.77 | -0.16 |
| N-(4-Hexyloxybenzylidene)-4-butylaniline | 352.20 | 512.02 | 484.98 | 27.04 | 5.28 |
| 2,4,6-Trimethyl-2,4,6-triphenylcyclotrisiloxane | 356.50 | 506.70 | 490.87 | 15.83 | 3.12 |
| Bis-(o-aminophenyl)-2,2'-dibenzimidazole oxide | 359.10 | 471.10 | 494.43 | -23.33 | -4.95 |
| o-Quinquephenyl | 363.90 | 444.30 | 501.00 | -56.70 | -12.76 |
| p-Quinquephenyl | 364.10 | 455.50 | 501.28 | -45.78 | -10.05 |
| m-Quinquephenyl | 364.20 | 443.70 | 501.41 | -57.71 | -13.01 |
| Risperidal | 367.40 | 502.00 | 505.80 | -3.80 | -0.76 |
| 2,3-Dimethyl-2,3-bis(4-t-butylphenyl)butane | 377.00 | 529.50 | 518.94 | 10.56 | 1.99 |
| Docosane | 378.90 | 563.60 | 521.54 | 42.06 | 7.46 |
| 1,1,3,3-Tetraphenyl-1,3-dimethyldisiloxane | 386.00 | 503.20 | 531.27 | -28.07 | -5.58 |
| 1,4-Bis(diphenylamino)benzene | 386.40 | 470.60 | 531.81 | -61.21 | -13.01 |
| 2,11-Dicyclohexyldodecane | 386.60 | 557.30 | 532.09 | 25.21 | 4.52 |
| 1,1-Dicyclohexyldodecane | 387.10 | 562.60 | 532.77 | 29.83 | 5.30 |
| Pentaphenylethane | 388.90 | 473.60 | 535.24 | -61.64 | -13.01 |
| Tetra-2-pentoxysilane | 396.10 | 610.23 | 545.10 | 65.13 | 10.67 |
| Tetra-3-methyl-1-butoxysilane | 399.90 | 591.28 | 550.30 | 40.98 | 6.93 |
| Tetra-1-pentoxysilane | 402.00 | 621.56 | 553.18 | 68.38 | 11.00 |
| 3,4-Dimethyl-3,4-bis(4-t-butylphenyl)hexane | 408.90 | 587.90 | 562.63 | 25.27 | 4.30 |
| Dibenzo-24-crown-8-ether | 411.90 | 577.00 | 566.73 | 10.27 | 1.78 |
| N-Formylmethionine-leucyl-phenylalanine methyl ether | 418.10 | 597.60 | 575.22 | 22.38 | 3.74 |
| Buckminsterfullerene | 423.30 | 536.90 | 582.34 | -45.44 | -8.46 |
| 4,4'-Bis-(N-carbazolyl) -1,1'-biphenyl | 433.80 | 539.36 | 596.72 | -57.36 | -10.64 |
| 3,4-Diethyl-3,4-bis(4-t-butylphenyl)-hexane | 437.40 | 631.30 | 601.65 | 29.65 | 4.70 |
| Hexacosane | 445.70 | 661.20 | 613.02 | 48.18 | 7.29 |
| Tetramethyltetraphenylcyclotetrasiloxane | 465.80 | 615.00 | 640.54 | -25.54 | -4.15 |
| 4,5-Diethyl-4,5-bis-(4-tert-butylphenyl)-octane | 474.50 | 618.50 | 652.46 | -33.96 | -5.49 |
| 2,4,5,7-Tetramethyl-4,5-bis(4-t-butylphenyl)octane | 474.80 | 683.90 | 652.87 | 31.03 | 4.54 |
| Hexaphenyldisiloxane | 496.00 | 681.00 | 681.90 | -0.90 | -0.13 |
| Fullerene C70 | 499.70 | 664.21 | 686.97 | -22.76 | -3.43 |
| 4,5-Dipropyl-4,5-bis-(4-t-butylphenyl)octane | 502.40 | 724.20 | 690.66 | 33.54 | 4.63 |
| Dibenzo-30-crown-10 | 506.00 | 680.40 | 695.59 | -15.19 | -2.23 |
| 2,3,6-Tris(phenylimino)-1,3,5-triphenyl-1,3,5-triazacyclohexane | 519.40 | 672.40 | 713.94 | -41.54 | -6.18 |
| Hexaphenylmelamine | 524.90 | 665.80 | 721.47 | -55.67 | -8.36 |
| Hexaphenylcyclotrisiloxane | 526.10 | 683.70 | 723.12 | -39.42 | -5.77 |
| Dotriacontane | 545.90 | 806.00 | 750.23 | 55.77 | 6.92 |
| 5,6-Dibutyl-5,6-bis(4-t-butylphenyl)decane | 566.20 | 805.50 | 778.03 | 27.47 | 3.41 |
| 1,1,3,3,5,5-Hexaphenyl-7,7-dimethylcyclotetrasiloxane | 584.00 | 815.50 | 802.40 | 13.10 | 1.61 |
| Tetra-2-ethyl-1-hexoxysilane | 595.20 | 870.88 | 817.74 | 53.14 | 6.10 |
| Tetrabutylammonium tetraphenylborate | 602.40 | 840.70 | 827.60 | 13.10 | 1.56 |
| Hexatriacontane | 612.70 | 881.00 | 841.70 | 39.30 | 4.46 |
| Octaphenyltetracyclosiloxane | 685.30 | 932.50 | 941.12 | -8.62 | -0.92 |
| Tetratetracontane | 746.30 | 1049.60 | 1024.65 | 24.95 | 2.38 |
| Octaphenylpentacyclosilsesquioxane | 792.40 | 1129.00 | 1087.78 | 41.22 | 3.65 |
| Pentacontane | 846.50 | 1220.90 | 1161.86 | 59.04 | 4.84 |
