# Supplementary material for: Calculation of the Isobaric Heat Capacities of the Liquid and Solid Phase of Organic Compounds at and around 298.15 K Based on Their “True” Molecular Volume
Source: Molecules. 2019 Apr 24;24(8):1626. doi: 10.3390/molecules24081626 (PMC6514989; doi:10.3390/molecules24081626)
Supplement: Supplementary file 1 [file molecules-24-01626-s001.zip › S09. Experimental and Calculated Cp(sol,298) Data Table to Figure 12.doc]

| **Molecule name** | **Molecular volume** | **Cp(sol,298) exp** | **Cp(sol,298) calc** | **Deviation** | **Dev. in %** |
| --- | --- | --- | --- | --- | --- |
| Oxalic acid | 67.90 | 91.00 | 90.80 | 0.20 | 0.21 |
| Squaric acid | 80.00 | 121.80 | 109.24 | 12.56 | 10.31 |
| Malonic acid | 81.70 | 127.63 | 111.83 | 15.80 | 12.38 |
| Lactic acid | 81.80 | 127.60 | 111.98 | 15.62 | 12.24 |
| 2-Methyl-2-propanol | 86.80 | 146.11 | 119.60 | 26.51 | 18.14 |
| Serine | 91.30 | 135.60 | 126.46 | 9.14 | 6.74 |
| Phenol | 92.30 | 127.44 | 127.98 | -0.54 | -0.42 |
| Dibromoacetic acid | 92.80 | 124.70 | 128.74 | -4.04 | -3.24 |
| 2-Furoic acid | 92.90 | 132.26 | 128.89 | 3.37 | 2.54 |
| Maleic acid | 94.10 | 135.60 | 130.72 | 4.88 | 3.60 |
| Fumaric acid | 96.00 | 141.80 | 133.62 | 8.18 | 5.77 |
| cis-3-Chloro-2-butenoic acid | 96.20 | 140.20 | 133.92 | 6.28 | 4.48 |
| trans-3-Chloro-2-butenoic acid | 97.20 | 159.80 | 135.45 | 24.35 | 15.24 |
| 4-Fluorophenol | 97.20 | 144.60 | 135.45 | 9.15 | 6.33 |
| 3-Thiophenecarboxylic acid | 99.70 | 134.17 | 139.25 | -5.08 | -3.79 |
| 2-Thiophenecarboxylic acid | 100.00 | 153.78 | 139.71 | 14.07 | 9.15 |
| Butanedioic acid | 100.90 | 152.93 | 141.08 | 11.85 | 7.75 |
| 1,3-Dihydroxybenzene | 101.00 | 135.53 | 141.24 | -5.71 | -4.21 |
| 1,4-Dihydroxybenzene | 101.00 | 136.40 | 141.24 | -4.84 | -3.55 |
| 1,2-Dihydroxybenzene | 101.10 | 144.30 | 141.39 | 2.91 | 2.02 |
| 2-Pyrazinecarboxylic acid | 102.50 | 143.50 | 143.52 | -0.02 | -0.01 |
| 5-Methylisoxazole-3-carboxylic acid | 105.30 | 146.20 | 147.79 | -1.59 | -1.09 |
| Trimethylacetic acid | 106.20 | 177.80 | 149.16 | 28.64 | 16.11 |
| Nicotinic acid | 107.00 | 151.30 | 150.38 | 0.92 | 0.61 |
| 2-Pyridinealdoxime | 108.00 | 151.93 | 151.90 | 0.03 | 0.02 |
| Diethanolamine | 108.30 | 137.00 | 152.36 | -15.36 | -11.21 |
| o-Cresol | 108.50 | 154.56 | 152.66 | 1.90 | 1.23 |
| p-Cresol | 108.80 | 150.20 | 153.12 | -2.92 | -1.94 |
| Threonine | 109.30 | 155.31 | 153.88 | 1.43 | 0.92 |
| 4-Amino-3-furazanecarboxamidoxime | 109.70 | 180.40 | 154.49 | 25.91 | 14.36 |
| Aspartic acid | 110.50 | 155.18 | 155.71 | -0.53 | -0.34 |
| Benzoic acid | 111.10 | 147.07 | 156.62 | -9.55 | -6.50 |
| 4-Hydroxybenzaldehyde | 111.50 | 149.90 | 157.23 | -7.33 | -4.89 |
| 2,2-Dimethyl-1,3-propanediol | 112.30 | 183.18 | 158.45 | 24.73 | 13.50 |
| Erythritol | 113.40 | 161.90 | 160.13 | 1.77 | 1.09 |
| Tartaric acid | 114.40 | 184.50 | 161.65 | 22.85 | 12.38 |
| Cyclohexanone oxime | 114.90 | 199.38 | 162.41 | 36.97 | 18.54 |
| 4-Nitrophenol | 115.40 | 143.90 | 163.17 | -19.27 | -13.39 |
| 3-Thiopheneacetic acid | 115.70 | 167.76 | 163.63 | 4.13 | 2.46 |
| TRIS | 116.40 | 171.27 | 164.70 | 6.57 | 3.84 |
| 2-Thiopheneacetic acid | 116.50 | 159.23 | 164.85 | -5.62 | -3.53 |
| Pentafluorophenol | 116.80 | 201.30 | 165.31 | 35.99 | 17.88 |
| 2,5-Dihydroxytoluene | 117.30 | 174.90 | 166.07 | 8.83 | 5.05 |
| 2-Methylbutanedioic acid | 117.50 | 199.60 | 166.37 | 33.23 | 16.65 |
| Pentanedioic acid | 117.60 | 186.90 | 166.53 | 20.37 | 10.90 |
| cis-1,2-Cyclohexanediol | 118.30 | 160.40 | 167.59 | -7.19 | -4.48 |
| trans-1,2-Cyclohexanediol | 118.40 | 163.20 | 167.75 | -4.55 | -2.79 |
| Salicylic acid | 118.50 | 160.90 | 167.90 | -7.00 | -4.35 |
| 4-Carboxyphenol | 120.10 | 155.20 | 170.34 | -15.14 | -9.75 |
| 3,4-Dihydroxybenzaldehyde | 120.30 | 205.90 | 170.64 | 35.26 | 17.12 |
| 3-Carboxyphenol | 120.40 | 157.30 | 170.79 | -13.49 | -8.58 |
| 3,5-Dimethylisoxazole-4-carboxylic acid | 120.40 | 177.80 | 170.79 | 7.01 | 3.94 |
| o-Anthranilic acid | 121.00 | 165.30 | 171.71 | -6.41 | -3.88 |
| m-Anthranilic acid | 123.50 | 162.80 | 175.52 | -12.72 | -7.81 |
| p-Anthranilic acid | 123.60 | 177.80 | 175.67 | 2.13 | 1.20 |
| 4-Ethylphenol | 124.50 | 206.90 | 177.04 | 29.86 | 14.43 |
| 2-Chlorobenzoic acid | 124.70 | 163.20 | 177.34 | -14.14 | -8.67 |
| 3-Chlorobenzoic acid | 125.70 | 163.60 | 178.87 | -15.27 | -9.33 |
| 4-Chlorobenzoic acid | 125.70 | 167.80 | 178.87 | -11.07 | -6.60 |
| 2-Methylbenzoic acid | 125.70 | 174.90 | 178.87 | -3.97 | -2.27 |
| Pentaerythritol | 127.60 | 188.40 | 181.76 | 6.64 | 3.52 |
| 3-Methylbenzoic acid | 127.80 | 163.60 | 182.07 | -18.47 | -11.29 |
| 4-Methylbenzoic acid | 127.80 | 169.00 | 182.07 | -13.07 | -7.73 |
| Xylose | 128.50 | 180.40 | 183.13 | -2.73 | -1.52 |
| D-Ribose | 128.50 | 183.20 | 183.13 | 0.07 | 0.04 |
| 2-Bromobenzoic acid | 128.60 | 154.00 | 183.29 | -29.29 | -19.02 |
| Glutamic acid | 128.70 | 175.06 | 183.44 | -8.38 | -4.79 |
| Arabinose | 128.90 | 184.10 | 183.74 | 0.36 | 0.19 |
| Hexamethyleneglycol | 129.50 | 190.00 | 184.66 | 5.34 | 2.81 |
| Benzoylformic acid | 129.60 | 192.90 | 184.81 | 8.09 | 4.19 |
| 3-Bromobenzoic acid | 129.90 | 151.40 | 185.27 | -33.87 | -22.37 |
| 4-Bromobenzoic acid | 129.90 | 151.40 | 185.27 | -33.87 | -22.37 |
| Xylitol | 130.00 | 207.00 | 185.42 | 21.58 | 10.43 |
| Thiophenedicarboxylic acid | 130.10 | 204.87 | 185.57 | 19.30 | 9.42 |
| 2-Nitrobenzoic acid | 130.70 | 191.60 | 186.49 | 5.11 | 2.67 |
| 1,6-Anhydro-beta-D-glucopyranose | 130.70 | 187.20 | 186.49 | 0.71 | 0.38 |
| 8-Quinolinol | 130.90 | 180.42 | 186.79 | -6.37 | -3.53 |
| 1,6-Hexanedioic acid | 134.30 | 196.60 | 191.97 | 4.63 | 2.35 |
| 2,6-Pyridinedicarboxylic acid | 134.60 | 166.31 | 192.43 | -26.12 | -15.70 |
| 3-Phenylpropiolic acid | 134.60 | 180.00 | 192.43 | -12.43 | -6.90 |
| 3-Nitrobenzoic acid | 134.70 | 179.90 | 192.58 | -12.68 | -7.05 |
| Benzofuran-2-carboxylic acid | 134.70 | 184.80 | 192.58 | -7.78 | -4.21 |
| 4-Nitrobenzoic acid | 134.80 | 180.30 | 192.73 | -12.43 | -6.90 |
| 2-Naphthol | 135.30 | 172.80 | 193.49 | -20.69 | -11.98 |
| 1-Naphthol | 135.40 | 166.90 | 193.65 | -26.75 | -16.03 |
| Methylsalicylate | 136.00 | 244.30 | 194.56 | 49.74 | 20.36 |
| Mandelic acid | 136.00 | 199.20 | 194.56 | 4.64 | 2.33 |
| Tyramine | 136.30 | 213.75 | 195.02 | 18.73 | 8.76 |
| Ethriol | 137.10 | 213.80 | 196.24 | 17.56 | 8.21 |
| 4-Hydroxy-3-methoxybenzaldehyde | 137.10 | 187.60 | 196.24 | -8.64 | -4.60 |
| 4-Methoxybenzoic acid | 137.20 | 207.50 | 196.39 | 11.11 | 5.35 |
| 2-Hydroxyacetanilide | 137.60 | 182.40 | 197.00 | -14.60 | -8.00 |
| Methyl 3-hydroxybenzoate | 137.60 | 205.00 | 197.00 | 8.00 | 3.90 |
| Methyl 4-hydroxybenzoate | 137.70 | 214.41 | 197.15 | 17.26 | 8.05 |
| Gallic acid | 138.00 | 199.55 | 197.61 | 1.94 | 0.97 |
| Phthalic acid | 138.20 | 188.11 | 197.91 | -9.80 | -5.21 |
| Isophthalic acid | 138.30 | 201.70 | 198.07 | 3.63 | 1.80 |
| trans-Cinnamic acid | 138.60 | 197.50 | 198.52 | -1.02 | -0.52 |
| Indole-2-carboxylic acid | 138.90 | 178.40 | 198.98 | -20.58 | -11.54 |
| Arabitol | 138.90 | 201.35 | 198.98 | 2.37 | 1.18 |
| N-Phenylglycine | 139.40 | 177.40 | 199.74 | -22.34 | -12.59 |
| Terephthalic acid | 139.50 | 199.60 | 199.89 | -0.29 | -0.15 |
| Mesitol | 140.20 | 189.60 | 200.96 | -11.36 | -5.99 |
| 3-Trifluoromethylbenzoic acid | 142.20 | 223.60 | 204.01 | 19.59 | 8.76 |
| Homocubane-4-carboxylic acid | 143.50 | 207.00 | 205.99 | 1.01 | 0.49 |
| Thionaphthene-2-carboxylic acid | 143.60 | 179.00 | 206.14 | -27.14 | -15.16 |
| 2-Deoxy-d-glucose | 143.70 | 200.20 | 206.29 | -6.09 | -3.04 |
| 2,5-Dibromobenzoic acid | 147.00 | 223.00 | 211.32 | 11.68 | 5.24 |
| 4,5-Dihydroxy-2-(dinitromethylene)imidazolidine | 147.10 | 217.76 | 211.47 | 6.29 | 2.89 |
| 2,4,6-Tribromophenol | 147.20 | 172.00 | 211.62 | -39.62 | -23.04 |
| Trimellitic anhydride | 147.30 | 248.90 | 211.78 | 37.12 | 14.91 |
| trans-4-Coumaric acid | 147.60 | 193.00 | 212.23 | -19.23 | -9.97 |
| 3,5-Dibromobenzoic acid | 148.30 | 223.10 | 213.30 | 9.80 | 4.39 |
| Trimethylhydroquinone | 148.30 | 217.60 | 213.30 | 4.30 | 1.98 |
| Inositol | 149.90 | 218.00 | 215.74 | 2.26 | 1.04 |
| 3-Aminocinnamic acid | 150.00 | 227.60 | 215.89 | 11.71 | 5.14 |
| 3-Chloromandelic acid | 150.10 | 211.10 | 216.04 | -4.94 | -2.34 |
| beta-D-Fructose | 150.90 | 227.70 | 217.26 | 10.44 | 4.58 |
| Heptanedioic acid | 151.00 | 240.70 | 217.41 | 23.29 | 9.67 |
| Vitamin B6 | 151.20 | 244.46 | 217.72 | 26.74 | 10.94 |
| Citric acid | 151.60 | 226.40 | 218.33 | 8.07 | 3.57 |
| D-Psicose | 151.60 | 221.30 | 218.33 | 2.97 | 1.34 |
| D-Tagatose | 152.40 | 228.00 | 219.55 | 8.45 | 3.71 |
| 2-Adamantanol | 153.60 | 207.20 | 221.38 | -14.18 | -6.84 |
| alpha-D-Glucose | 153.60 | 219.19 | 221.38 | -2.19 | -1.00 |
| D-Galactose | 153.80 | 213.70 | 221.68 | -7.98 | -3.73 |
| D-Mannose | 153.80 | 215.90 | 221.68 | -5.78 | -2.68 |
| Ethyl vanillin | 153.90 | 212.20 | 221.83 | -9.63 | -4.54 |
| 1-Adamantanol | 154.00 | 196.70 | 221.99 | -25.29 | -12.85 |
| Ethyl 4-hydroxybenzoate | 154.40 | 233.93 | 222.59 | 11.34 | 4.85 |
| Ethyl 3-hydroxybenzoate | 154.40 | 217.22 | 222.59 | -5.37 | -2.47 |
| D(+)-Carnitine | 155.40 | 224.70 | 224.12 | 0.58 | 0.26 |
| 2,4-Dihydroxycinnamic acid | 155.80 | 178.36 | 224.73 | -46.37 | -26.00 |
| Caffeic acid | 156.40 | 181.96 | 225.64 | -43.68 | -24.01 |
| Hippuric acid | 158.40 | 217.00 | 228.69 | -11.69 | -5.39 |
| Dulcose | 158.80 | 238.50 | 229.30 | 9.20 | 3.86 |
| 3-Nitrophthalic acid | 159.80 | 247.02 | 230.82 | 16.20 | 6.56 |
| 2,4,6-Trinitrophenol | 160.00 | 239.70 | 231.13 | 8.57 | 3.58 |
| 2-Nitrocinnamic acid | 160.10 | 240.60 | 231.28 | 9.32 | 3.87 |
| 4-Nitrophthalic acid | 160.60 | 232.24 | 232.04 | 0.20 | 0.09 |
| 3,4-Dihydroxyhydrocinnamic acid | 161.30 | 218.62 | 233.11 | -14.49 | -6.63 |
| 2-Hydroxybiphenyl | 161.50 | 227.61 | 233.41 | -5.80 | -2.55 |
| Pentachlorophenol | 161.80 | 201.96 | 233.87 | -31.91 | -15.80 |
| 3-Nitrocinnamic acid | 161.90 | 240.20 | 234.02 | 6.18 | 2.57 |
| 4-Nitrocinnamic acid | 161.90 | 238.10 | 234.02 | 4.08 | 1.71 |
| 1,8-Octanediol | 162.90 | 236.36 | 235.55 | 0.81 | 0.34 |
| Isoborneol | 163.20 | 261.06 | 236.00 | 25.06 | 9.60 |
| Sorbitol | 163.50 | 239.00 | 236.46 | 2.54 | 1.06 |
| Tyrosine | 164.40 | 216.44 | 237.83 | -21.39 | -9.88 |
| Octanedioic acid | 167.20 | 267.60 | 242.10 | 25.50 | 9.53 |
| 1-Methoxy-a-D-glucopyranoside | 167.30 | 233.70 | 242.25 | -8.55 | -3.66 |
| 4,4'-Dihydroxybiphenyl | 171.40 | 224.31 | 248.50 | -24.19 | -10.78 |
| 2,3,5,6-Tetramethylbenzoic acid | 172.80 | 231.70 | 250.63 | -18.93 | -8.17 |
| Ferulic acid | 173.20 | 235.94 | 251.24 | -15.30 | -6.48 |
| Dopa | 173.30 | 260.60 | 251.39 | 9.21 | 3.53 |
| 2,3,4,5-Tetramethylbenzoic acid | 174.40 | 249.50 | 253.07 | -3.57 | -1.43 |
| Menthol | 174.80 | 250.10 | 253.68 | -3.58 | -1.43 |
| 1,4-Bis-(2-hydroxyethyl)piperazine | 175.10 | 250.68 | 254.13 | -3.45 | -1.38 |
| Glyceryl-2-benzoate | 179.30 | 236.40 | 260.53 | -24.13 | -10.21 |
| Diphenylmethanol | 179.30 | 236.80 | 260.53 | -23.73 | -10.02 |
| 1,9-Nonanediol | 179.60 | 256.74 | 260.99 | -4.25 | -1.65 |
| 1-Benzoylglycerol | 179.80 | 238.90 | 261.29 | -22.39 | -9.37 |
| p-Methacryloyloxybenzoic acid | 182.30 | 257.90 | 265.10 | -7.20 | -2.79 |
| 2,6-Naphthalenedicarboxylic acid | 182.80 | 230.30 | 265.86 | -35.56 | -15.44 |
| 3-Cyano-4-methoxymethyl-5-nitro-6-methyl-(2-pyridone) | 183.90 | 275.80 | 267.54 | 8.26 | 2.99 |
| Nonanedioic acid | 184.40 | 294.50 | 268.30 | 26.20 | 8.90 |
| N-Methylephedrine | 184.70 | 298.89 | 268.76 | 30.13 | 10.08 |
| Bisphenol F | 188.50 | 246.30 | 274.55 | -28.25 | -11.47 |
| 9-Hydroxy-1,4-anthraquinone | 188.80 | 237.67 | 275.01 | -37.34 | -15.71 |
| N-Acetyl-D-glucosamine | 189.30 | 276.90 | 275.77 | 1.13 | 0.41 |
| Pentamethylbenzoic acid | 190.00 | 249.90 | 276.83 | -26.93 | -10.78 |
| 1,10-Decanediol | 196.30 | 279.26 | 286.43 | -7.17 | -2.57 |
| Decanedioic acid | 201.10 | 321.40 | 293.75 | 27.65 | 8.60 |
| Bisphenol E | 204.60 | 276.41 | 299.08 | -22.67 | -8.20 |
| Benzoylglycylglycine | 206.00 | 277.40 | 301.21 | -23.81 | -8.58 |
| Bisphenol S | 206.20 | 280.30 | 301.52 | -21.22 | -7.57 |
| 5-Hydroxyflavone | 206.70 | 279.80 | 302.28 | -22.48 | -8.03 |
| 3-Hydroxyflavone | 207.00 | 270.10 | 302.74 | -32.64 | -12.08 |
| Ibuprofen | 208.20 | 328.01 | 304.56 | 23.45 | 7.15 |
| 6-Hydroxyflavone | 208.80 | 250.30 | 305.48 | -55.18 | -22.04 |
| 7-Hydroxyflavone | 208.80 | 253.10 | 305.48 | -52.38 | -20.69 |
| Arabinosylhypoxanthine | 208.90 | 283.30 | 305.63 | -22.33 | -7.88 |
| N-Methylephedrine hydrochloride | 210.80 | 282.29 | 308.52 | -26.23 | -9.29 |
| 3,3'-Dihydroxy-4,4'-diaminodiphenylmethane | 210.90 | 281.20 | 308.68 | -27.48 | -9.77 |
| 2-(6-Methoxynaphthalen-2-yl)propanoic acid | 211.90 | 296.30 | 310.20 | -13.90 | -4.69 |
| 1,11-Undecanediol | 213.00 | 297.79 | 311.88 | -14.09 | -4.73 |
| Undecanedioic acid | 217.80 | 348.30 | 319.19 | 29.11 | 8.36 |
| Vidarabine | 218.70 | 290.10 | 320.56 | -30.46 | -10.50 |
| Bisphenol A | 219.00 | 301.34 | 321.02 | -19.68 | -6.53 |
| 1-(2,2,3-Trimethyl-1,3-oxazolidin-5-yl)-butane-1,2,3,4-tetrol | 219.90 | 331.46 | 322.39 | 9.07 | 2.74 |
| PMC | 220.10 | 315.30 | 322.69 | -7.39 | -2.35 |
| 4-(Phenylmethoxy)benzeneacetic acid | 224.60 | 340.00 | 329.55 | 10.45 | 3.07 |
| Tri-t-butylmethanol | 225.60 | 350.60 | 331.07 | 19.53 | 5.57 |
| Ellagic acid | 226.60 | 328.85 | 332.60 | -3.75 | -1.14 |
| 1,12-Dodecanediol | 229.70 | 330.23 | 337.32 | -7.09 | -2.15 |
| Trolox | 231.40 | 332.00 | 339.91 | -7.91 | -2.38 |
| Dodecanedioic acid | 234.50 | 375.20 | 344.63 | 30.57 | 8.15 |
| 1-Tridecanol | 237.50 | 378.00 | 349.20 | 28.80 | 7.62 |
| Tridecanoic acid | 239.90 | 387.60 | 352.86 | 34.74 | 8.96 |
| 1,13-Tridecanediol | 246.40 | 366.88 | 362.76 | 4.12 | 1.12 |
| 3,5-Di-t-butylsalicylic acid | 247.00 | 383.00 | 363.68 | 19.32 | 5.04 |
| Triphenylmethanol | 248.60 | 318.80 | 366.12 | -47.32 | -14.84 |
| 1,13-Tridecanedioic acid | 251.20 | 402.10 | 370.08 | 32.02 | 7.96 |
| Myristyl alcohol | 254.20 | 388.00 | 374.65 | 13.35 | 3.44 |
| Tetradecanoic acid | 256.60 | 432.01 | 378.30 | 53.71 | 12.43 |
| 1-Monocaprin | 258.20 | 410.00 | 380.74 | 29.26 | 7.14 |
| 1,14-Tetradecanediol | 263.10 | 379.61 | 388.21 | -8.60 | -2.26 |
| 1,14-Tetradecanedioic acid | 267.90 | 429.00 | 395.52 | 33.48 | 7.80 |
| 1-Pentadecanol | 270.90 | 400.00 | 400.09 | -0.09 | -0.02 |
| 1-Pentadecanoic acid | 273.30 | 443.28 | 403.75 | 39.53 | 8.92 |
| Bisphenol AP | 274.00 | 365.85 | 404.82 | -38.97 | -10.65 |
| N-(2-Hydroxy-4-methoxybenzylidene)-4-butylaniline | 275.20 | 451.00 | 406.64 | 44.36 | 9.84 |
| Cellobiose | 277.50 | 436.10 | 410.15 | 25.95 | 5.95 |
| Maltose | 277.90 | 434.70 | 410.76 | 23.94 | 5.51 |
| 1,15-Pentadecanediol | 279.80 | 377.45 | 413.65 | -36.20 | -9.59 |
| Saccharose | 281.50 | 424.30 | 416.24 | 8.06 | 1.90 |
| Lactose | 283.60 | 412.50 | 419.44 | -6.94 | -1.68 |
| 1-Hexadecanol | 287.60 | 422.00 | 425.54 | -3.54 | -0.84 |
| Palmitic acid | 290.00 | 460.70 | 429.19 | 31.51 | 6.84 |
| Glyceryl-2-laurate | 290.30 | 436.40 | 429.65 | 6.75 | 1.55 |
| 1-Glyceryl laurate | 291.30 | 447.70 | 431.17 | 16.53 | 3.69 |
| 1,16-Hexadecanediol | 296.50 | 426.18 | 439.10 | -12.92 | -3.03 |
| Hexadecanedioic acid | 301.30 | 482.80 | 446.41 | 36.39 | 7.54 |
| Margaric acid | 306.70 | 475.70 | 454.64 | 21.06 | 4.43 |
| Vitamin B2 | 321.30 | 442.20 | 476.88 | -34.68 | -7.84 |
| Stearic acid | 323.40 | 501.60 | 480.08 | 21.52 | 4.29 |
| 2-Monomyristin | 323.50 | 506.30 | 480.23 | 26.07 | 5.15 |
| Methylprednisolone | 338.50 | 472.10 | 503.09 | -30.99 | -6.56 |
| 1-Nonadecanoic acid | 340.10 | 525.40 | 505.52 | 19.88 | 3.78 |
| Arachidic acid | 356.80 | 545.10 | 530.97 | 14.13 | 2.59 |
| 2-Monopalmitin | 357.80 | 558.60 | 532.49 | 26.11 | 4.67 |
| 1-Palmitoylglycerol | 358.10 | 566.90 | 532.95 | 33.95 | 5.99 |
| 2-Stearoylglycerol | 391.10 | 610.40 | 583.23 | 27.17 | 4.45 |
| 1-Glyceryl stearate | 391.70 | 610.40 | 584.14 | 26.26 | 4.30 |
| N-Formylmethionine-leucyl-phenylalanine | 403.70 | 567.60 | 602.42 | -34.82 | -6.14 |
| Maltotriose | 406.70 | 662.50 | 606.99 | 55.51 | 8.38 |
| Simvastatin | 408.40 | 577.00 | 609.58 | -32.58 | -5.65 |
| Pyrimethanil laurate | 411.70 | 584.50 | 614.61 | -30.11 | -5.15 |
| Methylprednisolone aceponate | 426.90 | 604.80 | 637.77 | -32.97 | -5.45 |
| Tetraphenyltetrahydroxycyclotetrasiloxane | 432.00 | 626.50 | 645.54 | -19.04 | -3.04 |
| Cyclodextrin | 776.30 | 1153.00 | 1170.11 | -17.11 | -1.48 |
