# Supplementary material for: Calculation of the Isobaric Heat Capacities of the Liquid and Solid Phase of Organic Compounds at and around 298.15 K Based on Their “True” Molecular Volume
Source: Molecules. 2019 Apr 24;24(8):1626. doi: 10.3390/molecules24081626 (PMC6514989; doi:10.3390/molecules24081626)
Supplement: Supplementary file 1 [file molecules-24-01626-s001.zip › S11. Experimental and Calculated Cp(sol,298) Data Table to Figure 14.doc]

| **Molecule name** | **Molecular volume** | **Cp(sol,298) exp** | **Cp(sol,298) calc** | **Deviation** | **Dev. in %** |
| --- | --- | --- | --- | --- | --- |
| Oxalic acid | 67.90 | 91.00 | 91.73 | -0.73 | -0.80 |
| Squaric acid | 80.00 | 121.80 | 110.25 | 11.55 | 9.48 |
| Malonic acid | 81.70 | 127.63 | 112.85 | 14.78 | 11.58 |
| Lactic acid | 81.80 | 127.60 | 113.00 | 14.60 | 11.44 |
| Maleic acid | 94.10 | 135.60 | 131.83 | 3.77 | 2.78 |
| Fumaric acid | 96.00 | 141.80 | 134.73 | 7.07 | 4.98 |
| Butanedioic acid | 100.90 | 152.93 | 142.23 | 10.70 | 6.99 |
| 1,3-Dihydroxybenzene | 101.00 | 135.53 | 142.39 | -6.86 | -5.06 |
| 1,4-Dihydroxybenzene | 101.00 | 136.40 | 142.39 | -5.99 | -4.39 |
| 1,2-Dihydroxybenzene | 101.10 | 144.30 | 142.54 | 1.76 | 1.22 |
| Diethanolamine | 108.30 | 137.00 | 153.56 | -16.56 | -12.09 |
| 2,2-Dimethyl-1,3-propanediol | 112.30 | 183.18 | 159.68 | 23.50 | 12.83 |
| Erythritol | 113.40 | 161.90 | 161.36 | 0.54 | 0.33 |
| Tartaric acid | 114.40 | 184.50 | 162.89 | 21.61 | 11.71 |
| TRIS | 116.40 | 171.27 | 165.95 | 5.32 | 3.10 |
| 2,5-Dihydroxytoluene | 117.30 | 174.90 | 167.33 | 7.57 | 4.33 |
| 2-Methylbutanedioic acid | 117.50 | 199.60 | 167.64 | 31.96 | 16.01 |
| Pentanedioic acid | 117.60 | 186.90 | 167.79 | 19.11 | 10.22 |
| cis-1,2-Cyclohexanediol | 118.30 | 160.40 | 168.86 | -8.46 | -5.28 |
| trans-1,2-Cyclohexanediol | 118.40 | 163.20 | 169.02 | -5.82 | -3.56 |
| Salicylic acid | 118.50 | 160.90 | 169.17 | -8.27 | -5.14 |
| 4-Carboxyphenol | 120.10 | 155.20 | 171.62 | -16.42 | -10.58 |
| 3,4-Dihydroxybenzaldehyde | 120.30 | 205.90 | 171.92 | 33.98 | 16.50 |
| 3-Carboxyphenol | 120.40 | 157.30 | 172.08 | -14.78 | -9.39 |
| Pentaerythritol | 127.60 | 188.40 | 183.09 | 5.31 | 2.82 |
| Xylose | 128.50 | 180.40 | 184.47 | -4.07 | -2.26 |
| D-Ribose | 128.50 | 183.20 | 184.47 | -1.27 | -0.69 |
| Arabinose | 128.90 | 184.10 | 185.08 | -0.98 | -0.53 |
| Hexamethyleneglycol | 129.50 | 190.00 | 186.00 | 4.00 | 2.10 |
| Xylitol | 130.00 | 207.00 | 186.77 | 20.23 | 9.77 |
| Thiophenedicarboxylic acid | 130.10 | 204.87 | 186.92 | 17.95 | 8.76 |
| 1,6-Anhydro-beta-D-glucopyranose | 130.70 | 187.20 | 187.84 | -0.64 | -0.34 |
| 1,6-Hexanedioic acid | 134.30 | 196.60 | 193.35 | 3.25 | 1.65 |
| 2,6-Pyridinedicarboxylic acid | 134.60 | 166.31 | 193.81 | -27.50 | -16.53 |
| Mandelic acid | 136.00 | 199.20 | 195.95 | 3.25 | 1.63 |
| Ethriol | 137.10 | 213.80 | 197.63 | 16.17 | 7.56 |
| Gallic acid | 138.00 | 199.55 | 199.01 | 0.54 | 0.27 |
| Phthalic acid | 138.20 | 188.11 | 199.32 | -11.21 | -5.96 |
| Isophthalic acid | 138.30 | 201.70 | 199.47 | 2.23 | 1.11 |
| Arabitol | 138.90 | 201.35 | 200.39 | 0.96 | 0.48 |
| Terephthalic acid | 139.50 | 199.60 | 201.31 | -1.71 | -0.86 |
| 2-Deoxy-d-glucose | 143.70 | 200.20 | 207.73 | -7.53 | -3.76 |
| 4,5-Dihydroxy-2-(dinitromethylene)imidazolidine | 147.10 | 217.76 | 212.94 | 4.82 | 2.21 |
| trans-4-Coumaric acid | 147.60 | 193.00 | 213.70 | -20.70 | -10.73 |
| Trimethylhydroquinone | 148.30 | 217.60 | 214.77 | 2.83 | 1.30 |
| Inositol | 149.90 | 218.00 | 217.22 | 0.78 | 0.36 |
| 3-Chloromandelic acid | 150.10 | 211.10 | 217.53 | -6.43 | -3.05 |
| beta-D-Fructose | 150.90 | 227.70 | 218.75 | 8.95 | 3.93 |
| Heptanedioic acid | 151.00 | 240.70 | 218.91 | 21.79 | 9.05 |
| Vitamin B6 | 151.20 | 244.46 | 219.21 | 25.25 | 10.33 |
| Citric acid | 151.60 | 226.40 | 219.82 | 6.58 | 2.90 |
| D-Psicose | 151.60 | 221.30 | 219.82 | 1.48 | 0.67 |
| D-Tagatose | 152.40 | 228.00 | 221.05 | 6.95 | 3.05 |
| alpha-D-Glucose | 153.60 | 219.19 | 222.89 | -3.70 | -1.69 |
| D-Galactose | 153.80 | 213.70 | 223.19 | -9.49 | -4.44 |
| D-Mannose | 153.80 | 215.90 | 223.19 | -7.29 | -3.38 |
| 2,4-Dihydroxycinnamic acid | 155.80 | 178.36 | 226.25 | -47.89 | -26.85 |
| Caffeic acid | 156.40 | 181.96 | 227.17 | -45.21 | -24.85 |
| Dulcose | 158.80 | 238.50 | 230.84 | 7.66 | 3.21 |
| 3-Nitrophthalic acid | 159.80 | 247.02 | 232.37 | 14.65 | 5.93 |
| 4-Nitrophthalic acid | 160.60 | 232.24 | 233.60 | -1.36 | -0.58 |
| 3,4-Dihydroxyhydrocinnamic acid | 161.30 | 218.62 | 234.67 | -16.05 | -7.34 |
| 1,8-Octanediol | 162.90 | 236.36 | 237.12 | -0.76 | -0.32 |
| Sorbitol | 163.50 | 239.00 | 238.04 | 0.96 | 0.40 |
| Octanedioic acid | 167.20 | 267.60 | 243.70 | 23.90 | 8.93 |
| 1-Methoxy-a-D-glucopyranoside | 167.30 | 233.70 | 243.85 | -10.15 | -4.34 |
| 4,4'-Dihydroxybiphenyl | 171.40 | 224.31 | 250.13 | -25.82 | -11.51 |
| Ferulic acid | 173.20 | 235.94 | 252.88 | -16.94 | -7.18 |
| Dopa | 173.30 | 260.60 | 253.03 | 7.57 | 2.90 |
| 1,4-Bis-(2-hydroxyethyl)piperazine | 175.10 | 250.68 | 255.79 | -5.11 | -2.04 |
| Glyceryl-2-benzoate | 179.30 | 236.40 | 262.22 | -25.82 | -10.92 |
| 1,9-Nonanediol | 179.60 | 256.74 | 262.68 | -5.94 | -2.31 |
| 1-Benzoylglycerol | 179.80 | 238.90 | 262.98 | -24.08 | -10.08 |
| 2,6-Naphthalenedicarboxylic acid | 182.80 | 230.30 | 267.57 | -37.27 | -16.18 |
| Nonanedioic acid | 184.40 | 294.50 | 270.02 | 24.48 | 8.31 |
| Bisphenol F | 188.50 | 246.30 | 276.30 | -30.00 | -12.18 |
| N-Acetyl-D-glucosamine | 189.30 | 276.90 | 277.52 | -0.62 | -0.22 |
| 1,10-Decanediol | 196.30 | 279.26 | 288.23 | -8.97 | -3.21 |
| Decanedioic acid | 201.10 | 321.40 | 295.58 | 25.82 | 8.03 |
| Bisphenol E | 204.60 | 276.41 | 300.94 | -24.53 | -8.87 |
| Bisphenol S | 206.20 | 280.30 | 303.38 | -23.08 | -8.24 |
| Arabinosylhypoxanthine | 208.90 | 283.30 | 307.52 | -24.22 | -8.55 |
| 3,3'-Dihydroxy-4,4'-diaminodiphenylmethane | 210.90 | 281.20 | 310.58 | -29.38 | -10.45 |
| 1,11-Undecanediol | 213.00 | 297.79 | 313.79 | -16.00 | -5.37 |
| Undecanedioic acid | 217.80 | 348.30 | 321.14 | 27.16 | 7.80 |
| Vidarabine | 218.70 | 290.10 | 322.51 | -32.41 | -11.17 |
| Bisphenol A | 219.00 | 301.34 | 322.97 | -21.63 | -7.18 |
| 1-(2,2,3-Trimethyl-1,3-oxazolidin-5-yl)-butane-1,2,3,4-tetrol | 219.90 | 331.46 | 324.35 | 7.11 | 2.14 |
| Ellagic acid | 226.60 | 328.85 | 334.60 | -5.75 | -1.75 |
| 1,12-Dodecanediol | 229.70 | 330.23 | 339.35 | -9.12 | -2.76 |
| Trolox | 231.40 | 332.00 | 341.95 | -9.95 | -3.00 |
| Dodecanedioic acid | 234.50 | 375.20 | 346.70 | 28.50 | 7.60 |
| 1,13-Tridecanediol | 246.40 | 366.88 | 364.91 | 1.97 | 0.54 |
| 3,5-Di-t-butylsalicylic acid | 247.00 | 383.00 | 365.83 | 17.17 | 4.48 |
| 1,13-Tridecanedioic acid | 251.20 | 402.10 | 372.25 | 29.85 | 7.42 |
| 1-Monocaprin | 258.20 | 410.00 | 382.97 | 27.03 | 6.59 |
| 1,14-Tetradecanediol | 263.10 | 379.61 | 390.46 | -10.85 | -2.86 |
| 1,14-Tetradecanedioic acid | 267.90 | 429.00 | 397.81 | 31.19 | 7.27 |
| Bisphenol AP | 274.00 | 365.85 | 407.15 | -41.30 | -11.29 |
| Cellobiose | 277.50 | 436.10 | 412.50 | 23.60 | 5.41 |
| Maltose | 277.90 | 434.70 | 413.11 | 21.59 | 4.97 |
| 1,15-Pentadecanediol | 279.80 | 377.45 | 416.02 | -38.57 | -10.22 |
| Saccharose | 281.50 | 424.30 | 418.62 | 5.68 | 1.34 |
| Lactose | 283.60 | 412.50 | 421.84 | -9.34 | -2.26 |
| Glyceryl-2-laurate | 290.30 | 436.40 | 432.09 | 4.31 | 0.99 |
| 1-Glyceryl laurate | 291.30 | 447.70 | 433.62 | 14.08 | 3.14 |
| 1,16-Hexadecanediol | 296.50 | 426.18 | 441.58 | -15.40 | -3.61 |
| Hexadecanedioic acid | 301.30 | 482.80 | 448.93 | 33.87 | 7.02 |
| Vitamin B2 | 321.30 | 442.20 | 479.53 | -37.33 | -8.44 |
| 2-Monomyristin | 323.50 | 506.30 | 482.90 | 23.40 | 4.62 |
| Methylprednisolone | 338.50 | 472.10 | 505.86 | -33.76 | -7.15 |
| 2-Monopalmitin | 357.80 | 558.60 | 535.39 | 23.21 | 4.15 |
| 1-Palmitoylglycerol | 358.10 | 566.90 | 535.85 | 31.05 | 5.48 |
| 2-Stearoylglycerol | 391.10 | 610.40 | 586.36 | 24.04 | 3.94 |
| 1-Glyceryl stearate | 391.70 | 610.40 | 587.27 | 23.13 | 3.79 |
| Maltotriose | 406.70 | 662.50 | 610.23 | 52.27 | 7.89 |
| Tetraphenyltetrahydroxycyclotetrasiloxane | 432.00 | 626.50 | 648.95 | -22.45 | -3.58 |
| Cyclodextrin | 776.30 | 1153.00 | 1175.87 | -22.87 | -1.98 |
