# Supplementary material for: Calculation of the Isobaric Heat Capacities of the Liquid and Solid Phase of Organic Compounds at and around 298.15 K Based on Their “True” Molecular Volume
Source: Molecules. 2019 Apr 24;24(8):1626. doi: 10.3390/molecules24081626 (PMC6514989; doi:10.3390/molecules24081626)
Supplement: Supplementary file 1 [file molecules-24-01626-s001.zip › S12. Experimental and Calculated Cp(liq,250) Data Table to Figure 15A.doc]

| **Molecule name** | **Molecular volume** | **Cp(liq,250) exp** | **Cp(liq,250) calc** | **Deviation** | **Dev. in %** |
| --- | --- | --- | --- | --- | --- |
| Butane | 78.20 | 127.70 | 123.15 | 4.55 | 3.56 |
| Pentane | 94.90 | 152.15 | 151.77 | 0.38 | 0.25 |
| Ethyl propanoate | 107.00 | 196.90 | 172.51 | 24.39 | 12.39 |
| Hexane | 111.50 | 181.04 | 180.22 | 0.82 | 0.45 |
| Heptane | 128.20 | 209.10 | 208.84 | 0.26 | 0.13 |
| Cyclohexyl formate | 129.60 | 203.45 | 211.23 | -7.78 | -3.83 |
| 1,4-Diisocyanatobutane | 130.10 | 216.33 | 212.09 | 4.24 | 1.96 |
| Ethyl pentanoate | 140.10 | 244.99 | 229.23 | 15.76 | 6.43 |
| Methyl o-toluate | 143.90 | 231.14 | 235.74 | -4.60 | -1.99 |
| Cyclohexyl acetate | 144.50 | 227.81 | 236.77 | -8.96 | -3.93 |
| Octane | 144.90 | 237.46 | 237.45 | 0.01 | 0.00 |
| Methyl m-toluate | 145.00 | 231.56 | 237.63 | -6.07 | -2.62 |
| t-Butylbenzene | 148.60 | 220.62 | 243.79 | -23.17 | -10.50 |
| Nonane | 161.50 | 267.00 | 265.90 | 1.10 | 0.41 |
| Hexamethyldisiloxane | 168.30 | 290.88 | 277.55 | 13.33 | 4.58 |
| 5-Trimethylsilyl-2-norbornene | 174.60 | 263.20 | 288.35 | -25.15 | -9.56 |
| 1-Chlorononane | 176.00 | 278.00 | 290.75 | -12.75 | -4.59 |
| Decane | 178.20 | 297.47 | 294.52 | 2.95 | 0.99 |
| Cyclohexyl butanoate | 178.60 | 278.53 | 295.21 | -16.68 | -5.99 |
| Tetraethylurea | 184.50 | 325.90 | 305.32 | 20.58 | 6.32 |
| 2,4,6,8-Tetramethylcyclotetrasiloxane | 194.70 | 332.60 | 322.80 | 9.80 | 2.95 |
| Cyclohexyl valerate | 195.10 | 302.81 | 323.48 | -20.67 | -6.83 |
| 1-Butyl-3-methylimidazolium acetate | 200.70 | 363.09 | 333.08 | 30.01 | 8.27 |
| 2-Ethylhexyl acrylate | 201.00 | 336.00 | 333.59 | 2.41 | 0.72 |
| 1,1'-Diethylferrocene | 210.40 | 317.30 | 349.70 | -32.40 | -10.21 |
| 1-Butyl-3-methylimidazolium trifluoroacetate | 214.70 | 387.37 | 357.07 | 30.30 | 7.82 |
| Dibenzyldisulfide | 223.60 | 348.40 | 372.32 | -23.92 | -6.87 |
| Octamethyltrisiloxane | 231.80 | 397.68 | 386.37 | 11.31 | 2.84 |
| Decamethyltetrasiloxane | 294.20 | 503.26 | 493.31 | 9.95 | 1.98 |
