# Supplementary material for: Calculation of the Isobaric Heat Capacities of the Liquid and Solid Phase of Organic Compounds at and around 298.15 K Based on Their “True” Molecular Volume
Source: Molecules. 2019 Apr 24;24(8):1626. doi: 10.3390/molecules24081626 (PMC6514989; doi:10.3390/molecules24081626)
Supplement: Supplementary file 1 [file molecules-24-01626-s001.zip › S13. Experimental and Calculated Cp(liq,275) Data Table to Figure 15B.doc]

| **Molecule name** | **Molecular volume** | **Cp(liq,275) exp** | **Cp(liq,275) calc** | **Deviation** | **Dev. in %** |
| --- | --- | --- | --- | --- | --- |
| cis-1,2-Dichloroethylene | 67.90 | 111.00 | 106.60 | 4.40 | 3.96 |
| trans-1,2-Dichloroethylene | 68.10 | 112.70 | 106.96 | 5.74 | 5.09 |
| Butane | 78.20 | 134.10 | 125.03 | 9.07 | 6.77 |
| Trichloroethylene | 82.20 | 127.21 | 132.18 | -4.97 | -3.91 |
| 1,1-Dichloropropane | 89.70 | 150.24 | 145.60 | 4.64 | 3.09 |
| 1,2-Dichloropropane | 89.80 | 153.81 | 145.78 | 8.03 | 5.22 |
| 2,2-Dichloropropane | 89.80 | 152.80 | 145.78 | 7.02 | 4.60 |
| Pentane | 94.90 | 160.22 | 154.90 | 5.32 | 3.32 |
| Ethyl propanoate | 107.00 | 200.15 | 176.54 | 23.61 | 11.80 |
| Hexane | 111.50 | 188.10 | 184.59 | 3.51 | 1.87 |
| Heptane | 128.20 | 216.62 | 214.46 | 2.16 | 1.00 |
| Methyl benzoate | 128.70 | 210.85 | 215.36 | -4.51 | -2.14 |
| 1,4-Diisocyanatobutane | 130.10 | 220.57 | 217.86 | 2.71 | 1.23 |
| 1-Phenylpyrazole | 133.00 | 222.35 | 223.05 | -0.70 | -0.31 |
| 1-Phenylimidazole | 134.40 | 240.40 | 225.55 | 14.85 | 6.18 |
| Trichlorotoluene | 141.10 | 212.77 | 237.54 | -24.77 | -11.64 |
| Methyl o-toluate | 143.90 | 237.04 | 242.55 | -5.51 | -2.32 |
| Octane | 144.90 | 245.42 | 244.34 | 1.08 | 0.44 |
| Methyl m-toluate | 145.00 | 239.20 | 244.51 | -5.31 | -2.22 |
| Dipropyl carbonate | 150.50 | 267.66 | 254.35 | 13.31 | 4.97 |
| 3-Carene | 152.20 | 244.10 | 257.39 | -13.29 | -5.45 |
| Dihydropinene | 155.30 | 237.24 | 262.94 | -25.70 | -10.83 |
| Fenchone | 156.90 | 259.97 | 265.80 | -5.83 | -2.24 |
| 1-Methyl-4-isopropylcyclohexene | 160.20 | 247.09 | 271.70 | -24.61 | -9.96 |
| Nonane | 161.50 | 274.74 | 274.03 | 0.71 | 0.26 |
| 1,8-Cineole | 164.40 | 258.50 | 279.22 | -20.72 | -8.01 |
| Neopentylbenzene | 165.20 | 259.81 | 280.65 | -20.84 | -8.02 |
| Hexamethyldisiloxane | 168.30 | 299.33 | 286.19 | 13.14 | 4.39 |
| 5-Trimethylsilyl-2-norbornene | 174.60 | 279.00 | 297.46 | -18.46 | -6.62 |
| 1-Chlorononane | 176.00 | 286.00 | 299.97 | -13.97 | -4.88 |
| Decane | 178.20 | 304.78 | 303.90 | 0.88 | 0.29 |
| Cyclohexyl butanoate | 178.60 | 290.59 | 304.62 | -14.03 | -4.83 |
| Tetraethylurea | 184.50 | 335.55 | 315.17 | 20.38 | 6.07 |
| Undecane | 194.90 | 335.22 | 333.77 | 1.45 | 0.43 |
| Cyclohexyl valerate | 195.10 | 312.36 | 334.13 | -21.77 | -6.97 |
| 1-Butyl-3-methylimidazolium acetate | 200.70 | 372.61 | 344.15 | 28.46 | 7.64 |
| 2-Ethylhexyl acrylate | 201.00 | 345.30 | 344.68 | 0.62 | 0.18 |
| Dodecane | 211.60 | 366.33 | 363.64 | 2.69 | 0.73 |
| 1-Butyl-3-methylimidazolium trifluoroacetate | 214.70 | 397.35 | 369.19 | 28.16 | 7.09 |
| Dibenzyldisulfide | 223.60 | 386.35 | 385.11 | 1.24 | 0.32 |
| Tridecane | 228.60 | 397.36 | 394.05 | 3.31 | 0.83 |
| Octamethyltrisiloxane | 231.80 | 408.28 | 399.78 | 8.50 | 2.08 |
| Decamethyltetrasiloxane | 294.20 | 521.38 | 511.39 | 9.99 | 1.92 |
