# Supplementary material for: Calculation of the Isobaric Heat Capacities of the Liquid and Solid Phase of Organic Compounds at and around 298.15 K Based on Their “True” Molecular Volume
Source: Molecules. 2019 Apr 24;24(8):1626. doi: 10.3390/molecules24081626 (PMC6514989; doi:10.3390/molecules24081626)
Supplement: Supplementary file 1 [file molecules-24-01626-s001.zip › S14. Experimental and Calculated Cp(liq,325) Data Table to Figure 15C.doc]

| **Molecule name** | **Molecular volume** | **Cp(liq,325) exp** | **Cp(liq,325) calc** | **Deviation** | **Dev. in %** |
| --- | --- | --- | --- | --- | --- |
| DMSO | 69.90 | 155.75 | 121.67 | 34.08 | 21.88 |
| 1-Methylimidazole | 77.00 | 152.35 | 135.32 | 17.03 | 11.18 |
| Butane | 78.20 | 152.15 | 137.63 | 14.52 | 9.54 |
| Trichloroethylene | 82.20 | 130.78 | 145.32 | -14.54 | -11.12 |
| Dimethyl carbonate | 83.50 | 163.70 | 147.82 | 15.88 | 9.70 |
| Propylenecarbonate | 88.50 | 180.80 | 157.43 | 23.37 | 12.93 |
| 1,1-Dichloropropane | 89.70 | 157.80 | 159.73 | -1.93 | -1.23 |
| 2,2-Dichloropropane | 89.80 | 163.05 | 159.93 | 3.12 | 1.92 |
| 1,2-Dichloropropane | 89.80 | 160.97 | 159.93 | 1.04 | 0.65 |
| Pentane | 94.90 | 176.65 | 169.73 | 6.92 | 3.92 |
| Methylbenzene | 100.00 | 164.90 | 179.53 | -14.63 | -8.87 |
| n-Propyl acetate | 106.90 | 202.80 | 192.80 | 10.00 | 4.93 |
| Ethyl propanoate | 107.00 | 209.82 | 192.99 | 16.83 | 8.02 |
| 2-Chloroaniline | 109.70 | 204.53 | 198.18 | 6.35 | 3.11 |
| 3-Chloroaniline | 109.80 | 208.63 | 198.37 | 10.26 | 4.92 |
| Hexane | 111.50 | 205.89 | 201.64 | 4.25 | 2.06 |
| N-Ethyl-2-pyrrolidone | 115.30 | 210.60 | 208.94 | 1.66 | 0.79 |
| Diethylcarbonate | 117.10 | 226.49 | 212.40 | 14.09 | 6.22 |
| 2-Chloronitrobenzene | 119.80 | 213.29 | 217.59 | -4.30 | -2.02 |
| Tetramethylurea | 120.50 | 236.40 | 218.94 | 17.46 | 7.39 |
| Heptane | 128.20 | 235.34 | 233.74 | 1.60 | 0.68 |
| 3,4,5,6-Tetrahydrophthalic anhydride | 129.00 | 236.21 | 235.28 | 0.93 | 0.39 |
| Hexyl bromide | 130.10 | 228.59 | 237.39 | -8.80 | -3.85 |
| 1,4-Diisocyanatobutane | 130.10 | 230.96 | 237.39 | -6.43 | -2.79 |
| 1-Phenylpyrazole | 133.00 | 244.00 | 242.97 | 1.03 | 0.42 |
| 1-Phenylimidazole | 134.40 | 258.45 | 245.66 | 12.79 | 4.95 |
| Veratrole | 134.80 | 288.04 | 246.43 | 41.61 | 14.45 |
| Isoamyl acetate | 139.90 | 262.27 | 256.23 | 6.04 | 2.30 |
| Ethyl pentanoate | 140.10 | 265.07 | 256.62 | 8.45 | 3.19 |
| Methyl hexanoate | 140.30 | 252.12 | 257.00 | -4.88 | -1.94 |
| Trichlorotoluene | 141.10 | 225.16 | 258.54 | -33.38 | -14.82 |
| Octane | 144.90 | 266.37 | 265.84 | 0.53 | 0.20 |
| N-(2-Cyanoethyl)aniline | 146.20 | 243.60 | 268.34 | -24.74 | -10.16 |
| Heptyl bromide | 146.80 | 259.52 | 269.49 | -9.97 | -3.84 |
| 2-Ethylhexanal | 147.00 | 277.93 | 269.88 | 8.05 | 2.90 |
| Camphene | 148.40 | 251.91 | 272.57 | -20.66 | -8.20 |
| t-Butylbenzene | 148.60 | 254.36 | 272.96 | -18.60 | -7.31 |
| Dipropyl carbonate | 150.50 | 285.93 | 276.61 | 9.32 | 3.26 |
| trans-Decahydroquinoline | 151.60 | 264.14 | 278.72 | -14.58 | -5.52 |
| Fenchone | 156.90 | 281.80 | 288.91 | -7.11 | -2.52 |
| 1-Bromooctane | 163.50 | 286.20 | 301.60 | -15.40 | -5.38 |
| 1,8-Cineole | 164.40 | 285.17 | 303.33 | -18.16 | -6.37 |
| Neopentylbenzene | 165.20 | 282.09 | 304.86 | -22.77 | -8.07 |
| Hexamethyldisiloxane | 168.30 | 321.34 | 310.82 | 10.52 | 3.27 |
| 4-Phenyltoluene | 169.90 | 291.02 | 313.90 | -22.88 | -7.86 |
| 2-Phenyltoluene | 170.50 | 286.13 | 315.05 | -28.92 | -10.11 |
| Ethyl trans-cinnamate | 172.90 | 301.80 | 319.67 | -17.87 | -5.92 |
| Ethyl heptanoate | 173.40 | 325.77 | 320.63 | 5.14 | 1.58 |
| Methyl octanoate | 173.40 | 325.26 | 320.63 | 4.63 | 1.42 |
| 5-Trimethylsilyl-2-norbornene | 174.60 | 307.50 | 322.93 | -15.43 | -5.02 |
| 1-Chlorononane | 176.00 | 307.00 | 325.63 | -18.63 | -6.07 |
| 1-Ethyladamantane | 177.70 | 276.62 | 328.89 | -52.27 | -18.90 |
| 1,3-Dimethyladamantane | 178.20 | 276.85 | 329.85 | -53.00 | -19.15 |
| Decane | 178.20 | 325.27 | 329.85 | -4.58 | -1.41 |
| 1-Bromononane | 180.20 | 320.75 | 333.70 | -12.95 | -4.04 |
| 2,5,8,11-Tetraoxododecane | 183.40 | 371.00 | 339.85 | 31.15 | 8.40 |
| Tetraethylurea | 184.50 | 356.80 | 341.96 | 14.84 | 4.16 |
| 3,3'-Bitolyl | 186.20 | 308.70 | 345.23 | -36.53 | -11.83 |
| n-Octylacetate | 190.00 | 355.62 | 352.54 | 3.08 | 0.87 |
| Methyl nonanoate | 190.10 | 359.43 | 352.73 | 6.70 | 1.86 |
| n-Decylamine | 190.30 | 379.38 | 353.11 | 26.27 | 6.92 |
| Ethyl caprylate | 190.50 | 355.63 | 353.50 | 2.13 | 0.60 |
| Undecane | 194.90 | 358.80 | 361.96 | -3.16 | -0.88 |
| 1-Bromodecane | 196.90 | 348.11 | 365.80 | -17.69 | -5.08 |
| 1-Butyl-3-methylimidazolium acetate | 200.70 | 397.97 | 373.11 | 24.86 | 6.25 |
| 2-Ethylhexyl acrylate | 201.00 | 369.00 | 373.68 | -4.68 | -1.27 |
| Diethyl phthalate | 204.20 | 379.14 | 379.83 | -0.69 | -0.18 |
| Methyl decanoate | 207.10 | 392.78 | 385.41 | 7.37 | 1.88 |
| 1,4-Di-t-butylbenzene | 213.60 | 370.02 | 397.90 | -27.88 | -7.54 |
| 1-Bromoundecane | 213.60 | 386.94 | 397.90 | -10.96 | -2.83 |
| 1-Butyl-3-methylimidazolium trifluoroacetate | 214.70 | 420.96 | 400.02 | 20.94 | 4.98 |
| Dibenzyldisulfide | 223.60 | 458.84 | 417.12 | 41.72 | 9.09 |
| Methyl undecylate | 223.80 | 421.86 | 417.51 | 4.35 | 1.03 |
| Ethyl decanoate | 223.80 | 418.04 | 417.51 | 0.53 | 0.13 |
| 1-Bromododecane | 230.30 | 416.30 | 430.00 | -13.70 | -3.29 |
| Octamethyltrisiloxane | 231.80 | 436.69 | 432.89 | 3.80 | 0.87 |
| Methyl laurate | 240.20 | 458.36 | 449.03 | 9.33 | 2.03 |
| Tetradecane | 244.90 | 452.30 | 458.07 | -5.77 | -1.28 |
| Octamethylcyclotetrasiloxane | 255.90 | 508.27 | 479.21 | 29.06 | 5.72 |
| Ethyl dodecanoate | 256.90 | 480.45 | 481.14 | -0.69 | -0.14 |
| 1,4,7,10,13,16-Hexaoxacyclooctadecane | 258.10 | 518.70 | 483.44 | 35.26 | 6.80 |
| 1-Bromotetradecane | 263.70 | 468.67 | 494.21 | -25.54 | -5.45 |
| 4-Cyano-3-fluorophenyl 4-butylbenzoate | 269.80 | 493.29 | 505.93 | -12.64 | -2.56 |
| Cetane | 278.20 | 517.90 | 522.08 | -4.18 | -0.81 |
| Ethyl myristate | 290.20 | 543.80 | 545.15 | -1.35 | -0.25 |
| Decamethyltetrasiloxane | 294.20 | 559.17 | 552.84 | 6.33 | 1.13 |
| Heptadecane | 294.90 | 550.62 | 554.18 | -3.56 | -0.65 |
| Octadecane | 311.60 | 582.54 | 586.28 | -3.74 | -0.64 |
| Decamethylcyclopentasiloxane | 318.70 | 646.49 | 599.93 | 46.56 | 7.20 |
| Ethyl stearate | 357.40 | 684.20 | 674.32 | 9.88 | 1.44 |
| 1-Decyl-3-methylimidazolium bis(trifluoromethanesulfonyl) amide | 401.30 | 775.50 | 758.71 | 16.79 | 2.16 |
| 1-Tetradecyl-3-methylimidazolium bis(trifluoromethanesulfonyl) amide | 469.30 | 912.10 | 889.43 | 22.67 | 2.49 |
| 1-Hexadecyl-3-methylimidazolium bis(trifluoromethanesulfonyl) amide | 501.50 | 976.50 | 951.32 | 25.18 | 2.58 |
| Dodecahydrosqualene | 506.50 | 904.82 | 960.93 | -56.11 | -6.20 |
