# Supplementary material for: Calculation of the Isobaric Heat Capacities of the Liquid and Solid Phase of Organic Compounds at and around 298.15 K Based on Their “True” Molecular Volume
Source: Molecules. 2019 Apr 24;24(8):1626. doi: 10.3390/molecules24081626 (PMC6514989; doi:10.3390/molecules24081626)
Supplement: Supplementary file 1 [file molecules-24-01626-s001.zip › S15. Experimental and Calculated Cp(liq,350) Data Table to Figure 15D.doc]

| **Molecule name** | **Molecular volume** | **Cp(liq,350) exp** | **Cp(liq,350) calc** | **Deviation** | **Dev. in %** |
| --- | --- | --- | --- | --- | --- |
| DMSO | 69.90 | 158.02 | 131.68 | 26.34 | 16.67 |
| 1-Methylimidazole | 77.00 | 157.40 | 145.49 | 11.91 | 7.56 |
| Propylenecarbonate | 88.50 | 192.85 | 167.87 | 24.98 | 12.95 |
| 2-Aminopyridine | 90.20 | 217.70 | 171.18 | 46.52 | 21.37 |
| Pentane | 94.90 | 186.40 | 180.32 | 6.08 | 3.26 |
| Methylbenzene | 100.00 | 172.96 | 190.25 | -17.29 | -9.99 |
| n-Propyl acetate | 106.90 | 211.01 | 203.67 | 7.34 | 3.48 |
| Ethyl propanoate | 107.00 | 216.29 | 203.87 | 12.42 | 5.74 |
| 2-Chloroaniline | 109.70 | 209.11 | 209.12 | -0.01 | 0.00 |
| 3-Chloroaniline | 109.80 | 213.01 | 209.31 | 3.70 | 1.74 |
| Hexane | 111.50 | 217.00 | 212.62 | 4.38 | 2.02 |
| N-Ethyl-2-pyrrolidone | 115.30 | 219.53 | 220.02 | -0.49 | -0.22 |
| Diethylcarbonate | 117.10 | 235.66 | 223.52 | 12.14 | 5.15 |
| 2-Chloronitrobenzene | 119.80 | 218.83 | 228.77 | -9.94 | -4.54 |
| 3-Chloronitrobenzene | 120.90 | 222.58 | 230.91 | -8.33 | -3.74 |
| Heptane | 128.20 | 246.26 | 245.12 | 1.14 | 0.46 |
| 3,4,5,6-Tetrahydrophthalic anhydride | 129.00 | 277.07 | 246.67 | 30.40 | 10.97 |
| Hexyl bromide | 130.10 | 236.69 | 248.81 | -12.12 | -5.12 |
| 1,4-Diisocyanatobutane | 130.10 | 237.28 | 248.81 | -11.53 | -4.86 |
| 1-Phenylpyrazole | 133.00 | 254.00 | 254.46 | -0.46 | -0.18 |
| 1-Phenylimidazole | 134.40 | 272.30 | 257.18 | 15.12 | 5.55 |
| Veratrole | 134.80 | 297.22 | 257.96 | 39.26 | 13.21 |
| Isoamyl acetate | 139.90 | 272.62 | 267.88 | 4.74 | 1.74 |
| Ethyl pentanoate | 140.10 | 273.94 | 268.27 | 5.67 | 2.07 |
| Methyl hexanoate | 140.30 | 260.37 | 268.66 | -8.29 | -3.18 |
| 4-Methyl-4-cyclohexene-1,2-dicarboxylic anhydride | 144.70 | 313.28 | 277.22 | 36.06 | 11.51 |
| Octane | 144.90 | 278.23 | 277.61 | 0.62 | 0.22 |
| N-(2-Cyanoethyl)aniline | 146.20 | 287.66 | 280.14 | 7.52 | 2.61 |
| Heptyl bromide | 146.80 | 268.66 | 281.31 | -12.65 | -4.71 |
| 2-Ethylhexanal | 147.00 | 288.23 | 281.70 | 6.53 | 2.27 |
| Camphene | 148.40 | 265.63 | 284.42 | -18.79 | -7.07 |
| t-Butylbenzene | 148.60 | 266.69 | 284.81 | -18.12 | -6.79 |
| Dipropyl carbonate | 150.50 | 296.45 | 288.51 | 7.94 | 2.68 |
| trans-Decahydroquinoline | 151.60 | 274.53 | 290.65 | -16.12 | -5.87 |
| Fenchone | 156.90 | 295.56 | 300.96 | -5.40 | -1.83 |
| 1-Bromooctane | 163.50 | 289.74 | 313.80 | -24.06 | -8.30 |
| 1,8-Cineole | 164.40 | 301.22 | 315.55 | -14.33 | -4.76 |
| Neopentylbenzene | 165.20 | 296.97 | 317.11 | -20.14 | -6.78 |
| Hexamethyldisiloxane | 168.30 | 332.18 | 323.14 | 9.04 | 2.72 |
| 3,4-Dimethoxyphenylacetonitrile | 168.90 | 356.00 | 324.31 | 31.69 | 8.90 |
| 4-Phenyltoluene | 169.90 | 305.80 | 326.25 | -20.45 | -6.69 |
| 2-Phenyltoluene | 170.50 | 296.21 | 327.42 | -31.21 | -10.54 |
| Ethyl trans-cinnamate | 172.90 | 314.20 | 332.09 | -17.89 | -5.69 |
| Ethyl heptanoate | 173.40 | 334.19 | 333.06 | 1.13 | 0.34 |
| Methyl octanoate | 173.40 | 334.05 | 333.06 | 0.99 | 0.30 |
| 1-Chlorononane | 176.00 | 318.00 | 338.12 | -20.12 | -6.33 |
| 1-Ethyladamantane | 177.70 | 295.79 | 341.43 | -45.64 | -15.43 |
| Tolan | 178.00 | 306.04 | 342.01 | -35.97 | -11.75 |
| 1,3-Dimethyladamantane | 178.20 | 294.11 | 342.40 | -48.29 | -16.42 |
| Decane | 178.20 | 341.38 | 342.40 | -1.02 | -0.30 |
| 1-Bromononane | 180.20 | 332.07 | 346.29 | -14.22 | -4.28 |
| 2,5,8,11-Tetraoxododecane | 183.40 | 378.77 | 352.52 | 26.25 | 6.93 |
| n-Octylacetate | 190.00 | 369.11 | 365.36 | 3.75 | 1.02 |
| Methyl nonanoate | 190.10 | 368.80 | 365.56 | 3.24 | 0.88 |
| Diphenyldisulfide | 190.90 | 343.00 | 367.11 | -24.11 | -7.03 |
| 2,4,6,8-Tetramethylcyclotetrasiloxane | 194.70 | 384.60 | 374.51 | 10.09 | 2.62 |
| Undecane | 194.90 | 373.20 | 374.90 | -1.70 | -0.45 |
| Hexamethylcyclotrisiloxane | 195.90 | 409.01 | 376.84 | 32.17 | 7.86 |
| 1-Bromodecane | 196.90 | 360.59 | 378.79 | -18.20 | -5.05 |
| 1-Butyl-3-methylimidazolium acetate | 200.70 | 413.81 | 386.18 | 27.63 | 6.68 |
| 2-Ethylhexyl acrylate | 201.00 | 386.00 | 386.77 | -0.77 | -0.20 |
| Diethyl phthalate | 204.20 | 393.40 | 392.99 | 0.41 | 0.10 |
| Methyl decanoate | 207.10 | 402.98 | 398.64 | 4.34 | 1.08 |
| 1,4-Di-t-butylbenzene | 213.60 | 389.72 | 411.28 | -21.56 | -5.53 |
| 1-Bromoundecane | 213.60 | 399.40 | 411.28 | -11.88 | -2.98 |
| 1-Butyl-3-methylimidazolium trifluoroacetate | 214.70 | 433.02 | 413.42 | 19.60 | 4.53 |
| 4-t-Butylbiphenyl | 218.20 | 397.60 | 420.23 | -22.64 | -5.69 |
| Methyl undecylate | 223.80 | 432.38 | 431.13 | 1.25 | 0.29 |
| 4-t-Butyldiphenyl ether | 227.80 | 403.25 | 438.91 | -35.66 | -8.84 |
| 1-Bromododecane | 230.30 | 431.74 | 443.78 | -12.04 | -2.79 |
| Octamethyltrisiloxane | 231.80 | 452.05 | 446.70 | 5.35 | 1.18 |
| Benzylferrocene | 232.50 | 430.10 | 448.06 | -17.96 | -4.18 |
| Methyl laurate | 240.20 | 469.53 | 463.04 | 6.49 | 1.38 |
| Tetradecane | 244.90 | 467.80 | 472.18 | -4.38 | -0.94 |
| Octamethylcyclotetrasiloxane | 255.90 | 522.69 | 493.59 | 29.10 | 5.57 |
| 1,4,7,10,13,16-Hexaoxacyclooctadecane | 258.10 | 527.80 | 497.87 | 29.93 | 5.67 |
| 1-Bromotetradecane | 263.70 | 484.87 | 508.77 | -23.90 | -4.93 |
| 4-Cyano-3-fluorophenyl 4-butylbenzoate | 269.80 | 509.84 | 520.63 | -10.79 | -2.12 |
| Cetane | 278.20 | 536.70 | 536.98 | -0.28 | -0.05 |
| Decamethyltetrasiloxane | 294.20 | 577.85 | 568.11 | 9.74 | 1.69 |
| Heptadecane | 294.90 | 568.89 | 569.47 | -0.58 | -0.10 |
| Octadecane | 311.60 | 601.38 | 601.97 | -0.59 | -0.10 |
| Decamethylcyclopentasiloxane | 318.70 | 660.80 | 615.78 | 45.02 | 6.81 |
| 1-Decyl-3-methylimidazolium bis(trifluoromethanesulfonyl) amide | 401.30 | 796.00 | 776.50 | 19.50 | 2.45 |
| 1-Tetradecyl-3-methylimidazolium bis(trifluoromethanesulfonyl) amide | 469.30 | 936.50 | 908.81 | 27.69 | 2.96 |
| 1-Hexadecyl-3-methylimidazolium bis(trifluoromethanesulfonyl) amide | 501.50 | 1000.00 | 971.46 | 28.54 | 2.85 |
| Dodecahydrosqualene | 506.50 | 937.38 | 981.19 | -43.81 | -4.67 |
