# Supplementary material for: Calculation of the Isobaric Heat Capacities of the Liquid and Solid Phase of Organic Compounds at and around 298.15 K Based on Their “True” Molecular Volume
Source: Molecules. 2019 Apr 24;24(8):1626. doi: 10.3390/molecules24081626 (PMC6514989; doi:10.3390/molecules24081626)
Supplement: Supplementary file 1 [file molecules-24-01626-s001.zip › S16. Experimental and Calculated Cp(liq,250) Data Table to Figure 16A.doc]

| **Molecule name** | **Molecular volume** | **Cp(liq,250) exp** | **Cp(liq,250) calc** | **Deviation** | **Dev. in %** |
| --- | --- | --- | --- | --- | --- |
| Methanol | 36.90 | 74.06 | 70.47 | 3.59 | 4.84 |
| Ethanol | 53.60 | 97.68 | 98.98 | -1.30 | -1.33 |
| 1-Propanol | 70.30 | 122.43 | 127.49 | -5.06 | -4.13 |
| 1-Butanol | 87.00 | 151.38 | 156.00 | -4.62 | -3.05 |
| 1-Pentanol | 103.60 | 179.07 | 184.33 | -5.26 | -2.94 |
| 1-Hexanol | 120.60 | 207.60 | 213.35 | -5.75 | -2.77 |
| 2-t-Butoxyethanol | 129.40 | 244.90 | 228.37 | 16.53 | 6.75 |
| Butylcellosolve | 130.20 | 251.33 | 229.74 | 21.59 | 8.59 |
| 1-Heptanol | 137.30 | 241.00 | 241.86 | -0.86 | -0.36 |
| 1-Octanol | 153.70 | 251.00 | 269.86 | -18.86 | -7.51 |
