# Supplementary material for: Calculation of the Isobaric Heat Capacities of the Liquid and Solid Phase of Organic Compounds at and around 298.15 K Based on Their “True” Molecular Volume
Source: Molecules. 2019 Apr 24;24(8):1626. doi: 10.3390/molecules24081626 (PMC6514989; doi:10.3390/molecules24081626)
Supplement: Supplementary file 1 [file molecules-24-01626-s001.zip › S17. Experimental and Calculated Cp(liq,275) Data Table to Figure 16B.doc]

| **Molecule name** | **Molecular volume** | **Cp(liq,275) exp** | **Cp(liq,275) calc** | **Deviation** | **Dev. in %** |
| --- | --- | --- | --- | --- | --- |
| Methanol | 36.90 | 77.23 | 73.39 | 3.84 | 4.97 |
| Ethanol | 53.60 | 104.27 | 106.57 | -2.30 | -2.21 |
| Allyl alcohol | 65.10 | 135.44 | 129.42 | 6.02 | 4.45 |
| 1-Propanol | 70.30 | 132.17 | 139.75 | -7.58 | -5.73 |
| Cyclobutanol | 78.10 | 143.12 | 155.24 | -12.12 | -8.47 |
| 1-Butanol | 87.00 | 162.83 | 172.92 | -10.09 | -6.20 |
| Cyclopentanol | 93.40 | 164.20 | 185.64 | -21.44 | -13.05 |
| 2-(Ethylamino)ethanol | 99.40 | 218.80 | 197.55 | 21.25 | 9.71 |
| 1-Pentanol | 103.60 | 192.03 | 205.90 | -13.87 | -7.22 |
| cis-3-Hexen-1-ol | 114.30 | 213.90 | 227.15 | -13.25 | -6.20 |
| 1-Hexen-3-ol | 114.60 | 246.00 | 227.75 | 18.25 | 7.42 |
| trans-3-Hexen-1-ol | 114.60 | 218.40 | 227.75 | -9.35 | -4.28 |
| 2-(Isopropylamino)ethanol | 115.50 | 245.80 | 229.54 | 16.26 | 6.62 |
| 1-Hexanol | 120.60 | 222.00 | 239.67 | -17.67 | -7.96 |
| Cyclohexanemethanol | 125.30 | 212.45 | 249.01 | -36.56 | -17.21 |
| 2-t-Butoxyethanol | 129.40 | 259.35 | 257.15 | 2.20 | 0.85 |
| Butylcellosolve | 130.20 | 261.25 | 258.74 | 2.51 | 0.96 |
| 2-Diethylaminoethanol | 130.80 | 267.10 | 259.93 | 7.17 | 2.68 |
| Triethylmethanol | 135.50 | 318.19 | 269.27 | 48.92 | 15.37 |
| 2,4-Dimethyl-3-pentanol | 135.90 | 311.96 | 270.06 | 41.90 | 13.43 |
| 2-Methyl-3-hexanol | 136.70 | 288.35 | 271.65 | 16.70 | 5.79 |
| 2-Methyl-2-hexanol | 136.70 | 279.78 | 271.65 | 8.13 | 2.90 |
| 5-Methyl-2-hexanol | 136.80 | 268.64 | 271.85 | -3.21 | -1.20 |
| 3-Heptanol | 137.20 | 289.76 | 272.65 | 17.11 | 5.91 |
| 4-Heptanol | 137.20 | 289.34 | 272.65 | 16.69 | 5.77 |
| 1-Heptanol | 137.30 | 255.95 | 272.85 | -16.90 | -6.60 |
| Cyclohexaneethanol | 142.50 | 241.95 | 283.18 | -41.23 | -17.04 |
| 4-Ethyl-3-hexanol | 152.20 | 315.45 | 302.45 | 13.00 | 4.12 |
| 3-Methyl-4-heptanol | 152.80 | 316.53 | 303.64 | 12.89 | 4.07 |
| 2,5-Dimethyl-3-hexanol | 152.90 | 339.40 | 303.84 | 35.56 | 10.48 |
| 4-Methyl-4-heptanol | 153.00 | 319.77 | 304.04 | 15.73 | 4.92 |
| 1-Octanol | 153.70 | 285.00 | 305.43 | -20.43 | -7.17 |
| 3-Octanol | 153.90 | 321.60 | 305.82 | 15.78 | 4.91 |
| Cyclohexanepropanol | 159.20 | 268.99 | 316.35 | -47.36 | -17.61 |
| trans-Geraniol | 172.60 | 322.20 | 342.97 | -20.77 | -6.45 |
| beta-Citronellol | 179.30 | 330.50 | 356.28 | -25.78 | -7.80 |
