# Supplementary material for: Calculation of the Isobaric Heat Capacities of the Liquid and Solid Phase of Organic Compounds at and around 298.15 K Based on Their “True” Molecular Volume
Source: Molecules. 2019 Apr 24;24(8):1626. doi: 10.3390/molecules24081626 (PMC6514989; doi:10.3390/molecules24081626)
Supplement: Supplementary file 1 [file molecules-24-01626-s001.zip › S18. Experimental and Calculated Cp(liq,325) Data Table to Figure 16C.doc]

| **Molecule name** | **Molecular volume** | **Cp(liq,325) exp** | **Cp(liq,325) calc** | **Deviation** | **Dev. in %** |
| --- | --- | --- | --- | --- | --- |
| Methanol | 36.90 | 86.95 | 110.59 | -23.64 | -27.19 |
| Ethanol | 53.60 | 124.26 | 144.49 | -20.23 | -16.28 |
| Ethan-1,2-diol | 62.70 | 158.15 | 162.96 | -4.81 | -3.04 |
| Allyl alcohol | 65.10 | 160.47 | 167.83 | -7.36 | -4.59 |
| 1-Propanol | 70.30 | 160.86 | 178.38 | -17.52 | -10.89 |
| Cyclobutanol | 78.10 | 171.60 | 194.21 | -22.61 | -13.18 |
| Propan-1,3-diol | 79.40 | 188.65 | 196.85 | -8.20 | -4.35 |
| Methyl cellosolve | 80.10 | 183.85 | 198.27 | -14.42 | -7.85 |
| 1-Butanol | 87.00 | 198.32 | 212.28 | -13.96 | -7.04 |
| Cyclopentanol | 93.40 | 206.20 | 225.27 | -19.07 | -9.25 |
| Tetramethylene glycol | 96.10 | 218.83 | 230.75 | -11.92 | -5.45 |
| 2-Ethoxyethanol | 96.90 | 221.04 | 232.37 | -11.33 | -5.13 |
| 2-(Ethylamino)ethanol | 99.40 | 239.50 | 237.45 | 2.05 | 0.86 |
| 1-Pentanol | 103.60 | 231.90 | 245.97 | -14.07 | -6.07 |
| Cyclohexanol | 109.50 | 241.30 | 257.95 | -16.65 | -6.90 |
| 1,5-Pentanediol | 112.80 | 252.30 | 264.64 | -12.34 | -4.89 |
| 2-Propoxyethanol | 113.60 | 252.80 | 266.27 | -13.47 | -5.33 |
| cis-3-Hexen-1-ol | 114.30 | 255.20 | 267.69 | -12.49 | -4.89 |
| 1-Hexen-3-ol | 114.60 | 289.10 | 268.30 | 20.80 | 7.20 |
| trans-3-Hexen-1-ol | 114.60 | 257.80 | 268.30 | -10.50 | -4.07 |
| 2-(Isopropylamino)ethanol | 115.50 | 277.20 | 270.12 | 7.08 | 2.55 |
| 1-Hexanol | 120.60 | 268.40 | 280.47 | -12.07 | -4.50 |
| s-Phenethyl alcohol | 124.20 | 276.94 | 287.78 | -10.84 | -3.91 |
| Cyclohexanemethanol | 125.30 | 265.56 | 290.01 | -24.45 | -9.21 |
| Cycloheptanol | 125.50 | 280.30 | 290.42 | -10.12 | -3.61 |
| Hexamethyleneglycol | 129.50 | 288.99 | 298.54 | -9.55 | -3.30 |
| 2-Diethylaminoethanol | 130.80 | 295.80 | 301.18 | -5.38 | -1.82 |
| Triethylmethanol | 135.50 | 373.15 | 310.72 | 62.43 | 16.73 |
| 2,4-Dimethyl-3-pentanol | 135.90 | 382.38 | 311.53 | 70.85 | 18.53 |
| 2,2-Dimethyl-3-pentanol | 135.90 | 376.65 | 311.53 | 65.12 | 17.29 |
| 2-Methyl-3-hexanol | 136.70 | 358.69 | 313.15 | 45.54 | 12.70 |
| 2-Methyl-2-hexanol | 136.70 | 349.37 | 313.15 | 36.22 | 10.37 |
| 5-Methyl-2-hexanol | 136.80 | 324.68 | 313.36 | 11.32 | 3.49 |
| 3-Heptanol | 137.20 | 345.22 | 314.17 | 31.05 | 9.00 |
| 4-Heptanol | 137.20 | 345.13 | 314.17 | 30.96 | 8.97 |
| 1-Heptanol | 137.30 | 301.55 | 314.37 | -12.82 | -4.25 |
| Cyclohexaneethanol | 142.50 | 293.27 | 324.92 | -31.65 | -10.79 |
| Cyclooctanol | 142.50 | 320.20 | 324.92 | -4.72 | -1.48 |
| 1,7-Heptanediol | 146.20 | 322.54 | 332.43 | -9.89 | -3.07 |
| 4-Ethyl-3-hexanol | 152.20 | 392.85 | 344.61 | 48.24 | 12.28 |
| 3-Methyl-4-heptanol | 152.80 | 388.95 | 345.83 | 43.12 | 11.09 |
| 2,5-Dimethyl-3-hexanol | 152.90 | 406.57 | 346.03 | 60.54 | 14.89 |
| 4-Methyl-4-heptanol | 153.00 | 401.59 | 346.24 | 55.35 | 13.78 |
| 1-Octanol | 153.70 | 336.00 | 347.66 | -11.66 | -3.47 |
| 3-Octanol | 153.90 | 375.23 | 348.06 | 27.17 | 7.24 |
| Cyclohexanepropanol | 159.20 | 324.00 | 358.82 | -34.82 | -10.75 |
| 1-Nonanol | 170.70 | 370.70 | 382.16 | -11.46 | -3.09 |
| trans-Geraniol | 172.60 | 370.60 | 386.02 | -15.42 | -4.16 |
| Menthol | 174.80 | 390.80 | 390.48 | 0.32 | 0.08 |
| beta-Citronellol | 179.30 | 386.50 | 399.62 | -13.12 | -3.39 |
| 1,9-Nonanediol | 179.60 | 397.51 | 400.22 | -2.71 | -0.68 |
| n-Decyl alcohol | 187.00 | 404.95 | 415.24 | -10.29 | -2.54 |
| n-Decanoic acid | 189.90 | 374.26 | 421.13 | -46.87 | -12.52 |
| 1-Undecanol | 204.10 | 448.00 | 449.95 | -1.95 | -0.44 |
| 1-Dodecanol | 220.80 | 474.60 | 483.85 | -9.25 | -1.95 |
| 1-Tridecanol | 237.50 | 510.50 | 517.74 | -7.24 | -1.42 |
| Myristyl alcohol | 254.20 | 541.50 | 551.64 | -10.14 | -1.87 |
| 1-Pentadecanol | 270.90 | 561.50 | 585.53 | -24.03 | -4.28 |
| 1-Hexadecanol | 287.60 | 586.30 | 619.43 | -33.13 | -5.65 |
