# Supplementary material for: Calculation of the Isobaric Heat Capacities of the Liquid and Solid Phase of Organic Compounds at and around 298.15 K Based on Their “True” Molecular Volume
Source: Molecules. 2019 Apr 24;24(8):1626. doi: 10.3390/molecules24081626 (PMC6514989; doi:10.3390/molecules24081626)
Supplement: Supplementary file 1 [file molecules-24-01626-s001.zip › S19. Experimental and Calculated Cp(liq,350) Data Table to Figure 16D.doc]

| **Molecule name** | **Molecular volume** | **Cp(liq,350) exp** | **Cp(liq,350) calc** | **Deviation** | **Dev. in %** |
| --- | --- | --- | --- | --- | --- |
| Methanol | 36.90 | 93.71 | 119.37 | -25.66 | -27.38 |
| Ethanol | 53.60 | 137.66 | 155.50 | -17.84 | -12.96 |
| Ethan-1,2-diol | 62.70 | 166.35 | 175.19 | -8.84 | -5.32 |
| 1-Propanol | 70.30 | 179.50 | 191.64 | -12.14 | -6.76 |
| Cyclobutanol | 78.10 | 191.60 | 208.52 | -16.92 | -8.83 |
| Propan-1,3-diol | 79.40 | 201.37 | 211.33 | -9.96 | -4.95 |
| 1-Butanol | 87.00 | 219.46 | 227.78 | -8.32 | -3.79 |
| Cyclopentanol | 93.40 | 228.10 | 241.63 | -13.53 | -5.93 |
| Tetramethylene glycol | 96.10 | 235.34 | 247.47 | -12.13 | -5.15 |
| 2-(Ethylamino)ethanol | 99.40 | 252.90 | 254.61 | -1.71 | -0.68 |
| 1-Pentanol | 103.60 | 255.45 | 263.70 | -8.25 | -3.23 |
| Cyclohexanol | 109.50 | 268.00 | 276.46 | -8.46 | -3.16 |
| 1,5-Pentanediol | 112.80 | 273.78 | 283.60 | -9.82 | -3.59 |
| cis-3-Hexen-1-ol | 114.30 | 274.60 | 286.85 | -12.25 | -4.46 |
| 1-Hexen-3-ol | 114.60 | 303.80 | 287.50 | 16.30 | 5.37 |
| trans-3-Hexen-1-ol | 114.60 | 276.90 | 287.50 | -10.60 | -3.83 |
| 2-(Isopropylamino)ethanol | 115.50 | 294.90 | 289.45 | 5.45 | 1.85 |
| 1-Hexanol | 120.60 | 295.20 | 300.48 | -5.28 | -1.79 |
| s-Phenethyl alcohol | 124.20 | 288.84 | 308.27 | -19.43 | -6.73 |
| Cyclohexanemethanol | 125.30 | 291.51 | 310.65 | -19.14 | -6.57 |
| Cycloheptanol | 125.50 | 303.60 | 311.08 | -7.48 | -2.47 |
| Hexamethyleneglycol | 129.50 | 314.84 | 319.74 | -4.90 | -1.56 |
| 2-Diethylaminoethanol | 130.80 | 307.70 | 322.55 | -14.85 | -4.83 |
| 8-Quinolinol | 130.90 | 292.20 | 322.77 | -30.57 | -10.46 |
| Triethylmethanol | 135.50 | 364.00 | 332.72 | 31.28 | 8.59 |
| 2,4-Dimethyl-3-pentanol | 135.90 | 374.57 | 333.59 | 40.98 | 10.94 |
| 2,2-Dimethyl-3-pentanol | 135.90 | 374.32 | 333.59 | 40.73 | 10.88 |
| 2-Methyl-3-hexanol | 136.70 | 372.49 | 335.32 | 37.17 | 9.98 |
| 2-Methyl-2-hexanol | 136.70 | 370.24 | 335.32 | 34.92 | 9.43 |
| 5-Methyl-2-hexanol | 136.80 | 349.62 | 335.54 | 14.08 | 4.03 |
| 4-Heptanol | 137.20 | 368.58 | 336.40 | 32.18 | 8.73 |
| 3-Heptanol | 137.20 | 365.75 | 336.40 | 29.35 | 8.02 |
| 1-Heptanol | 137.30 | 332.40 | 336.62 | -4.22 | -1.27 |
| Cyclohexaneethanol | 142.50 | 320.60 | 347.87 | -27.27 | -8.51 |
| Cyclooctanol | 142.50 | 343.00 | 347.87 | -4.87 | -1.42 |
| 1,7-Heptanediol | 146.20 | 350.87 | 355.88 | -5.01 | -1.43 |
| 4-Ethyl-3-hexanol | 152.20 | 405.08 | 368.86 | 36.22 | 8.94 |
| 3-Methyl-4-heptanol | 152.80 | 405.49 | 370.16 | 35.33 | 8.71 |
| 2,5-Dimethyl-3-hexanol | 152.90 | 419.80 | 370.37 | 49.43 | 11.77 |
| 4-Methyl-4-heptanol | 153.00 | 417.89 | 370.59 | 47.30 | 11.32 |
| 1-Octanol | 153.70 | 368.20 | 372.11 | -3.91 | -1.06 |
| 3-Octanol | 153.90 | 396.68 | 372.54 | 24.14 | 6.09 |
| Cyclohexanepropanol | 159.20 | 354.61 | 384.01 | -29.40 | -8.29 |
| 1,8-Octanediol | 162.90 | 392.32 | 392.01 | 0.31 | 0.08 |
| 1-Nonanol | 170.70 | 404.70 | 408.89 | -4.19 | -1.04 |
| trans-Geraniol | 172.60 | 394.30 | 413.00 | -18.70 | -4.74 |
| Menthol | 174.80 | 422.30 | 417.76 | 4.54 | 1.07 |
| beta-Citronellol | 179.30 | 414.60 | 427.50 | -12.90 | -3.11 |
| 1,9-Nonanediol | 179.60 | 432.87 | 428.15 | 4.72 | 1.09 |
| n-Decyl alcohol | 187.00 | 436.50 | 444.16 | -7.66 | -1.76 |
| 1-Undecanol | 204.10 | 481.00 | 481.16 | -0.16 | -0.03 |
| 1-Dodecanol | 220.80 | 507.00 | 517.30 | -10.30 | -2.03 |
| 1-Tridecanol | 237.50 | 544.00 | 553.44 | -9.44 | -1.73 |
| Myristyl alcohol | 254.20 | 574.00 | 589.57 | -15.57 | -2.71 |
| 1-Pentadecanol | 270.90 | 591.00 | 625.71 | -34.71 | -5.87 |
| 1-Hexadecanol | 287.60 | 643.80 | 661.84 | -18.04 | -2.80 |
