# Supplementary material for: Calculation of the Isobaric Heat Capacities of the Liquid and Solid Phase of Organic Compounds at and around 298.15 K Based on Their “True” Molecular Volume
Source: Molecules. 2019 Apr 24;24(8):1626. doi: 10.3390/molecules24081626 (PMC6514989; doi:10.3390/molecules24081626)
Supplement: Supplementary file 1 [file molecules-24-01626-s001.zip › S20. Experimental and Calculated Cp(sol,250) Data Table to Figure 17A.doc]

| **Molecule name** | **Molecular volume** | **Cp(sol,250) exp** | **Cp(sol,250) calc** | **Deviation** | **Dev. in %** |
| --- | --- | --- | --- | --- | --- |
| Urea | 53.90 | 80.13 | 71.16 | 8.97 | 11.19 |
| Imidazole | 61.20 | 72.14 | 79.61 | -7.47 | -10.35 |
| Methylurea | 70.20 | 99.28 | 90.02 | 9.26 | 9.33 |
| Oxalamide | 73.20 | 96.68 | 93.49 | 3.19 | 3.30 |
| Pyrazine | 74.30 | 86.03 | 94.76 | -8.73 | -10.15 |
| 1-Methylimidazole | 77.00 | 100.00 | 97.89 | 2.11 | 2.11 |
| 2-Methylimidazole | 78.00 | 88.28 | 99.04 | -10.76 | -12.19 |
| Ethylurea | 87.50 | 126.60 | 110.03 | 16.57 | 13.08 |
| N,N-Dimethylurea | 87.60 | 120.70 | 110.15 | 10.55 | 8.74 |
| N,N'-Dimethylurea | 87.90 | 117.40 | 110.50 | 6.90 | 5.88 |
| Malonamide | 89.30 | 115.75 | 112.12 | 3.63 | 3.14 |
| 2-Aminopyridine | 90.20 | 120.00 | 113.16 | 6.84 | 5.70 |
| 2-Ethylimidazole | 93.30 | 115.08 | 116.74 | -1.66 | -1.45 |
| Isopropylurea | 104.20 | 141.50 | 129.35 | 12.15 | 8.58 |
| Succindiamide | 105.80 | 135.73 | 131.21 | 4.52 | 3.33 |
| 1,3,5-Trithiane | 106.70 | 118.06 | 132.25 | -14.19 | -12.02 |
| 1,4-Dichlorobenzene | 111.90 | 124.06 | 138.26 | -14.20 | -11.45 |
| 2-Amino-5-nitropyridine | 113.50 | 135.50 | 140.11 | -4.61 | -3.41 |
| t-Butylurea | 119.80 | 163.00 | 147.40 | 15.60 | 9.57 |
| N,N-Diethylurea | 119.80 | 160.70 | 147.40 | 13.30 | 8.27 |
| 1,4-Dibromobenzene | 120.20 | 127.67 | 147.87 | -20.20 | -15.82 |
| sec-Butylurea | 120.30 | 165.20 | 147.98 | 17.22 | 10.42 |
| Tetramethylurea | 120.50 | 208.00 | 148.21 | 59.79 | 28.74 |
| n-Butylurea | 120.90 | 169.50 | 148.68 | 20.82 | 12.29 |
| N,N'-Diethylurea | 121.40 | 192.90 | 149.25 | 43.65 | 22.63 |
| Glutaramide | 122.50 | 144.92 | 150.53 | -5.61 | -3.87 |
| 4-Dimethylaminopyridine | 123.10 | 155.28 | 151.22 | 4.06 | 2.61 |
| 1,3,5-Trichlorobenzene | 126.10 | 139.83 | 154.69 | -14.86 | -10.63 |
| 3,4,5,6-Tetrahydrophthalic anhydride | 129.00 | 136.24 | 158.05 | -21.81 | -16.01 |
| 1,3,5-Tribromobenzene | 138.60 | 144.67 | 169.15 | -24.48 | -16.92 |
| Adipamide | 139.20 | 175.41 | 169.85 | 5.56 | 3.17 |
| Acenaphthylene | 141.00 | 152.72 | 171.93 | -19.21 | -12.58 |
| 4-Methyl-4-cyclohexene-1,2-dicarboxylic anhydride | 144.70 | 160.56 | 176.21 | -15.65 | -9.75 |
| Methyl 4-methylbenzoate | 145.00 | 172.77 | 176.56 | -3.79 | -2.19 |
| 4,4'-Bipyridine | 145.30 | 155.30 | 176.90 | -21.60 | -13.91 |
| N-(2-Cyanoethyl)aniline | 146.20 | 147.99 | 177.94 | -29.95 | -20.24 |
| Acenaphthene | 147.10 | 155.44 | 178.99 | -23.55 | -15.15 |
| trans-Decahydroquinoline | 151.60 | 159.34 | 184.19 | -24.85 | -15.60 |
| Camphor | 154.40 | 229.00 | 187.43 | 41.57 | 18.15 |
| Pimelamide | 155.40 | 197.84 | 188.59 | 9.25 | 4.68 |
| 2-Chloro-N,N-dimethylnicotinamide | 155.90 | 221.10 | 189.17 | 31.93 | 14.44 |
| 2-(Chloromethylthio)benzothiazole | 161.00 | 224.50 | 195.07 | 29.43 | 13.11 |
| 1-Bromoadamantane | 163.30 | 175.52 | 197.73 | -22.21 | -12.65 |
| Neopentylbenzene | 165.20 | 196.13 | 199.93 | -3.80 | -1.94 |
| Caffeine | 167.10 | 203.16 | 202.12 | 1.04 | 0.51 |
| 3,4-Dimethoxyphenylacetonitrile | 168.90 | 208.40 | 204.21 | 4.19 | 2.01 |
| 4-Phenyltoluene | 169.90 | 197.73 | 205.36 | -7.63 | -3.86 |
| 2-Phenyltoluene | 170.50 | 180.78 | 206.06 | -25.28 | -13.98 |
| Benzophenone | 170.90 | 185.50 | 206.52 | -21.02 | -11.33 |
| Suberamide | 171.60 | 210.82 | 207.33 | 3.49 | 1.66 |
| Monuron | 172.00 | 220.80 | 207.79 | 13.01 | 5.89 |
| Tolan | 178.00 | 186.45 | 214.73 | -28.28 | -15.17 |
| Tryptophane | 182.20 | 192.81 | 219.59 | -26.78 | -13.89 |
| Acetylferrocene | 183.30 | 204.60 | 220.87 | -16.27 | -7.95 |
| 1,3-Di-t-butyl urea | 185.80 | 253.00 | 223.76 | 29.24 | 11.56 |
| 3,3'-Bitolyl | 186.20 | 198.25 | 224.22 | -25.98 | -13.10 |
| 4,4'-Dimethylbiphenyl | 186.20 | 204.70 | 224.22 | -19.52 | -9.54 |
| Octyl urea | 187.70 | 231.60 | 225.96 | 5.64 | 2.44 |
| Azelamide | 188.30 | 231.45 | 226.65 | 4.80 | 2.07 |
| Sebacamide | 206.00 | 250.22 | 247.13 | 3.09 | 1.24 |
| Isoproturon | 209.40 | 245.60 | 251.06 | -5.46 | -2.22 |
| 1,4-Di-t-butylbenzene | 213.60 | 269.68 | 255.92 | 13.76 | 5.10 |
| 4-t-Butylbiphenyl | 218.20 | 255.34 | 261.24 | -5.90 | -2.31 |
| 1,1'-Diacetylferrocene | 218.50 | 251.00 | 261.59 | -10.59 | -4.22 |
| t-Butyl 1-phenylethylcarbamate | 220.70 | 258.29 | 264.13 | -5.84 | -2.26 |
| Undecanediamide | 221.70 | 272.00 | 265.29 | 6.71 | 2.47 |
| 4-t-Butyldiphenyl ether | 227.80 | 257.50 | 272.35 | -14.85 | -5.77 |
| Benzylferrocene | 232.50 | 255.90 | 277.78 | -21.88 | -8.55 |
| Benzoylferrocene | 234.80 | 261.70 | 280.45 | -18.75 | -7.16 |
| Dodecanediamide | 238.40 | 278.82 | 284.61 | -5.79 | -2.08 |
| 4-Nitro-4'-t-butyl-diphenylether | 251.00 | 300.90 | 299.19 | 1.71 | 0.57 |
| Tetradecanediamide | 272.90 | 325.66 | 324.52 | 1.14 | 0.35 |
| 4,4'-Di-t-butylbiphenyl | 282.80 | 352.70 | 335.98 | 16.72 | 4.74 |
| Ethyl stearate | 357.40 | 472.00 | 422.28 | 49.72 | 10.53 |
| Dibenzo-24-crown-8-ether | 411.90 | 487.00 | 485.33 | 1.67 | 0.34 |
| N-Formylmethionine-leucyl-phenylalanine methyl ether | 418.10 | 506.50 | 492.50 | 14.00 | 2.76 |
| Buckminsterfullerene | 423.30 | 551.70 | 498.52 | 53.18 | 9.64 |
| Hexatriacontane | 612.70 | 720.70 | 717.64 | 3.06 | 0.43 |
| Tetratetracontane | 746.30 | 862.50 | 872.20 | -9.70 | -1.12 |
| Octaphenylpentacyclosilsesquioxane | 792.40 | 928.80 | 925.53 | 3.27 | 0.35 |
| Pentacontane | 846.50 | 964.50 | 988.12 | -23.62 | -2.45 |
