# Supplementary material for: Calculation of the Isobaric Heat Capacities of the Liquid and Solid Phase of Organic Compounds at and around 298.15 K Based on Their “True” Molecular Volume
Source: Molecules. 2019 Apr 24;24(8):1626. doi: 10.3390/molecules24081626 (PMC6514989; doi:10.3390/molecules24081626)
Supplement: Supplementary file 1 [file molecules-24-01626-s001.zip › S21. Experimental and Calculated Cp(sol,275) Data Table to Figure 17B.doc]

| **Molecule name** | **Molecular volume** | **Cp(sol,275) exp** | **Cp(sol,275) calc** | **Deviation** | **Dev. in %** |
| --- | --- | --- | --- | --- | --- |
| Urea | 53.90 | 86.86 | 73.75 | 13.11 | 15.09 |
| Imidazole | 61.20 | 78.31 | 82.99 | -4.68 | -5.98 |
| Methylurea | 70.20 | 106.20 | 94.38 | 11.82 | 11.13 |
| Oxalamide | 73.20 | 103.38 | 98.18 | 5.20 | 5.03 |
| Trifluoroacetamide | 73.30 | 98.43 | 98.31 | 0.12 | 0.12 |
| Pyrazine | 74.30 | 94.34 | 99.57 | -5.23 | -5.55 |
| 2-Methylimidazole | 78.00 | 96.13 | 104.26 | -8.13 | -8.46 |
| 1,3-Oxazolidine-2-thione | 84.90 | 109.50 | 112.99 | -3.49 | -3.19 |
| N,N-Dimethylurea | 87.60 | 127.70 | 116.41 | 11.29 | 8.84 |
| N,N'-Dimethylurea | 87.90 | 125.55 | 116.79 | 8.76 | 6.98 |
| Malonamide | 89.30 | 125.02 | 118.56 | 6.46 | 5.16 |
| 2-Aminopyridine | 90.20 | 130.71 | 119.70 | 11.01 | 8.42 |
| 2-Ethylimidazole | 93.30 | 122.97 | 123.63 | -0.66 | -0.53 |
| 2-Mercapto-2-thiazoline | 94.20 | 118.30 | 124.77 | -6.47 | -5.47 |
| Tetrahydro-2H-1,3-oxazine-2-thione | 102.00 | 128.10 | 134.64 | -6.54 | -5.11 |
| Isopropylurea | 104.20 | 153.75 | 137.42 | 16.33 | 10.62 |
| Thiobarbituric acid | 108.60 | 128.80 | 142.99 | -14.19 | -11.02 |
| 1,4-Dichlorobenzene | 111.90 | 133.86 | 147.17 | -13.31 | -9.94 |
| 2-Amino-5-nitropyridine | 113.50 | 147.50 | 149.20 | -1.70 | -1.15 |
| t-Butylurea | 119.80 | 175.00 | 157.17 | 17.83 | 10.19 |
| N,N-Diethylurea | 119.80 | 172.25 | 157.17 | 15.08 | 8.75 |
| 1,4-Dibromobenzene | 120.20 | 137.19 | 157.68 | -20.49 | -14.93 |
| sec-Butylurea | 120.30 | 178.90 | 157.81 | 21.09 | 11.79 |
| 1,3-Benzoxazole-2-thione | 120.30 | 144.30 | 157.81 | -13.51 | -9.36 |
| n-Butylurea | 120.90 | 184.15 | 158.57 | 25.58 | 13.89 |
| N,N'-Diethylurea | 121.40 | 208.20 | 159.20 | 49.00 | 23.54 |
| Glutaramide | 122.50 | 158.81 | 160.59 | -1.78 | -1.12 |
| 2-Thiobenzoxazole | 122.70 | 144.30 | 160.84 | -16.54 | -11.47 |
| 4-Dimethylaminopyridine | 123.10 | 168.04 | 161.35 | 6.69 | 3.98 |
| 1,3,5-Trichlorobenzene | 126.10 | 150.20 | 165.15 | -14.95 | -9.95 |
| 3,4,5,6-Tetrahydrophthalic anhydride | 129.00 | 163.39 | 168.82 | -5.43 | -3.32 |
| 1,3-Benzothiazole-2-thione | 129.20 | 146.70 | 169.07 | -22.37 | -15.25 |
| 1,3,5-Tribromobenzene | 138.60 | 153.95 | 180.97 | -27.02 | -17.55 |
| Adipamide | 139.20 | 188.80 | 181.73 | 7.07 | 3.74 |
| 4-Methyl-4-cyclohexene-1,2-dicarboxylic anhydride | 144.70 | 183.80 | 188.69 | -4.89 | -2.66 |
| Methyl 4-methylbenzoate | 145.00 | 187.08 | 189.07 | -1.99 | -1.07 |
| 4,4'-Bipyridine | 145.30 | 167.50 | 189.45 | -21.95 | -13.11 |
| N-(2-Cyanoethyl)aniline | 146.20 | 162.95 | 190.59 | -27.64 | -16.96 |
| Camphene | 148.40 | 220.70 | 193.38 | 27.32 | 12.38 |
| 3,4-Dimethoxybenzaldehyde | 154.10 | 198.20 | 200.59 | -2.39 | -1.21 |
| Camphor | 154.40 | 236.70 | 200.97 | 35.73 | 15.09 |
| Pimelamide | 155.40 | 217.02 | 202.24 | 14.78 | 6.81 |
| 4-Methoxycinnamaldehyde | 155.50 | 203.40 | 202.37 | 1.03 | 0.51 |
| 2-Chloro-N,N-dimethylnicotinamide | 155.90 | 240.50 | 202.87 | 37.63 | 15.65 |
| 2-(Chloromethylthio)benzothiazole | 161.00 | 236.80 | 209.33 | 27.47 | 11.60 |
| 1-Bromoadamantane | 163.30 | 199.50 | 212.24 | -12.74 | -6.39 |
| Barbital | 164.30 | 215.90 | 213.51 | 2.39 | 1.11 |
| Caffeine | 167.10 | 214.51 | 217.05 | -2.54 | -1.18 |
| 3,4-Dimethoxyphenylacetonitrile | 168.90 | 225.20 | 219.33 | 5.87 | 2.61 |
| 4-Phenyltoluene | 169.90 | 218.05 | 220.59 | -2.54 | -1.17 |
| Benzophenone | 170.90 | 205.50 | 221.86 | -16.36 | -7.96 |
| Suberamide | 171.60 | 234.06 | 222.75 | 11.31 | 4.83 |
| 2-Phenylbenzimidazole | 171.90 | 189.51 | 223.13 | -33.62 | -17.74 |
| Monuron | 172.00 | 238.60 | 223.25 | 15.35 | 6.43 |
| 1,3-Diethyl-2-thiobarbituric acid | 172.70 | 221.20 | 224.14 | -2.94 | -1.33 |
| Tolan | 178.00 | 205.42 | 230.85 | -25.43 | -12.38 |
| Tryptophane | 182.20 | 211.89 | 236.17 | -24.28 | -11.46 |
| 1,3-Di-t-butyl urea | 185.80 | 275.25 | 240.72 | 34.53 | 12.54 |
| 4,4'-Dimethylbiphenyl | 186.20 | 224.20 | 241.23 | -17.03 | -7.60 |
| Octyl urea | 187.70 | 259.85 | 243.13 | 16.72 | 6.44 |
| Azelamide | 188.30 | 253.07 | 243.89 | 9.18 | 3.63 |
| 2-Benzylbenzimidazole | 190.80 | 220.51 | 247.05 | -26.54 | -12.04 |
| Diphenyldisulfide | 190.90 | 219.14 | 247.18 | -28.04 | -12.79 |
| Sebacamide | 206.00 | 272.67 | 266.29 | 6.38 | 2.34 |
| Isoproturon | 209.40 | 267.50 | 270.60 | -3.10 | -1.16 |
| 4,4'-Diaminodiphenyldisulfide | 213.70 | 271.32 | 276.04 | -4.72 | -1.74 |
| 4-t-Butylbiphenyl | 218.20 | 281.82 | 281.74 | 0.08 | 0.03 |
| t-Butyl 1-phenylethylcarbamate | 220.70 | 286.52 | 284.90 | 1.62 | 0.56 |
| Undecanediamide | 221.70 | 296.20 | 286.17 | 10.03 | 3.39 |
| 4-t-Butyldiphenyl ether | 227.80 | 282.15 | 293.89 | -11.74 | -4.16 |
| Benzylferrocene | 232.50 | 286.60 | 299.84 | -13.24 | -4.62 |
| Benzoylferrocene | 234.80 | 289.15 | 302.75 | -13.60 | -4.70 |
| 3,4,4'-Trichlorocarbanilide | 236.70 | 274.29 | 305.16 | -30.87 | -11.25 |
| Dodecanediamide | 238.40 | 306.67 | 307.31 | -0.64 | -0.21 |
| 4-Nitro-4'-t-butyl-diphenylether | 251.00 | 330.70 | 323.26 | 7.44 | 2.25 |
| Tetradecanediamide | 272.90 | 357.70 | 350.98 | 6.72 | 1.88 |
| 4,4'-Di-t-butylbiphenyl | 282.80 | 384.05 | 363.51 | 20.54 | 5.35 |
| Risperidal | 367.40 | 476.00 | 470.61 | 5.39 | 1.13 |
| N-Formylmethionine-leucyl-phenylalanine methyl ether | 418.10 | 551.75 | 534.79 | 16.96 | 3.07 |
| Buckminsterfullerene | 423.30 | 487.50 | 541.37 | -53.87 | -11.05 |
| Hexatriacontane | 612.70 | 796.15 | 781.14 | 15.01 | 1.89 |
| Tetratetracontane | 746.30 | 960.70 | 950.26 | 10.44 | 1.09 |
| Pentacontane | 846.50 | 1079.95 | 1077.10 | 2.85 | 0.26 |
