# Supplementary material for: Calculation of the Isobaric Heat Capacities of the Liquid and Solid Phase of Organic Compounds at and around 298.15 K Based on Their “True” Molecular Volume
Source: Molecules. 2019 Apr 24;24(8):1626. doi: 10.3390/molecules24081626 (PMC6514989; doi:10.3390/molecules24081626)
Supplement: Supplementary file 1 [file molecules-24-01626-s001.zip › S22. Experimental and Calculated Cp(sol,325) Data Table to Figure 17C.doc]

| **Molecule name** | **Molecular volume** | **Cp(sol,325) exp** | **Cp(sol,325) calc** | **Deviation** | **Dev. in %** |
| --- | --- | --- | --- | --- | --- |
| Urea | 53.90 | 100.36 | 78.17 | 22.19 | 22.11 |
| Methylurea | 70.20 | 126.20 | 102.36 | 23.84 | 18.89 |
| Trifluoroacetamide | 73.30 | 127.50 | 106.96 | 20.54 | 16.11 |
| 1,3-Oxazolidine-2-thione | 84.90 | 126.20 | 124.17 | 2.03 | 1.61 |
| N,N-Dimethylurea | 87.60 | 141.35 | 128.17 | 13.18 | 9.32 |
| N,N'-Dimethylurea | 87.90 | 140.55 | 128.62 | 11.93 | 8.49 |
| 2-Mercapto-2-thiazoline | 94.20 | 134.30 | 137.96 | -3.66 | -2.73 |
| Tetrahydro-2H-1,3-oxazine-2-thione | 102.00 | 154.20 | 149.54 | 4.66 | 3.02 |
| 1,3,5-Trithiane | 106.70 | 143.90 | 156.51 | -12.61 | -8.76 |
| Thiobarbituric acid | 108.60 | 147.90 | 159.33 | -11.43 | -7.73 |
| 4-Chloroaniline | 109.80 | 163.23 | 161.11 | 2.12 | 1.30 |
| 1,4-Dichlorobenzene | 111.90 | 162.21 | 164.22 | -2.01 | -1.24 |
| 2-Amino-5-nitropyridine | 113.50 | 174.95 | 166.60 | 8.35 | 4.77 |
| N,N-Diethylurea | 119.80 | 196.40 | 175.95 | 20.45 | 10.41 |
| 1,4-Dibromobenzene | 120.20 | 155.19 | 176.54 | -21.35 | -13.76 |
| 1,3-Benzoxazole-2-thione | 120.30 | 164.00 | 176.69 | -12.69 | -7.74 |
| 4-Chloronitrobenzene | 120.90 | 184.20 | 177.58 | 6.62 | 3.60 |
| N,N'-Diethylurea | 121.40 | 228.90 | 178.32 | 50.58 | 22.10 |
| 2-Thiobenzoxazole | 122.70 | 164.00 | 180.25 | -16.25 | -9.91 |
| 4-Dimethylaminopyridine | 123.10 | 194.11 | 180.84 | 13.27 | 6.84 |
| 1,3,5-Trichlorobenzene | 126.10 | 170.30 | 185.29 | -14.99 | -8.80 |
| 1,3-Benzothiazole-2-thione | 129.20 | 168.10 | 189.89 | -21.79 | -12.96 |
| 1,3,5-Tribromobenzene | 138.60 | 170.40 | 203.84 | -33.44 | -19.62 |
| Acenaphthylene | 141.00 | 200.72 | 207.40 | -6.68 | -3.33 |
| 4-Methyl-4-cyclohexene-1,2-dicarboxylic anhydride | 144.70 | 260.20 | 212.89 | 47.31 | 18.18 |
| 4,4'-Bipyridine | 145.30 | 206.70 | 213.78 | -7.08 | -3.42 |
| Acenaphthene | 147.10 | 213.88 | 216.45 | -2.57 | -1.20 |
| Camphor | 154.40 | 258.80 | 227.28 | 31.52 | 12.18 |
| 1-Bromoadamantane | 163.30 | 268.60 | 240.49 | 28.11 | 10.47 |
| Barbital | 164.30 | 247.00 | 241.97 | 5.03 | 2.04 |
| Caffeine | 167.10 | 238.90 | 246.12 | -7.22 | -3.02 |
| Anthracene | 169.70 | 231.30 | 249.98 | -18.68 | -8.08 |
| 2-Phenylbenzimidazole | 171.90 | 231.72 | 253.25 | -21.53 | -9.29 |
| Monuron | 172.00 | 278.20 | 253.39 | 24.81 | 8.92 |
| 1,3-Diethyl-2-thiobarbituric acid | 172.70 | 256.10 | 254.43 | 1.67 | 0.65 |
| Tolan | 178.00 | 242.41 | 262.30 | -19.89 | -8.20 |
| Tryptophane | 182.20 | 247.06 | 268.53 | -21.47 | -8.69 |
| Pyrene | 185.50 | 249.38 | 273.42 | -24.04 | -9.64 |
| 1,3-Di-t-butyl urea | 185.80 | 296.50 | 273.87 | 22.63 | 7.63 |
| 4,4'-Dimethylbiphenyl | 186.20 | 261.15 | 274.46 | -13.31 | -5.10 |
| 2-Benzylbenzimidazole | 190.80 | 244.91 | 281.29 | -36.38 | -14.85 |
| 4'-Bromomethyl-2-cyanobiphenyl | 206.50 | 277.96 | 304.58 | -26.62 | -9.58 |
| Isoproturon | 209.40 | 330.70 | 308.88 | 21.82 | 6.60 |
| 6-Aminoflavone | 211.30 | 303.34 | 311.70 | -8.36 | -2.76 |
| Tetracene | 212.90 | 283.00 | 314.08 | -31.08 | -10.98 |
| t-Butyl 1-phenylethylcarbamate | 220.70 | 336.29 | 325.65 | 10.64 | 3.16 |
| Benzylferrocene | 232.50 | 337.10 | 343.16 | -6.06 | -1.80 |
| Benzoylferrocene | 234.80 | 341.30 | 346.57 | -5.27 | -1.54 |
| 3,4,4'-Trichlorocarbanilide | 236.70 | 294.87 | 349.39 | -54.52 | -18.49 |
| 4-Nitro-4'-t-butyl-diphenylether | 251.00 | 397.47 | 370.60 | 26.87 | 6.76 |
| Pentacene | 256.30 | 361.67 | 378.47 | -16.80 | -4.64 |
| Myclobutanil | 257.10 | 398.00 | 379.65 | 18.35 | 4.61 |
| Coronene | 259.80 | 349.91 | 383.66 | -33.75 | -9.65 |
| 4,4'-Di-t-butylbiphenyl | 282.80 | 441.60 | 417.78 | 23.82 | 5.39 |
| Dibenzo-18-crown-6 | 333.20 | 474.60 | 492.56 | -17.96 | -3.78 |
| Risperidal | 367.40 | 537.00 | 543.30 | -6.30 | -1.17 |
| Dibenzo-24-crown-8-ether | 411.90 | 668.90 | 609.33 | 59.57 | 8.91 |
| N-Formylmethionine-leucyl-phenylalanine methyl ether | 418.10 | 664.05 | 618.52 | 45.53 | 6.86 |
| Buckminsterfullerene | 423.30 | 588.00 | 626.24 | -38.24 | -6.50 |
| Dibenzo-30-crown-10 | 506.00 | 755.50 | 748.94 | 6.56 | 0.87 |
