# Supplementary material for: Calculation of the Isobaric Heat Capacities of the Liquid and Solid Phase of Organic Compounds at and around 298.15 K Based on Their “True” Molecular Volume
Source: Molecules. 2019 Apr 24;24(8):1626. doi: 10.3390/molecules24081626 (PMC6514989; doi:10.3390/molecules24081626)
Supplement: Supplementary file 1 [file molecules-24-01626-s001.zip › S23. Experimental and Calculated Cp(sol,350) Data Table to Figure 17D.doc]

| **Molecule name** | **Molecular volume** | **Cp(sol,350) exp** | **Cp(sol,350) calc** | **Deviation** | **Dev. in %** |
| --- | --- | --- | --- | --- | --- |
| Butane | 78.20 | 166.70 | 117.83 | 48.87 | 29.31 |
| 2-Mercapto-2-thiazoline | 94.20 | 146.80 | 143.10 | 3.70 | 2.52 |
| Tetrahydro-2H-1,3-oxazine-2-thione | 102.00 | 169.90 | 155.42 | 14.48 | 8.52 |
| 1,3,5-Trithiane | 106.70 | 152.02 | 162.85 | -10.83 | -7.12 |
| Thiobarbituric acid | 108.60 | 156.60 | 165.85 | -9.25 | -5.91 |
| 1,4-Dichlorobenzene | 111.90 | 181.70 | 171.06 | 10.64 | 5.86 |
| 2-Amino-5-nitropyridine | 113.50 | 189.50 | 173.59 | 15.91 | 8.40 |
| 1,4-Dibromobenzene | 120.20 | 166.20 | 184.17 | -17.97 | -10.81 |
| 1,3-Benzoxazole-2-thione | 120.30 | 175.20 | 184.33 | -9.13 | -5.21 |
| 2-Thiobenzoxazole | 122.70 | 175.20 | 188.12 | -12.92 | -7.37 |
| 4-Dimethylaminopyridine | 123.10 | 207.69 | 188.75 | 18.94 | 9.12 |
| 1,3,5-Trichlorobenzene | 126.10 | 200.99 | 193.49 | 7.50 | 3.73 |
| 1,3-Benzothiazole-2-thione | 129.20 | 179.50 | 198.38 | -18.88 | -10.52 |
| 1,3,5-Tribromobenzene | 138.60 | 178.96 | 213.23 | -34.27 | -19.15 |
| Acenaphthene | 147.10 | 239.24 | 226.65 | 12.59 | 5.26 |
| Camphor | 154.40 | 272.00 | 238.18 | 33.82 | 12.43 |
| 1-Bromoadamantane | 163.30 | 302.15 | 252.24 | 49.91 | 16.52 |
| Barbital | 164.30 | 262.70 | 253.82 | 8.88 | 3.38 |
| Caffeine | 167.10 | 254.40 | 258.24 | -3.84 | -1.51 |
| Anthracene | 169.70 | 252.25 | 262.35 | -10.10 | -4.00 |
| 2-Phenylbenzimidazole | 171.90 | 249.26 | 265.82 | -16.56 | -6.64 |
| Monuron | 172.00 | 302.40 | 265.98 | 36.42 | 12.04 |
| 1,3-Diethyl-2-thiobarbituric acid | 172.70 | 273.30 | 267.09 | 6.21 | 2.27 |
| Tryptophane | 182.20 | 265.98 | 282.09 | -16.11 | -6.06 |
| Pyrene | 185.50 | 276.20 | 287.30 | -11.10 | -4.02 |
| 4,4'-Dimethylbiphenyl | 186.20 | 281.00 | 288.41 | -7.41 | -2.64 |
| 2-Benzylbenzimidazole | 190.80 | 261.16 | 295.67 | -34.51 | -13.21 |
| 4'-Bromomethyl-2-cyanobiphenyl | 206.50 | 298.70 | 320.47 | -21.77 | -7.29 |
| 6-Aminoflavone | 211.30 | 326.13 | 328.05 | -1.92 | -0.59 |
| Tetracene | 212.90 | 304.10 | 330.58 | -26.48 | -8.71 |
| Benzylferrocene | 232.50 | 358.60 | 361.53 | -2.93 | -0.82 |
| Benzoylferrocene | 234.80 | 371.70 | 365.16 | 6.54 | 1.76 |
| 3,4,4'-Trichlorocarbanilide | 236.70 | 307.35 | 368.16 | -60.81 | -19.79 |
| 4-Nitro-4'-t-butyl-diphenylether | 251.00 | 454.38 | 390.75 | 63.63 | 14.00 |
| Pentacene | 256.30 | 366.00 | 399.12 | -33.12 | -9.05 |
| Coronene | 259.80 | 373.94 | 404.65 | -30.71 | -8.21 |
| Fenoxycarb | 276.60 | 434.02 | 431.18 | 2.83 | 0.65 |
| 4,4'-Di-t-butylbiphenyl | 282.80 | 474.60 | 440.97 | 33.63 | 7.09 |
| Dibenzo-18-crown-6 | 333.20 | 506.20 | 520.57 | -14.37 | -2.84 |
| Risperidal | 367.40 | 571.00 | 574.59 | -3.59 | -0.63 |
| Dibenzo-24-crown-8-ether | 411.90 | 675.00 | 644.87 | 30.13 | 4.46 |
| Buckminsterfullerene | 423.30 | 633.90 | 662.87 | -28.97 | -4.57 |
| Dibenzo-30-crown-10 | 506.00 | 826.40 | 793.49 | 32.91 | 3.98 |
