# Supplementary material for: Calculation of the Isobaric Heat Capacities of the Liquid and Solid Phase of Organic Compounds at and around 298.15 K Based on Their “True” Molecular Volume
Source: Molecules. 2019 Apr 24;24(8):1626. doi: 10.3390/molecules24081626 (PMC6514989; doi:10.3390/molecules24081626)
Supplement: Supplementary file 1 [file molecules-24-01626-s001.zip › S24. Experimental and Calculated Cp(sol,250) Data Table to Figure 18A.doc]

| **Molecule name** | **Molecular volume** | **Cp(sol,250) exp** | **Cp(sol,250) calc** | **Deviation** | **Dev. in %** |
| --- | --- | --- | --- | --- | --- |
| 2-Pyrazinecarboxylic acid | 102.50 | 125.60 | 118.29 | 7.31 | 5.82 |
| Nicotinic acid | 107.00 | 125.80 | 124.04 | 1.76 | 1.40 |
| 2-Pyridinealdoxime | 108.00 | 128.78 | 125.31 | 3.47 | 2.69 |
| Erythritol | 113.40 | 140.29 | 132.21 | 8.08 | 5.76 |
| cis-1,2-Cyclohexanediol | 118.30 | 128.68 | 138.48 | -9.80 | -7.61 |
| trans-1,2-Cyclohexanediol | 118.40 | 139.48 | 138.60 | 0.88 | 0.63 |
| D-Ribose | 128.50 | 154.92 | 151.51 | 3.41 | 2.20 |
| Xylitol | 130.00 | 174.10 | 153.43 | 20.67 | 11.88 |
| 1,6-Anhydro-beta-D-glucopyranose | 130.70 | 155.50 | 154.32 | 1.18 | 0.76 |
| 8-Quinolinol | 130.90 | 146.35 | 154.58 | -8.23 | -5.62 |
| 2,6-Pyridinedicarboxylic acid | 134.60 | 128.84 | 159.30 | -30.46 | -23.64 |
| Arabitol | 138.90 | 170.71 | 164.80 | 5.91 | 3.46 |
| Inositol | 149.90 | 181.94 | 178.85 | 3.09 | 1.70 |
| beta-D-Fructose | 150.90 | 193.28 | 180.13 | 13.15 | 6.80 |
| D-Psicose | 151.60 | 186.65 | 181.02 | 5.63 | 3.01 |
| D-Tagatose | 152.40 | 190.03 | 182.05 | 7.98 | 4.20 |
| 2-Adamantanol | 153.60 | 168.12 | 183.58 | -15.46 | -9.20 |
| alpha-D-Glucose | 153.60 | 184.33 | 183.58 | 0.75 | 0.41 |
| D-Mannose | 153.80 | 187.32 | 183.84 | 3.48 | 1.86 |
| 1-Adamantanol | 154.00 | 157.10 | 184.09 | -26.99 | -17.18 |
| D(+)-Carnitine | 155.40 | 191.60 | 185.88 | 5.72 | 2.99 |
| Sorbitol | 163.50 | 195.26 | 196.23 | -0.97 | -0.50 |
| Dopa | 173.30 | 217.60 | 208.75 | 8.85 | 4.07 |
| Menthol | 174.80 | 206.50 | 210.67 | -4.17 | -2.02 |
| N-Methylephedrine | 184.70 | 260.66 | 223.32 | 37.34 | 14.33 |
| n-Decyl alcohol | 187.00 | 247.17 | 226.26 | 20.91 | 8.46 |
| Ibuprofen | 208.20 | 265.10 | 253.35 | 11.75 | 4.43 |
| Arabinosylhypoxanthine | 208.90 | 239.55 | 254.24 | -14.69 | -6.13 |
| N-Methylephedrine hydrochloride | 210.80 | 237.12 | 256.67 | -19.55 | -8.24 |
| Vidarabine | 218.70 | 244.15 | 266.76 | -22.61 | -9.26 |
| 3,5-Di-t-butylsalicylic acid | 247.00 | 332.70 | 302.92 | 29.78 | 8.95 |
| Tetradecanoic acid | 256.60 | 328.06 | 315.19 | 12.87 | 3.92 |
| Saccharose | 281.50 | 356.88 | 347.00 | 9.88 | 2.77 |
| Palmitic acid | 290.00 | 362.65 | 357.87 | 4.78 | 1.32 |
| Vitamin B2 | 321.30 | 375.10 | 397.86 | -22.76 | -6.07 |
| Stearic acid | 323.40 | 405.48 | 400.54 | 4.94 | 1.22 |
| Methylprednisolone | 338.50 | 396.30 | 419.84 | -23.54 | -5.94 |
| Arachidic acid | 356.80 | 435.65 | 443.22 | -7.57 | -1.74 |
| N-Formylmethionine-leucyl-phenylalanine | 403.70 | 478.70 | 503.15 | -24.45 | -5.11 |
| Pyrimethanil laurate | 411.70 | 504.18 | 513.37 | -9.19 | -1.82 |
| Methylprednisolone aceponate | 426.90 | 513.60 | 532.79 | -19.19 | -3.74 |
| Tetraphenyltetrahydroxycyclotetrasiloxane | 432.00 | 537.30 | 539.31 | -2.01 | -0.37 |
| Octaphenyltetrahydroxytricyclooctasiloxane | 816.00 | 1058.00 | 1029.96 | 28.04 | 2.65 |
