# Supplementary material for: Calculation of the Isobaric Heat Capacities of the Liquid and Solid Phase of Organic Compounds at and around 298.15 K Based on Their “True” Molecular Volume
Source: Molecules. 2019 Apr 24;24(8):1626. doi: 10.3390/molecules24081626 (PMC6514989; doi:10.3390/molecules24081626)
Supplement: Supplementary file 1 [file molecules-24-01626-s001.zip › S25. Experimental and Calculated Cp(sol,275) Data Table to Figure 18B.doc]

| **Molecule name** | **Molecular volume** | **Cp(sol,275) exp** | **Cp(sol,275) calc** | **Deviation** | **Dev. in %** |
| --- | --- | --- | --- | --- | --- |
| 2-Pyrazinecarboxylic acid | 102.50 | 134.30 | 136.04 | -1.74 | -1.30 |
| Nicotinic acid | 107.00 | 138.00 | 142.07 | -4.07 | -2.95 |
| 2-Pyridinealdoxime | 108.00 | 140.76 | 143.40 | -2.64 | -1.88 |
| 4-Hydroxybenzaldehyde | 111.50 | 139.30 | 148.09 | -8.79 | -6.31 |
| Erythritol | 113.40 | 152.00 | 150.63 | 1.37 | 0.90 |
| cis-1,2-Cyclohexanediol | 118.30 | 144.36 | 157.19 | -12.83 | -8.89 |
| trans-1,2-Cyclohexanediol | 118.40 | 146.88 | 157.33 | -10.45 | -7.11 |
| D-Ribose | 128.50 | 170.73 | 170.85 | -0.12 | -0.07 |
| Xylitol | 130.00 | 189.56 | 172.86 | 16.70 | 8.81 |
| 1,6-Anhydro-beta-D-glucopyranose | 130.70 | 171.80 | 173.79 | -1.99 | -1.16 |
| 8-Quinolinol | 130.90 | 163.83 | 174.06 | -10.23 | -6.24 |
| 2,6-Pyridinedicarboxylic acid | 134.60 | 147.12 | 179.01 | -31.89 | -21.68 |
| 4-Hydroxy-3-methoxybenzaldehyde | 137.10 | 176.30 | 182.36 | -6.06 | -3.44 |
| Arabitol | 138.90 | 185.17 | 184.77 | 0.40 | 0.22 |
| Inositol | 149.90 | 199.08 | 199.50 | -0.42 | -0.21 |
| beta-D-Fructose | 150.90 | 212.00 | 200.83 | 11.17 | 5.27 |
| D-Psicose | 151.60 | 204.17 | 201.77 | 2.40 | 1.17 |
| D-Tagatose | 152.40 | 210.00 | 202.84 | 7.16 | 3.41 |
| alpha-D-Glucose | 153.60 | 202.20 | 204.45 | -2.25 | -1.11 |
| D-Mannose | 153.80 | 205.67 | 204.72 | 0.95 | 0.46 |
| Ethyl vanillin | 153.90 | 198.50 | 204.85 | -6.35 | -3.20 |
| D(+)-Carnitine | 155.40 | 208.50 | 206.86 | 1.64 | 0.79 |
| Isoborneol | 163.20 | 239.94 | 217.30 | 22.64 | 9.44 |
| Sorbitol | 163.50 | 212.01 | 217.70 | -5.69 | -2.68 |
| Dopa | 173.30 | 239.80 | 230.82 | 8.98 | 3.74 |
| Menthol | 174.80 | 228.20 | 232.83 | -4.63 | -2.03 |
| N-Methylephedrine | 184.70 | 280.42 | 246.08 | 34.34 | 12.25 |
| Ibuprofen | 208.20 | 291.70 | 277.54 | 14.16 | 4.85 |
| Arabinosylhypoxanthine | 208.90 | 261.97 | 278.48 | -16.51 | -6.30 |
| N-Methylephedrine hydrochloride | 210.80 | 259.80 | 281.02 | -21.22 | -8.17 |
| Vidarabine | 218.70 | 267.50 | 291.60 | -24.10 | -9.01 |
| 3,5-Di-t-butylsalicylic acid | 247.00 | 361.50 | 329.48 | 32.02 | 8.86 |
| Tetradecanoic acid | 256.60 | 371.47 | 342.33 | 29.14 | 7.84 |
| Saccharose | 281.50 | 392.52 | 375.67 | 16.85 | 4.29 |
| Palmitic acid | 290.00 | 408.03 | 387.05 | 20.98 | 5.14 |
| Vitamin B2 | 321.30 | 408.00 | 428.95 | -20.95 | -5.13 |
| Stearic acid | 323.40 | 447.27 | 431.76 | 15.51 | 3.47 |
| Methylprednisolone | 338.50 | 435.00 | 451.97 | -16.97 | -3.90 |
| Arachidic acid | 356.80 | 487.33 | 476.47 | 10.86 | 2.23 |
| N-Formylmethionine-leucyl-phenylalanine | 403.70 | 521.35 | 539.26 | -17.91 | -3.43 |
| Pyrimethanil laurate | 411.70 | 541.43 | 549.97 | -8.54 | -1.58 |
| Methylprednisolone aceponate | 426.90 | 559.40 | 570.31 | -10.91 | -1.95 |
