# Supplementary material for: Calculation of the Isobaric Heat Capacities of the Liquid and Solid Phase of Organic Compounds at and around 298.15 K Based on Their “True” Molecular Volume
Source: Molecules. 2019 Apr 24;24(8):1626. doi: 10.3390/molecules24081626 (PMC6514989; doi:10.3390/molecules24081626)
Supplement: Supplementary file 1 [file molecules-24-01626-s001.zip › S26. Experimental and Calculated Cp(sol,325) Data Table to Figure 18C.doc]

| **Molecule name** | **Molecular volume** | **Cp(sol,325) exp** | **Cp(sol,325) calc** | **Deviation** | **Dev. in %** |
| --- | --- | --- | --- | --- | --- |
| 2-Pyrazinecarboxylic acid | 102.50 | 153.10 | 155.34 | -2.24 | -1.46 |
| Nicotinic acid | 107.00 | 165.80 | 162.68 | 3.12 | 1.88 |
| 2-Pyridinealdoxime | 108.00 | 164.54 | 164.31 | 0.23 | 0.14 |
| Benzoic acid | 111.10 | 156.29 | 169.36 | -13.07 | -8.36 |
| 4-Hydroxybenzaldehyde | 111.50 | 162.80 | 170.02 | -7.22 | -4.43 |
| Erythritol | 113.40 | 181.48 | 173.11 | 8.37 | 4.61 |
| cis-1,2-Cyclohexanediol | 118.30 | 175.88 | 181.10 | -5.22 | -2.97 |
| trans-1,2-Cyclohexanediol | 118.40 | 174.68 | 181.27 | -6.59 | -3.77 |
| D-Ribose | 128.50 | 214.87 | 197.73 | 17.14 | 7.97 |
| Xylose | 128.50 | 190.99 | 197.73 | -6.74 | -3.53 |
| Xylitol | 130.00 | 231.90 | 200.18 | 31.72 | 13.68 |
| 1,6-Anhydro-beta-D-glucopyranose | 130.70 | 206.50 | 201.32 | 5.18 | 2.51 |
| 8-Quinolinol | 130.90 | 198.30 | 201.65 | -3.35 | -1.69 |
| 2,6-Pyridinedicarboxylic acid | 134.60 | 187.75 | 207.68 | -19.93 | -10.62 |
| 4-Hydroxy-3-methoxybenzaldehyde | 137.10 | 202.10 | 211.76 | -9.66 | -4.78 |
| Arabitol | 138.90 | 224.62 | 214.69 | 9.93 | 4.42 |
| 2-Deoxy-d-glucose | 143.70 | 214.00 | 222.52 | -8.52 | -3.98 |
| 2,5-Dibromobenzoic acid | 147.00 | 242.23 | 227.90 | 14.33 | 5.92 |
| 3,5-Dibromobenzoic acid | 148.30 | 242.21 | 230.02 | 12.19 | 5.03 |
| Inositol | 149.90 | 241.80 | 232.63 | 9.17 | 3.79 |
| beta-D-Fructose | 150.90 | 251.60 | 234.26 | 17.34 | 6.89 |
| D-Psicose | 151.60 | 240.70 | 235.40 | 5.30 | 2.20 |
| D-Tagatose | 152.40 | 250.00 | 236.70 | 13.30 | 5.32 |
| 2-Adamantanol | 153.60 | 228.65 | 238.66 | -10.01 | -4.38 |
| alpha-D-Glucose | 153.60 | 241.09 | 238.66 | 2.43 | 1.01 |
| D-Mannose | 153.80 | 235.24 | 238.99 | -3.75 | -1.59 |
| D-Galactose | 153.80 | 236.04 | 238.99 | -2.95 | -1.25 |
| Ethyl vanillin | 153.90 | 231.70 | 239.15 | -7.45 | -3.22 |
| 1-Adamantanol | 154.00 | 219.09 | 239.31 | -20.22 | -9.23 |
| D(+)-Carnitine | 155.40 | 243.50 | 241.60 | 1.90 | 0.78 |
| Isoborneol | 163.20 | 294.30 | 254.31 | 39.99 | 13.59 |
| Sorbitol | 163.50 | 254.58 | 254.80 | -0.22 | -0.09 |
| 1-Methoxy-a-D-glucopyranoside | 167.30 | 268.21 | 261.00 | 7.21 | 2.69 |
| Dopa | 173.30 | 285.40 | 270.78 | 14.62 | 5.12 |
| N-Methylephedrine | 184.70 | 321.32 | 289.37 | 31.95 | 9.94 |
| N-Acetyl-D-glucosamine | 189.30 | 292.85 | 296.87 | -4.02 | -1.37 |
| 5-Hydroxyflavone | 206.70 | 308.55 | 325.24 | -16.69 | -5.41 |
| 3-Hydroxyflavone | 207.00 | 285.46 | 325.73 | -40.27 | -14.11 |
| Ibuprofen | 208.20 | 366.38 | 327.69 | 38.69 | 10.56 |
| 7-Hydroxyflavone | 208.80 | 278.76 | 328.67 | -49.91 | -17.90 |
| 6-Hydroxyflavone | 208.80 | 279.07 | 328.67 | -49.60 | -17.77 |
| Arabinosylhypoxanthine | 208.90 | 308.16 | 328.83 | -20.67 | -6.71 |
| N-Methylephedrine hydrochloride | 210.80 | 309.50 | 331.93 | -22.43 | -7.25 |
| Vidarabine | 218.70 | 316.55 | 344.81 | -28.26 | -8.93 |
| 3,5-Di-t-butylsalicylic acid | 247.00 | 416.30 | 390.95 | 25.35 | 6.09 |
| Cellobiose | 277.50 | 463.30 | 440.68 | 22.62 | 4.88 |
| Maltose | 277.90 | 488.81 | 441.34 | 47.47 | 9.71 |
| Saccharose | 281.50 | 462.15 | 447.21 | 14.94 | 3.23 |
| Lactose | 283.60 | 461.54 | 450.63 | 10.91 | 2.36 |
| Methylprednisolone | 338.50 | 514.75 | 540.15 | -25.40 | -4.93 |
| N-Formylmethionine-leucyl-phenylalanine | 403.70 | 618.80 | 646.46 | -27.66 | -4.47 |
| Maltotriose | 406.70 | 701.60 | 651.35 | 50.25 | 7.16 |
| Simvastatin | 408.40 | 623.85 | 654.12 | -30.27 | -4.85 |
| Methylprednisolone aceponate | 426.90 | 657.05 | 684.29 | -27.24 | -4.15 |
| Cyclodextrin | 776.30 | 1267.92 | 1254.00 | 13.92 | 1.10 |
