# Supplementary material for: Calculation of the Isobaric Heat Capacities of the Liquid and Solid Phase of Organic Compounds at and around 298.15 K Based on Their “True” Molecular Volume
Source: Molecules. 2019 Apr 24;24(8):1626. doi: 10.3390/molecules24081626 (PMC6514989; doi:10.3390/molecules24081626)
Supplement: Supplementary file 1 [file molecules-24-01626-s001.zip › S27. Experimental and Calculated Cp(sol,350) Data Table to Figure 18D.doc]

| **Molecule name** | **Molecular volume** | **Cp(sol,350) exp** | **Cp(sol,350) calc** | **Deviation** | **Dev. in %** |
| --- | --- | --- | --- | --- | --- |
| 2-Pyrazinecarboxylic acid | 102.50 | 161.80 | 162.21 | -0.41 | -0.25 |
| 2-Pyridinealdoxime | 108.00 | 175.43 | 172.33 | 3.10 | 1.77 |
| Benzoic acid | 111.10 | 167.52 | 178.03 | -10.51 | -6.28 |
| 4-Hydroxybenzaldehyde | 111.50 | 174.30 | 178.77 | -4.47 | -2.56 |
| Erythritol | 113.40 | 195.43 | 182.27 | 13.16 | 6.74 |
| cis-1,2-Cyclohexanediol | 118.30 | 192.50 | 191.28 | 1.22 | 0.63 |
| trans-1,2-Cyclohexanediol | 118.40 | 186.14 | 191.47 | -5.33 | -2.86 |
| Xylose | 128.50 | 206.37 | 210.05 | -3.68 | -1.79 |
| Xylitol | 130.00 | 257.90 | 212.81 | 45.09 | 17.48 |
| 1,6-Anhydro-beta-D-glucopyranose | 130.70 | 227.10 | 214.10 | 13.00 | 5.72 |
| 2,6-Pyridinedicarboxylic acid | 134.60 | 208.64 | 221.28 | -12.64 | -6.06 |
| 2-Deoxy-d-glucose | 143.70 | 233.88 | 238.03 | -4.15 | -1.77 |
| Inositol | 149.90 | 263.15 | 249.43 | 13.72 | 5.21 |
| beta-D-Fructose | 150.90 | 269.69 | 251.27 | 18.42 | 6.83 |
| 2-Adamantanol | 153.60 | 290.18 | 256.24 | 33.94 | 11.70 |
| alpha-D-Glucose | 153.60 | 261.14 | 256.24 | 4.90 | 1.88 |
| D-Mannose | 153.80 | 253.03 | 256.61 | -3.58 | -1.42 |
| D-Galactose | 153.80 | 255.74 | 256.61 | -0.87 | -0.34 |
| Sorbitol | 163.50 | 275.71 | 274.46 | 1.25 | 0.45 |
| 1-Methoxy-a-D-glucopyranoside | 167.30 | 314.27 | 281.45 | 32.82 | 10.44 |
| Dopa | 173.30 | 309.20 | 292.50 | 16.70 | 5.40 |
| N-Acetyl-D-glucosamine | 189.30 | 321.74 | 321.94 | -0.20 | -0.06 |
| 5-Hydroxyflavone | 206.70 | 325.65 | 353.96 | -28.31 | -8.69 |
| 3-Hydroxyflavone | 207.00 | 304.53 | 354.51 | -49.98 | -16.41 |
| 7-Hydroxyflavone | 208.80 | 300.95 | 357.82 | -56.87 | -18.90 |
| 6-Hydroxyflavone | 208.80 | 304.14 | 357.82 | -53.68 | -17.65 |
| N-Methylephedrine hydrochloride | 210.80 | 336.70 | 361.50 | -24.80 | -7.37 |
| 3,5-Di-t-butylsalicylic acid | 247.00 | 446.20 | 428.12 | 18.08 | 4.05 |
| Cellobiose | 277.50 | 507.86 | 484.24 | 23.62 | 4.65 |
| Maltose | 277.90 | 548.23 | 484.98 | 63.25 | 11.54 |
| Saccharose | 281.50 | 468.57 | 491.60 | -23.03 | -4.92 |
| Lactose | 283.60 | 501.07 | 495.47 | 5.60 | 1.12 |
| Methylprednisolone | 338.50 | 556.80 | 596.50 | -39.70 | -7.13 |
| Maltotriose | 406.70 | 778.42 | 722.00 | 56.42 | 7.25 |
| Simvastatin | 408.40 | 668.44 | 725.13 | -56.69 | -8.48 |
| Cyclodextrin | 776.30 | 1416.77 | 1402.14 | 14.63 | 1.03 |
