# Supplementary material for: Calculation of the Isobaric Heat Capacities of the Liquid and Solid Phase of Organic Compounds at and around 298.15 K Based on Their “True” Molecular Volume
Source: Molecules. 2019 Apr 24;24(8):1626. doi: 10.3390/molecules24081626 (PMC6514989; doi:10.3390/molecules24081626)
Supplement: Supplementary file 1 [file molecules-24-01626-s001.zip › Table 1.doc]

| **Molecule** | **Vm (present work)** | **Vm (ref. 41)** | **Vm (ref. 35)** |
| --- | --- | --- | --- |
| Carbon dioxide | 34.1 | 30.61 |  |
| Water | 19.2 | 16.82 |  |
| Ammonia | 22.7 | 21.91 |  |
| Methane | 28.1 | 28.01 | 28.42 |
| Ethane | 44.9 | 44.63 | 45.38 |
| Propane | 61.5 | 61.39 | 62.37 |
| Ethylene | 39.6 | 40.25 | 39.64 |
| Acetylene | 34.8 | 36.15 | 38.35 |
| Butadiyne | 58.3 | 59.74 | 64.31 |
| Benzene | 83.4 | 85.39 | 80.28 |
| Toluene | 100 | 101.8 | 98.79 |
| Biphenyl | 153.3 | 157.1 | 152.2 |
| Fluoroethane | 49.8 | 47.45 | 49.17 |
| Chloroethane | 59.1 | 59.45 | 58.96 |
| Bromobenzene | 101.8 | 106.6 | 100.3 |
| Iodobenzene | 108 | 114.7 | 108.7 |
| Cyclopropane | 54.6 | 42.7 / 50 |  |
| Cyclohexane | 100.5 | 99.1 | 100 |
| Acetone | 64.6 | 62.86 | 64.81 |
| Methanol | 36.9 | 34.89 | 36.04 |
| Acetic acid | 56.7 | 51.18 | 55.1 |
