# Supplementary material for: Calculation of the Isobaric Heat Capacities of the Liquid and Solid Phase of Organic Compounds at and around 298.15 K Based on Their “True” Molecular Volume
Source: Molecules. 2019 Apr 24;24(8):1626. doi: 10.3390/molecules24081626 (PMC6514989; doi:10.3390/molecules24081626)
Supplement: Supplementary file 1 [file molecules-24-01626-s001.zip › Table 10.doc]

| **Molecule name** | **Vm** | **Cp(sol,298) exp** | **Cp(sol,298) calc** | **Deviation** | **Dev. in %** |
| --- | --- | --- | --- | --- | --- |
| Oxalic acid | 67.90 | 91.00 | 90.02 | 0.98 | 1.07 |
| Squaric acid | 80.00 | 121.80 | 108.73 | 13.07 | 10.73 |
| Malonic acid | 81.70 | 127.63 | 111.36 | 16.27 | 12.75 |
| Lactic acid | 81.80 | 127.60 | 111.52 | 16.08 | 12.60 |
| Maleic acid | 94.10 | 135.60 | 130.54 | 5.06 | 3.73 |
| Fumaric acid | 96.00 | 141.80 | 133.47 | 8.33 | 5.87 |
| Butanedioic acid | 100.90 | 152.93 | 141.05 | 11.88 | 7.77 |
| 1,3-Dihydroxybenzene | 101.00 | 135.53 | 141.20 | -5.67 | -4.19 |
| 1,4-Dihydroxybenzene | 101.00 | 136.40 | 141.20 | -4.80 | -3.52 |
| 1,2-Dihydroxybenzene | 101.10 | 144.30 | 141.36 | 2.94 | 2.04 |
| Diethanolamine | 108.30 | 137.00 | 152.49 | -15.49 | -11.31 |
| 2,2-Dimethyl-1,3-propanediol | 112.30 | 183.18 | 158.68 | 24.50 | 13.38 |
| Erythritol | 113.40 | 161.90 | 160.38 | 1.52 | 0.94 |
| Tartaric acid | 114.40 | 184.50 | 161.92 | 22.58 | 12.24 |
| TRIS | 116.40 | 171.27 | 165.02 | 6.25 | 3.65 |
| 2,5-Dihydroxytoluene | 117.30 | 174.90 | 166.41 | 8.49 | 4.86 |
| 2-Methylbutanedioic acid | 117.50 | 199.60 | 166.72 | 32.88 | 16.47 |
| Pentanedioic acid | 117.60 | 186.90 | 166.87 | 20.03 | 10.72 |
| cis-1,2-Cyclohexanediol | 118.30 | 160.40 | 167.95 | -7.55 | -4.71 |
| trans-1,2-Cyclohexanediol | 118.40 | 163.20 | 168.11 | -4.91 | -3.01 |
| Salicylic acid | 118.50 | 160.90 | 168.26 | -7.36 | -4.58 |
| 4-Carboxyphenol | 120.10 | 155.20 | 170.74 | -15.54 | -10.01 |
| 3,4-Dihydroxybenzaldehyde | 120.30 | 205.90 | 171.05 | 34.85 | 16.93 |
| 3-Carboxyphenol | 120.40 | 157.30 | 171.20 | -13.90 | -8.84 |
| Pentaerythritol | 127.60 | 188.40 | 182.33 | 6.07 | 3.22 |
| Xylose | 128.50 | 180.40 | 183.73 | -3.33 | -1.84 |
| D-Ribose | 128.50 | 183.20 | 183.73 | -0.53 | -0.29 |
| Arabinose | 128.90 | 184.10 | 184.34 | -0.24 | -0.13 |
| Hexamethyleneglycol | 129.50 | 190.00 | 185.27 | 4.73 | 2.49 |
| Xylitol | 130.00 | 207.00 | 186.05 | 20.95 | 10.12 |
| Thiophenedicarboxylic acid | 130.10 | 204.87 | 186.20 | 18.67 | 9.11 |
| 1,6-Anhydro-beta-D-glucopyranose | 130.70 | 187.20 | 187.13 | 0.07 | 0.04 |
| 1,6-Hexanedioic acid | 134.30 | 196.60 | 192.69 | 3.91 | 1.99 |
| 2,6-Pyridinedicarboxylic acid | 134.60 | 166.31 | 193.16 | -26.85 | -16.14 |
| Mandelic acid | 136.00 | 199.20 | 195.32 | 3.88 | 1.95 |
| Ethriol | 137.10 | 213.80 | 197.02 | 16.78 | 7.85 |
| Gallic acid | 138.00 | 199.55 | 198.42 | 1.13 | 0.57 |
| Phthalic acid | 138.20 | 188.11 | 198.72 | -10.61 | -5.64 |
| Isophthalic acid | 138.30 | 201.70 | 198.88 | 2.82 | 1.40 |
| Arabitol | 138.90 | 201.35 | 199.81 | 1.54 | 0.77 |
| Terephthalic acid | 139.50 | 199.60 | 200.74 | -1.14 | -0.57 |
| 2-Deoxy-d-glucose | 143.70 | 200.20 | 207.23 | -7.03 | -3.51 |
| 4,5-Dihydroxy-2-(dinitromethylene)imidazolidine | 147.10 | 217.76 | 212.49 | 5.27 | 2.42 |
| trans-4-Coumaric acid | 147.60 | 193.00 | 213.26 | -20.26 | -10.50 |
| Trimethylhydroquinone | 148.30 | 217.60 | 214.34 | 3.26 | 1.50 |
| Inositol | 149.90 | 218.00 | 216.82 | 1.18 | 0.54 |
| 3-Chloromandelic acid | 150.10 | 211.10 | 217.13 | -6.03 | -2.85 |
| beta-D-Fructose | 150.90 | 227.70 | 218.36 | 9.34 | 4.10 |
| Heptanedioic acid | 151.00 | 240.70 | 218.52 | 22.18 | 9.22 |
| Vitamin B6 | 151.20 | 244.46 | 218.83 | 25.63 | 10.49 |
| Citric acid | 151.60 | 226.40 | 219.44 | 6.96 | 3.07 |
| D-Psicose | 151.60 | 221.30 | 219.44 | 1.86 | 0.84 |
| D-Tagatose | 152.40 | 228.00 | 220.68 | 7.32 | 3.21 |
| alpha-D-Glucose | 153.60 | 219.19 | 222.54 | -3.35 | -1.53 |
| D-Galactose | 153.80 | 213.70 | 222.85 | -9.15 | -4.28 |
| D-Mannose | 153.80 | 215.90 | 222.85 | -6.95 | -3.22 |
| 2,4-Dihydroxycinnamic acid | 155.80 | 178.36 | 225.94 | -47.58 | -26.68 |
| Caffeic acid | 156.40 | 181.96 | 226.87 | -44.91 | -24.68 |
| Dulcose | 158.80 | 238.50 | 230.58 | 7.92 | 3.32 |
| 3-Nitrophthalic acid | 159.80 | 247.02 | 232.12 | 14.90 | 6.03 |
| 4-Nitrophthalic acid | 160.60 | 232.24 | 233.36 | -1.12 | -0.48 |
| 3,4-Dihydroxyhydrocinnamic acid | 161.30 | 218.62 | 234.44 | -15.82 | -7.24 |
| 1,8-Octanediol | 162.90 | 236.36 | 236.92 | -0.56 | -0.24 |
| Sorbitol | 163.50 | 239.00 | 237.84 | 1.16 | 0.48 |
| Octanedioic acid | 167.20 | 267.60 | 243.57 | 24.03 | 8.98 |
| 1-Methoxy-a-D-glucopyranoside | 167.30 | 233.70 | 243.72 | -10.02 | -4.29 |
| 4,4'-Dihydroxybiphenyl | 171.40 | 224.31 | 250.06 | -25.75 | -11.48 |
| Ferulic acid | 173.20 | 235.94 | 252.84 | -16.90 | -7.16 |
| Dopa | 173.30 | 260.60 | 253.00 | 7.60 | 2.92 |
| 1,4-Bis-(2-hydroxyethyl)piperazine | 175.10 | 250.68 | 255.78 | -5.10 | -2.03 |
| Glyceryl-2-benzoate | 179.30 | 236.40 | 262.28 | -25.88 | -10.95 |
| 1,9-Nonanediol | 179.60 | 256.74 | 262.74 | -6.00 | -2.34 |
| 1-Benzoylglycerol | 179.80 | 238.90 | 263.05 | -24.15 | -10.11 |
| 2,6-Naphthalenedicarboxylic acid | 182.80 | 230.30 | 267.69 | -37.39 | -16.23 |
| Nonanedioic acid | 184.40 | 294.50 | 270.16 | 24.34 | 8.26 |
| Bisphenol F | 188.50 | 246.30 | 276.50 | -30.20 | -12.26 |
| N-Acetyl-D-glucosamine | 189.30 | 276.90 | 277.74 | -0.84 | -0.30 |
| 1,10-Decanediol | 196.30 | 279.26 | 288.56 | -9.30 | -3.33 |
| Decanedioic acid | 201.10 | 321.40 | 295.98 | 25.42 | 7.91 |
| Bisphenol E | 204.60 | 276.41 | 301.40 | -24.99 | -9.04 |
| Bisphenol S | 206.20 | 280.30 | 303.87 | -23.57 | -8.41 |
| Arabinosylhypoxanthine | 208.90 | 283.30 | 308.04 | -24.74 | -8.73 |
| 3,3'-Dihydroxy-4,4'-diaminodiphenylmethane | 210.90 | 281.20 | 311.14 | -29.94 | -10.65 |
| 1,11-Undecanediol | 213.00 | 297.79 | 314.38 | -16.59 | -5.57 |
| Undecanedioic acid | 217.80 | 348.30 | 321.81 | 26.49 | 7.61 |
| Vidarabine | 218.70 | 290.10 | 323.20 | -33.10 | -11.41 |
| Bisphenol A | 219.00 | 301.34 | 323.66 | -22.32 | -7.41 |
| 1-(2,2,3-Trimethyl-1,3-oxazolidin-5-yl)-butane-1,2,3,4-tetrol | 219.90 | 331.46 | 325.05 | 6.41 | 1.93 |
| Ellagic acid | 226.60 | 328.85 | 335.41 | -6.56 | -2.00 |
| 1,12-Dodecanediol | 229.70 | 330.23 | 340.21 | -9.98 | -3.02 |
| Trolox | 231.40 | 332.00 | 342.83 | -10.83 | -3.26 |
| Dodecanedioic acid | 234.50 | 375.20 | 347.63 | 27.57 | 7.35 |
| 1,13-Tridecanediol | 246.40 | 366.88 | 366.03 | 0.85 | 0.23 |
| 3,5-Di-t-butylsalicylic acid | 247.00 | 383.00 | 366.96 | 16.04 | 4.19 |
| 1,13-Tridecanedioic acid | 251.20 | 402.10 | 373.45 | 28.65 | 7.13 |
| 1-Monocaprin | 258.20 | 410.00 | 384.27 | 25.73 | 6.27 |
| 1,14-Tetradecanediol | 263.10 | 379.61 | 391.85 | -12.24 | -3.22 |
| 1,14-Tetradecanedioic acid | 267.90 | 429.00 | 399.27 | 29.73 | 6.93 |
| Bisphenol AP | 274.00 | 365.85 | 408.70 | -42.85 | -11.71 |
| Cellobiose | 277.50 | 436.10 | 414.12 | 21.98 | 5.04 |
| Maltose | 277.90 | 434.70 | 414.73 | 19.97 | 4.59 |
| 1,15-Pentadecanediol | 279.80 | 377.45 | 417.67 | -40.22 | -10.66 |
| Saccharose | 281.50 | 424.30 | 420.30 | 4.00 | 0.94 |
| Lactose | 283.60 | 412.50 | 423.55 | -11.05 | -2.68 |
| Glyceryl-2-laurate | 290.30 | 436.40 | 433.91 | 2.49 | 0.57 |
| 1-Glyceryl laurate | 291.30 | 447.70 | 435.45 | 12.25 | 2.74 |
| 1,16-Hexadecanediol | 296.50 | 426.18 | 443.49 | -17.31 | -4.06 |
| Hexadecanedioic acid | 301.30 | 482.80 | 450.92 | 31.88 | 6.60 |
| Vitamin B2 | 321.30 | 442.20 | 481.84 | -39.64 | -8.96 |
| 2-Monomyristin | 323.50 | 506.30 | 485.24 | 21.06 | 4.16 |
| Methylprednisolone | 338.50 | 472.10 | 508.44 | -36.34 | -7.70 |
| 2-Monopalmitin | 357.80 | 558.60 | 538.28 | 20.32 | 3.64 |
| 1-Palmitoylglycerol | 358.10 | 566.90 | 538.74 | 28.16 | 4.97 |
| 2-Stearoylglycerol | 391.10 | 610.40 | 589.77 | 20.63 | 3.38 |
| 1-Glyceryl stearate | 391.70 | 610.40 | 590.70 | 19.70 | 3.23 |
| Maltotriose | 406.70 | 662.50 | 613.89 | 48.61 | 7.34 |
| Tetraphenyltetrahydroxycyclotetrasiloxane | 432.00 | 626.50 | 653.01 | -26.51 | -4.23 |
| Cyclodextrin | 776.30 | 1153.00 | 1185.38 | -32.38 | -2.81 |
| Octaphenyltetrahydroxytricyclooctasiloxane | 816.00 | 1273.00 | 1246.77 | 26.23 | 2.06 |
