# Supplementary material for: Calculation of the Isobaric Heat Capacities of the Liquid and Solid Phase of Organic Compounds at and around 298.15 K Based on Their “True” Molecular Volume
Source: Molecules. 2019 Apr 24;24(8):1626. doi: 10.3390/molecules24081626 (PMC6514989; doi:10.3390/molecules24081626)
Supplement: Supplementary file 1 [file molecules-24-01626-s001.zip › Table 11.doc]

| **Molecule name** | **Cp(sol,298) exp** | **Cp(sol,298) calc** | **Deviation** | **Dev. in %** |
| --- | --- | --- | --- | --- |
| Azaferrocene | 183.40 | 196.40 | -13.00 | -7.09 |
| Nickelocene | 202.39 | 200.70 | 1.69 | 0.84 |
| Ferrocene | 195.90 | 201.10 | -5.20 | -2.65 |
| Manganocene | 208.15 | 207.20 | 0.95 | 0.46 |
| Acetylferrocene | 246.00 | 253.40 | -7.40 | -3.01 |
| 1,1'-Diacetylferrocene | 293.90 | 301.60 | -7.70 | -2.62 |
| Benzylferrocene | 312.60 | 320.70 | -8.10 | -2.59 |
| Benzoylferrocene | 313.60 | 323.80 | -10.20 | -3.25 |
| 1,1'-Dibenzoylferrocene | 466.10 | 450.70 | 15.40 | 3.30 |
| **Mean** |  |  | **-3.73** | **-1.85** |
| **Standar deviation** |  |  | **8.95** | **3.37** |
