# Supplementary material for: Calculation of the Isobaric Heat Capacities of the Liquid and Solid Phase of Organic Compounds at and around 298.15 K Based on Their “True” Molecular Volume
Source: Molecules. 2019 Apr 24;24(8):1626. doi: 10.3390/molecules24081626 (PMC6514989; doi:10.3390/molecules24081626)
Supplement: Supplementary file 1 [file molecules-24-01626-s001.zip › Table 12.doc]

| **Molecule name** | **Cp(sol,298) exp** | **Cp(sol,298) calc** | **Deviation** | **Dev. in %** |
| --- | --- | --- | --- | --- |
| Tetraisopropoxysilane | 359.48 | 365.90 | -6.42 | -1.79 |
| Tetrapropyl silicate | 363.39 | 369.80 | -6.41 | -1.76 |
| Tetra-2-butoxysilane | 487.27 | 453.50 | 33.77 | 6.93 |
| Tetra-2-methyl-1-propoxysilane | 477.59 | 459.90 | 17.69 | 3.70 |
| Tetrabutoxysilane | 493.68 | 461.10 | 32.58 | 6.60 |
| 2,4,6-Trimethyl-2,4,6-triphenylcyclotrisiloxane | 506.70 | 490.20 | 16.50 | 3.26 |
| 1,1,3,3-Tetraphenyl-1,3-dimethyldisiloxane | 503.20 | 530.50 | -27.30 | -5.43 |
| Tetra-3-methyl-1-butoxysilane | 591.28 | 549.50 | 41.78 | 7.07 |
| Tetramethyltetraphenylcyclotetrasiloxane | 615.00 | 639.60 | -24.60 | -4.00 |
| Hexaphenyldisiloxane | 681.00 | 680.90 | 0.10 | 0.01 |
| Hexaphenylcyclotrisiloxane | 683.70 | 722.00 | -38.30 | -5.60 |
| 1,1,3,3,5,5-Hexaphenyl-7,7-dimethylcyclotetrasiloxane | 815.50 | 801.20 | 14.30 | 1.75 |
| Tetra-2-ethyl-1-hexoxysilane | 870.88 | 816.50 | 54.38 | 6.24 |
| Octaphenyltetracyclosiloxane | 932.50 | 939.60 | -7.10 | -0.76 |
| Octaphenylpentacyclosilsesquioxane | 1129.00 | 1086.00 | 43.00 | 3.81 |
| Octaphenyltetrahydroxytricyclooctasiloxane | 1273.00 | 1246.80 | 26.20 | 2.06 |
| **Mean** |  |  | **10.64** | **1.38** |
| **Standard deviation** |  |  | **28.70** | **4.40** |
