# Supplementary material for: Calculation of the Isobaric Heat Capacities of the Liquid and Solid Phase of Organic Compounds at and around 298.15 K Based on Their “True” Molecular Volume
Source: Molecules. 2019 Apr 24;24(8):1626. doi: 10.3390/molecules24081626 (PMC6514989; doi:10.3390/molecules24081626)
Supplement: Supplementary file 1 [file molecules-24-01626-s001.zip › Table 13.doc]

| **Molecule name** | **Cp(sol,298) exp** | **Cp(sol,298) calc** | **Deviation** | **Dev. in %** |
| --- | --- | --- | --- | --- |
| Benzene | 97.90 | 116.90 | -19.00 | -19.41 |
| 2,4-Hexadiyne | 133.57 | 128.60 | 4.97 | 3.72 |
| Norbornene | 129.90 | 138.10 | -8.20 | -6.31 |
| Nortricyclene | 129.00 | 140.80 | -11.80 | -9.15 |
| Norbornane | 151.00 | 149.60 | 1.40 | 0.93 |
| Bicyclo[2.2.2]octane | 157.69 | 171.00 | -13.31 | -8.44 |
| Naphthalene | 168.20 | 175.80 | -7.60 | -4.52 |
| exo-Dicyclopentadiene | 188.70 | 179.80 | 8.90 | 4.72 |
| Bullvalene | 190.38 | 189.30 | 1.08 | 0.57 |
| Acenaphthylene | 183.59 | 195.70 | -12.11 | -6.60 |
| 2-Methylnaphthalene | 195.80 | 198.20 | -2.40 | -1.23 |
| 2,2,3,3-Tetramethylbutane | 232.20 | 198.60 | 33.60 | 14.47 |
| Adamantane | 190.00 | 201.30 | -11.30 | -5.95 |
| Acenaphthene | 191.38 | 203.90 | -12.52 | -6.54 |
| Camphene | 235.13 | 205.70 | 29.43 | 12.52 |
| 1,2,4,5-Tetramethylbenzene | 215.10 | 207.30 | 7.80 | 3.63 |
| Biphenyl | 198.17 | 212.40 | -14.23 | -7.18 |
| 1,8-Dimethylnaphthalene | 242.80 | 215.10 | 27.70 | 11.41 |
| Fluorene | 203.13 | 219.20 | -16.07 | -7.91 |
| Guajen | 216.47 | 219.80 | -3.33 | -1.54 |
| 2,7-Dimethylnaphthalene | 204.39 | 220.50 | -16.11 | -7.88 |
| 2,6-Dimethylnaphthalene | 204.20 | 220.50 | -16.30 | -7.98 |
| Pentamethylbenzene | 210.50 | 225.80 | -15.30 | -7.27 |
| Bicyclo[3.3.3]undecane | 213.20 | 232.80 | -19.60 | -9.19 |
| Phenanthrene | 220.30 | 233.80 | -13.50 | -6.13 |
| Anthracene | 210.50 | 234.90 | -24.40 | -11.59 |
| 4-Phenyltoluene | 238.74 | 235.10 | 3.64 | 1.52 |
| Cyclohexylbenzene | 263.17 | 235.90 | 27.27 | 10.36 |
| Diphenylmethane | 233.50 | 236.00 | -2.50 | -1.07 |
| 9,10-Dihydrophenanthrene | 243.80 | 243.30 | 0.50 | 0.21 |
| 9,10-Dihydroanthracene | 219.06 | 244.70 | -25.64 | -11.70 |
| Tolan | 225.90 | 246.10 | -20.20 | -8.94 |
| Hexamethylbenzene | 245.60 | 248.90 | -3.30 | -1.34 |
| trans-Stilbene | 235.00 | 250.00 | -15.00 | -6.38 |
| Tetrahydroanthracene | 247.02 | 251.10 | -4.08 | -1.65 |
| 4-Methylphenanthrene | 262.80 | 251.90 | 10.90 | 4.15 |
| Fluoranthene | 230.25 | 253.50 | -23.25 | -10.10 |
| Pyrene | 229.70 | 256.40 | -26.70 | -11.62 |
| 1,2-Diphenylethane | 253.76 | 257.30 | -3.54 | -1.40 |
| 4,4'-Dimethylbiphenyl | 242.26 | 257.30 | -15.04 | -6.21 |
| [2.2]Paracyclophane | 252.70 | 261.40 | -8.70 | -3.44 |
| Diamantane | 223.40 | 262.10 | -38.70 | -17.32 |
| [2,2]Metaparacyclophane | 261.50 | 266.80 | -5.30 | -2.03 |
| 1,2,3,4,5,6,7,8-Octahydroanthracene | 277.96 | 266.80 | 11.16 | 4.01 |
| 4,5,9,10-Tetrahydropyrene | 257.12 | 273.20 | -16.08 | -6.25 |
| 1,2,3,6,7,8-Hexahydropyrene | 255.54 | 280.50 | -24.96 | -9.77 |
| [2,2]Metacyclophane | 240.60 | 280.60 | -40.00 | -16.63 |
| Triphenylene | 259.20 | 290.70 | -31.50 | -12.15 |
| Perhydrophenanthrene | 289.50 | 291.60 | -2.10 | -0.73 |
| Benz[a]anthracene | 273.60 | 293.00 | -19.40 | -7.09 |
| Tetracene | 260.50 | 294.00 | -33.50 | -12.86 |
| 1,4-Di-t-butylbenzene | 323.15 | 294.90 | 28.25 | 8.74 |
| Tri-t-butylmethane | 354.80 | 300.50 | 54.30 | 15.30 |
| 4-t-Butylbiphenyl | 304.73 | 301.10 | 3.63 | 1.19 |
| anti-trans-Truxane | 275.30 | 303.50 | -28.20 | -10.24 |
| p-Terphenyl | 279.60 | 308.00 | -28.40 | -10.16 |
| m-Terphenyl | 281.00 | 308.70 | -27.70 | -9.86 |
| o-Terphenyl | 274.75 | 309.10 | -34.35 | -12.50 |
| 2,2'-Diindanyl | 332.60 | 313.10 | 19.50 | 5.86 |
| Perylene | 274.90 | 313.20 | -38.30 | -13.93 |
| Triptycene | 283.10 | 319.90 | -36.80 | -13.00 |
| 1-Methyl-7-isopropylphenanthrene | 294.60 | 321.90 | -27.30 | -9.27 |
| [3,3]Paracyclophane | 324.30 | 322.00 | 2.30 | 0.71 |
| Tritane | 308.80 | 332.60 | -23.80 | -7.71 |
| Dicumyl | 321.10 | 341.60 | -20.50 | -6.38 |
| Triphenylethylene | 309.20 | 347.30 | -38.10 | -12.32 |
| 1,1,1-Triphenylethane | 316.70 | 351.80 | -35.10 | -11.08 |
| 1,1,2-Triphenylethane | 319.70 | 352.80 | -33.10 | -10.35 |
| 1,2'-Dinaphthylmethane | 314.60 | 352.90 | -38.30 | -12.17 |
| Pentacene | 311.50 | 353.20 | -41.70 | -13.39 |
| Coronene | 317.17 | 358.00 | -40.83 | -12.87 |
| Cetane | 441.80 | 383.20 | 58.60 | 13.26 |
| 4,4'-Di-t-butylbiphenyl | 412.41 | 389.40 | 23.01 | 5.58 |
| Hexacyclopropylethane | 396.10 | 401.20 | -5.10 | -1.29 |
| 1,1'-Diphenyl-1,1'-bicyclopentyl | 375.50 | 401.80 | -26.30 | -7.00 |
| 1,2,3-Triphenylbenzene | 352.37 | 403.10 | -50.73 | -14.40 |
| p-Quaterphenyl | 363.50 | 404.70 | -41.20 | -11.33 |
| 1,3,5-Triphenylbenzene | 361.00 | 404.70 | -43.70 | -12.11 |
| m-Quaterphenyl | 359.50 | 404.70 | -45.20 | -12.57 |
| o-Quaterphenyl | 359.10 | 406.40 | -47.30 | -13.17 |
| Tetraphenylmethane | 368.20 | 423.30 | -55.10 | -14.96 |
| Octadecane | 485.64 | 428.80 | 56.84 | 11.70 |
| 1,1,1,2-Tetraphenylethane | 395.40 | 440.00 | -44.60 | -11.28 |
| 1,1'-Diphenyl-1,1'-bicyclohexyl | 403.80 | 442.30 | -38.50 | -9.53 |
| 1,1,2,2-Tetraphenylethane | 399.60 | 444.10 | -44.50 | -11.14 |
| Tetraphenylethylene | 388.70 | 445.00 | -56.30 | -14.48 |
| Eicosane | 479.90 | 475.10 | 4.80 | 1.00 |
| o-Quinquephenyl | 444.30 | 500.30 | -56.00 | -12.60 |
| p-Quinquephenyl | 455.50 | 500.60 | -45.10 | -9.90 |
| m-Quinquephenyl | 443.70 | 500.70 | -57.00 | -12.85 |
| 2,3-Dimethyl-2,3-bis(4-t-butylphenyl)butane | 529.50 | 518.20 | 11.30 | 2.13 |
| Docosane | 563.60 | 520.80 | 42.80 | 7.59 |
| 2,11-Dicyclohexyldodecane | 557.30 | 531.40 | 25.90 | 4.65 |
| 1,1-Dicyclohexyldodecane | 562.60 | 532.00 | 30.60 | 5.44 |
| Pentaphenylethane | 473.60 | 534.50 | -60.90 | -12.86 |
| 3,4-Dimethyl-3,4-bis(4-t-butylphenyl)hexane | 587.90 | 561.80 | 26.10 | 4.44 |
| 3,4-Diethyl-3,4-bis(4-t-butylphenyl)-hexane | 631.30 | 600.70 | 30.60 | 4.85 |
| Hexacosane | 661.20 | 612.10 | 49.10 | 7.43 |
| 4,5-Diethyl-4,5-bis-(4-tert-butylphenyl)-octane | 618.50 | 651.50 | -33.00 | -5.34 |
| 2,4,5,7-Tetramethyl-4,5-bis(4-t-butylphenyl)octane | 683.90 | 651.90 | 32.00 | 4.68 |
| Fullerene C70 | 664.21 | 685.90 | -21.69 | -3.27 |
| 4,5-Dipropyl-4,5-bis-(4-t-butylphenyl)octane | 724.20 | 689.60 | 34.60 | 4.78 |
| Dotriacontane | 806.00 | 749.00 | 57.00 | 7.07 |
| 5,6-Dibutyl-5,6-bis(4-t-butylphenyl)decane | 805.50 | 776.90 | 28.60 | 3.55 |
| Hexatriacontane | 881.00 | 840.40 | 40.60 | 4.61 |
| Tetratetracontane | 1049.60 | 1023.00 | 26.60 | 2.53 |
| Pentacontane | 1220.90 | 1159.90 | 61.00 | 5.00 |
| **Mean** |  |  | **-8.83** | **-4.06** |
| **Standard deviation** |  |  | **38.20** | **9.60** |
