# Supplementary material for: Calculation of the Isobaric Heat Capacities of the Liquid and Solid Phase of Organic Compounds at and around 298.15 K Based on Their “True” Molecular Volume
Source: Molecules. 2019 Apr 24;24(8):1626. doi: 10.3390/molecules24081626 (PMC6514989; doi:10.3390/molecules24081626)
Supplement: Supplementary file 1 [file molecules-24-01626-s001.zip › Table 14.doc]

| **Molecules class** | **N** | **Corr. coeff. R^2^** | **σ (J/mol/K)** | **Intercept** | **Slope** |
| --- | --- | --- | --- | --- | --- |
| OH-free compounds | 555 | 0.9766 | 23.14 | 2.8899 | 1.3669 |
| All alcohols/acids, sugars | 242 | 0.9792 | 20.87 | -14.7861 | 1.5364 |
| Monoalcohols/-acids | 123 | 0.9613 | 21.62 | -11.2179 | 1.5050 |
| Polyalcohols/-acids, sugars | 119 | 0.9866 | 19.75 | -14.9656 | 1.5462 |
