# Supplementary material for: Calculation of the Isobaric Heat Capacities of the Liquid and Solid Phase of Organic Compounds at and around 298.15 K Based on Their “True” Molecular Volume
Source: Molecules. 2019 Apr 24;24(8):1626. doi: 10.3390/molecules24081626 (PMC6514989; doi:10.3390/molecules24081626)
Supplement: Supplementary file 1 [file molecules-24-01626-s001.zip › Table 15.doc]

**Table 15**. Statistics of liquid heat capacities of OH-free compounds at various temperatures

| **Cp at various K** | **N** | **Corr. coeff. R^2^** | **σ (J/mol/K)** | **Intercept** | **Slope** |
| --- | --- | --- | --- | --- | --- |
| Cp(liq,250) | 29 | 0.9588 | 16.08 | -10.8602 | 1.7137 |
| Cp(liq,275) | 43 | 0.9765 | 13.58 | -14.8530 | 1.7887 |
| Cp(liq,298) | 1100 | 0.9893 | 20.55 | -6.8820 | 1.8302 |
| Cp(liq,325) | 94 | 0.9860 | 19.56 | -12.6937 | 1.9223 |
| Cp(liq,350) | 87 | 0.9842 | 20.29 | -4.3295 | 1.9458 |
