# Supplementary material for: Calculation of the Isobaric Heat Capacities of the Liquid and Solid Phase of Organic Compounds at and around 298.15 K Based on Their “True” Molecular Volume
Source: Molecules. 2019 Apr 24;24(8):1626. doi: 10.3390/molecules24081626 (PMC6514989; doi:10.3390/molecules24081626)
Supplement: Supplementary file 1 [file molecules-24-01626-s001.zip › Table 16.doc]

**Table 16**. Statistics of liquid heat capacities of OH-carrying compounds at various temperatures

| **Cp at various K** | **N** | **Corr. coeff. R^2^** | **σ (J/mol/K)** | **Intercept** | **Slope** |
| --- | --- | --- | --- | --- | --- |
| Cp(liq,250) | 10 | 0.9702 | 11.04 | 7.4825 | 1.7070 |
| Cp(liq,275) | 36 | 0.8988 | 21.70 | 0.0897 | 1.9866 |
| Cp(liq,298) | 203 | 0.9696 | 24.79 | 17.7056 | 1.9911 |
| Cp(liq,325) | 59 | 0.9337 | 27.08 | 35.6984 | 2.0297 |
| Cp(liq,350) | 56 | 0.9605 | 21.51 | 39.5214 | 2.1639 |
