# Supplementary material for: Calculation of the Isobaric Heat Capacities of the Liquid and Solid Phase of Organic Compounds at and around 298.15 K Based on Their “True” Molecular Volume
Source: Molecules. 2019 Apr 24;24(8):1626. doi: 10.3390/molecules24081626 (PMC6514989; doi:10.3390/molecules24081626)
Supplement: Supplementary file 1 [file molecules-24-01626-s001.zip › Table 17.doc]

**Table 17**. Statistics of solid heat capacities of OH-free compounds at various temperatures

| **Cp at various K** | **N** | **Corr. coeff. R^2^** | **σ (J/mol/K)** | **Intercept** | **Slope** |
| --- | --- | --- | --- | --- | --- |
| Cp(sol,250) | 81 | 0.9873 | 19.37 | 8.8062 | 1.1569 |
| Cp(sol,275) | 83 | 0.9883 | 17.71 | 5.5180 | 1.2659 |
| Cp(sol,298) | 559 | 0.9760 | 23.66 | 2.6919 | 1.3694 |
| Cp(sol,325) | 60 | 0.9721 | 23.48 | -1.7976 | 1.4837 |
| Cp(sol,350) | 43 | 0.9711 | 25.59 | -5.6718 | 1.5794 |
