# Supplementary material for: Calculation of the Isobaric Heat Capacities of the Liquid and Solid Phase of Organic Compounds at and around 298.15 K Based on Their “True” Molecular Volume
Source: Molecules. 2019 Apr 24;24(8):1626. doi: 10.3390/molecules24081626 (PMC6514989; doi:10.3390/molecules24081626)
Supplement: Supplementary file 1 [file molecules-24-01626-s001.zip › Table 18.doc]

**Table 18**. Statistics of solid heat capacities of OH-carrying compounds at various temperatures

| **Cp at various K** | **N** | **Corr. coeff. R^2^** | **σ (J/mol/K)** | **Intercept** | **Slope** |
| --- | --- | --- | --- | --- | --- |
| Cp(sol,250) | 43 | 0.9916 | 15.50 | -12.6827 | 1.2778 |
| Cp(sol,275) | 42 | 0.9846 | 15.10 | -1.1741 | 1.3387 |
| Cp(sol,298) | 241 | 0.9743 | 20.77 | -12.6463 | 1.5236 |
| Cp(sol,325) | 55 | 0.9859 | 21.94 | -11.7895 | 1.6305 |
| Cp(sol,350) | 36 | 0.9846 | 28.61 | -26.4116 | 1.8402 |
