# Supplementary material for: Calculation of the Isobaric Heat Capacities of the Liquid and Solid Phase of Organic Compounds at and around 298.15 K Based on Their “True” Molecular Volume
Source: Molecules. 2019 Apr 24;24(8):1626. doi: 10.3390/molecules24081626 (PMC6514989; doi:10.3390/molecules24081626)
Supplement: Supplementary file 1 [file molecules-24-01626-s001.zip › Table 19.doc]

| **Example** | **T/K** | **Cp(liq,T) exp** | **Cp(liq,T) calc** | **Deviation** | **Dev in %** |
| --- | --- | --- | --- | --- | --- |
| 1-Phenylpyrazole [219] | 275.00 | 222.35 | 223.04 | -0.69 | -0.31 |
|  | 288.20 | 227.50 | 230.51 | -3.01 | -1.32 |
|  | 298.15 | 231.60 | 236.50 | -4.90 | -2.12 |
|  | 308.20 | 235.70 | 238.92 | -3.22 | -1.37 |
|  | 325.00 | 236.19 | 242.97 | -6.78 | -2.87 |
| 1-Octanol [44] | 250.00 | 251.00 | 269.85 | -18.85 | -7.51 |
|  | 260.00 | 268.00 | 284.08 | -16.08 | -6.00 |
|  | 275.00 | 285.00 | 305.43 | -20.43 | -7.17 |
|  | 290.00 | 298.00 | 315.00 | -17.00 | -5.70 |
|  | 298.15 | 312.10 | 320.20 | -8.10 | -2.60 |
|  | 310.00 | 317.50 | 332.32 | -14.82 | -4.67 |
|  | 325.00 | 336.00 | 347.66 | -11.66 | -3.47 |
|  | 340.00 | 355.60 | 362.33 | -6.73 | -1.89 |
|  | 350.00 | 368.30 | 372.11 | -3.81 | -1.04 |
