# Supplementary material for: Calculation of the Isobaric Heat Capacities of the Liquid and Solid Phase of Organic Compounds at and around 298.15 K Based on Their “True” Molecular Volume
Source: Molecules. 2019 Apr 24;24(8):1626. doi: 10.3390/molecules24081626 (PMC6514989; doi:10.3390/molecules24081626)
Supplement: Supplementary file 1 [file molecules-24-01626-s001.zip › Table 2.doc]

| **Cation** | **Vm (A^3^)** | **Anion** | **Vm (A^3^)** |
| --- | --- | --- | --- |
| Methyltributylammonium | 237.06 | Nitrate | 40.26 |
| Pyrrolidinium | 81.56 | Acetate | 54.06 |
| 1-Methyl-1-propylpyrrolidinium | 145.36 | Propionate | 70.56 |
| 1-Butyl-1-methylpyrrolidinium | 161.36 | Butanoate | 87.26 |
| 1-Methyl-1-pentylpyrrolidinium | 178.76 | Pentanoate | 103.96 |
| 1-Hexyl-1-methylpyrrolidinium | 195.46 | Trifluoroacetate | 68.66 |
| 1-Methyl-1-octylpyrrolidinium | 228.86 | Hydrogen sulfate | 58.16 |
| 1-Butyl-1-methylpiperidinium | 177.16 | Methylsulfate | 75.56 |
| 1,3-Dimethylimidazolium | 97.76 | Ethylsulfate | 92.26 |
| 1-Ethyl-3-methylimidazolium | 112.36 | Octylsulfate | 192.46 |
| 1-Butyl-3-methylimidazolium | 146.2 | Methanesulfonate | 66.86 |
| 1-Hexyl-3-methylimidazolium | 178.7 | Trifluoromethanesulfonate | 81.66 |
| 2,3-Dimethyl-1-hexylimidazolium | 196.86 | Toluenesulfonate | 138.36 |
| 1-Octyl-3-methylimidazolium | 203.91 | Docusate | 401.86 |
| 1-Decyl-3-methylimidazolium | 248.36 | Thiocyanate | 54.76 |
| 1-Dodecyl-3-methylimidazolium | 281.46 | Tetrafluoroborate | 46.46 |
| 1-Ethylpyridinium | 114.16 | Tetracyanoborate | 100.26 |
| 1-Propylpyridinium | 129.36 | Dicyanoamide | 57.26 |
| 1-Butylpyridinium | 145.96 | Tricyanomethide | 76.26 |
| 1-Octylpyridinium | 212.36 | Dimethylphosphate | 96.86 |
| 1-Ethyl-3-methylpyridinium | 129.06 | Diethylphosphate | 130.36 |
| 1-Hexyl-3-methylpyridinium | 196.76 | Hexafluorophosphate | 78.36 |
| 1-Ethyl-2-heptylpyridinium | 227.96 | Tris(pentafluoroethyl)trifluorophosphate | 216.96 |
| 1-Ethyl-2-octylpyridinium | 244.56 | Bis(2,2,4-trimethylpentyl)phosphinate | 304.86 |
| 1-Ethyl-2-nonylpyridinium | 261.46 | Bis(trifluoromethylsulfonyl)amide | 156.46 |
| 4-Dimethylamino-1-hexylpyridinium | 224.56 | Saccharinate | 138.06 |
| Ethyl tributylphosphonium | 270.16 | Serinate | 91.26 |
| Tetrabutylphosphonium | 302.96 | L-Valinate | 114.66 |
| Tetradecyl trihexylphosphonium | 570.96 | L-Threoninate | 105.16 |
