# Supplementary material for: Calculation of the Isobaric Heat Capacities of the Liquid and Solid Phase of Organic Compounds at and around 298.15 K Based on Their “True” Molecular Volume
Source: Molecules. 2019 Apr 24;24(8):1626. doi: 10.3390/molecules24081626 (PMC6514989; doi:10.3390/molecules24081626)
Supplement: Supplementary file 1 [file molecules-24-01626-s001.zip › Table 20.doc]

| **Example** | **T/K** | **Cp(sol,T) exp** | **Cp(sol,T) calc** | **Deviation** | **Dev in %** |
| --- | --- | --- | --- | --- | --- |
| 3,4-Dimethoxyphenylacetonitrile [195] | 250 | 208.4 | 204.2 | 4.2 | 2.01 |
|  | 265 | 218.2 | 213.3 | 4.9 | 2.26 |
|  | 275 | 225.2 | 219.3 | 5.9 | 2.61 |
|  | 280 | 228.8 | 222.5 | 6.3 | 2.77 |
|  | 290 | 236.6 | 228.8 | 7.8 | 3.31 |
|  | 298.15 | 243.5 | 233.9 | 9.6 | 3.94 |
|  | 310 | 254.8 | 240.5 | 14.3 | 5.62 |
|  | 320 | 265.6 | 246.0 | 19.6 | 7.37 |
|  | 325 | * | 248.8 |  |  |
| 2-Pyrazinecarboxylic acid [116] | 250 | 125.2 | 118.3 | 6.9 | 5.52 |
|  | 260 | 129 | 125.4 | 3.6 | 2.81 |
|  | 275 | 134.3 | 136.0 | -1.7 | -1.30 |
|  | 290 | 140.2 | 140.5 | -0.3 | -0.24 |
|  | 298.15 | 143.2 | 143.0 | 0.2 | 0.14 |
|  | 310 | 147.5 | 148.4 | -0.9 | -0.63 |
|  | 325 | 153.1 | 155.3 | -2.2 | -1.46 |
|  | 340 | 158.2 | 159.4 | -1.2 | -0.78 |
|  | 350 | 161.7 | 162.2 | -0.5 | -0.31 |
