# Supplementary material for: Calculation of the Isobaric Heat Capacities of the Liquid and Solid Phase of Organic Compounds at and around 298.15 K Based on Their “True” Molecular Volume
Source: Molecules. 2019 Apr 24;24(8):1626. doi: 10.3390/molecules24081626 (PMC6514989; doi:10.3390/molecules24081626)
Supplement: Supplementary file 1 [file molecules-24-01626-s001.zip › Table 3.doc]

| **Molecule name** | **Cp(liq,298) exp** | **Cp(liq,298) calc** | **Deviation** | **Dev. in %** |
| --- | --- | --- | --- | --- |
| Pyrrolidinium nitrate | 228.00 | 215.40 | 12.60 | 5.53 |
| 1-Ethyl-3-methylimidazolium bromide | 264.80 | 256.10 | 8.70 | 3.29 |
| 3,3-Dinitroazetidinium nitrate | 272.95 | 270.60 | 2.35 | 0.86 |
| 1-Propyl-3-methylimidazolium bromide | 281.40 | 286.80 | -5.40 | -1.92 |
| 1-Ethyl-3-methylimidazolium thiocyanate | 281.45 | 295.40 | -13.95 | -4.96 |
| 1-Ethyl-3-methylimidazolium acetate | 321.90 | 296.60 | 25.30 | 7.86 |
| 1-Methyltetrahydrothiophenium dicyanamide | 338.50 | 300.80 | 37.70 | 11.14 |
| 1-Ethyl-3-methylimidazolium tetrafluoroborate | 305.00 | 302.10 | 2.90 | 0.95 |
| 1,3-Dimethylimidazolium methosulfate | 341.00 | 305.20 | 35.80 | 10.50 |
| 1-Butyl-3-methylimidazolium chloride | 317.00 | 305.90 | 11.10 | 3.50 |
| 1-Ethyl-3-methylimidazolium dicyanamide | 314.64 | 314.90 | -0.26 | -0.08 |
| 1-Butyl-3-methylimidazolium bromide | 317.00 | 317.90 | -0.90 | -0.28 |
| 1-Ethyl-3-methylimidazolium methanesulfonate | 345.50 | 322.00 | 23.50 | 6.80 |
| 1-Ethyl-3-methylimidazolium trifluoroacetate | 316.00 | 323.90 | -7.90 | -2.50 |
| 1-Ethyltetrahydrothiophenium dicyanamide | 335.38 | 330.90 | 4.48 | 1.34 |
| 1-Butyl-3-methylimidazolium nitrate | 353.50 | 333.30 | 20.20 | 5.71 |
| 1-Propylpyridinium tetrafluoroborate | 363.00 | 335.40 | 27.60 | 7.60 |
| 1-Ethyl-3-methylimidazolium methylsulfate | 341.00 | 338.70 | 2.30 | 0.67 |
| 1-Ethyl-3-methylimidazolium tricyanomethide | 358.70 | 340.10 | 18.60 | 5.19 |
| 1-Ethyl-3-methylimidazolium hexafluorophosphate | 343.60 | 341.30 | 2.30 | 0.67 |
| 1-Ethylpyridinium hexafluorophosphate | 293.00 | 341.60 | -48.60 | -16.59 |
| 1-Butyl-3-methylimidazolium iodide | 314.00 | 341.70 | -27.70 | -8.82 |
| 1-Benzyl-3-methylimidazolium chloride | 339.40 | 343.80 | -4.40 | -1.30 |
| 1-Ethyl-3-methylimidazolium trifluoromethylsulfonate | 362.80 | 349.60 | 13.20 | 3.64 |
| 1-Butyl-3-methylimidazolium thiocyanate | 385.00 | 356.00 | 29.00 | 7.53 |
| 1-Butyl-3-methylimidazolium acetate | 383.20 | 359.60 | 23.60 | 6.16 |
| 1-Butyl-3-methylimidazolium tetrafluoroborate | 363.00 | 362.50 | 0.50 | 0.14 |
| 1-Methylazepanium methosulfate | 332.08 | 364.90 | -32.82 | -9.88 |
| 1-Butylpyridinium tetrafluoroborate | 383.80 | 365.60 | 18.20 | 4.74 |
| 1-Ethyl-3-methylimidazolium ethosulfate | 378.00 | 366.70 | 11.30 | 2.99 |
| 1-Propylpyridinium hexafluorophosphate | 328.70 | 371.90 | -43.20 | -13.14 |
| 1-Propyl-3-methylimidazolium hexafluorophosphate | 374.40 | 372.00 | 2.40 | 0.64 |
| 1-Butyl-3-methylimidazolium dicyanoamide | 365.00 | 373.00 | -8.00 | -2.19 |
| 1-Ethyl-3-methylimidazolium dimethylphosphate | 411.78 | 375.20 | 36.58 | 8.88 |
| 1-Hexyl-3-methylimidazolium bromide | 344.00 | 376.90 | -32.90 | -9.56 |
| 1-(3-Cyanopropyl)-pyridinium dicyanamide | 422.00 | 379.40 | 42.60 | 10.09 |
| 1-Butyl-3-methylimidazolium trifluoroacetate | 408.20 | 385.10 | 23.10 | 5.66 |
| 1-Butyl-2-methylimidazolium trifluoroacetate | 407.90 | 388.90 | 19.00 | 4.66 |
| 1-Butyltetrahydrothiophenium dicyanamide | 395.19 | 391.40 | 3.79 | 0.96 |
| 1-Butyl-2,3-dimethylimidazolium tetrafluoroborate | 416.01 | 392.40 | 23.61 | 5.68 |
| 1-Butyl-3-methylpyridinium tetrafluoroborate | 388.00 | 392.90 | -4.90 | -1.26 |
| 1-Butyl-4-methylpyridinium tetrafluoroborate | 414.00 | 393.00 | 21.00 | 5.07 |
| 1-(3-Cyanopropyl)-2,3-dimethylimidazolium tetrafluoroborate | 339.00 | 396.70 | -57.70 | -17.02 |
| 1-Ethyl-3-methylpyridinium ethylsulfate | 389.00 | 397.10 | -8.10 | -2.08 |
| 1-Butyl-3-methylimidazolium methosulfate | 416.00 | 397.30 | 18.70 | 4.50 |
| 1-Benzyl-3-methylimidazolium tetrafluoroborate | 387.50 | 400.00 | -12.50 | -3.23 |
| 1-Butyl-3-methylimidazolium hexafluorophosphate | 407.70 | 402.10 | 5.60 | 1.37 |
| 1-Butyl-1-methylpyrrolidinium dicyanamide | 413.00 | 405.30 | 7.70 | 1.86 |
| 1-(3-Cyanopropyl)-2,3-dimethylimidazolium dicyanamide | 444.00 | 408.90 | 35.10 | 7.91 |
| 1-Butyl-3-methylimidazolium trifluoromethylsulfonate | 417.00 | 409.70 | 7.30 | 1.75 |
| 1-Hexyl-3-methylimidazolium tetrafluoroborate | 416.00 | 421.50 | -5.50 | -1.32 |
| 1,2-Diethylpyridinium ethylsulfate | 412.00 | 427.80 | -15.80 | -3.83 |
| 1-Butyl-2,3-dimethylimidazolium hexafluorophosphate | 433.60 | 432.40 | 1.20 | 0.28 |
| 1-Pentyl-3-methylimidazolium hexafluorophosphate | 437.40 | 432.90 | 4.50 | 1.03 |
| 1-Butyl-1-methylpyrrolidinium trifluoromethanesulfonate | 435.00 | 437.40 | -2.40 | -0.55 |
| 1-Octyl-3-methylimidazolium bromide | 392.00 | 438.00 | -46.00 | -11.73 |
| 1-Ethyl-3-methylimidazolium toluenesulfonate | 484.20 | 451.10 | 33.10 | 6.84 |
| 1-Methyl-3-propylimidazolium 2-amino-4-carboxybutanoate | 517.10 | 456.80 | 60.30 | 11.66 |
| 1-Hexyl-3-methylimidazolium hexafluorophosphate | 424.00 | 461.50 | -37.50 | -8.84 |
| 1-Hexyl-3-methylimidazolium trifluoromethylsulfonate | 502.30 | 470.00 | 32.30 | 6.43 |
| Ethyl 1-ethylnicotinate ethosulfate | 513.00 | 476.70 | 36.30 | 7.08 |
| 1-Butyl-1-methylpyrrolidinium tetracyanoborate | 524.00 | 482.50 | 41.50 | 7.92 |
| 1-Octyl-3-methylimidazolium tetrafluoroborate | 498.00 | 483.10 | 14.90 | 2.99 |
| 1-Ethyl-3-methylimidazolium bis(trifluoromethanesulfonyl) amide | 500.00 | 483.20 | 16.80 | 3.36 |
| 1-Benzyl-3-methylimidazolium tetrafluoroethanesulfonate | 502.80 | 485.10 | 17.70 | 3.52 |
| 1-Butyl-3-methylpyridinium tetracyanoborate | 495.00 | 487.00 | 8.00 | 1.62 |
| N-Ethylpyridinium bis(trifluoromethylsulfonyl)amide | 502.15 | 487.20 | 14.95 | 2.98 |
| 1-Ethyl-3-methylimidazolium 2-(2-methoxyethoxy)ethylsulfate | 526.00 | 492.90 | 33.10 | 6.29 |
| 1-Heptyl-3-methylimidazolium hexafluorophosphate | 500.60 | 493.90 | 6.70 | 1.34 |
| 1-Ethyl-2,3-dimethylimidazolium bis(trifluoromethanesulfonyl) amide | 493.00 | 508.50 | -15.50 | -3.14 |
| 1-Methyl-3-butylimidazolium saccharinate | 565.66 | 509.30 | 56.36 | 9.96 |
| 1-Isopropyl-3-methylimidazolium bis(trifluoromethanesulfonyl) amide | 529.90 | 512.70 | 17.20 | 3.25 |
| 1-Propyl-3-methylimidazolium bis(trifluoromethanesulfonyl) amide | 534.90 | 513.10 | 21.80 | 4.08 |
| 1-Butyl-3-methylimidazolium toluenesulfonate | 548.40 | 514.40 | 34.00 | 6.20 |
| N-Ethyl-2-methylpyridinium bis(trifluoromethylsulfonyl)amide | 534.50 | 515.50 | 19.00 | 3.55 |
| 1-Octyl-3-methylimidazolium hexafluorophosphate | 536.10 | 524.50 | 11.60 | 2.16 |
| 1-Octyl-3-methylimidazolium trifluoromethylsulfonate | 577.70 | 530.70 | 47.00 | 8.14 |
| 1-Cyclopropylmethyl-3-methylimidazolium bis(trifluoromethanesulfonyl) amide | 539.10 | 532.60 | 6.50 | 1.21 |
| Trimethyl butylammonium bis(trifluoromethylsulfonyl)amide | 559.20 | 536.70 | 22.50 | 4.02 |
| 1,2-Diethylpyridinium bis(trifluoromethanesulfonyl) amide | 566.10 | 538.80 | 27.30 | 4.82 |
| 1-Butyl-3-methylimidazolium bis(trifluoromethanesulfonyl) amide | 536.00 | 542.00 | -6.00 | -1.12 |
| 1-sec-Butyl-3-methylimidazolium bis(trifluoromethanesulfonyl) amide | 557.10 | 542.40 | 14.70 | 2.64 |
| 1-Isobutyl-3-methylimidazolium bis(trifluoromethanesulfonyl) amide | 557.10 | 543.30 | 13.80 | 2.48 |
| 1-Methyl-1-propylpyrrolidinium bis(trifluoromethanesulfonyl) amide | 554.00 | 543.30 | 10.70 | 1.93 |
| 1-Propyl-2,3-dimethylimidazolium bis(trifluoromethanesulfonyl) amide | 554.50 | 543.60 | 10.90 | 1.97 |
| N-Propyl-2-methylpyridinium bis(trifluoromethylsulfonyl)amide | 557.96 | 545.80 | 12.16 | 2.18 |
| N-Butylpyridinium bis(trifluoromethanesulfonyl) amide | 566.52 | 546.70 | 19.82 | 3.50 |
| N-Propyl-3-methylpyridinium bis(trifluoromethylsulfonyl)amide | 517.00 | 547.20 | -30.20 | -5.84 |
| 1-Nonyl-3-methylimidazolium hexafluorophosphate | 569.40 | 553.50 | 15.90 | 2.79 |
| N-Octylisoquinolinium thiocyanate | 522.00 | 557.90 | -35.90 | -6.88 |
| N-Ethyl-4-dimethylaminopyridinium bis(trifluoromethanesulfonyl) amide | 594.30 | 568.30 | 26.00 | 4.37 |
| 1-Pentyl-3-methylimidazolium bis(trifluoromethanesulfonyl) amide | 595.60 | 571.30 | 24.30 | 4.08 |
| 1-Ethyl-2-propylpyridinium bis(trifluoromethanesulfonyl) amide | 593.90 | 574.60 | 19.30 | 3.25 |
| 1-Isobutyl-1-methylpyrrolidinium bis(trifluoromethylsulfonyl)amide | 582.20 | 576.20 | 6.00 | 1.03 |
| 1-Isobutyl-3-methylpyridinium bis(trifluoromethylsulfonyl)amide | 579.00 | 576.70 | 2.30 | 0.40 |
| N-Butyl-3-methylpyridinium bis(trifluoromethylsulfonyl)amide | 578.10 | 577.30 | 0.80 | 0.14 |
| 1-Butyl-1-methylpyrrolidinium bis(trifluoromethylsulfonyl)amide | 572.00 | 578.00 | -6.00 | -1.05 |
| 1-Butyl-3-cyanopyridinium bis(trifluoromethylsulfonyl)amide | 586.00 | 579.50 | 6.50 | 1.11 |
| 1-Benzyl-3-methylimidazolium bis(trifluoromethylsulfonyl) amide | 607.80 | 581.40 | 26.40 | 4.34 |
| 1-Decyl-3-methylimidazolium hexafluorophosphate | 603.30 | 585.00 | 18.30 | 3.03 |
| 1-Cyclopentylmethyl-3-methylimidazolium bis(trifluoromethanesulfonyl) amide | 560.60 | 586.30 | -25.70 | -4.58 |
| Tri(butyl) methylphosphonium methylsulfate | 617.80 | 592.70 | 25.10 | 4.06 |
| 1-Hexyl-3-methylimidazolium bis(trifluoromethanesulfonyl) amide | 629.20 | 603.80 | 25.40 | 4.04 |
| 1-Butyl-1-methylpiperidinium bis(trifluoromethylsulfonyl)amide | 607.50 | 606.10 | 1.40 | 0.23 |
| 1-Ethyl-2-butylpyridinium bis(trifluoromethanesulfonyl) amide | 623.60 | 606.20 | 17.40 | 2.79 |
| N-Hexylpyridinium bis(trifluoromethanesulfonyl) amide | 612.00 | 606.90 | 5.10 | 0.83 |
| 1-Methyl-1-pentylpyrrolidinium bis(trifluoromethanesulfonyl) amide | 622.60 | 608.40 | 14.20 | 2.28 |
| 1-Butyl-3-methylimidazolium octylsulfate | 635.00 | 610.10 | 24.90 | 3.92 |
| 1-Cyclohexylmethyl-3-methylimidazolium bis(trifluoromethanesulfonyl) amide | 617.10 | 615.50 | 1.60 | 0.26 |
| N-Butyl-4-dimethylaminopyridinium bis(trifluoromethanesulfonyl) amide | 657.71 | 626.70 | 31.01 | 4.71 |
| 1-Heptyl-3-methylimidazolium bis(trifluoromethanesulfonyl) amide | 659.20 | 630.80 | 28.40 | 4.31 |
| 2,3-Dimethyl-1-Hexylimidazolium bis(trifluoromethanesulfonyl) amide | 686.00 | 632.30 | 53.70 | 7.83 |
| 1-Ethyl-2-pentylpyridinium bis(trifluoromethanesulfonyl) amide | 652.70 | 636.60 | 16.10 | 2.47 |
| 1-Hexyl-3-methylpyridinium bis(trifluoromethylsulfonyl)amide | 624.00 | 637.70 | -13.70 | -2.20 |
| 1-Hexyl-1-methylpyrrolidinium bis(trifluoromethanesulfonyl) amide | 655.10 | 638.80 | 16.30 | 2.49 |
| 1-Hexyl-4-cyanopyridinium bis(trifluoromethylsulfonyl)amide | 633.00 | 639.60 | -6.60 | -1.04 |
| 1-Hexyl-3-cyanopyridinium bis(trifluoromethylsulfonyl)amide | 658.00 | 639.70 | 18.30 | 2.78 |
| 1-Dodecyl-3-methylimidazolium hexafluorophosphate | 666.30 | 646.50 | 19.80 | 2.97 |
| 1-Octyl-3-methylimidazolium bis(trifluoromethanesulfonyl) amide | 654.00 | 664.00 | -10.00 | -1.53 |
| 1-Methyl-1-heptylpyrrolidinium bis(trifluoromethanesulfonyl) amide | 685.10 | 664.80 | 20.30 | 2.96 |
| 1-Octylpyridinium bis(trifluoromethylsulfonyl)amide | 686.00 | 665.90 | 20.10 | 2.93 |
| 1-Ethyl-2-hexylpyridinium bis(trifluoromethanesulfonyl) amide | 685.50 | 667.10 | 18.40 | 2.68 |
| 1-Hexyl-3,5-dimethylpyridinium bis(trifluoromethylsulfonyl)amide | 620.00 | 667.80 | -47.80 | -7.71 |
| 1-(3,4,5,6-Perfluorohexyl)-3-methylimidazolium-3-methylimidazolium bis(trifluoromethanesulfonyl) amide | 725.00 | 678.60 | 46.40 | 6.40 |
| 4-Dimethylamino-1-hexylpyridinium bis(trifluoromethanesulfonyl) amide | 731.00 | 687.20 | 43.80 | 5.99 |
| N-Octyl-3-methylpyridinium bis(trifluoromethanesulfonyl) amide | 669.00 | 697.10 | -28.10 | -4.20 |
| 1-Ethyl-2-heptylpyridinium bis(trifluoromethanesulfonyl) amide | 717.50 | 697.40 | 20.10 | 2.80 |
| 1-Methyl-1-octylpyrrolidinium bis(trifluoromethanesulfonyl) amide | 716.30 | 699.40 | 16.90 | 2.36 |
| 1-Octyl-3-cyanopyridinium bis(trifluoromethylsulfonyl)amide | 709.00 | 700.10 | 8.90 | 1.26 |
| 1-Hexyl-3-methylimidazolium tris(pentafluoroethyl)trifluorophosphate | 730.00 | 710.60 | 19.40 | 2.66 |
| N-Hexyl-3-methyl-4-dimethylaminopyridinium bis(trifluoromethanesulfonyl) amide | 725.00 | 710.90 | 14.10 | 1.94 |
| Butyl 1-butylnicotinate bis(trifluoromethylsulfonyl)amide | 707.00 | 713.40 | -6.40 | -0.91 |
| Ethyl tri(butyl)phosphonium diethylphosphate | 711.00 | 723.00 | -12.00 | -1.69 |
| 1-Decyl-3-methylimidazolium bis(trifluoromethanesulfonyl) amide | 754.80 | 724.40 | 30.40 | 4.03 |
| 1-Ethyl-2-octylpyridinium bis(trifluoromethanesulfonyl) amide | 749.40 | 725.00 | 24.40 | 3.26 |
| 1-Ethyl-2-nonylpyridinium bis(trifluoromethanesulfonyl) amide | 778.50 | 751.40 | 27.10 | 3.48 |
| Tetrabutylphosphonium L-valinate | 747.00 | 753.40 | -6.40 | -0.86 |
| 1-Methyl-1-decylpyrrolidinium bis(trifluoromethanesulfonyl) amide | 778.90 | 760.20 | 18.70 | 2.40 |
| 1-Dodecyl-3-methylimidazolium bis(trifluoromethanesulfonyl) amide | 820.20 | 787.10 | 33.10 | 4.04 |
| 1-Ethyl-2-decylpyridinium bis(trifluoromethanesulfonyl) amide | 811.20 | 788.60 | 22.60 | 2.79 |
| 1-Hexyl-2-propyl-3,5-diethylpyridinium bis(trifluoromethylsulfonyl)amide | 766.00 | 809.50 | -43.50 | -5.68 |
| Trihexyl tetradecyl phosphonium acetate | 1078.20 | 1129.60 | -51.40 | -4.77 |
| Tetradecyl trihexylphosphonium bis(trifluoromethylsulfonyl)amide | 1298.80 | 1316.30 | -17.50 | -1.35 |
| Trihexyltetradecylphosphonium tris(pentafluoroethyl)trifluorophosphate | 1441.40 | 1435.80 | 5.60 | 0.39 |
| Trihexyltetradecylphosphonium bis(2,2,4-trimethylpentyl)phosphinate | 1635.00 | 1587.20 | 47.80 | 2.92 |
| **Mean** |  |  | **9.30** | **1.60** |
| **Standard deviation** |  |  | **24.49** | **5.14** |
