# Supplementary material for: Calculation of the Isobaric Heat Capacities of the Liquid and Solid Phase of Organic Compounds at and around 298.15 K Based on Their “True” Molecular Volume
Source: Molecules. 2019 Apr 24;24(8):1626. doi: 10.3390/molecules24081626 (PMC6514989; doi:10.3390/molecules24081626)
Supplement: Supplementary file 1 [file molecules-24-01626-s001.zip › Table 4.doc]

| **Molecule name** | **Cp(liq,298) exp** | **Cp(liq,298) calc** | **Deviation** | **Dev. in %** |
| --- | --- | --- | --- | --- |
| Tetramethyoxysilane | 240.50 | 239.40 | 1.10 | 0.46 |
| Hexamethyldisiloxane | 309.09 | 300.80 | 8.29 | 2.68 |
| 2,4,6,8-Tetramethylcyclotetrasiloxane | 354.40 | 348.80 | 5.60 | 1.58 |
| Hexamethylcyclotrisiloxane | 360.00 | 350.90 | 9.10 | 2.53 |
| Tetraethoxysilane | 364.40 | 361.30 | 3.10 | 0.85 |
| Octamethyltrisiloxane | 420.64 | 416.20 | 4.44 | 1.06 |
| Octamethylcyclotetrasiloxane | 495.94 | 459.90 | 36.04 | 7.27 |
| 1,1,3,3-Tetraethyl-5,5-dimethylcyclotrisiloxane | 502.90 | 471.10 | 31.80 | 6.32 |
| Octamethyltetrasiloxane | 509.60 | 474.50 | 35.10 | 6.89 |
| Tetrapropoxysilane | 460.10 | 482.70 | -22.60 | -4.91 |
| 1,1,3,3-Tetramethyl-1,3-diphenyldisiloxane | 508.10 | 499.40 | 8.70 | 1.71 |
| 1,1,1,3,5,5,5-Heptamethyl-3-phenyltrisiloxane | 519.60 | 517.10 | 2.50 | 0.48 |
| Decamethyltetrasiloxane | 538.77 | 529.60 | 9.17 | 1.70 |
| Hexaethylcyclotrisiloxane | 535.10 | 531.60 | 3.50 | 0.65 |
| Decamethylcyclopentasiloxane | 634.63 | 574.20 | 60.43 | 9.52 |
| 1,1,1,5,5,5-Hexamethyl-3,3-diphenyltrisiloxane | 648.00 | 601.30 | 46.70 | 7.21 |
| Tetrabutoxysilane | 580.20 | 604.20 | -24.00 | -4.14 |
| 1,1-Diphenyl-3,3,5,5,7,7-hexamethylcyclotetrasiloxane | 633.00 | 664.30 | -31.30 | -4.94 |
| 1,1,3,3-Tetraethyl-5,5-diphenylcylotrisiloxane | 629.60 | 667.60 | -38.00 | -6.04 |
| Octaethylcyclotetrasiloxane | 746.00 | 695.50 | 50.50 | 6.77 |
| Tetraheptoxysilane | 909.40 | 968.50 | -59.10 | -6.50 |
| Tetraoctoxysilane | 1095.40 | 1089.90 | 5.50 | 0.50 |
| Tetradecoxysilane | 1373.50 | 1332.90 | 40.60 | 2.96 |
| **Mean** |  |  | **8.14** | **1.50** |
| **Standard deviation** |  |  | **30.22** | **4.69** |
