# Supplementary material for: Calculation of the Isobaric Heat Capacities of the Liquid and Solid Phase of Organic Compounds at and around 298.15 K Based on Their “True” Molecular Volume
Source: Molecules. 2019 Apr 24;24(8):1626. doi: 10.3390/molecules24081626 (PMC6514989; doi:10.3390/molecules24081626)
Supplement: Supplementary file 1 [file molecules-24-01626-s001.zip › Table 5.doc]

| **Molecule name** | **Cp(liq,298) exp** | **Cp(liq,298) calc** | **Deviation** | **Dev. in %** |
| --- | --- | --- | --- | --- |
| Cyclopropane | 81.20 | 94.00 | -12.80 | -15.76 |
| Propylene | 102.00 | 96.40 | 5.60 | 5.49 |
| s-trans-1,3-Butadiene | 123.65 | 116.90 | 6.75 | 5.46 |
| 1,2-Butadiene | 123.00 | 117.30 | 5.70 | 4.63 |
| 1-Butyne | 132.60 | 118.90 | 13.70 | 10.33 |
| 2-Butyne | 124.30 | 119.10 | 5.20 | 4.18 |
| Cyclobutane | 106.30 | 120.40 | -14.10 | -13.26 |
| Cyclopentadiene | 115.30 | 122.50 | -7.20 | -6.24 |
| cis-2-Butene | 127.00 | 124.90 | 2.10 | 1.65 |
| Isobutylene | 121.30 | 126.00 | -4.70 | -3.87 |
| trans-2-Butene | 124.30 | 126.10 | -1.80 | -1.45 |
| 1-Butene | 128.96 | 126.80 | 2.16 | 1.67 |
| Cyclopentene | 122.38 | 135.30 | -12.92 | -10.56 |
| Butane | 140.90 | 136.90 | 4.00 | 2.84 |
| Methylenecyclobutane | 133.60 | 138.20 | -4.60 | -3.44 |
| Spiro[2.2]pentane | 134.52 | 142.60 | -8.08 | -6.01 |
| Isoprene | 151.08 | 145.20 | 5.88 | 3.89 |
| cis-1,3-Pentadiene | 146.57 | 146.20 | 0.37 | 0.25 |
| Benzene | 136.80 | 146.30 | -9.50 | -6.94 |
| 1,4-Pentadiene | 146.82 | 146.70 | 0.12 | 0.08 |
| 3-Methyl-1,2-butadiene | 152.42 | 146.90 | 5.52 | 3.62 |
| 2,3-Pentadiene | 152.34 | 147.00 | 5.34 | 3.51 |
| trans-1,3-Pentadiene | 149.33 | 147.00 | 2.33 | 1.56 |
| 1,2-Pentadiene | 150.83 | 147.70 | 3.13 | 2.08 |
| Cyclopentane | 128.80 | 148.50 | -19.70 | -15.30 |
| Trimethylethylene | 152.80 | 154.50 | -1.70 | -1.11 |
| Isopentene | 157.30 | 155.70 | 1.60 | 1.02 |
| cis-2-Pentene | 151.80 | 156.10 | -4.30 | -2.83 |
| 1,3-Cyclohexadiene | 141.30 | 156.30 | -15.00 | -10.62 |
| trans-2-Pentene | 157.00 | 156.40 | 0.60 | 0.38 |
| 3-Methyl-1-butene | 156.10 | 156.80 | -0.70 | -0.45 |
| 1-Pentene | 155.30 | 157.10 | -1.80 | -1.16 |
| 2,5-Norbornadiene | 161.20 | 157.50 | 3.70 | 2.30 |
| 1,4-Cyclohexadiene | 142.20 | 158.30 | -16.10 | -11.32 |
| 3-Methylcyclopentene | 152.30 | 165.40 | -13.10 | -8.60 |
| 1-Methylcyclopentene | 153.10 | 166.00 | -12.90 | -8.43 |
| Isopentane | 164.85 | 166.50 | -1.65 | -1.00 |
| 2,2-Dimethylpropane | 153.10 | 166.90 | -13.80 | -9.01 |
| Pentane | 167.19 | 167.20 | -0.01 | -0.01 |
| Cyclohexene | 152.90 | 167.40 | -14.50 | -9.48 |
| Quadricyclane | 157.60 | 168.10 | -10.50 | -6.66 |
| Methylbenzene | 158.70 | 176.60 | -17.90 | -11.28 |
| 1,5-Hexadiene | 133.10 | 177.20 | -44.10 | -33.13 |
| Cyclohexane | 158.10 | 177.30 | -19.20 | -12.14 |
| Methylcyclopentane | 158.70 | 178.40 | -19.70 | -12.41 |
| Tetramethylethene | 174.68 | 183.00 | -8.32 | -4.76 |
| cis-2-Hexene | 178.36 | 186.00 | -7.64 | -4.28 |
| 3,3-Dimethyl-1-butene | 188.30 | 186.50 | 1.80 | 0.96 |
| 1-Hexene | 183.30 | 187.50 | -4.20 | -2.29 |
| Ethynylbenzene | 180.10 | 188.20 | -8.10 | -4.50 |
| 1,3,5-Cycloheptatriene | 162.76 | 189.30 | -26.54 | -16.31 |
| Cyclooctatetraene | 185.18 | 194.20 | -9.02 | -4.87 |
| Norcarane | 187.90 | 194.80 | -6.90 | -3.67 |
| Styrene | 201.90 | 195.50 | 6.40 | 3.17 |
| Cycloheptene | 171.70 | 195.50 | -23.80 | -13.86 |
| Neohexane | 189.67 | 196.50 | -6.83 | -3.60 |
| 2,3-Dimethylbutane | 188.80 | 196.60 | -7.80 | -4.13 |
| 4-Methylcyclohexene | 180.42 | 196.60 | -16.18 | -8.97 |
| 1-Ethylcyclopentene | 188.30 | 196.90 | -8.60 | -4.57 |
| Methylenecyclohexane | 177.40 | 196.90 | -19.50 | -10.99 |
| Ethylidenecyclopentane | 181.20 | 197.00 | -15.80 | -8.72 |
| 3-Methylpentane | 190.83 | 197.20 | -6.37 | -3.34 |
| 2-Methylpentane | 193.96 | 197.40 | -3.44 | -1.77 |
| Hexane | 197.66 | 197.50 | 0.16 | 0.08 |
| Cycloheptane | 180.61 | 204.50 | -23.89 | -13.23 |
| Ethylbenzene | 185.78 | 205.70 | -19.92 | -10.72 |
| Bicyclo[2.2.2]oct-2-ene | 156.73 | 205.90 | -49.17 | -31.37 |
| 1,2-Dimethylbenzene | 187.65 | 206.10 | -18.45 | -9.83 |
| 1,3-Dimethylbenzene | 183.18 | 207.00 | -23.82 | -13.00 |
| 1,4-Dimethylbenzene | 183.65 | 207.10 | -23.45 | -12.77 |
| Indene | 186.94 | 207.10 | -20.16 | -10.78 |
| Methylcyclohexane | 184.96 | 207.50 | -22.54 | -12.19 |
| cis-1,2-Dimethylcyclopentane | 190.80 | 207.80 | -17.00 | -8.91 |
| Ethylcyclopentane | 187.40 | 208.20 | -20.80 | -11.10 |
| trans-1,3-Dimethylcyclopentane | 190.80 | 208.50 | -17.70 | -9.28 |
| 1,1-Dimethylcyclopentane | 187.40 | 208.70 | -21.30 | -11.37 |
| 1,5-Cyclooctadiene | 208.10 | 209.10 | -1.00 | -0.48 |
| Indane | 190.25 | 215.80 | -25.55 | -13.43 |
| 1-Heptene | 211.79 | 217.80 | -6.01 | -2.84 |
| endo-2-Methylnorbornane | 184.30 | 218.90 | -34.60 | -18.77 |
| exo-2-Methylnorbornane | 185.80 | 219.30 | -33.50 | -18.03 |
| trans-Bicyclo[3.3.0]octane | 180.30 | 220.30 | -40.00 | -22.19 |
| cis-Bicyclo[3.3.0]octane | 213.40 | 220.30 | -6.90 | -3.23 |
| cis-Bicyclo[4,2,0]octane | 258.60 | 221.20 | 37.40 | 14.46 |
| cis- Cyclooctene | 207.80 | 222.90 | -15.10 | -7.27 |
| Naphthalene | 196.06 | 224.70 | -28.64 | -14.61 |
| alpha-Methylstyrene | 202.10 | 224.70 | -22.60 | -11.18 |
| Ethylidenecyclohexane | 203.80 | 225.40 | -21.60 | -10.60 |
| Triptane | 213.51 | 225.90 | -12.39 | -5.80 |
| 3,3-Dimethylpentane | 214.80 | 226.40 | -11.60 | -5.40 |
| 2,2-Dimethylpentane | 221.12 | 226.80 | -5.68 | -2.57 |
| 3-Ethylpentane | 219.58 | 227.00 | -7.42 | -3.38 |
| 2,3-Dimethylpentane | 218.30 | 227.20 | -8.90 | -4.08 |
| 2,4-Dimethylpentane | 224.22 | 227.40 | -3.18 | -1.42 |
| Heptane | 225.33 | 227.80 | -2.47 | -1.10 |
| 2-Methylhexane | 222.92 | 227.90 | -4.98 | -2.23 |
| Allylcyclopentane | 202.90 | 228.40 | -25.50 | -12.57 |
| Hemimellitene | 216.44 | 233.90 | -17.46 | -8.07 |
| 1,2,4-Trimethylbenzene | 213.11 | 234.50 | -21.39 | -10.04 |
| Isocumene | 214.72 | 235.10 | -20.38 | -9.49 |
| Cumene | 215.40 | 235.10 | -19.70 | -9.15 |
| 1,1-Dimethylcyclohexane | 209.24 | 236.50 | -27.26 | -13.03 |
| cis-1,4-Dimethylcyclohexane | 212.09 | 236.70 | -24.61 | -11.60 |
| trans-1,3-Dimethylcyclohexane | 212.84 | 236.70 | -23.86 | -11.21 |
| trans-1,2-Dimethylcyclohexane | 209.41 | 237.30 | -27.89 | -13.32 |
| 1,3,5-Trimethylbenzene | 207.85 | 237.50 | -29.65 | -14.27 |
| Ethylcyclohexane | 211.79 | 237.60 | -25.81 | -12.19 |
| Cyclooctane | 215.53 | 237.70 | -22.17 | -10.29 |
| Diisobutylene | 240.20 | 237.80 | 2.40 | 1.00 |
| trans-1,4-Dimethylcyclohexane | 210.25 | 238.00 | -27.75 | -13.20 |
| cis-1,3-Dimethylcyclohexane | 209.37 | 238.10 | -28.73 | -13.72 |
| Propylcyclopentane | 216.27 | 238.50 | -22.23 | -10.28 |
| 1-Octyne | 242.14 | 240.40 | 1.74 | 0.72 |
| 4-Octyne | 233.60 | 240.60 | -7.00 | -3.00 |
| 1,2,3,4-Tetrahydronaphthalene | 217.44 | 246.50 | -29.06 | -13.36 |
| trans-2-Octene | 239.30 | 247.50 | -8.20 | -3.43 |
| Caprylene | 241.21 | 248.20 | -6.99 | -2.90 |
| cis-Hydrindan | 214.18 | 249.20 | -35.02 | -16.35 |
| trans-Hydrindan | 209.70 | 249.50 | -39.80 | -18.98 |
| cis-Bicyclo[6.1.0]nonane | 235.10 | 252.90 | -17.80 | -7.57 |
| 1-Methylnaphthalene | 224.39 | 253.90 | -29.51 | -13.15 |
| 2-Methylnaphthalene | 228.00 | 254.50 | -26.50 | -11.62 |
| Isooctane | 242.49 | 254.50 | -12.01 | -4.95 |
| 2,3,4-Trimethylpentane | 247.32 | 255.10 | -7.78 | -3.15 |
| 2,3,3-Trimethylpentane | 245.56 | 256.30 | -10.74 | -4.37 |
| 3,3-Dimethylhexane | 246.60 | 256.60 | -10.00 | -4.06 |
| 2,2,3-Trimethylpentane | 245.60 | 256.70 | -11.10 | -4.52 |
| 3-Methylheptane | 250.20 | 257.00 | -6.80 | -2.72 |
| 2,5-Dimethylhexane | 249.20 | 257.00 | -7.80 | -3.13 |
| 2-Methylheptane | 252.00 | 257.10 | -5.10 | -2.02 |
| Allylcyclohexane | 233.50 | 257.40 | -23.90 | -10.24 |
| 4-Methylheptane | 251.08 | 258.10 | -7.02 | -2.80 |
| Octane | 255.68 | 258.20 | -2.52 | -0.99 |
| Tetrahydrodicyclopentadiene | 236.50 | 261.10 | -24.60 | -10.40 |
| Prehnitene | 244.30 | 262.50 | -18.20 | -7.45 |
| t-Butylbenzene | 241.59 | 264.90 | -23.31 | -9.65 |
| p-Cymene | 242.30 | 264.90 | -22.60 | -9.33 |
| Butylbenzene | 243.50 | 265.50 | -22.00 | -9.03 |
| 1,2,4,5-Tetramethylbenzene | 220.10 | 266.60 | -46.50 | -21.13 |
| Propylcyclohexane | 242.04 | 267.90 | -25.86 | -10.68 |
| Butylcyclopentane | 245.35 | 268.80 | -23.45 | -9.56 |
| 3-Carene | 254.26 | 271.40 | -17.14 | -6.74 |
| Biphenyl | 259.54 | 273.40 | -13.86 | -5.34 |
| 4,7-Dimethylindane | 241.50 | 275.30 | -33.80 | -14.00 |
| 1,1-Dimethylindane | 249.40 | 275.50 | -26.10 | -10.47 |
| 4,6-Dimethylindane | 240.90 | 275.60 | -34.70 | -14.40 |
| Dihydropinene | 251.26 | 277.10 | -25.84 | -10.28 |
| trans-Decalin | 229.17 | 278.60 | -49.43 | -21.57 |
| 1-Nonene | 270.36 | 278.60 | -8.24 | -3.05 |
| Bicyclopentyl | 238.90 | 279.20 | -40.30 | -16.87 |
| Tetraethylmethane | 278.20 | 281.40 | -3.20 | -1.15 |
| 2,2,4,4-Tetramethylpentane | 266.30 | 283.10 | -16.80 | -6.31 |
| 2,7-Dimethylnaphthalene | 251.85 | 284.20 | -32.35 | -12.84 |
| 2,2,3,3-Tetramethylpentane | 271.50 | 285.40 | -13.90 | -5.12 |
| 1-Methyl-4-isopropylcyclohexene | 257.80 | 286.00 | -28.20 | -10.94 |
| Nonane | 284.43 | 288.40 | -3.97 | -1.40 |
| Neopentylbenzene | 271.90 | 295.10 | -23.20 | -8.53 |
| t-Butylcyclohexane | 264.80 | 295.60 | -30.80 | -11.63 |
| Butylcyclohexane | 271.04 | 298.30 | -27.26 | -10.06 |
| Diphenylmethane | 266.10 | 304.80 | -38.70 | -14.54 |
| 2-Phenyltoluene | 275.77 | 304.80 | -29.03 | -10.53 |
| 1-Decene | 300.83 | 308.90 | -8.07 | -2.68 |
| 5-Methylnonane | 314.43 | 317.00 | -2.57 | -0.82 |
| 1-Ethyladamantane | 258.45 | 317.70 | -59.25 | -22.93 |
| 4-Methylnonane | 317.36 | 317.70 | -0.34 | -0.11 |
| 3-Methylnonane | 308.99 | 317.70 | -8.71 | -2.82 |
| Tolan | 297.50 | 318.30 | -20.80 | -6.99 |
| 2-Methylnonane | 313.30 | 318.40 | -5.10 | -1.63 |
| 1,3-Dimethyladamantane | 258.31 | 318.80 | -60.49 | -23.42 |
| Decane | 316.32 | 318.80 | -2.48 | -0.78 |
| cis,trans,trans-1,5,9-Cyclododecatriene | 287.76 | 319.10 | -31.34 | -10.89 |
| Hexamethylbenzene | 370.70 | 322.00 | 48.70 | 13.14 |
| 1,1-Diphenylethylene | 299.20 | 323.20 | -24.00 | -8.02 |
| trans-Stilbene | 343.10 | 323.40 | 19.70 | 5.74 |
| 1,2,3,4-Tetrahydrophenanthrene | 278.26 | 324.00 | -45.74 | -16.44 |
| 1,1,4,7-Tetramethylindane | 302.50 | 331.20 | -28.70 | -9.49 |
| 2-Ethylbiphenyl | 302.73 | 331.50 | -28.77 | -9.50 |
| 1,2-Diphenylethane | 320.10 | 333.10 | -13.00 | -4.06 |
| 3,3'-Bitolyl | 295.45 | 333.20 | -37.75 | -12.78 |
| 2-Methyldiphenylmethane | 296.58 | 334.00 | -37.42 | -12.62 |
| 2,2'-Dimethylbiphenyl | 298.06 | 334.00 | -35.94 | -12.06 |
| 1,1-Diphenylethane | 295.00 | 334.30 | -39.30 | -13.32 |
| 2,3-Dihydro-1,1,4,6-tetramethyl-1H-indene | 299.60 | 335.60 | -36.00 | -12.02 |
| Bicyclohexyl | 283.00 | 337.80 | -54.80 | -19.36 |
| 1-Undecene | 329.95 | 339.30 | -9.35 | -2.83 |
| 2-Methyldecane | 341.21 | 348.80 | -7.59 | -2.22 |
| Undecane | 345.05 | 349.10 | -4.05 | -1.17 |
| 4-Isopropylbiphenyl | 343.90 | 363.30 | -19.40 | -5.64 |
| 1-Dodecene | 360.66 | 369.70 | -9.04 | -2.51 |
| 2,2,4,6,6-Pentamethylheptane | 350.98 | 372.20 | -21.22 | -6.05 |
| Dodecane | 376.10 | 379.40 | -3.30 | -0.88 |
| 1,4-Di-t-butylbenzene | 347.84 | 383.10 | -35.26 | -10.14 |
| Heptylcyclohexane | 363.20 | 389.40 | -26.20 | -7.21 |
| 4-t-Butylbiphenyl | 383.60 | 391.40 | -7.80 | -2.03 |
| 1-Tridecene | 391.80 | 400.00 | -8.20 | -2.09 |
| m-Terphenyl | 417.10 | 401.60 | 15.50 | 3.72 |
| o-Terphenyl | 369.05 | 402.10 | -33.05 | -8.96 |
| Tridecane | 407.10 | 410.40 | -3.30 | -0.81 |
| Tetradecane | 438.90 | 440.00 | -1.10 | -0.25 |
| Decylcyclopentane | 426.52 | 450.20 | -23.68 | -5.55 |
| Pentadecane | 469.90 | 470.30 | -0.40 | -0.09 |
| Decylcyclohexane | 455.60 | 479.70 | -24.10 | -5.29 |
| 2,2,4,4,6,8,8-Heptamethylnonane | 458.80 | 488.00 | -29.20 | -6.36 |
| Cetene | 485.83 | 491.10 | -5.27 | -1.08 |
| Cetane | 501.61 | 500.60 | 1.01 | 0.20 |
| Hexaethylcyclohexane | 530.10 | 528.10 | 2.00 | 0.38 |
| Heptadecane | 534.34 | 531.00 | 3.34 | 0.63 |
| Octadecane | 564.40 | 561.30 | 3.10 | 0.55 |
| Pristane | 569.76 | 586.20 | -16.44 | -2.89 |
| Nonadecane | 604.00 | 591.60 | 12.40 | 2.05 |
| Eicosane | 663.60 | 622.90 | 40.70 | 6.13 |
| 1,1-Diphenyldodecane | 593.70 | 636.60 | -42.90 | -7.23 |
| 1-Phenyl-1-cyclohexyldodecane | 611.10 | 669.70 | -58.60 | -9.59 |
| Docosane | 698.00 | 683.70 | 14.30 | 2.05 |
| 4'-Heptyl-p-tercyclohexyl | 752.70 | 709.60 | 43.10 | 5.73 |
| 4'-Heptyl-m-tercyclohexyl | 668.60 | 709.60 | -41.00 | -6.13 |
| Pentacosane | 769.00 | 774.80 | -5.80 | -0.75 |
| 11-Cyclohexyleicosane | 787.40 | 781.00 | 6.40 | 0.81 |
| Heptacosane | 828.40 | 835.50 | -7.10 | -0.86 |
| Dodecahydrosqualene | 886.36 | 915.70 | -29.34 | -3.31 |
| 11-Decylheneicosane | 949.80 | 953.90 | -4.10 | -0.43 |
| Unatriacontane | 912.10 | 956.90 | -44.80 | -4.91 |
| **Mean** |  |  | **-14.38** | **-6.34** |
| **Standard deviation** |  |  | **22.10** | **9.42** |
