# Supplementary material for: Calculation of the Isobaric Heat Capacities of the Liquid and Solid Phase of Organic Compounds at and around 298.15 K Based on Their “True” Molecular Volume
Source: Molecules. 2019 Apr 24;24(8):1626. doi: 10.3390/molecules24081626 (PMC6514989; doi:10.3390/molecules24081626)
Supplement: Supplementary file 1 [file molecules-24-01626-s001.zip › Table 6.doc]

| **Molecule name** | **Vm** | **Cp(liq,298) exp** | **Cp(liq,298) calc** | **Deviation** | **Dev. in %** |
| --- | --- | --- | --- | --- | --- |
| Methanol | 36.90 | 81.92 | 94.46 | -12.54 | -15.31 |
| Formic acid | 39.90 | 99.04 | 100.25 | -1.21 | -1.22 |
| Ethanol | 53.60 | 115.90 | 126.66 | -10.76 | -9.28 |
| Acetic acid | 56.70 | 123.10 | 132.64 | -9.54 | -7.75 |
| Allyl alcohol | 65.10 | 146.30 | 148.84 | -2.54 | -1.74 |
| Colamine | 65.60 | 137.70 | 149.80 | -12.10 | -8.79 |
| Acrylic acid | 67.40 | 144.20 | 153.27 | -9.07 | -6.29 |
| 1-Propanol | 70.30 | 146.88 | 158.86 | -11.98 | -8.16 |
| 2-Propanol | 70.30 | 154.43 | 158.86 | -4.43 | -2.87 |
| Chloroacetic acid | 70.40 | 179.90 | 159.06 | 20.84 | 11.58 |
| Propanoic acid | 73.30 | 158.60 | 164.65 | -6.05 | -3.81 |
| Cyclobutanol | 78.10 | 153.70 | 173.90 | -20.20 | -13.14 |
| Methyl cellosolve | 80.10 | 176.40 | 177.76 | -1.36 | -0.77 |
| Hydroxymethyl acetate | 82.40 | 214.00 | 182.19 | 31.81 | 14.86 |
| Methacrylic acid | 84.00 | 161.10 | 185.28 | -24.18 | -15.01 |
| Dichloroacetic acid | 84.50 | 188.00 | 186.24 | 1.76 | 0.94 |
| 2-Methyl-1-propanol | 86.50 | 181.05 | 190.10 | -9.05 | -5.00 |
| 2-Butanol | 86.50 | 196.67 | 190.10 | 6.57 | 3.34 |
| 2-Methyl-2-propanol | 86.80 | 218.60 | 190.68 | 27.92 | 12.77 |
| 1-Butanol | 87.00 | 177.16 | 191.06 | -13.90 | -7.85 |
| 2-Methylpropanoic acid | 89.80 | 181.70 | 196.46 | -14.76 | -8.12 |
| Furfuranol | 89.80 | 204.01 | 196.46 | 7.55 | 3.70 |
| Butanoic acid | 89.90 | 177.70 | 196.66 | -18.96 | -10.67 |
| Phenol | 92.30 | 200.00 | 201.28 | -1.28 | -0.64 |
| Cyclopentanol | 93.40 | 185.40 | 203.40 | -18.00 | -9.71 |
| 2-Ethoxyethanol | 96.90 | 210.30 | 210.15 | 0.15 | 0.07 |
| Dimethylvinylcarbinol | 97.80 | 208.40 | 211.89 | -3.49 | -1.67 |
| Acetylacetone (enol form) | 98.60 | 208.40 | 213.43 | -5.03 | -2.41 |
| Cellosolve acetate | 98.70 | 203.00 | 213.62 | -10.62 | -5.23 |
| 2-Aminoisobutanol | 98.70 | 229.50 | 213.62 | 15.88 | 6.92 |
| 2-(Ethylamino)ethanol | 99.40 | 227.00 | 214.97 | 12.03 | 5.30 |
| Tetrahydrofurfuryl alcohol | 102.40 | 190.00 | 220.76 | -30.76 | -16.19 |
| 2-Methyl-2-butanol | 102.90 | 247.30 | 221.72 | 25.58 | 10.34 |
| Isopentyl alcohol | 103.00 | 209.60 | 221.91 | -12.31 | -5.87 |
| 3-Methyl-2-butanol | 103.10 | 245.90 | 222.11 | 23.79 | 9.67 |
| Neopentyl alcohol | 103.50 | 244.30 | 222.88 | 21.42 | 8.77 |
| 1-Pentanol | 103.60 | 208.14 | 223.07 | -14.93 | -7.17 |
| 3-Pentanol | 103.80 | 239.70 | 223.46 | 16.24 | 6.78 |
| 3-Methylbutanoic acid | 106.20 | 197.10 | 228.08 | -30.98 | -15.72 |
| Pentanoic acid | 106.60 | 210.00 | 228.86 | -18.86 | -8.98 |
| o-Cresol | 108.50 | 229.75 | 232.52 | -2.77 | -1.21 |
| Benzyl alcohol | 108.60 | 215.90 | 232.71 | -16.81 | -7.79 |
| p-Cresol | 108.80 | 221.03 | 233.10 | -12.07 | -5.46 |
| m-Cresol | 109.00 | 224.93 | 233.48 | -8.55 | -3.80 |
| Cyclohexanol | 109.50 | 213.40 | 234.45 | -21.05 | -9.86 |
| 2-Hydroxybenzaldehyde | 109.90 | 222.00 | 235.22 | -13.22 | -5.95 |
| 2-Isopropoxyethanol | 113.20 | 238.80 | 241.58 | -2.78 | -1.16 |
| 2-Propoxyethanol | 113.60 | 241.60 | 242.35 | -0.75 | -0.31 |
| cis-3-Hexen-1-ol | 114.30 | 234.00 | 243.70 | -9.70 | -4.15 |
| trans-3-Hexen-1-ol | 114.60 | 237.20 | 244.28 | -7.08 | -2.98 |
| 1-Hexen-3-ol | 114.60 | 269.30 | 244.28 | 25.02 | 9.29 |
| 2-(Isopropylamino)ethanol | 115.50 | 258.82 | 246.02 | 12.80 | 4.95 |
| Ethyl lactate | 115.80 | 254.00 | 246.60 | 7.40 | 2.91 |
| 2-Methoxyphenol | 118.10 | 240.00 | 251.03 | -11.03 | -4.60 |
| Tetrahydro-2H-pyran-2-methanol | 119.00 | 222.00 | 252.77 | -30.77 | -13.86 |
| 2-Ethyl-1-butanol | 119.30 | 246.65 | 253.34 | -6.69 | -2.71 |
| 3-Methyl-3-pentanol | 119.40 | 293.30 | 253.54 | 39.76 | 13.56 |
| 3,3-Dimethyl-1-butanol | 119.60 | 236.08 | 253.92 | -17.84 | -7.56 |
| 4-Methyl-2-pentanol | 119.70 | 272.34 | 254.12 | 18.22 | 6.69 |
| 3-Methyl-2-pentanol | 119.70 | 275.89 | 254.12 | 21.77 | 7.89 |
| 3-Hexanol | 119.90 | 269.27 | 254.50 | 14.77 | 5.49 |
| 2-Methyl-2-pentanol | 120.00 | 289.03 | 254.69 | 34.34 | 11.88 |
| 2-Methyl-1-pentanol | 120.10 | 248.40 | 254.89 | -6.49 | -2.61 |
| 4-Hydroxy-4-methyl-2-pentanone | 120.50 | 221.30 | 255.66 | -34.36 | -15.53 |
| 1-Hexanol | 120.60 | 242.70 | 255.85 | -13.15 | -5.42 |
| 2-Hexanol | 120.60 | 256.31 | 255.85 | 0.46 | 0.18 |
| n-Hexanoic acid | 123.30 | 225.10 | 261.06 | -35.96 | -15.98 |
| s-Phenethyl alcohol | 124.20 | 257.45 | 262.79 | -5.34 | -2.07 |
| Phenethyl alcohol | 124.50 | 252.64 | 263.37 | -10.73 | -4.25 |
| Cyclohexanemethanol | 125.30 | 236.50 | 264.91 | -28.41 | -12.01 |
| cis-2-Methylcyclohexanol | 125.30 | 268.95 | 264.91 | 4.04 | 1.50 |
| 1-Methylcyclohexanol | 125.30 | 279.05 | 264.91 | 14.14 | 5.07 |
| Cycloheptanol | 125.50 | 250.22 | 265.30 | -15.08 | -6.03 |
| trans-2-Methylcyclohexanol | 126.10 | 262.98 | 266.46 | -3.48 | -1.32 |
| 2,4-Dibromophenol | 129.10 | 259.40 | 272.24 | -12.84 | -4.95 |
| 2-t-Butoxyethanol | 129.40 | 273.45 | 272.82 | 0.63 | 0.23 |
| Butylcellosolve | 130.20 | 271.66 | 274.36 | -2.70 | -0.99 |
| 2-Diethylaminoethanol | 130.80 | 281.20 | 275.52 | 5.68 | 2.02 |
| 2-Phenoxyethanol | 134.80 | 294.63 | 283.23 | 11.40 | 3.87 |
| 1-Naphthol | 135.40 | 284.50 | 284.39 | 0.11 | 0.04 |
| Triethylmethanol | 135.50 | 353.90 | 284.58 | 69.32 | 19.59 |
| 2,4-Dimethyl-3-pentanol | 135.90 | 312.00 | 285.35 | 26.65 | 8.54 |
| 2,2-Dimethyl-3-pentanol | 135.90 | 349.00 | 285.35 | 63.65 | 18.24 |
| 2-Methyl-2-hexanol | 136.70 | 313.54 | 286.89 | 26.65 | 8.50 |
| 2-Methyl-3-hexanol | 136.70 | 324.00 | 286.89 | 37.11 | 11.45 |
| 5-Methyl-2-hexanol | 136.80 | 295.20 | 287.09 | 8.11 | 2.75 |
| 2-Heptanol | 137.20 | 298.63 | 287.86 | 10.77 | 3.61 |
| 4-Heptanol | 137.20 | 306.77 | 287.86 | 18.91 | 6.16 |
| 3-Heptanol | 137.20 | 314.20 | 287.86 | 26.34 | 8.38 |
| 1-Heptanol | 137.30 | 274.81 | 288.05 | -13.24 | -4.82 |
| Enanthic acid | 139.80 | 267.31 | 292.87 | -25.56 | -9.56 |
| Hydrocinnamyl alcohol | 141.10 | 280.74 | 295.38 | -14.64 | -5.21 |
| Cyclohexaneethanol | 142.50 | 266.00 | 298.08 | -32.08 | -12.06 |
| 2-Hydroxyethyl-2',2'-dimethylpropionate | 148.90 | 308.00 | 310.42 | -2.42 | -0.79 |
| 4-Ethyl-3-hexanol | 152.20 | 361.30 | 316.78 | 44.52 | 12.32 |
| Ethyl salicylate | 152.70 | 283.07 | 317.75 | -34.68 | -12.25 |
| 4-Methyl-2-heptanol | 152.70 | 312.50 | 317.75 | -5.25 | -1.68 |
| 3-Methyl-2-heptanol | 152.80 | 297.50 | 317.94 | -20.44 | -6.87 |
| 4-Methyl-3-heptanol | 152.80 | 309.20 | 317.94 | -8.74 | -2.83 |
| 3-Methyl-4-heptanol | 152.80 | 355.80 | 317.94 | 37.86 | 10.64 |
| 2,5-Dimethyl-3-hexanol | 152.90 | 339.40 | 318.13 | 21.27 | 6.27 |
| 2-Ethyl-1-hexanol | 153.00 | 317.50 | 318.32 | -0.82 | -0.26 |
| 4-Methyl-4-heptanol | 153.00 | 366.90 | 318.32 | 48.58 | 13.24 |
| 5-Methyl-2-heptanol | 153.10 | 296.20 | 318.52 | -22.32 | -7.54 |
| 2-Methyl-4-heptanol | 153.10 | 331.80 | 318.52 | 13.28 | 4.00 |
| 5-Methyl-1-heptanol | 153.20 | 304.20 | 318.71 | -14.51 | -4.77 |
| 6-Methyl-3-heptanol | 153.40 | 310.50 | 319.10 | -8.60 | -2.77 |
| 2-Methyl-2-heptanol | 153.40 | 337.60 | 319.10 | 18.50 | 5.48 |
| 2-Methyl-1-heptanol | 153.50 | 313.00 | 319.29 | -6.29 | -2.01 |
| 6-Methyl-2-heptanol | 153.50 | 315.10 | 319.29 | -4.19 | -1.33 |
| 1-Octanol | 153.70 | 312.10 | 319.67 | -7.57 | -2.43 |
| 4-Octanol | 153.90 | 332.09 | 320.06 | 12.03 | 3.62 |
| 3-Octanol | 153.90 | 338.50 | 320.06 | 18.44 | 5.45 |
| 2-Octanol | 154.00 | 330.10 | 320.25 | 9.85 | 2.98 |
| N-Methyl-2-hydroxyethylammonium propionate | 154.40 | 328.00 | 321.02 | 6.98 | 2.13 |
| n-Octanoic acid | 156.60 | 304.00 | 325.27 | -21.27 | -7.00 |
| Cyclohexanepropanol | 159.20 | 293.00 | 330.28 | -37.28 | -12.72 |
| 4-Allyl-2-methoxyphenol | 161.80 | 343.10 | 335.29 | 7.81 | 2.28 |
| 2-(2-(2-Methoxyethoxy)ethoxy)ethanol | 166.10 | 357.05 | 343.58 | 13.47 | 3.77 |
| 1-Ethyl-3-methylimidazolium hydrogen sulfate | 169.60 | 295.50 | 350.33 | -54.83 | -18.55 |
| 3,7-Dimethyl-6-octen-1-yn-3-ol | 170.00 | 385.30 | 351.10 | 34.20 | 8.88 |
| 4-Nonanol | 170.60 | 367.86 | 352.26 | 15.60 | 4.24 |
| 5-Nonanol | 170.60 | 370.75 | 352.26 | 18.49 | 4.99 |
| 3-Nonanol | 170.60 | 373.63 | 352.26 | 21.37 | 5.72 |
| 1-Nonanol | 170.70 | 341.00 | 352.45 | -11.45 | -3.36 |
| 2-Nonanol | 170.70 | 356.32 | 352.45 | 3.87 | 1.09 |
| N-Methyl-2-hydroxyethylammonium butanoate | 171.10 | 361.00 | 353.22 | 7.78 | 2.16 |
| trans-Geraniol | 172.60 | 346.10 | 356.12 | -10.02 | -2.90 |
| Pelargonic acid | 173.20 | 362.37 | 357.27 | 5.10 | 1.41 |
| Diethyleneglycol monobutyl ether | 173.30 | 354.89 | 357.47 | -2.58 | -0.73 |
| Linalool | 174.20 | 372.40 | 359.20 | 13.20 | 3.54 |
| beta-Citronellol | 179.30 | 357.90 | 369.04 | -11.14 | -3.11 |
| 1-Ethyl-3-methylimidazolium methylphosphonate | 183.60 | 354.64 | 377.33 | -22.69 | -6.40 |
| 4-Propyl-4-heptanol | 185.70 | 446.60 | 381.38 | 65.22 | 14.60 |
| 3,7-Dimethyl-1-octanol | 186.10 | 367.21 | 382.15 | -14.94 | -4.07 |
| n-Decyl alcohol | 187.00 | 377.00 | 383.88 | -6.88 | -1.82 |
| 5-Decanol | 187.30 | 405.77 | 384.46 | 21.31 | 5.25 |
| N-Methyl-2-hydroxyethylammonium pentanoate | 187.80 | 401.00 | 385.43 | 15.57 | 3.88 |
| n-Decanoic acid | 189.90 | 361.10 | 389.48 | -28.38 | -7.86 |
| 2-(2'-Hydroxyethoxy)ethyl pivalate | 191.90 | 368.83 | 393.33 | -24.50 | -6.64 |
| 1-(2-Hydroxyethyl)-3-methyl-1H-imidazolium 2,2,2-trifluoroacetate | 192.20 | 369.00 | 393.91 | -24.91 | -6.75 |
| 1-Undecanol | 204.10 | 406.34 | 416.86 | -10.52 | -2.59 |
| n-Undecanoic acid | 206.50 | 415.10 | 421.48 | -6.38 | -1.54 |
| 1-Dodecanol | 220.80 | 438.42 | 449.06 | -10.64 | -2.43 |
| 1H,1H-Perfluorooctan-1-ol | 222.70 | 468.60 | 452.72 | 15.88 | 3.39 |
| Lauric acid | 223.20 | 404.28 | 453.68 | -49.40 | -12.22 |
| Tributylmethanol | 235.70 | 548.60 | 477.79 | 70.81 | 12.91 |
| 1-Tridecanol | 237.50 | 476.00 | 481.26 | -5.26 | -1.11 |
| 1H,1H-Perfluorononan-1-ol | 250.30 | 515.90 | 505.94 | 9.96 | 1.93 |
| Pentaethylene glycol monomethyl ether | 252.10 | 537.12 | 509.41 | 27.71 | 5.16 |
| Myristyl alcohol | 254.20 | 505.80 | 513.46 | -7.66 | -1.51 |
| 3,7,11-Trimethyl-1-dodecen-3-ol | 262.20 | 574.50 | 528.88 | 45.62 | 7.94 |
| 1-Pentadecanol | 270.90 | 535.10 | 545.66 | -10.56 | -1.97 |
| 1H,1H-Perfluorodecan-1-ol | 274.50 | 563.90 | 552.60 | 11.30 | 2.00 |
| 1-Hexadecanol | 287.60 | 523.80 | 577.86 | -54.06 | -10.32 |
| 1H,1H-Perfluoroundecan-1-ol | 302.10 | 602.40 | 605.82 | -3.42 | -0.57 |
| (9Z)-Octadecenoic acid | 317.00 | 577.00 | 634.55 | -57.55 | -9.97 |
| 1H,1H-Perfluorododecan-1-ol | 328.00 | 645.20 | 655.76 | -10.56 | -1.64 |
| Methyltributylammonium serinate | 331.10 | 635.00 | 661.74 | -26.74 | -4.21 |
| 3,7,11,15-Tetramethyl-1-hexadecyn-3-ol | 340.80 | 712.50 | 680.44 | 32.06 | 4.50 |
| Isophytol | 344.10 | 729.70 | 686.80 | 42.90 | 5.88 |
| Heptylpentaoxyethylene | 352.40 | 722.90 | 702.81 | 20.09 | 2.78 |
| 1H,1H-Perfluorotetradecan-1-ol | 379.80 | 725.30 | 755.64 | -30.34 | -4.18 |
| Tetrabutylphosphonium L-serinate | 389.50 | 748.00 | 774.34 | -26.34 | -3.52 |
