# Supplementary material for: Calculation of the Isobaric Heat Capacities of the Liquid and Solid Phase of Organic Compounds at and around 298.15 K Based on Their “True” Molecular Volume
Source: Molecules. 2019 Apr 24;24(8):1626. doi: 10.3390/molecules24081626 (PMC6514989; doi:10.3390/molecules24081626)
Supplement: Supplementary file 1 [file molecules-24-01626-s001.zip › Table 7.doc]

| **Molecule name** | **Vm** | **Cp(liq,298) exp** | **Cp(liq,298) calc** | **Deviation** | **Dev. in %** |
| --- | --- | --- | --- | --- | --- |
| Ethan-1,2-diol | 62.7 | 149.80 | 151.62 | -1.82 | -1.21 |
| 1,2-Propanediol | 79.4 | 180.30 | 185.73 | -5.43 | -3.01 |
| Propan-1,3-diol | 79.4 | 175.78 | 185.73 | -9.95 | -5.66 |
| DL-Lactic acid | 81.8 | 210.50 | 190.63 | 19.87 | 9.44 |
| Lactic acid | 81.8 | 210.50 | 190.63 | 19.87 | 9.44 |
| 1,2,3-Propanetriol | 88.2 | 218.90 | 203.70 | 15.20 | 6.94 |
| 2,3-Butyleneglycol | 96.0 | 213.00 | 219.63 | -6.63 | -3.11 |
| 1,2-Butanediol | 96.0 | 230.82 | 219.63 | 11.19 | 4.85 |
| 1,3-Butyleneglycol | 96.1 | 218.89 | 219.83 | -0.94 | -0.43 |
| Tetramethylene glycol | 96.1 | 203.79 | 219.83 | -16.04 | -7.87 |
| Butanedioic acid | 100.9 | 238.60 | 229.64 | 8.96 | 3.76 |
| Diethyleneglycol | 105.8 | 243.90 | 239.64 | 4.26 | 1.75 |
| 1,5-Pentanediol | 112.8 | 232.49 | 253.94 | -21.45 | -9.23 |
| Pentanedioic acid | 117.6 | 270.50 | 263.74 | 6.76 | 2.50 |
| Hexyleneglycol | 126.6 | 263.10 | 282.12 | -19.02 | -7.23 |
| 1,2-Hexanediol | 129.4 | 293.10 | 287.84 | 5.26 | 1.79 |
| Dipropylene glycol | 138.8 | 322.10 | 307.04 | 15.06 | 4.68 |
| 1,7-Heptanediol | 146.2 | 297.00 | 322.15 | -25.15 | -8.47 |
| Triethyleneglycol | 148.8 | 327.60 | 327.46 | 0.14 | 0.04 |
| Heptanedioic acid | 151.0 | 334.30 | 331.95 | 2.35 | 0.70 |
| Octanedioic acid | 167.2 | 366.20 | 365.03 | 1.17 | 0.32 |
| Nonanedioic acid | 184.4 | 398.10 | 400.16 | -2.06 | -0.52 |
| Tetraglycol | 191.8 | 428.90 | 415.27 | 13.63 | 3.18 |
| Decanedioic acid | 201.1 | 430.00 | 434.27 | -4.27 | -0.99 |
| Tripropylene glycol | 214.3 | 440.60 | 461.22 | -20.62 | -4.68 |
| Undecanedioic acid | 217.8 | 461.90 | 468.37 | -6.47 | -1.40 |
| Dodecanedioic acid | 234.5 | 493.80 | 502.48 | -8.68 | -1.76 |
| Pentaglycol | 234.8 | 515.50 | 503.09 | 12.41 | 2.41 |
| 1,13-Tridecanedioic acid | 251.2 | 525.70 | 536.58 | -10.88 | -2.07 |
| Tetrapropylene glycol | 255.3 | 559.80 | 544.95 | 14.85 | 2.65 |
| 1,14-Tetradecanedioic acid | 267.9 | 557.60 | 570.68 | -13.08 | -2.35 |
| Hexaethylene glycol | 277.8 | 620.10 | 590.90 | 29.20 | 4.71 |
| Hexadecanedioic acid | 301.3 | 621.40 | 638.89 | -17.49 | -2.81 |
| Pentapropylene glycol | 312.4 | 685.80 | 661.56 | 24.24 | 3.53 |
| Ricinelaidic acid | 325.8 | 646.00 | 688.93 | -42.93 | -6.65 |
| Hexapropylene glycol | 369.9 | 807.50 | 778.99 | 28.51 | 3.53 |
