# Supplementary material for: Calculation of the Isobaric Heat Capacities of the Liquid and Solid Phase of Organic Compounds at and around 298.15 K Based on Their “True” Molecular Volume
Source: Molecules. 2019 Apr 24;24(8):1626. doi: 10.3390/molecules24081626 (PMC6514989; doi:10.3390/molecules24081626)
Supplement: Supplementary file 1 [file molecules-24-01626-s001.zip › Table 8.doc]

| **Molecules class** | **N** | **Corr. coeff. R^2^** | **σ (J/mol/K)** | **Intercept** | **Slope** |
| --- | --- | --- | --- | --- | --- |
| OH-free compounds | 1102 | 0.9890 | 20.70 | -5.3055 | 1.8183 |
| All alcohols/acids | 201 | 0. 9724 | 23.24 | 20.9141 | 1.9676 |
| Monoalcohols/-acids | 164 | 0. 9685 | 22.91 | 23.3101 | 1.9282 |
| Polyalcohols/-acids | 36 | 0.9910 | 16.03 | 23.5782 | 2.0422 |
