# Supplementary material for: Calculation of the Isobaric Heat Capacities of the Liquid and Solid Phase of Organic Compounds at and around 298.15 K Based on Their “True” Molecular Volume
Source: Molecules. 2019 Apr 24;24(8):1626. doi: 10.3390/molecules24081626 (PMC6514989; doi:10.3390/molecules24081626)
Supplement: Supplementary file 1 [file molecules-24-01626-s001.zip › Table 9.doc]

| **Molecule name** | **Vm** | **Cp(sol,298) exp** | **Cp(sol,298) calc** | **Deviation** | **Dev. in %** |
| --- | --- | --- | --- | --- | --- |
| 2-Methyl-2-propanol | 86.80 | 146.11 | 119.41 | 26.70 | 18.27 |
| Serine | 91.30 | 135.60 | 126.18 | 9.42 | 6.94 |
| Phenol | 92.30 | 127.44 | 127.69 | -0.25 | -0.20 |
| Dibromoacetic acid | 92.80 | 124.70 | 128.44 | -3.74 | -3.00 |
| 2-Furoic acid | 92.90 | 132.26 | 128.59 | 3.67 | 2.77 |
| cis-3-Chloro-2-butenoic acid | 96.20 | 140.20 | 133.56 | 6.64 | 4.74 |
| 4-Fluorophenol | 97.20 | 144.60 | 135.06 | 9.54 | 6.59 |
| trans-3-Chloro-2-butenoic acid | 97.20 | 159.80 | 135.06 | 24.74 | 15.48 |
| 3-Thiophenecarboxylic acid | 99.70 | 134.17 | 138.83 | -4.66 | -3.47 |
| 2-Thiophenecarboxylic acid | 100.00 | 153.78 | 139.28 | 14.50 | 9.43 |
| 2-Pyrazinecarboxylic acid | 102.50 | 143.50 | 143.04 | 0.46 | 0.32 |
| 5-Methylisoxazole-3-carboxylic acid | 105.30 | 146.20 | 147.25 | -1.05 | -0.72 |
| Trimethylacetic acid | 106.20 | 177.80 | 148.61 | 29.19 | 16.42 |
| Nicotinic acid | 107.00 | 151.30 | 149.81 | 1.49 | 0.98 |
| 2-Pyridinealdoxime | 108.00 | 151.93 | 151.32 | 0.61 | 0.40 |
| o-Cresol | 108.50 | 154.56 | 152.07 | 2.49 | 1.61 |
| p-Cresol | 108.80 | 150.20 | 152.52 | -2.32 | -1.55 |
| Threonine | 109.30 | 155.31 | 153.27 | 2.04 | 1.31 |
| 4-Amino-3-furazanecarboxamidoxime | 109.70 | 180.40 | 153.88 | 26.52 | 14.70 |
| Aspartic acid | 110.50 | 155.18 | 155.08 | 0.10 | 0.06 |
| Benzoic acid | 111.10 | 147.07 | 155.98 | -8.91 | -6.06 |
| 4-Hydroxybenzaldehyde | 111.50 | 149.90 | 156.59 | -6.69 | -4.46 |
| Cyclohexanone oxime | 114.90 | 199.38 | 161.70 | 37.68 | 18.90 |
| 4-Nitrophenol | 115.40 | 143.90 | 162.45 | -18.55 | -12.89 |
| 3-Thiopheneacetic acid | 115.70 | 167.76 | 162.91 | 4.85 | 2.89 |
| 2-Thiopheneacetic acid | 116.50 | 159.23 | 164.11 | -4.88 | -3.06 |
| Pentafluorophenol | 116.80 | 201.30 | 164.56 | 36.74 | 18.25 |
| 3,5-Dimethylisoxazole-4-carboxylic acid | 120.40 | 177.80 | 169.98 | 7.82 | 4.40 |
| o-Anthranilic acid | 121.00 | 165.30 | 170.88 | -5.58 | -3.38 |
| m-Anthranilic acid | 123.50 | 162.80 | 174.64 | -11.84 | -7.28 |
| p-Anthranilic acid | 123.60 | 177.80 | 174.80 | 3.00 | 1.69 |
| 4-Ethylphenol | 124.50 | 206.90 | 176.15 | 30.75 | 14.86 |
| 2-Chlorobenzoic acid | 124.70 | 163.20 | 176.45 | -13.25 | -8.12 |
| 2-Methylbenzoic acid | 125.70 | 174.90 | 177.96 | -3.06 | -1.75 |
| 3-Chlorobenzoic acid | 125.70 | 163.60 | 177.96 | -14.36 | -8.77 |
| 4-Chlorobenzoic acid | 125.70 | 167.80 | 177.96 | -10.16 | -6.05 |
| 3-Methylbenzoic acid | 127.80 | 163.60 | 181.12 | -17.52 | -10.71 |
| 4-Methylbenzoic acid | 127.80 | 169.00 | 181.12 | -12.12 | -7.17 |
| 2-Bromobenzoic acid | 128.60 | 154.00 | 182.32 | -28.32 | -18.39 |
| Glutamic acid | 128.70 | 175.06 | 182.47 | -7.41 | -4.23 |
| Benzoylformic acid | 129.60 | 192.90 | 183.82 | 9.08 | 4.70 |
| 3-Bromobenzoic acid | 129.90 | 151.40 | 184.28 | -32.88 | -21.71 |
| 4-Bromobenzoic acid | 129.90 | 151.40 | 184.28 | -32.88 | -21.71 |
| 2-Nitrobenzoic acid | 130.70 | 191.60 | 185.48 | 6.12 | 3.19 |
| 8-Quinolinol | 130.90 | 180.42 | 185.78 | -5.36 | -2.97 |
| 3-Phenylpropiolic acid | 134.60 | 180.00 | 191.35 | -11.35 | -6.31 |
| 3-Nitrobenzoic acid | 134.70 | 179.90 | 191.50 | -11.60 | -6.45 |
| Benzofuran-2-carboxylic acid | 134.70 | 184.80 | 191.50 | -6.70 | -3.63 |
| 4-Nitrobenzoic acid | 134.80 | 180.30 | 191.65 | -11.35 | -6.30 |
| 2-Naphthol | 135.30 | 172.80 | 192.40 | -19.60 | -11.34 |
| 1-Naphthol | 135.40 | 166.90 | 192.55 | -25.65 | -15.37 |
| Methylsalicylate | 136.00 | 244.30 | 193.46 | 50.84 | 20.81 |
| Tyramine | 136.30 | 213.75 | 193.91 | 19.84 | 9.28 |
| 4-Hydroxy-3-methoxybenzaldehyde | 137.10 | 187.60 | 195.11 | -7.51 | -4.00 |
| 4-Methoxybenzoic acid | 137.20 | 207.50 | 195.26 | 12.24 | 5.90 |
| 2-Hydroxyacetanilide | 137.60 | 182.40 | 195.86 | -13.46 | -7.38 |
| Methyl 3-hydroxybenzoate | 137.60 | 205.00 | 195.86 | 9.14 | 4.46 |
| Methyl 4-hydroxybenzoate | 137.70 | 214.41 | 196.01 | 18.40 | 8.58 |
| trans-Cinnamic acid | 138.60 | 197.50 | 197.37 | 0.13 | 0.07 |
| Indole-2-carboxylic acid | 138.90 | 178.40 | 197.82 | -19.42 | -10.89 |
| N-Phenylglycine | 139.40 | 177.40 | 198.57 | -21.17 | -11.94 |
| Mesitol | 140.20 | 189.60 | 199.78 | -10.18 | -5.37 |
| 3-Trifluoromethylbenzoic acid | 142.20 | 223.60 | 202.79 | 20.81 | 9.31 |
| Homocubane-4-carboxylic acid | 143.50 | 207.00 | 204.74 | 2.26 | 1.09 |
| Thionaphthene-2-carboxylic acid | 143.60 | 179.00 | 204.89 | -25.89 | -14.47 |
| 2,5-Dibromobenzoic acid | 147.00 | 223.00 | 210.01 | 12.99 | 5.82 |
| 2,4,6-Tribromophenol | 147.20 | 172.00 | 210.31 | -38.31 | -22.27 |
| Trimellitic anhydride | 147.30 | 248.90 | 210.46 | 38.44 | 15.44 |
| 3,5-Dibromobenzoic acid | 148.30 | 223.10 | 211.97 | 11.13 | 4.99 |
| 3-Aminocinnamic acid | 150.00 | 227.60 | 214.53 | 13.07 | 5.74 |
| 2-Adamantanol | 153.60 | 207.20 | 219.94 | -12.74 | -6.15 |
| Ethyl vanillin | 153.90 | 212.20 | 220.40 | -8.20 | -3.86 |
| 1-Adamantanol | 154.00 | 196.70 | 220.55 | -23.85 | -12.12 |
| Ethyl 3-hydroxybenzoate | 154.40 | 217.22 | 221.15 | -3.93 | -1.81 |
| Ethyl 4-hydroxybenzoate | 154.40 | 233.93 | 221.15 | 12.78 | 5.46 |
| D(+)-Carnitine | 155.40 | 224.70 | 222.65 | 2.05 | 0.91 |
| Hippuric acid | 158.40 | 217.00 | 227.17 | -10.17 | -4.69 |
| 2,4,6-Trinitrophenol | 160.00 | 239.70 | 229.58 | 10.12 | 4.22 |
| 2-Nitrocinnamic acid | 160.10 | 240.60 | 229.73 | 10.87 | 4.52 |
| 2-Hydroxybiphenyl | 161.50 | 227.61 | 231.83 | -4.22 | -1.86 |
| Pentachlorophenol | 161.80 | 201.96 | 232.28 | -30.32 | -15.02 |
| 3-Nitrocinnamic acid | 161.90 | 240.20 | 232.43 | 7.77 | 3.23 |
| 4-Nitrocinnamic acid | 161.90 | 238.10 | 232.43 | 5.67 | 2.38 |
| Isoborneol | 163.20 | 261.06 | 234.39 | 26.67 | 10.22 |
| Tyrosine | 164.40 | 216.44 | 236.20 | -19.76 | -9.13 |
| 2,3,5,6-Tetramethylbenzoic acid | 172.80 | 231.70 | 248.84 | -17.14 | -7.40 |
| 2,3,4,5-Tetramethylbenzoic acid | 174.40 | 249.50 | 251.25 | -1.75 | -0.70 |
| Menthol | 174.80 | 250.10 | 251.85 | -1.75 | -0.70 |
| Diphenylmethanol | 179.30 | 236.80 | 258.62 | -21.82 | -9.22 |
| p-Methacryloyloxybenzoic acid | 182.30 | 257.90 | 263.14 | -5.24 | -2.03 |
| 3-Cyano-4-methoxymethyl-5-nitro-6-methyl-(2-pyridone) | 183.90 | 275.80 | 265.54 | 10.26 | 3.72 |
| N-Methylephedrine | 184.70 | 298.89 | 266.75 | 32.14 | 10.75 |
| 9-Hydroxy-1,4-anthraquinone | 188.80 | 237.67 | 272.92 | -35.25 | -14.83 |
| Pentamethylbenzoic acid | 190.00 | 249.90 | 274.72 | -24.82 | -9.93 |
| Benzoylglycylglycine | 206.00 | 277.40 | 298.80 | -21.40 | -7.72 |
| 5-Hydroxyflavone | 206.70 | 279.80 | 299.86 | -20.06 | -7.17 |
| 3-Hydroxyflavone | 207.00 | 270.10 | 300.31 | -30.21 | -11.18 |
| Ibuprofen | 208.20 | 328.01 | 302.11 | 25.90 | 7.89 |
| 6-Hydroxyflavone | 208.80 | 250.30 | 303.02 | -52.72 | -21.06 |
| 7-Hydroxyflavone | 208.80 | 253.10 | 303.02 | -49.92 | -19.72 |
| N-Methylephedrine hydrochloride | 210.80 | 282.29 | 306.03 | -23.74 | -8.41 |
| 2-(6-Methoxynaphthalen-2-yl)propanoic acid | 211.90 | 296.30 | 307.68 | -11.38 | -3.84 |
| PMC | 220.10 | 315.30 | 320.02 | -4.72 | -1.50 |
| 4-(Phenylmethoxy)benzeneacetic acid | 224.60 | 340.00 | 326.80 | 13.20 | 3.88 |
| Tri-t-butylmethanol | 225.60 | 350.60 | 328.30 | 22.30 | 6.36 |
| 1-Tridecanol | 237.50 | 378.00 | 346.21 | 31.79 | 8.41 |
| Tridecanoic acid | 239.90 | 387.60 | 349.82 | 37.78 | 9.75 |
| Triphenylmethanol | 248.60 | 318.80 | 362.91 | -44.11 | -13.84 |
| Myristyl alcohol | 254.20 | 388.00 | 371.34 | 16.66 | 4.29 |
| Tetradecanoic acid | 256.60 | 432.01 | 374.95 | 57.06 | 13.21 |
| 1-Pentadecanol | 270.90 | 400.00 | 396.48 | 3.52 | 0.88 |
| 1-Pentadecanoic acid | 273.30 | 443.28 | 400.09 | 43.19 | 9.74 |
| N-(2-Hydroxy-4-methoxybenzylidene)-4-butylaniline | 275.20 | 451.00 | 402.95 | 48.05 | 10.65 |
| 1-Hexadecanol | 287.60 | 422.00 | 421.61 | 0.39 | 0.09 |
| Palmitic acid | 290.00 | 460.70 | 425.22 | 35.48 | 7.70 |
| Margaric acid | 306.70 | 475.70 | 450.35 | 25.35 | 5.33 |
| Stearic acid | 323.40 | 501.60 | 475.49 | 26.11 | 5.21 |
| 1-Nonadecanoic acid | 340.10 | 525.40 | 500.62 | 24.78 | 4.72 |
| Arachidic acid | 356.80 | 545.10 | 525.75 | 19.35 | 3.55 |
| N-Formylmethionine-leucyl-phenylalanine | 403.70 | 567.60 | 596.33 | -28.73 | -5.06 |
| Simvastatin | 408.40 | 577.00 | 603.41 | -26.41 | -4.58 |
| Pyrimethanil laurate | 411.70 | 584.50 | 608.37 | -23.87 | -4.08 |
| Methylprednisolone aceponate | 426.90 | 604.80 | 631.25 | -26.45 | -4.37 |
